# Exploring the Dispersion and Electrostatic Components in Arene–Arene Interactions between Ligands and G4 DNA to Develop G4-Ligands

**DOI:** 10.1021/acs.jmedchem.3c02127

**Published:** 2024-01-19

**Authors:** Måns Andreasson, Maxime Donzel, Alva Abrahamsson, Andreas Berner, Mara Doimo, Anna Quiroga, Anna Eriksson, Yu-Kai Chao, Jeroen Overman, Nils Pemberton, Sjoerd Wanrooij, Erik Chorell

**Affiliations:** † Chemical Biology Consortium Sweden, Department of Chemistry, Umeå University, 901 87 Umeå, Sweden; ‡ Departments of Medical Biochemistry and Biophysics, Umeå University, Umeå 90736, Sweden; § Clinical Genetics Unit, Department of Women and Children’s Health, Padua University, 35128 Padua, Italy; ∥ Mechanistic and Structural Biology, Discovery Sciences, R&D, AstraZeneca, Cambridge CB2 0AA, U.K.; ⊥ Medicinal Chemistry, Research and Early Development, Respiratory and Immunology (R&I), Bio Pharmaceuticals R&D, AstraZeneca, Gothenburg SE-43183, Sweden

## Abstract

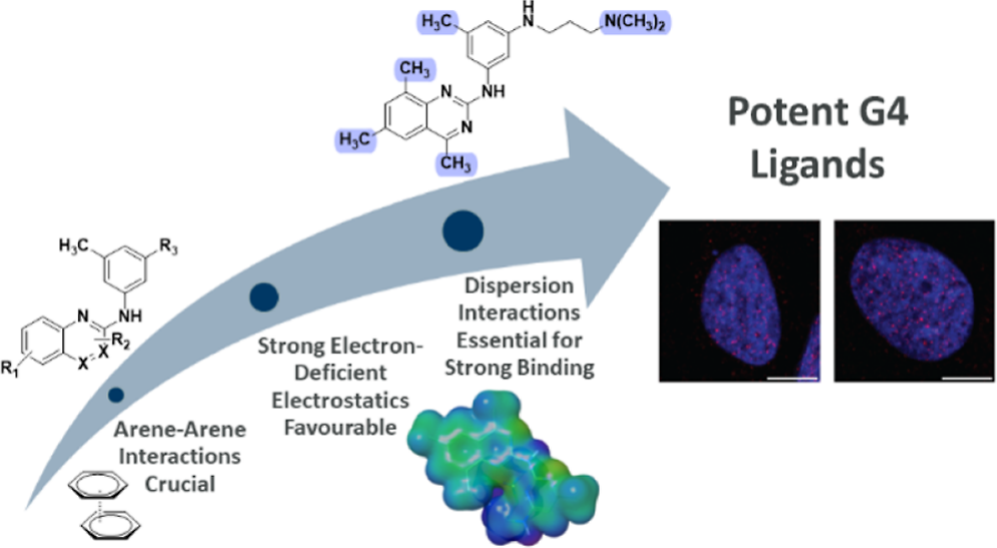

G-Quadruplex (G4) DNA structures are important regulatory elements in central biological processes. Small molecules that selectively bind and stabilize G4 structures have therapeutic potential, and there are currently >1000 known G4 ligands. Despite this, only two G4 ligands ever made it to clinical trials. In this work, we synthesized several heterocyclic G4 ligands and studied their interactions with G4s (e.g., G4s from the *c-MYC*, *c-KIT*, and *BCL-2* promoters) using biochemical assays. We further studied the effect of selected compounds on cell viability, the effect on the number of G4s in cells, and their pharmacokinetic properties. This identified potent G4 ligands with suitable properties and further revealed that the dispersion component in arene–arene interactions in combination with electron-deficient electrostatics is central for the ligand to bind with the G4 efficiently. The presented design strategy can be applied in the further development of G4-ligands with suitable properties to explore G4s as therapeutic targets.

## Introduction

Interest in the secondary DNA structures known as G-quadruplex structures (G4s) grows continuously as their central roles in biological processes become increasingly evident.^1,2^ G4 structures can assemble in guanine-rich nucleotide sequences of DNA by self-stacking off the guanine bases through arene–arene interactions and the coordination between K^+^ or other cations with the carbonyl oxygens of the guanines. The structures are further stabilized by internal Hoogsteen hydrogen bonding between the guanines in each plane (quartet) of the G4 structure.^3^ These structures possess considerable thermodynamic stability, similar to or higher than duplex DNA,^4^ and their folding kinetics can be rapid.^5^ Disruption of the duplex DNA is necessary to enable G4 formation, hence their formation is favored during events that cause a local disruption of the traditional Watson–Crick base pairing, such as during transcription, replication, DNA damage repair, or during negative supercoiling.^2^

The locations of G4 structures are highly conserved in the human genome, suggesting their involvement in central biological processes in vivo.^6^ It is estimated that there are hundreds of thousands of sequences capable of forming G4 structures in the human genome,^7^ and G4s are abundant in promotor regions of oncogenes.^8−10^ In these specific loci, G4 structures play a crucial role in the transcriptional regulation of oncogenes, with one prominent example being the c-MYC gene.^11^ The *c-MYC* gene is upregulated in about 70% of all types of human cancers,^12^ and suppressing c-MYC expression has thus emerged as a promising strategy to impede cancer progression.^11^ The transcriptional regulation of *c-MYC* is foremost controlled by the guanine-rich nuclease hypersensitivity element III_1_,^11^ which contains a G4-forming sequence (Pu27) that can be represented by the mutated sequences Pu22 and Pu24T.^13−15^ When the sequence is folded into a G4 structure, the expression of the c-MYC protein is silenced.^15^ Compounds that bind and stabilize the G4 structure in the *c-MYC* promotor region has been shown to downregulate the c-MYC protein, thereby reducing cancer growth.^16^ Targeting the *c-MYC* G4 is therefore considered a promising anticancer strategy, especially considering the difficulties in targeting the MYC protein itself.^17^ Beyond c-MYC, several other oncogenes are intricately connected to G4-mediated regulation, offering potential avenues for innovative cancer treatments. Examples include *cKIT*,^18,19^*KRas*,^20^ and *BCL2*,^21^ among others.^10^

The design and discovery of small organic molecules that target G4 structures is an attractive area of research.^15,16,22−25^ Known G4 ligands often contain permanently charged species or multiple basic amine residues,^16,22^ and the advances of G4 ligands into selective G4 binders with satisfactory pharmacokinetic properties are still scarce.^25,26^ This hampers further elucidations of G4s as drug targets and can be linked to the lack of detailed descriptors of the interaction between G4 DNA and G4-ligands. Current guidelines in G4 ligand design propose that rigid aromatic systems can engage in stacking interactions with the guanines on the G4 surface, electron-withdrawing groups can enhance binding, and cationic groups can engage in electrostatic interactions with the phosphate backbone.^16,22^ Gaining a deeper and more detailed understanding of these binding interactions is essential to advance the development of G4 ligands with properties suitable to explore G4 DNA as drug targets.^16,22^

Arene–arene interactions (also known as π–π-stacking interactions) are vital in biological interactions including the interactions between DNA and organic molecules.^27,28^ The two main energetic components of arene–arene interactions are dispersion and electrostatics.^29^ Dispersion is usually the dominant energetic component in biological systems, and the interactions typically become more favorable the more substituents the interacting arenes have, regardless of their electronic (donating or withdrawing) character. Dispersion interactions are direct interactions between substituents on one arene with the other arene partner.^29−31^ The electrostatic component is affected by a local dipole through space that substituents impose on the overall electrostatic potential of the arene in the molecule as a whole, frequently depicted by ESP (electrostatic potential) maps.^30,31^ The importance of the electrostatic component for the arene–arene interactions depends on the electronic nature of the other arene partner.^27^ Generally, either electron-rich or -deficient electrostatics will be more beneficial for the binding.^29,31^

Incorporating charged cationic species is a common and important element in G4 ligand design, which often is ascribed to their ability to interact with the negatively charged phosphate backbone.^16,22^ These interactions suggest that ion-pairing between the ligands and the phosphate backbone, situated in a solvent-exposed aqueous environment, could lead to favorable binding interactions. However, the formation of salt bridges in solvent-exposed areas contributes very little, or nothing, to the overall binding free energy due to the high desolvation costs of the charged residues.^32−34^ Consequently, the rationale that such interactions would be a strong contributor to the binding energy between G4 structures and organic molecules appears contradictory. Hence, there is a need to investigate why the inclusion of cationic groups enhances G4 binding.

**Figure 1:**
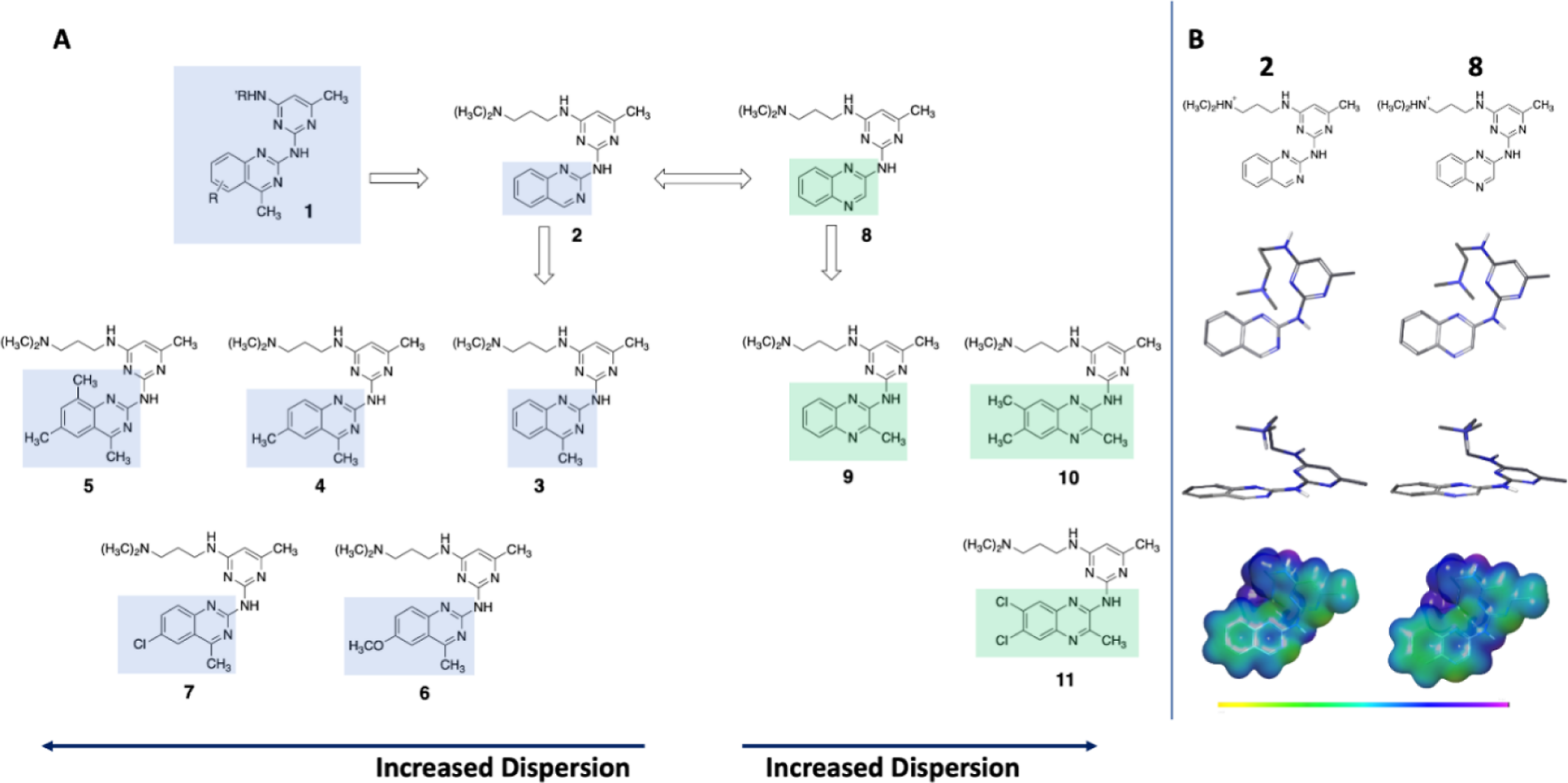
(A) Summary of the target compounds with a quinazoline (**2–7**) or quinoxaline (**8–11**) core and varying substitution patterns. (B) Conformational preference of compounds **2** and **8**, showing that the two scaffolds share the same conformational preference as well as electrostatic potential. The ESP map is shown with an ISO-value of 0.005 and an energy span of −40–140 kcal/mol. The color span represents different energy levels going from yellow/green (lowest negative, −40 kcal/mol) to purple (highest positive, 140 kcal/mol).

In this work, we designed a small series of molecules based on a quinazoline-pyrimidine scaffold of type **1** (Figure 1A)^35^ to investigate how different substitution patterns affect the arene–arene interactions and thus the G4 binding and stabilization. We hypothesized that compound **2**, bearing no substituents on the quinazoline core, should display less favorable binding with the G4 if the dispersion in the arene–arene interactions is essential for the binding. Sequential introduction of more substituents (**3–7**) should increase the dispersion component and correspondingly result in increased binding despite the electronic character of the substituents. To confirm this hypothesis in a different system, we next replaced the quinazoline (**2**) with a quinoxaline (**8–11**), which also would give information about how small changes in the arene impact binding (Figure 1A). Finally, the importance of the amine side chain was probed by comparison with the methyl side chain analogues. Collectively, this work gives a higher resolution to the interactions between G4 ligands and G4 DNA and demonstrates that this can be used to develop G4 ligands with good pharmacokinetic properties that target G4 DNA in cells.

## Results and Discussion

We first performed MM (molecular mechanics) calculations to ensure that all compounds shared an analogous conformational bias. This showed that all compounds exhibited comparable crescent-like conformations, indicating that conformational variances are unlikely to significantly affect the studied binding interactions (Figures 1B and S5–S14). All compounds can also exist in a linear conformation, this state is however both higher in energy and less populated, and only the crescent conformation is thus displayed for clarity.

The presence of the aliphatic amine that is cationic at physiological pH induces a strong electron-deficient electrostatic component in the molecule, which transfers to the arene that binds. This is clearly visible in the ESP maps for all the compounds (**2–11**) (Figures 1B and S5–S14).

### Organic Synthesis

**Scheme 1:**
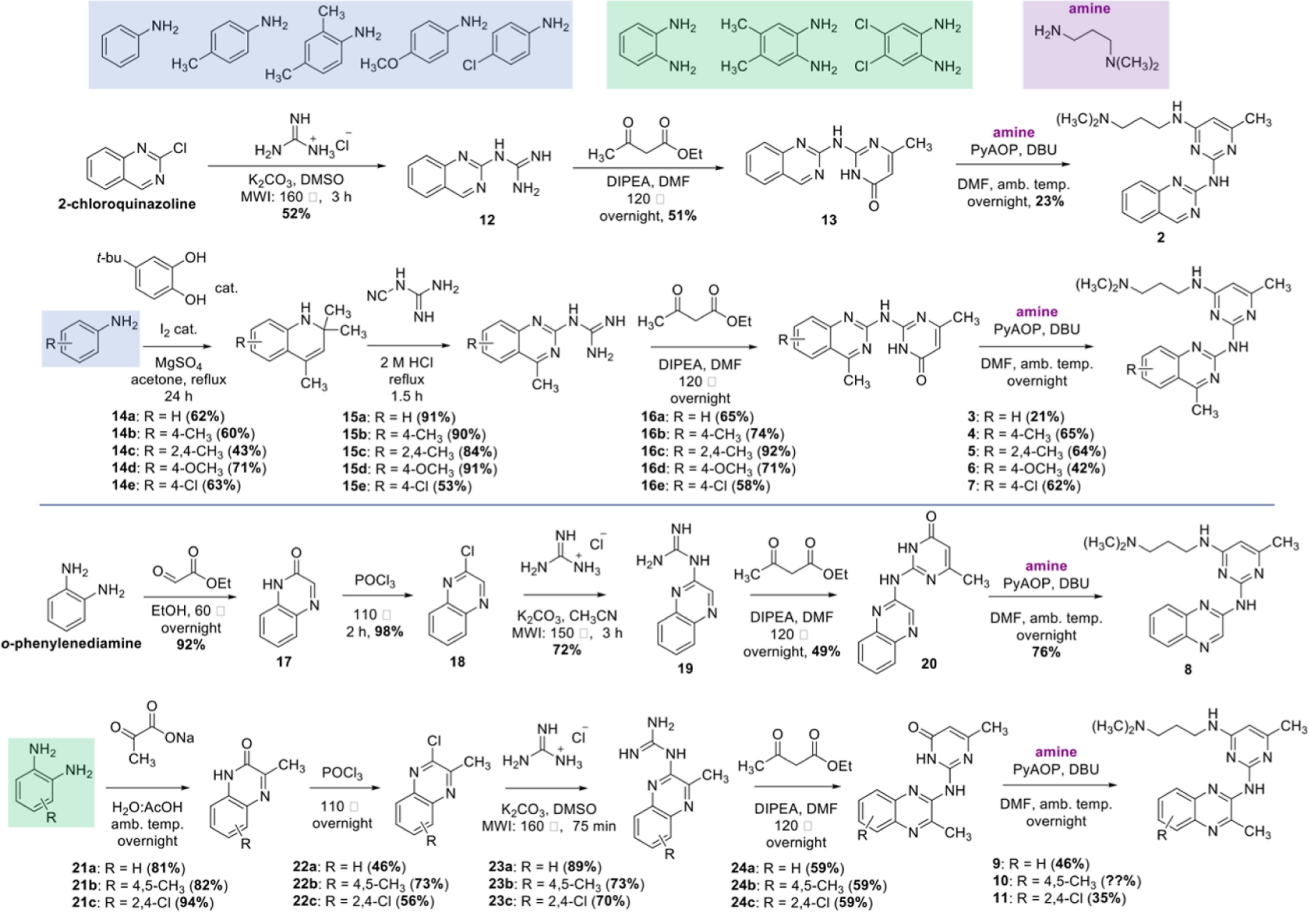
Total Synthetic Scheme for the Target Compounds (**2–11**)

The synthesis of the unsubstituted quinazoline (**2**) was performed starting from the commercially available 2-chloroquinazoline which was reacted with guanidine hydrochloride through a microwave-mediated S_N_Ar reaction in DMSO to afford **12** in 52% yield. Condensation between **12** and ethyl acetoacetate in DMF afforded **13** and a subsequent PyAOP-mediated S_N_Ar reaction^36^ between **13** and 3-(dimethylamino)propylamine gave **2** in 23% yield. Substituted quinazolines (**3–7**) were synthesized starting from the appropriate anilines highlighted in blue (Scheme 1). Thus, each aniline was first reacted with mesityl oxide (formed in situ) to afford the different dihydroquinolines (**14a–e**) in 43–71% yields. An acid-mediated ring-opening ring-closing reaction with cyanoguanidine afforded each quinazoline guanidine (**15a–e**) in 53–91% yields. Using the same procedure of condensation followed by a PyAOP-mediated S_N_Ar reaction afforded the final quinazolines (**3–7**) in yields of 21–65%. The quinoxalines (**8–11**) were synthesized using a similar approach. Hence, the unsubstituted quinoxaline (**8**) was synthesized starting from condensation between *o*-phenylenediamine and ethyl glyoxylate followed by a deoxychlorination of **17** in the presence of phosphoryl chloride (POCl_3_) to afford **18** in 90% overall yield. A microwave-mediated S_N_Ar reaction between **18** and guanidine hydrochloride in CH_3_CN afforded **19** in 72% yield. Condensation between **19** and ethyl acetoacetate followed by PyAOP-mediated S_N_Ar between **20** and 3-(dimethylamino)propylamine afforded **8** in 76% yields. The substituted quinoxalines (**9–11**) were synthesized from the corresponding diamines, highlighted in green, in a similar way to the synthesis of **8**. Consequently, sodium pyruvate was used in the condensation step to afford the substituted quinoxalinones (**21a–c**) in yields of 81–94%. Subsequent deoxychlorination with POCl_3_ afforded the chlorinated quinoxalines (**22a–c**) in 46–73% yields. S_N_Ar with guanidine hydrochloride in DMSO afforded quinoxaline guanidines (**23a–c**) in yields between 79 and 89% and subsequent condensation followed by PyAOP-mediated S_N_Ar afforded the final quinoxaline compounds (**9–11**) in yields of 35–46%. The compound synthesis is shown in Scheme 1.

### FRET Melting Assay

**Figure 2:**
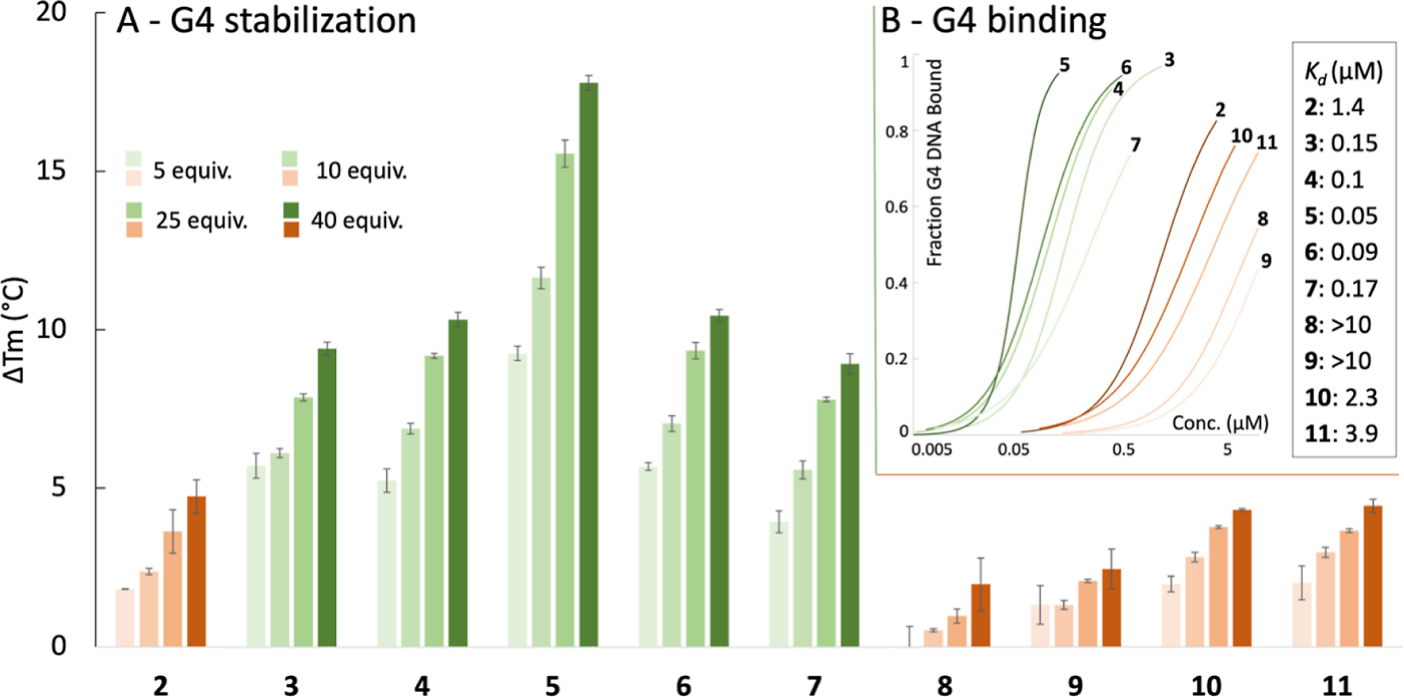
(A) FRET melting assay with compounds (**2–11**) at 5, 10, 25, and 40 equiv of added compound for Pu22 G4 DNA (0.2 μM), showing the ability of the compounds to affect the thermal stability of the G4 structure measured by a change in melting temperature (Δ*T*_m_). Error bars correspond to the SD of six independent experiments. (B) MST binding curves and *K*_d_ value for each compound (**2–11**) with Pu22 G4 DNA (see more details in Figure S3).

The synthesized compounds (**2–11**) were first analyzed using a Förster resonance energy transfer (FRET) assay (Figure 2A). This assay measures the compounds’ ability to affect the thermal stability of fluorescently labeled DNA, in this case, the *c-MYC* Pu22 G4 DNA (Table S1). Quinazoline compound **2**, bearing no substituents on the quinazoline core, showed a clear but modest ability to stabilize the G4 DNA, reaching an increased thermal stability of 7.5 °C at 40 equivalences of the added compound. The addition of one methyl group to the quinazoline core (**3**) provides almost twice as strong thermal stability compared to **2**. The addition of a second methyl group (**4**) further increased stabilization, albeit modestly (by 0.5 °C). This slight increase between **3** and **4** could stem from the suboptimal positioning of the added methyl substituent in relation to the guanines on the G4 surface, compared to other positions on the quinazoline ring. However, incorporating a third methyl group into the quinazoline core (**5**) resulted in a substantial increase, reaching an induced thermal stability of 20 °C at 40 equivalences. These results suggest that the ligand/G4 arene–arene interactions are predominantly dispersion-driven. Furthermore, the methyl-substituted (**4**) and the methoxy-substituted (**6**) display a similar G4 stabilization despite the strong electrostatic character of the methoxy substituent compared to the neutral methyl group, which further supports that these interactions are dispersion-driven. This conclusion is further supported by the fact that the replacement of the methoxy group in **6** with chlorine in **7** results in a very similar stabilization as **3**, **4**, and **6**.

Replacing the quinazoline core in **2** to the quinoxaline core (**8**) results in a compound with almost no ability to stabilize the G4 structure. This shows that the interactions are very sensitive to minor alterations in the arene system despite the overall structural similarity to compound **2**. It further emphasizes that the arene–arene interactions alone are essential for compounds to stabilize G4 DNA and that no other interactions seem to be able to compensate for this effect. However, increasing the substitution on the quinoxaline core (**9–11**) results in a clear increase in G4 DNA stabilization, which seems independent of the substituents’ electronic character. These findings closely mirror the trends observed with the quinazoline cores (**3–7**), underscoring the importance of the dispersion factor in arene–arene interactions with G4 DNA. We also conducted the same assay on another *c-MYC* G4 DNA sequence (Pu24T), which displayed identical trends for all the compounds (**2–11**) (Figure S1).

### Binding Affinity

To evaluate the apparent binding affinities (*K*_d_) for the compound series, we next used microscale thermophoresis (MST) with fluorescently labeled *c-MYC* Pu22 G4 DNA (Table S1). Overall, the measured MST binding results for compounds **2–11** corresponded well with the results from the FRET assay (Figures 2B and S3). The hydrogen-substituted quinazoline compound (**2**) measured an apparent *K*_d_ of 1.4 μM, whereas the methyl-substituted quinazoline **3** gave an apparent *K*_d_ of 0.15 μM. The addition of one methyl substituent in **3** thus resulted in an almost ten times higher MST binding affinity as compared to **2**. Compounds **4** and **5**, with two or three methyl groups on the quinazoline core, respectively, displayed apparent *K*_d_-values of 0.1 μM (**4**) and 0.05 μM (**5**), both being very potent G4 binders. Exchange of one of the methyl substituents in **4** to a methoxy group (**6**) once again provides similar results with an MST binding affinity of 0.09 μM, and the chlorine quinazoline (**7**) shows a similar but slightly lower binding (0.17 μM).

Comparison between the unsubstituted quinazoline core (**2**) and the unsubstituted quinoxaline core (**8**) is in line with the FRET assay and shows a big drop in binding for **8** with an apparent *K*_d_ > 10 μM. Addition of one methyl group on the quinoxaline core (**9**) results in a similarly weak binding. However, adding two additional methyl groups (**10**) or two chlorines (**11**) both results in a better binder compared to **8** and **9** (apparent *K*_d_ values: 2.26 μM and 3.85 μM for **10** and **11**, respectively, compared to >10 μM for both **8** and **9**).

Overall, the MST results are in good agreement with the results from the FRET assay and revealed both highly efficient G4 ligands and suggest that dispersion is essential for the molecules to exhibit strong binding and stabilization of G4 DNA.

### Second Compound Series Without Basic Amine Side Chain

**Figure 3:**
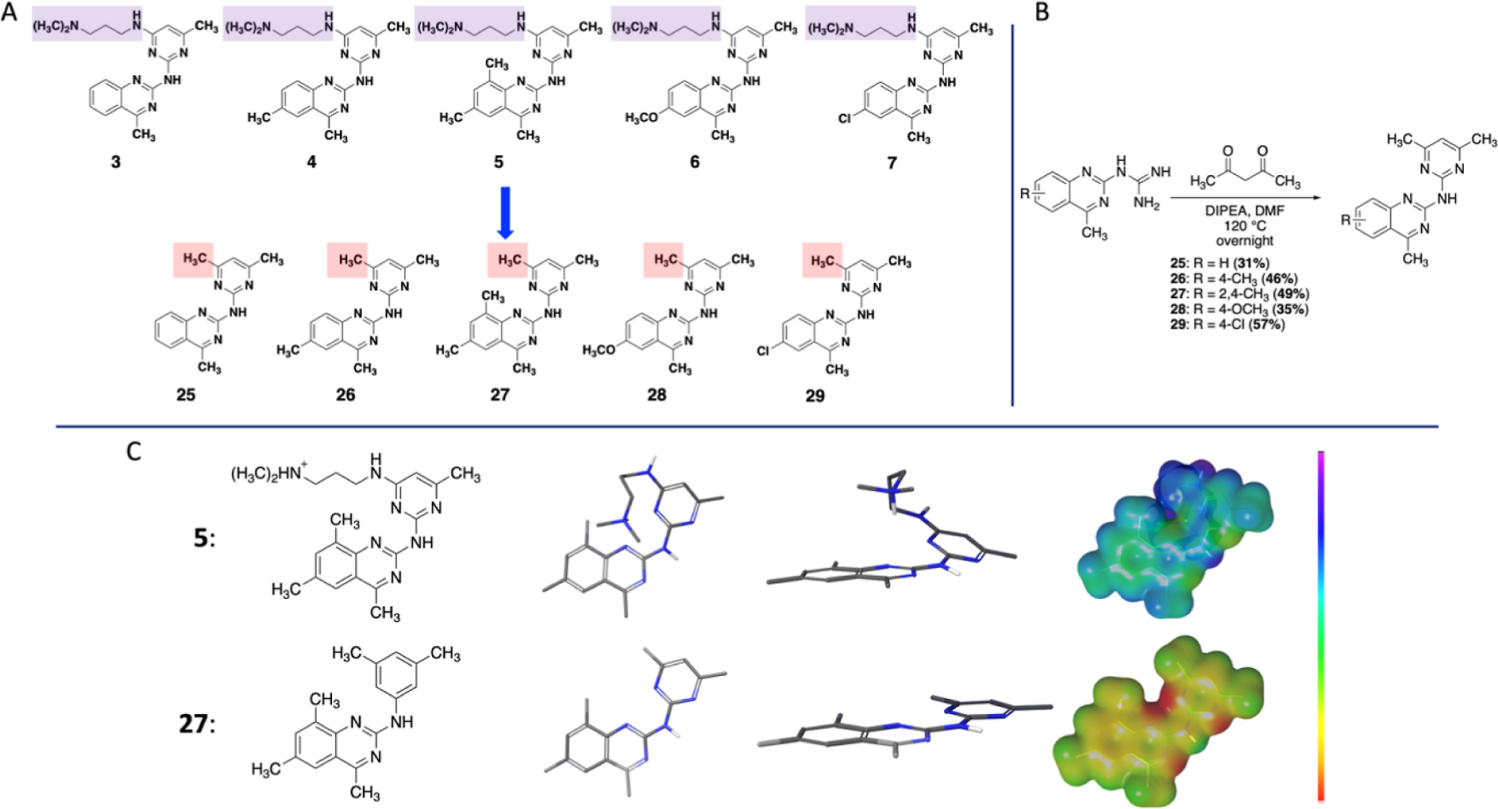
(A) Replacement of the amine side chain in the best quinazoline compounds (**3–7**) with a methyl group to get the corresponding methylated derivatives (**12–16**). (B) Synthesis of the methylated quinazolines (**25–29**). (C) Comparison of the amine side chain quinazolines and their methylated analogues, illustrated by **5** and **27**, highlighting their electrostatic differences. The ESP map is shown with an ISO-value of 0.005 and an energy span of −40–140 kcal/mol. The color span represents different energy levels going from yellow/green (lowest negative, −40 kcal/mol) to purple (highest positive, 140 kcal/mol).

All the designed compounds (**2–11**) have a strong electron-deficient character throughout the entire molecule because of the charged amine, clearly shown by the ESP maps (Figures 1 and S5–S14). To investigate the importance of the electrostatic nature of the compounds, we next wanted to convert key compounds into more electron-rich species and measure how this affects G4 binding and stabilization. Thus, we selected the five best quinazoline binders (**3–7**) and envisioned replacing the amine side chain with a methyl group to give compounds **25–29**, illustrated in Figure 3A. Synthesis of the new derivatives (**12–16**) could easily be achieved by condensing the quinazoline guanidines (**15a–e**) with acetylacetone (Figure 3B), similar to the condensations performed with ethyl acetoacetate for the first compound series (Scheme 1). As exemplified by the ESP map in Figure 3C that compares compounds **5** and **27**, the replacement of the amine side chain with a methyl group results in significantly more electron-rich species. The same electrostatic trend was observed for the whole set (**25–29**, Figures S15–S19) and the conformational preference of these compounds overlapped well with the first set of compounds (**2–11** vs **25–29**) (Figures 3C and S15–S19).

### Effect of Side Chain and Electrostatics on G4 Binding and Stabilization

**Figure 4:**
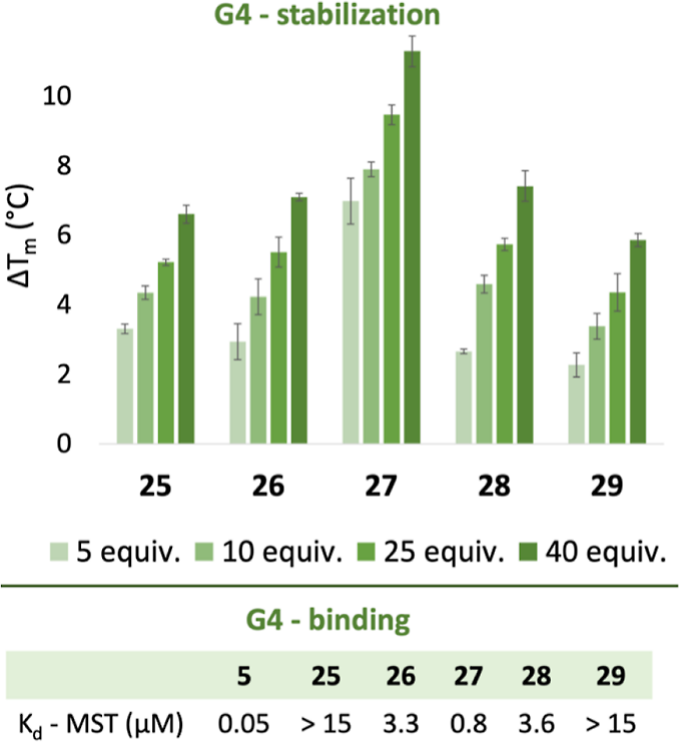
(Top) Evaluation of the ability of compounds **12–16** to affect the thermal stability of the *c-MYC* Pu22 G4 DNA using the FRET melting assay. 5, 10, 25, and 40 equiv of compounds (**12–16**) were added to 0.2 0.05 of the *c-MYC* Pu22 G4 DNA structure. Error bars correspond to the SD of six independent experiments. (Bottom) Apparent binding affinity (*K*_d_) for each compound using MST.

The new compounds (**25–29**) were first evaluated in the FRET melting assay with *c-MYC* Pu22 G4 DNA (Figure 4, top). In line with the first set of compounds, this shows that the more substituted compound (**27**) has the highest ability to stabilize G4 DNA (12 °C increase at 8 μM). Furthermore, variation of the electrostatic influence of the substituents (hydrogen vs methyl vs methoxy vs chlorine) did not significantly affect the G4 stabilization ability of the compounds. The same trend was observed for the *c-MYC* Pu24T G4 DNA (Figure S2).

The binding affinity of the new set of compounds was next evaluated using MST (Figures 4 and S4). Compound **25**, with only one methyl group on the quinazoline core, proved to be a poor binder with an apparent *K*_d_ higher than 15 μM. The addition of a methyl- or a methoxy-group (**26** and **28**, respectively) improved the MST binding affinity to 3.3 and 3.6 μM, respectively. As observed with the first set of compounds, the chlorine analogue (**29**) had a weaker binding affinity (apparent *K*_d_ > 15 μM) compared to **26** and **28**. The most substituted compound (**27**), having three methyl groups on the quinazoline core, displayed a superior *K*_d_ value of 0.8 μM in this assay and was thus the best binder in the second compound series (Figures 4 and S4).

To dissect the driving interactions between G4 DNA and the ligands further, we performed isothermal titration calorimetry (ITC) measurements of the most active quinazoline **5** (with amine side chain) and its matched pair analogue **27** (without amine side chain). Unfortunately, this was only possible using reverse titration, adding G4 DNA to the ligand, and a direct comparison to the MST binding affinities, which were performed by titrating the ligand to the G4, can thus not be performed. However, ITC displayed the same trends with *K*_d_-values of 0.88 μM (**5**) and 1.04 μM (**27**) and further showed that compound **27** had a Δ*H*-value that was −1.1 kcal/mol lower than compound **5** (Figure S20). This shows that the dispersion arene–arene interaction likely is the main direct interaction driving the binding event and that the amine does not contribute to a favorable ionic interaction with the phosphate backbone. The reason why **5** is more efficient in stabilizing G4 DNA could instead be linked to the entropic penalty (−*T*Δ*S*), which is less for **5** (1.85 kcal/mol) compared to **27** (3.2 kcal/mol).

Overall, comparing the first (**2–11**) and second (**25–29**) sets of compounds underscores that a strong dispersion component in the arene–arene interaction seems to result in potent G4 binders. Additionally, there is no ambiguity that the aliphatic amine side chains contribute considerably to G4 binding and stabilization. However, previous studies combined with our ITC measurements for **5** and **27** show that this is not linked to electrostatic interactions between the cationic amines and the anionic DNA.^32−34^ Furthermore, recent work with other compound classes and known G4 ligands has indicated that electrostatically electron-deficient arenes are central for the binding interactions.^37^ We here expand on this and propose that the introduction of the aliphatic amine improves the binding of the compounds to G4 DNA indirectly by changing the electrostatic nature of the arenes. For example, there is already a strong dispersion component in **27**, and this component could be further enhanced by making the arene electron-deficient, as in **5**. Consequently, an electrostatically electron-deficient arene in synergism with a prominent dispersion component appears to be vital for compounds to engage in potent arene–arene interactions with the G4 surface. However, the reason for the improved binding of electrostatically electron-deficient compounds can be more complex, e.g., including factors that affect the entropic (Δ*S*) term in the binding event such as conformational freedom and bulk solvent release.

### Polymerase Extension Assay

**Figure 5:**
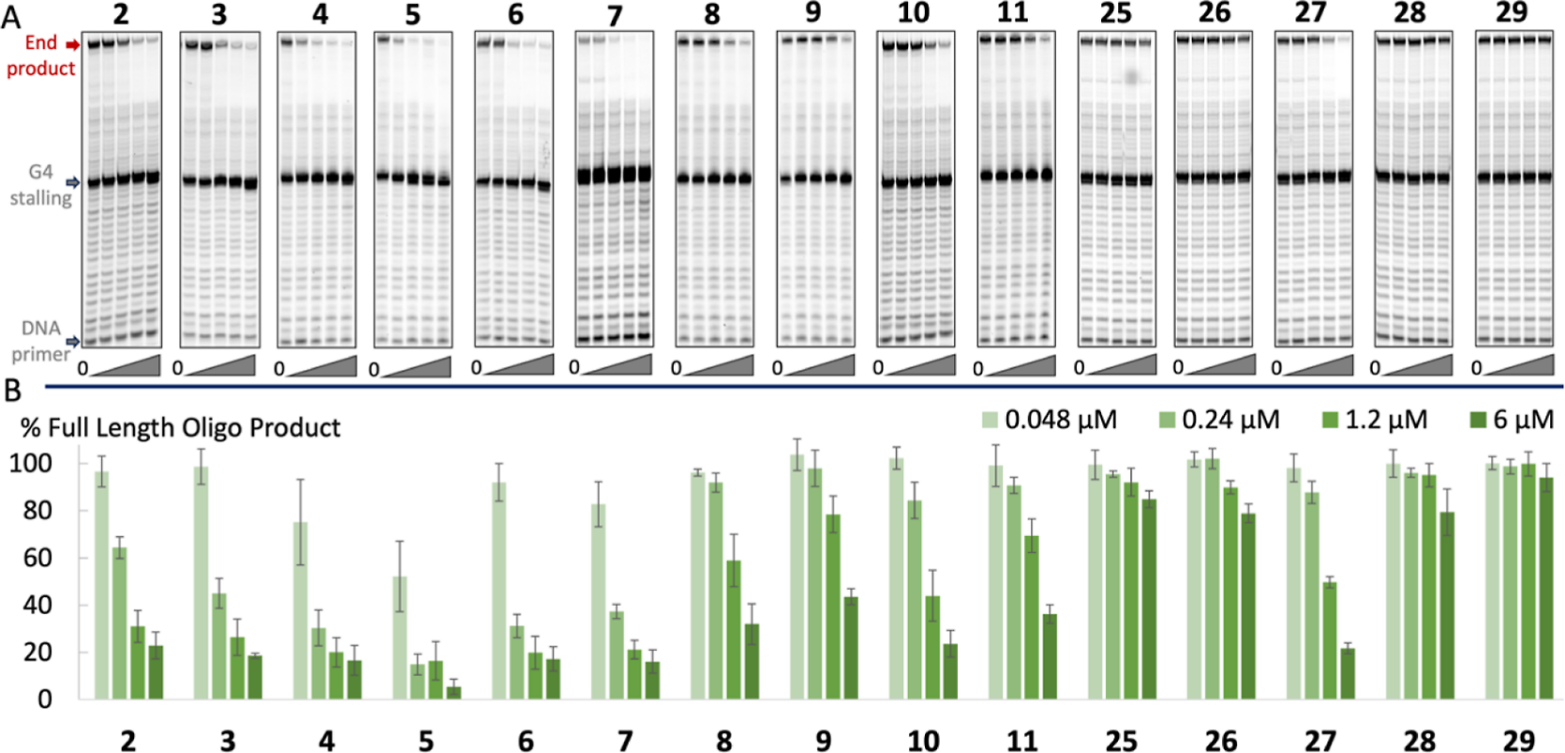
(A) Polymerase extension assay on a template with a G4 structure in the presence of each compound (**2–11**, **25–29**) using increasing concentrations of compound 0, 0.048, 0.24, 1.2, and 6.0 μM. The starting DNA primer, DNA runoff (end) product, and the site of the G4 structure on the template are indicated. (B) Quantification of the Taq-Polymerase stop assay with all the synthesized compounds (**2–11** and **25–29**) at 0.048, 0.24, 1.2, and 6 μM compared to DMSO control. The DNA concentration in the assay is 0.048 μM. Error bars correspond to the SD of at least two independent experiments.

To challenge all the synthesized compounds (**2–11** and **25–29**) in a more intricate context, we conducted a Taq polymerase stop assay to evaluate their capacity to impede DNA synthesis through G4 stabilization. In this assay, we compared the progress of the Taq DNA polymerase along two distinct DNA templates: one with a G4 structure (Figure 5) and another serving as a non-G4 control (Figure S21). If an external species (e.g., G4 ligand) binds and stabilizes the G4 structure, the polymerase will be partially halted. The extent of this DNA polymerase stalling is dependent on the ability of the compound to stabilize the G4 on the DNA template. The increased G4 stabilization can be detected as a reduction in the amount of newly synthesized DNA that has reached the end of the DNA template (full-length/end-product). Analysis of these shorter terminated DNA products will give information about the precise location of the DNA polymerase stalling with a single nucleotide resolution. If the ligand imposes little to no stabilization or binding, the G4 is readily resolved by the DNA polymerase, and no difference in the amount of DNA end-product can therefore be expected upon the addition of the compound.

All quinazoline amines (**2–7**) blocked the Taq DNA polymerase at the first G-tetrad on the template strand (Figure 5). Quinazoline **5** showed a very potent reduction of the DNA end-product with a 50% inhibition at a 1/1 ratio of compound to DNA template (0.048 μM), which corresponded very well with the *K*_d_ value obtained (Figures 2B and S3). The trends for compounds **2**, **3**, **4**, **6**, and **7** also correlated very well with the results from the FRET (Figure 2A) and MST (Figures 2B and S3) assays. Comparison between the compounds at 0.24 μM showed that the addition of one methyl group (**2** to **3**) reduces the full-length DNA product from about 65 to 45%. Adding a second methyl group (**4**) reduced the amount of full-length product further (to about 30%), which is similar to that of the methoxy- and chloro-substituted quinazolines **6** and **7**. At this concentration, the trimethylated **5** almost completely blocked the Taq DNA polymerase at the G4 structure. This data, therefore, also supports the hypothesis that the dispersion component in the arene–arene interactions is essential for the strong binding and stabilization of G4 DNA in a more complex setting. All compounds displayed good selectivity for G4 DNA over double-stranded DNA (Figure S21). At high concentrations (>4 μM), an effect on the Taq polymerase was observed also in the absence of a G4 structure for compound **5**. However, 50% inhibition for **5** is reached already at 0.048 μM when the G4 sequence is used (Figure 5), leaving a wide concentration window between the effect on G4 DNA and double-stranded DNA.

The quinoxalines (**8–11**) were significantly less effective in blocking the DNA polymerase (Figure 5), which can be directly linked to their lower ability to bind and stabilize G4 DNA structures. The most substituted methyl quinoxaline **10** was slightly more effective than the other analogues, but the variation was less pronounced in this series. Still, this underscored how critical the design of the arene partner is to generate a strong G4-ligand.

Replacing the aliphatic amine side chain with a methyl in quinazolines (**25–29**) resulted in a sharp reduction in G4 stabilization (Figure 5). The most substituted quinazolines **27** was also the most active in this series, reaching 50% inhibition at 1.2 μM. A pronounced electron-deficient electrostatic component thus seems necessary for the compounds to stabilize G4 structures in the polymerase extension assay. This was reinforced by the fact that hydrogen-substituted quinazoline **2** with an amine side chain resulted in a better or comparable stabilizer with the poly methyl substituted **27** without an amine side chain, despite **27** showing better results in both the FRET and MST assays. Still, the combination of strong dispersion and electron-deficient electrostatics results in the best compounds to stabilize G4 structures also in the Taq polymerase stop assay.

### Nuclear Magnetic Resonance Titrations

**Figure 6:**
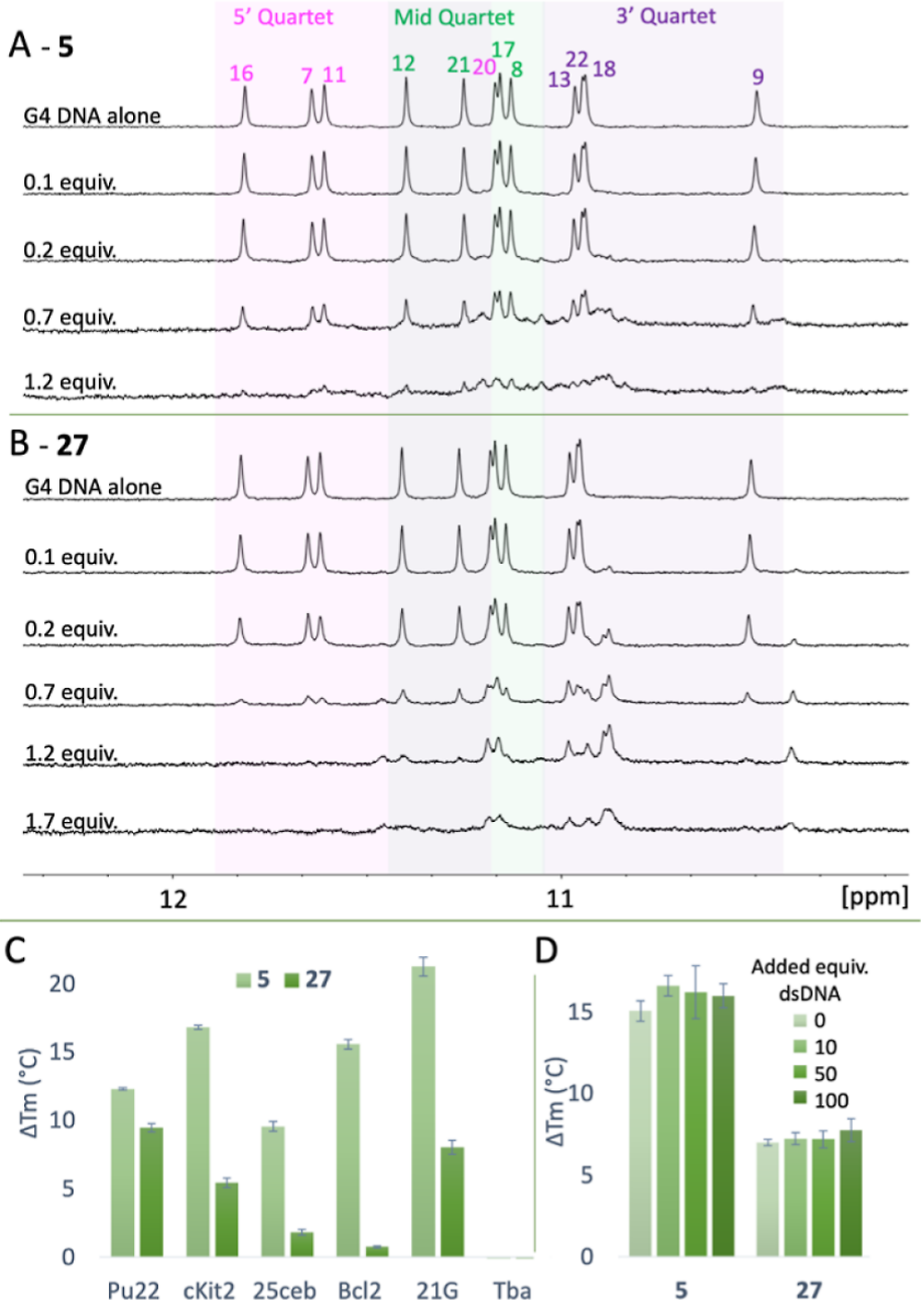
^1^H NMR (850 MHz) titrations for *c-MYC* Pu22 G4 DNA with (A) **5** and (B) **27**. The initial DNA concentration was 90 μM, and the compound was then added in different equivalences so that the final ratio of G4/compound corresponded to 1/1.2 (**5**) and 1/1.7 (**27**). (C) FRET melting assay with **5** and **27** at 2 and 5 μM concentrations, respectively, for G4 DNA structures (0.2 μM), showing the ability of the compounds to stabilize different G4 DNA structures. cKIT2 (*c-KIT* promoter) and 25 ceb (human minisatellite) are parallel G4 forming sequences. Bcl2 (*BCL-2* promoter) and 21G (human telomere) are hybrid G4-forming sequences. Bom17 (*Bombyx* telomere) and Tba (thrombin binding aptamer) are antiparallel G4 forming sequences. Error bars correspond to the SD of at least six independent experiments. (D) Ability of **5** and **27** at 2 and 5 μM concentrations, respectively, to affect the thermal stability of *c-MYC* Pu22 G4 DNA (0.2 μM) in the presence of different amounts (0–100 equiv) of a double-stranded competitor dsDNA (ds26). Error bars correspond to the SD of at least six independent experiments.

To study the interactions of compounds **5** and **27** with G4 DNA in more detail, we performed a nuclear magnetic resonance (NMR) titration assay for both compounds with *c-MYC* Pu22 G4 DNA. In this assay, we monitored chemical shift alterations and the broadening of the well-defined imino protons from the G4 DNA located at 10–12 ppm in the NMR spectrum upon the addition of different compound concentrations. In agreement with the FRET and MST results, the NMR data show that both **5** and **27** bind with the *c-MYC* Pu22 G4 DNA (Figure 6). The titrations with compound **5** (Figure 6A) had a notable effect on the imino peaks already at 0.7 equivalences, and additional peaks from the G4:**5** complex appear in the imino region when compound **5** binds to the G4 structure. Compound **27** (Figure 6B) also results in a clear effect on the imino protons at 0.7 equivalences, although the peaks are broadened and do not form as clearly defined new peaks as for **5**. This effect is likely due to the higher *K*_d_ value of **27** compared to **5**, giving faster on–off rates. Although it is challenging to conclude the details of the binding interactions, the observed impact on the protons in the 3′ quartet for both compounds, especially compound **27**, strongly implies the presence of an end-stacking type of interaction.

### Selectivity of Compounds **5** and **27**

Additional G4 DNA structures were examined in the FRET melting assay to study the selectivity of compound (**5**) and its methylated analogue (**27**) toward different G4 structures. Compounds **5** and **27** were tested at 2 and 5 μM, respectively, for their abilities to increase the thermal stability of G4 DNA structures (Figure 6C and Table S1). Overall, this confirms that compound **5** is a much stronger G4 stabilizer, as compared to **27**. Compound **5** efficiently stabilizes the telomeric and oncogenic G4s, whereas compound **27** shows a very different and weaker stabilization pattern, which shows that the amine side chain influences both G4 stabilization and selectivity. Neither compound affected the thermal stability of Tba (thrombin binding aptamer), and no clear preference for a specific G4 topology can be observed.

We next challenged the compounds’ G4 selectivity with a FRET melting competition assay using **5** (2 μM) and **27** (5 μM) with *c-MYC* Pu22 G4 DNA and increasing amounts of dsDNA (Figure 6D and Table S1). No significant change was observed in the compound-induced thermal stability of *c-MYC* Pu22 G4 DNA, which shows that the compounds have a strong selectivity for G4 DNA over dsDNA (Figure 6D). In addition, we investigated if we could detect any binding of the compounds to dsDNA using MST with a fluorescently labeled dsDNA (Table S1). In line with the other assays, no binding could be observed, and combined with the low effect on the non-G4 template in the DNA polymerase stop assay, we conclude that the compounds are highly selective for G4 DNA over dsDNA.

### Physicochemical Profiling

All compounds (**2–11**, **25–29**) were screened for their log*D* and solubility at pH 7.4 and for their intrinsic clearance in rat hepatocytes and human microsomes (Table S2). Overall, the quinazolines (**2–7**) displayed good properties with high solubility (858 to over 1000 μM) and low clearance. The quinoxalines (**8–11**) also displayed good solubility and low clearance in human microsomes but slightly higher clearance in rat hepatocytes. Replacing the basic amine side chain to a methyl substituent in the quinazolines (**25–29**) still retained good solubility (134 to over 1000 μM) but increased clearance in both rat hepatocytes and human microsomes. The log*D* was low for compounds with a basic amine side chain (**2–11**) and increased slightly when the amine was replaced with a methyl (**25–29**). As expected, additional substituents also affected the logD with the chloro-substituents increasing log*D* the most.

We next performed further profiling of the most promising compound **5** and its matched pair neutral analogue **27**, by measuring intrinsic permeability in a Caco-2 assay (Table S3). Neutral compound **27** showed very good permeability in this assay (*P*_app_ 70 × 10^–6^ cm/s) while **5** containing the basic amine displayed low permeability (*P*_app_ < 0.13 × 10^–6^ cm/s) which could indicate that this compound will struggle to permeate cell membranes. Cytotoxicity for the two compounds was measured in a THP-1 cell line; compound **27** showed no cytotoxicity below 50 μM. Compound **5** on the other hand showed noticeable cytotoxicity in this assay with an IC_50_ of 2.9 μM. This was further confirmed using Immortalized Human Total Liver Cell Population (IHTLCP) and HeLa cells (Figures S22 and S23). So, even though compound **5** displayed a low permeability in the Caco-2 assay, sufficient amounts of compound can still enter the cell to exert cytotoxic effects. This observed cytotoxicity of **5** can be linked to its potent G4 binding and stabilization, as it can potentially act as a pan-G4 stabilizer and affect various G4-regulated cellular processes, such as telomere maintenance, gene transcription, and DNA replication.

### G4 Stabilization in Cells

**Figure 7:**
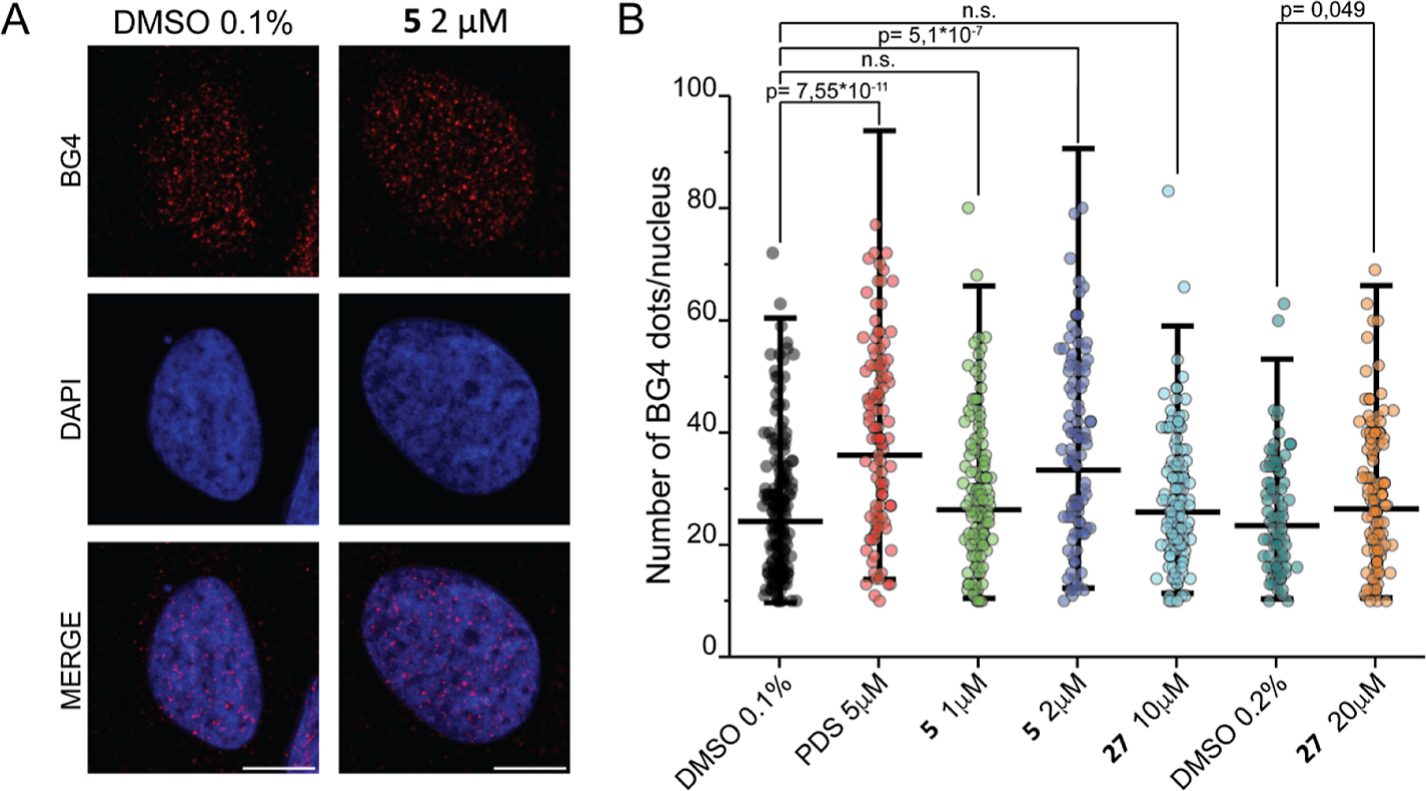
(A) Representative images of HeLa cells stained with the BG4 antibody after treatment for 24 h. Scale bar = 10 μM. (B) Quantification of BG4-positive cell nuclei. Data represent populations of individual cells for each condition of the final experiment: DMSO 0.1% = 166 cells, **PDS** 5 μM = 109 cells; **5** 1 μM = 130 cells; **5** 2 μM = 100 cells; **27** 10 μM = 132 cells; **27** 20 μM = 118 cells and DMSO 0.2% = 90 cells. Means ± 2SD are indicated. Analysis of the data was performed using Welch-corrected two-sample *t* tests of ln-transformed data, and *p* values are indicated (n.s. = not statistically significant).

To determine if **5** and **27** stabilize G4 DNA structures in cultured human cells, we used the anti-G4 DNA antibody BG4 and performed immunofluorescence microscopy to visualize and quantify G4 DNA structures in HeLa cells. At the compound concentrations of **5** that were close to the cell viability IC_50_ (Figure S22), we found that the number of BG4 foci per cell nucleus increased significantly in the cells treated with 2 μM of **5** compared to the cells treated with 1 μM of **5** and compared to mock-treated cells (Figures 7 and S24). Compound **27** seems to increase the number of BG4 foci at the highest concentration (20 μM) suggesting that this compound also affects G4s in cells although this effect is not statistically fully significant. The effect of **5** at 2 μM is comparable to the effect seen for one of the most well-known and potent G4-ligands in the literature, PDS, when it is used at 2.5 times higher concentration (5 μM). Together, these data support the hypothesis that the strong G4 binding and stabilization of **5** combined with adequate solubility and apparent permeability result in a strong stabilization of G4s in cells which can explain the strong cell viability effects seen for this compound.

## Conclusions

We have designed a small library of G4 ligands to evaluate the key components driving G4/ligand interactions and investigate how such ligands can be optimized toward further drug development efforts. To do this, we used orthogonal assays to measure the synthesized compounds’ abilities to bind and stabilize G4 DNA. This revealed highly selective and potent G4 ligands with *K*_d_-values in the nanomolar range. The driving interaction for the G4:ligand complex is arene–arene interactions, and we show that the dispersion component in combination with electron-deficient electrostatics is central to optimize these interactions. The electrostatic component can be introduced using an aliphatic amine that is charged at physiological pH. We propose that such amine side chains do not increase the binding affinity by direct interactions with the DNA backbone, but instead indirectly through the induction of an electron-deficient arene which in turn enhances the interactions with the G4. Furthermore, we show that the chemical composition of the central arene fragment of the G4 ligand is crucial for its ability to bind and stabilize G4 DNA, where the change of position of one atom can be detrimental to the G4 binding ability.

In support of the other assays, the pharmacokinetic profiling revealed compounds **5** and **27** to be potent G4 binders with good solubility but with varying cell permeability, underscoring the value in the rational design of flexible small organic molecules without permanent charges toward targeting G4 DNA. Even though compound **5** was still able to enter cells, the impact on cell permeability upon the inclusion of one aliphatic amine was considerable, underscoring the potential problematic nature of including several cations of sort in G4 ligands if a therapeutic end goal is desired. Finally, the combination of strong G4 binding and stabilization observed for compound **5** with suitable pharmacokinetic properties also translates into an increased number of G4s in cells, which confirms **5** as a highly potent G4-ligand suitable for cell studies. This successful example combined with the comprehensive insights into the interactions between G4 ligands and G4 structures aspire to catalyze the development and refinement of G4 ligands for the exploration of G4 DNA as potential therapeutic targets.

## Experimental Section

### Chemistry

All reagents and solvents were used as received from commercial suppliers unless stated otherwise. TLC was performed on aluminum backed silica gel plates (median pore size 60 Å, fluorescent indicator 254 nm) and detected with UV light. Flash column chromatography was performed using silica gel with an average particle diameter of 50 μm (range 40–65 μm, pore diameter 53 Å), eluents are given in brackets. ^1^H and ^13^C NMR spectra were recorded on a Bruker 400 or 600 MHz spectrometer at 298 K, calibrated by using the residual peak of the solvents as the internal standard (CDCl_3_: δ (ppm) H = 7.26; δ (ppm) C = 77.16. DMSO-*d*_6_: δ (ppm) H = 2.50; δ (ppm) C = 39.50). There is always one carbon which is not visible (or merged with another carbon) in the ^13^C NMR for compounds (**2–11**), even if more than 4000 scans with concentrated samples were measured at 150 MHz. All tested compounds showed a purity of ≥95% determined by the UV chromatogram by LC–MS analysis. LC–MS was performed on an Agilent 6150 Series Quadrupole LC/MS system. Microwave reactions were carried out in an Initiator + microwave instrument from Biotage, using sealed 0.2–0.5, 2–5, or 10–20 mL process vials. Reaction times refer to irradiation time at the target temperature, not the total irradiation time. The temperature was measured with an IR sensor.

### 1-(Quinazolin-2-yl)guanidine (**12**)

A microwave vial (2–5 mL) was charged with 2-chloroquinazoline (200 mg, 1.22 mmol), guanidine hydrochloride (232 mg, 2.43 mmol), and K_2_CO_3_ (504 mg, 3.65 mmol). Then, DMSO (4 mL) was added, and the mixture was reacted using MWI (160 °C, 3 h). The mixture was then cooled to ambient temperature. Water was added, and the mixture was sonicated and then cooled over ice. The solids were then collected with suction filtration and washed with small portions off water and Et_2_O to afford **12** (118 mg, 52%) as a yellow solid. ^1^H NMR (400 MHz, DMSO-*d*_6_): δ (ppm) 9.41 (s, 1H), 7.99 (dd, *J* = 8.1, 1.3 Hz, 1H), 7.88 (ddd, *J* = 8.5, 6.8, 1.3 Hz, 1H), 7.80 (dd, *J* = 8.5, 1.3 Hz, 1H), 7.50 (ddd, *J* = 8.1, 6.8, 1.3 Hz, 1H). ^13^C NMR (151 MHz, DMSO-*d*_6_): δ (ppm) 162.7, 158.7, 157.4, 149.3, 134.7, 127.8, 125.6, 124.9, 120.8.

### 6-Methyl-2-(quinazolin-2-ylamino)pyrimidin-4(3*H*)-one (**13**)

A vial was charged with **12** (117 mg, 0.625 mmol). Then, DMF (3 mL), DIPEA (218 μL, 1.25 mmol), and ethyl acetoacetate (797 μL, 6.25 mmol) were added sequentially, and the solution was stirred at 120 °C for 24 h. The mixture was then diluted with Et_2_O, and the precipitate was collected with suction filtration and the solids were washed with more Et_2_O. Purification by flash column chromatography (eluent: 3 → 3.5% MeOH in CH_2_Cl_2_) afforded **13** (64 mg, 40%) as a pale-brown solid. ^1^H NMR (400 MHz, DMSO-*d*_6_): δ (ppm) 13.24 (s, 1H, NH), 11.51 (s, 1H, NH), 9.53 (s, 1H), 8.09 (d, *J* = 8.1 Hz, 1H), 7.98 (t, *J* = 7.7 Hz, 1H), 7.79 (d, *J* = 8.6 Hz, 1H), 7.60 (t, *J* = 7.5 Hz, 1H), 5.84 (s, 1H), 2.17 (s, 3H, CH_3_). ^13^C NMR (100 MHz, DMSO-*d*_6_): δ (ppm) 163.6, 161.1, 155.6, 151.4, 149.2, 148.5, 135.8, 128.4, 125.9, 125.3, 121.2, 104.7, 23.5.

### N4-(3-(Dimethylamino)propyl)-6-methyl-N2-(quinazolin-2-yl)pyrimidine-2,4-diamine (**2**)

A vial was charged with **13** (60 mg, 0.24 mmol) and PyAOP (161 mg, 0.309 mmol). Then, DMF (1.5 mL) and DBU (53 μL, 0.35 mmol) were added, and the resulting solution was stirred at ambient temperature for 1 h. Propylamine (75 μL, 0.60 mmol) was then added, and the solution was stirred overnight. The reaction solution was then concentrated under reduced pressure and diluted with water. The AQ layer was then extracted with CHCl_3_/IPA (3/1, 15 mL × 3). The combined org. layer was then dried over Na_2_SO_4_, filtered, and concentrated under reduced pressure. Purification by flash column chromatography (eluent: 15 → 20% MeOH (1% NH_4_OH) in CH_2_Cl_2_) afforded **2** (18 mg, 23%) as a dark-yellow solid. ^1^H NMR (400 MHz, DMSO-*d*_6_): δ (ppm) 9.37 (s, 1H), 7.98 (d, *J* = 8.1 Hz, 1H), 7.86 (ddd, *J* = 8.5, 6.9, 1.5 Hz, 1H), 7.71 (d, *J* = 8.5 Hz, 1H), 7.48 (t, *J* = 7.5 Hz, 1H), 7.37 (s, 1H) 6.01 (s, 1H), 3.29 (s, 2H), 2.72–2.60 (m, 2H), 2.37 (s, 6H), 2.18 (s, 3H), 1.84 (app. dt, *J* = 12.2, 7.5 Hz, 2H). ^13^C NMR (151 MHz, DMSO-*d*_6_): δ (ppm) 163.4, 162.0, 157.9, 156.2, 150.7, 134.4, 127.8, 126.0, 124.5, 121.1, 98.3, 55.6, 47.6, 43.7, 25.7, 23.2. HRMS *m*/*z*: [M – H]^+^ calcd for C_18_H_24_N_7_^+^, 338.2099; found, 338.2108.

### 2,2,4-Trimethyl-1,2-dihydroquinoline (**14a**)

A round-bottom flask (RBF) was charged with MgSO_4_ (6.46 g, 53.7 mmol), catechol (53.6 mg, 0.322 mmol), and I_2_ (136 mg, 0.537 mmol). Then, aniline (979 μL, 10.7 mmol) and acetone (20 mL) were added, and the mixture was stirred at reflux overnight. After cooling down to ambient temperature, the mixture was filtered over a plug of Celite with EtOAc, and the resulting org. solution was concentrated under reduced pressure. Purification by flash column chromatography (eluent: 5% EtOAc in *n*-heptane) afforded **14a** (1.15 g, 62%) as orange oil. ^1^H NMR (400 MHz, CDCl_3_): δ (ppm) 7.06 (dd, *J* = 7.6, 1.5 Hz, 1H), 6.98 (td, *J* = 7.6, 1.5 Hz, 1H), 6.64 (td, *J* = 7.5, 1.2 Hz, 1H), 6.46 (dd, *J* = 7.9, 1.2 Hz, 1H), 5.31 (d, *J* = 1.5 Hz, 1H), 3.87 (s, 1H), 1.99 (d, *J* = 1.4 Hz, 3H), 1.28 (s, 6H). ^13^C NMR (100 MHz, CDCl_3_): δ (ppm) 143.2, 128.6, 128.4, 128.4, 123.6, 121.6, 117.2, 113.0, 51.8, 31.0, 18.6.

### 2,2,4,6-Tetramethyl-1,2-dihydroquinoline (**14b**)

A RBF (100 mL) was charged with MgSO_4_ (11.2 g, 93.3 mmol), catechol (93 mg, 0.56 mmol), and I_2_ (236 mg, 0.930 mmol). Then, 4-methylaniline (2.00 g, 18.7 mmol) and acetone (50 mL) were added, and the mixture was refluxed for 24 h. After cooling down, the mixture was filtered over a plug of Celite with EtOAc and the resulting org. solution was concentrated under reduced pressure. Purification by flash column chromatography (eluent: 2% EtOAc in *n*-heptane) afforded **14b** (2.11 g, 60%) as pink oil. ^1^H NMR (400 MHz, CDCl_3_): δ (ppm) 6.88 (d, *J* = 2.0 Hz, 1H), 6.84–6.77 (m, 1H), 6.38 (d, *J* = 7.9 Hz, 1H), 5.32 (d, *J* = 1.7 Hz, 1H), 3.56 (s, 1H, NH), 2.23 (s, 3H), 1.99 (d, *J* = 1.7 Hz, 3H), 1.26 (s, 6H, (CH_3_)_2_). ^13^C NMR (100 MHz, CDCl_3_): δ (ppm) 141.1, 128.9, 128.8, 128.7, 126.3, 124.3, 121.8, 113.1, 51.8, 30.9, 20.8, 18.8.

### 2,2,4,6,8-Pentamethyl-1,2-dihydroquinoline (**14c**)

The reaction was carried out in two batches. A μ-wave vial (10–20 mL) was charged with MgSO_4_ (5.00 g, 41.5 mmol), catechol (41 mg, 0.25 mmol), and I_2_ (105 mg, 0.412 mmol) each. Then, 2,4-dimethylaniline (1.02 mL, 8.25 mmol) and acetone (20 mL) were added, and the mixture was heated at 110 °C in the sealed tube for 24 h. After cooling down, the mixture was filtered over a plug of Celite with EtOAc and the resulting org. solution was concentrated under reduced pressure. Purification by flash column chromatography (eluent: 2.5% EtOAc in *n*-heptane) afforded the **14c** (1.43 g, 43%) as brown oil. ^1^H NMR (400 MHz, CDCl_3_): δ (ppm) 6.81 (d, *J* = 2.2 Hz, 1H), 6.75 (d, *J* = 2.2 Hz, 1H), 5.32 (d, *J* = 1.5 Hz, 1H), 3.46 (s, 1H, NH), 2.22 (s, 3H), 2.08 (s, 3H), 2.00 (d, *J* = 1.5 Hz, 3H), 1.28 (s, 6H, (CH_3_)_2_). ^13^C NMR (100 MHz, CDCl_3_): δ (ppm) 139.0, 130.5, 129.0, 128.3, 125.4, 122.3, 121.2, 119.9, 51.8, 31.2, 20.8, 19.1, 17.0.

### 6-Methoxy-2,2,4-trimethyl-1,2-dihydroquinoline (**14d**)

A RBF (100 mL) was charged with MgSO_4_ (9.80 g, 81.2 mmol), catechol (81 mg, 0.49 mmol), and I_2_ (206 mg, 0.812 mmol). Then, 4-methoxyaniline (2.00 g, 16.2 mmol) and acetone (50 mL) were added, and the mixture was refluxed for 24 h. After cooling down, the mixture was filtered over a plug of Celite with EtOAc and the resulting org. solution was concentrated under reduced pressure. Purification by flash column chromatography (eluent: 3% EtOAc in *n*-heptane) afforded **14d** (2.34 g, 71%) as a brown oil. ^1^H NMR (400 MHz, CDCl_3_): δ (ppm) 6.69 (s, 1H), 6.61 (dd, *J* = 8.4, 2.8 Hz, 1H), 6.41 (s, 1H), 5.37 (s, 1H), 3.75 (s, 3H), 3.47 (s, 1H), 1.98 (s, 3H), 1.25 (s, 6H). ^13^C NMR (100 MHz, CDCl_3_): δ (ppm) 152.0, 137.5, 129.8, 128.5, 123.0, 113.7, 113.5, 110.1, 55.9, 51.7, 30.4, 18.6.

### 6-Chloro-2,2,4-trimethyl-1,2-dihydroquinoline (**14e**)

A μ-wave vial was charged with MgSO_4_ (6.02 g, 50.0 mmol), catechol (50 mg, 0.30 mmol), and I_2_ (127 mg, 0.500 mmol). Then, 4-chloroaniline (1.3 g, 10 mmol) and acetone (20 mL) were added, the tube was sealed, and the mixture was stirred at 110 °C 24 h. After cooling down was the mixture was filtered over a plug of Celite with EtOAc and the resulting org. solution was concentrated under reduced pressure. Purification by flash column chromatography (eluent: 2% EtOAc in *n*-heptane) afforded **14e** (1.3 g, 63%) product as yellow oil. ^1^H NMR (400 MHz, CDCl_3_): δ (ppm) 7.00 (d, *J* = 2.3 Hz, 1H), 6.92 (dd, *J* = 8.4, 2.4 Hz, 1H), 6.36 (d, *J* = 8.4 Hz, 1H), 5.35 (s, 1H), 3.67 (s, 1H), 1.96 (d, *J* = 1.5 Hz, 3H), 1.27 (s, 6H). ^13^C NMR (151 MHz, CDCl_3_): δ (ppm) 141.7, 129.6, 128.0, 127.9, 123.6, 123.2, 122.0, 114.1, 52.2, 31.0, 18.6.

### 1-(4-Methylquinazolin-2-yl)guanidine (**15a**)

To a vial containing **14a** (1.11 g, 6.43 mmol) was added 2-cyanoguanidine (1.08 g, 12.8 mmol) followed by HCl (2 M, 3.54 mL), and the mixture was refluxed for 1.5 h. Then, the mixture was allowed to cool to ambient temperature, and NaOH (15%, 2.06 mL) was added. The formed precipitate was collected with suction filtration and washed with water. The solid was sonicated 30 min in CH_2_Cl_2_ and then filtered again to afford **15a** (1.18 g, 91%) as a pale-beige solid. ^1^H NMR (400 MHz, chloroform-*d*): δ (ppm) 8.07 (dd, *J* = 8.3, 1.3 Hz, 1H), 7.79 (ddd, *J* = 8.3, 6.8, 1.4 Hz, 1H), 7.69 (d, *J* = 7.8 Hz, 1H), 7.41 (ddd, *J* = 8.2, 6.8, 1.2 Hz, 1H), 2.79 (s, 3H). ^13^C NMR (100 MHz, DMSO-*d*_6_): δ (ppm) 169.6, 161.5, 159.3, 150.1, 134.0, 126.3, 126.0, 124.0, 119.9, 22.0.

### 1-(4,6-Dimethylquinazolin-2-yl)guanidine (**15b**)

A RBF (50 mL) containing **14b** (2.10 g, 11.2 mmol) was charged with 2-cyanoguanidine (1.90 g, 22.6 mmol). Then, HCl (2 M, 6.2 mL) was added, and the mixture was stirred at 110 °C for 1.5 h. The mixture was then allowed to cool to ambient temperature and basified with NaOH (15%, 3.7 mL) and diluted with more water. The mixture was then sonicated, and the formed precipitate was then collected with suction filtration and washed with more water and Et_2_O to afford **15b** (2.16 g, 90%) as a white solid. ^1^H NMR (400 MHz, DMSO-*d*_6_): δ (ppm) 7.93 (s, 1H, H), 7.72 (s, 2H), 2.82 (s, 3H), 2.49 (s, 3H). No carbon could be obtained due to poor solubility.

### 1-(4,6,8-Trimethylquinazolin-2-yl)guanidine (**15c**)

A RBF (25 mL) containing **14c** (1.43 g, 7.10 mmol) was charged with 2-cyanoguanidine (1.20 g, 14.3 mmol). Then, HCl (2 M, 3.9 mL) was added, and the mixture was stirred at 110 °C overnight. The mixture was then allowed to cool to ambient temperature and basified with NaOH (15%, 2.3 mL) and diluted with more water. The mixture was then sonicated, and the formed precipitate was then collected with suction filtration and washed with more water and Et_2_O to afford **15c** (1.36 g, 84%) as a white solid. ^1^H NMR (400 MHz, DMSO-*d*_6_): δ (ppm) 7.60 (s, 1H), 7.43 (s, 1H), 7.13 (s, 4H, NH), 2.68 (s, 3H), 2.45 (s, 3H), 2.39 (s, 3H). No carbon could be obtained due to poor solubility.

### 1-(6-Methoxy-4-methylquinazolin-2-yl)guanidine (**15d**)

A vial containing **14d** (1.145 g, 5.63 mmol) was charged with 2-cyanoguanidine (947 mg, 11.27 mmol). Then, HCl (2 M, 3.1 mL) was added, and the mixture was stirred at 110 °C for overnight. The mixture was then allowed to cool to ambient temperature and basified with NaOH (15%, 1.8 mL) and diluted with more water. The mixture was then sonicated, and the formed precipitate was then collected with suction filtration and washed with more water and Et_2_O to afford **15d** (1.22 g, 94%) as a white solid. ^1^H NMR (400 MHz, DMSO-*d*_6_): δ (ppm) 7.55 (d, *J* = 9.1 Hz, 1H), 7.39 (dd, *J* = 9.1, 2.8 Hz, 1H), 7.30 (d, *J* = 2.8 Hz, 1H), 3.87 (s, 3H), 2.72 (s, 3H). ^13^C NMR (100 MHz, DMSO-*d*_6_): δ (ppm) 170.8, 157.5, 155.7, 152.2, 144.5, 128.7, 127.7, 122.3, 104.7, 56.3, 22.2.

### 1-(6-Chloro-4-methylquinazolin-2-yl)guanidine (**15e**)

A vial containing **14e** (1.3 g, 6.3 mmol) was charged with 2-cyanoguanidine (1.05 g, 12.5 mmol). Then, HCl (2 M, 3.44 mL) was added, and the mixture was stirred at 110 °C for 1.5 h. The mixture was then allowed to cool to ambient temperature and basified with NaOH (15%, 2 mL) and diluted with more water. The mixture was then sonicated, and the formed precipitate was then collected with suction filtration and washed with more water and Et_2_O to afford **15e** (780 mg, 53%) as a white solid. ^1^H NMR (600 MHz, DMSO-*d*_6_): δ (ppm) 8.20 (d, *J* = 2.3 Hz, 1H), 7.87 (dd, *J* = 8.9, 2.3 Hz, 1H), 7.81 (d, *J* = 8.9 Hz, 1H), 2.82 (s, 3H). ^13^C NMR (100 MHz, DMSO-*d*_6_): δ (ppm) 168.4, 162.0, 159.4, 148.5, 133.7, 127.9, 126.9, 124.5, 119.8, 21.6.

### 6-Methyl-2-((4-methylquinazolin-2-yl)amino)pyrimidin-4(3*H*)-one (**16a**)

To a vial containing **15a** (1.18 g, 5.84 mmol) were added DMF (5 mL), ethyl acetoacetate (7.45 mL, 58.4 mmol), and DIPEA (2.03 mL, 11.7 mmol), the vial was sealed, and the mixture was stirred at 120 °C for 3 days. The mixture was then diluted with Et_2_O, and the precipitate was collected with suction filtration and washed with more Et_2_O. **16a** (1 g, 65%) was then collected as a beige solid. ^1^H NMR (400 MHz, DMSO-*d*_6_): δ (ppm) 13.38 (s, 1H), 11.33 (s, 1H), 8.23 (dd, *J* = 8.3, 1.4 Hz, 1H), 7.95 (ddd, *J* = 8.4, 6.9, 1.4 Hz, 1H), 7.76 (d, *J* = 8.3 Hz, 1H), 7.58 (ddd, *J* = 8.2, 6.9, 1.2 Hz, 1H), 5.83 (s, 1H), 2.90 (s, 3H), 2.16 (s, 3H). No carbon could be obtained due to poor solubility.

### 2-((4,6-Dimethylquinazolin-2-yl)amino)-6-methylpyrimidin-4(3*H*)-one (**16b**)

A vial (2–5 mL) was charged with **15b** (250 mg, 1.16 mmol). Then, DMF (2 mL), DIPEA (405 μL, 2.33 mmol), and ethyl acetoacetate (1.48 mL, 11.6 mmol) were added sequentially, and the solution was stirred at 120 °C overnight. The mixture was then diluted with Et_2_O and sonicated. The precipitate was collected with suction filtration and washed with more Et_2_O to afford **16b** (240 mg, 74%) as a pale beige solid. ^1^H NMR (400 MHz, DMSO-*d*_6_): δ (ppm) 13.33 (s, 1H, NH), 11.21 (s, 1H, NH), 7.96 (d, *J* = 2.2 Hz, 1H), 7.76 (dd, *J* = 8.5, 2.2 Hz, 1H), 7.63 (d, *J* = 8.5 Hz, 1H), 5.80 (s, 1H), 2.85 (s, 3H), 2.48 (s, 3H), 2.15 (s, 3H). ^13^C NMR (100 MHz, DMSO-*d*_6_): δ (ppm) 170.9, 165.6, 161.1, 154.1, 151.5, 146.4, 137.1, 135.3, 125.5, 125.0, 120.4, 104.5, 23.6, 21.5, 21.0.

### 6-Methyl-2-((4,6,8-trimethylquinazolin-2-yl)amino)pyrimidin-4(3*H*)-one (**16c**)

A vial (2–5 mL) was charged with **15c** (250 mg, 1.09 mmol). Then, DMF (2 mL), DIPEA (380 μL, 2.18 mmol), and ethyl acetoacetate (1.4 mL, 11 mmol) were added sequentially, and the solution was stirred at 120 °C for 3 days. The mixture was then diluted with Et_2_O and sonicated. The precipitate was collected with suction filtration and washed with more Et_2_O to afford **16c** (295 mg, 92%) as a pale beige solid. ^1^H NMR (400 MHz, DMSO-*d*_6_): δ (ppm) 13.61 (s, 1H, NH), 11.27 (s, 1H, NH), 7.83 (s, 1H), 7.66 (s, 1H), 5.82 (s, 1H), 2.85 (s, 3H), 2.59 (s, 3H), 2.46 (s, 3H), 2.16 (s, 3H). ^13^C NMR (151 MHz, DMSO-*d*_6_): δ (ppm) 171.3, 165.7, 161.0, 153.4, 151.7, 145.4, 136.9, 134.6, 133.1, 122.6, 120.2, 104.4, 23.7, 21.7, 21.0, 17.1.

### 2-((6-Methoxy-4-methylquinazolin-2-yl)amino)-6-methylpyrimidin-4(3*H*)-one (**16d**)

A vial (2–5 mL) was charged with **15d** (231 mg, 1.00 mmol). Then, DMF (3 mL), DIPEA (348 μL, 2.00 mmol), and ethyl acetoacetate (1.28 mL, 10.2 mmol) were added sequentially, and the solution was stirred at 120 °C for 40 h. The mixture was then diluted with Et_2_O and sonicated. The precipitate was collected with suction filtration and washed with more Et_2_O to afford **16d** (210 mg, 71%) as a pale beige solid. ^1^H NMR (600 MHz, DMSO-*d*_6_): δ (ppm) 13.31 (s, 1H), 11.20 (s, 1H), 7.72 (d, *J* = 9.1 Hz, 1H), 7.61 (dd, *J* = 9.1, 2.8 Hz, 1H), 7.50 (d, *J* = 2.8 Hz, 1H), 5.80 (s, 1H), 3.94 (s, 3H), 2.89 (s, 3H), 2.15 (s, 3H). ^13^C NMR (151 MHz, DMSO-*d*_6_): δ (ppm) 170.0, 165.7, 161.1, 156.5, 153.2, 151.6, 143.8, 127.4, 127.0, 121.2, 104.6, 104.3, 55.8, 23.7, 21.8.

### 2-((6-Chloro-4-methylquinazolin-2-yl)amino)-6-methylpyrimidin-4(3*H*)-one (**16e**)

A vial (2–5 mL) was charged with **15e** (236 mg, 1.01 mmol). Then, DMF (3 mL), DIPEA (348 μL, 2.00 mmol), and ethyl acetoacetate (1.28 mL, 10.2 mmol) were added sequentially, and the solution was stirred at 120 °C overnight. The mixture was then diluted with Et_2_O and sonicated. The precipitate was collected with suction filtration and washed with more Et_2_O to afford **16e** (175 mg, 58%) as a pale beige solid. ^1^H NMR (600 MHz, DMSO-*d*_6_): δ (ppm) 13.20 (s, 1H), 11.42 (s, 1H), 8.32 (d, *J* = 2.5 Hz, 1H), 7.95 (dd, *J* = 8.9, 2.4 Hz, 1H), 7.79 (d, *J* = 8.9 Hz, 1H), 5.84 (s, 1H), 2.89 (s, 3H), 2.16 (s, 3H). ^13^C NMR (151 MHz, DMSO-*d*_6_): δ (ppm) 171.5, 165.5, 161.0, 154.8, 151.3, 146.9, 135.5, 129.5, 128.0, 125.4, 121.2, 104.8, 23.6, 21.7.

### N4-(3-(Dimethylamino)propyl)-6-methyl-N2-(4-methylquinazolin-2-yl)pyrimidine-2,4-diamine (**3**)

To a vial containing **16a** (267 mg, 1.00 mmol) in CH_3_CN (4 mL), PyAOP (573 mg, 1.10 mmol) and DBU (224 μL, 1.50 mmol) were added successively, and the mixture was stirred at 60 °C overnight. Propylamine (252 μL, 2.00 mmol) was then added, and the solution was stirred for 3 h at 60 °C. The reaction solution concentrated under reduced pressure and then diluted with water and the AQ layer was then extracted with CHCl_3_/IPA (3/1, 15 mL × 3), and the combined org. layer was dried over Na_2_SO_4_, filtered, and concentrated under reduced pressure. Purification by flash column chromatography (eluent: 15 → 20% MeOH (1% NH_4_OH) in CH_2_Cl_2_) afforded **3** (75 mg, 21%) as a pale-yellow solid. ^1^H NMR (400 MHz, DMSO-*d*_6_): δ (ppm) 9.44 (s, 1H), 8.10 (dd, *J* = 8.3, 1.3 Hz, 1H), 7.80 (ddd, *J* = 8.3, 6.8, 1.4 Hz, 1H), 7.65 (d, *J* = 8.3 Hz, 1H), 7.43 (ddd, *J* = 8.1, 6.8, 1.2 Hz, 1H), 7.17 (s, 1H), 3.65–3.14 (m, 2H), 2.81 (s, 3H), 2.31 (t, *J* = 7.0 Hz, 2H), 2.16 (s, 3H), 2.13 (s, 6H), 1.73 (p, *J* = 7.1 Hz, 2H). ^13^C NMR (100 MHz, DMSO-*d*_6_): δ (ppm) 169.2, 163.4, 161.7, 158.3, 155.5, 150.6, 133.8, 133.7, 126.6, 125.6, 124.1, 120.3, 56.6, 44.9, 38.2, 26.7, 23.2, 21.3. HRMS *m*/*z*: [M – H]^+^ calcd for C_19_H_26_N_7_^+^, 352.2244; found, 352.2244.

### N4-(3-(Dimethylamino)propyl)-N2-(4,6-dimethylquinazolin-2-yl)-6-methylpyrimidine-2,4-diamine (**4**)

A vial was charged with **16b** (100 mg, 0.356 mmol) and PyAOP (241 mg, 0.462 mmol). Then, DMF (2 mL) and DBU (80 μL, 0.54 mmol) were added and the resulting solution was stirred for 1 h. Propylamine (112 μL, 0.890 mmol) was then added, and the solution was stirred at ambient temperature overnight. The solvent was removed under reduced pressure, and the crude mixture was diluted in water and extracted with IPA/CHCl_3_ (3 × 10 mL). The combined org. layer was then dried over Na_2_SO_4_, filtered, and concentrated under reduced pressure. Purification by flash column chromatography (eluent: 15 → 20% MeOH (1% NH_4_OH) in CH_2_Cl_2_) to afford **4** (85 mg, 65%) as a pale-yellow solid. ^1^H NMR (600 MHz, DMSO-*d*_6_): δ (ppm) 7.87 (s, 1H), 7.64 (dd, *J* = 8.5, 1.8 Hz, 1H), 7.57 (d, *J* = 8.5 Hz, 1H), 7.14 (s, 1H), 5.94 (s, 1H), 3.27–3.21 (m, 2H), 2.78 (s, 3H), 2.47 (s, 3H), 2.28 (t, *J* = 7.1 Hz, 2H), 2.15 (s, 3H), 2.11 (s, 6H), 1.71 (p, *J* = 7.1 Hz, 2H). ^13^C NMR (151 MHz, DMSO-*d*_6_): δ (ppm) 168.3, 163.4, 158.4, 155.0, 149.1, 135.6, 133.5, 126.4, 124.4, 120.2, 97.9, 56.7, 45.0, 38.1, 26.8, 23.3, 21.3, 21.0. HRMS *m*/*z*: [M – H]^+^ calcd for C_20_H_28_N_7_^+^, 366.2401; found, 366.2425.

### N4-(3-(Dimethylamino)propyl)-6-methyl-N2-(4,6,8-trimethylquinazolin-2-yl)pyrimidine-2,4-diamine (**5**)

A vial was charged with **16c** (100 mg, 0.339 mmol) and PyAOP (230 mg, 0.441 mmol). Then, DMF (2 mL) and DBU (76 μL, 0.51 mmol) were added and the resulting solution was stirred for 1 h. Propylamine (107 μL, 0.850 mmol) was then added, and the solution was stirred at ambient temperature overnight. The solvent was removed under reduced pressure, and the crude mixture was diluted in water and extracted with IPA/CHCl_3_ (3 × 10 mL). The combined org. layer was then dried over Na_2_SO_4_, filtered, and concentrated under reduced pressure. Purification by flash column chromatography (eluent: 15 → 25% MeOH (1% NH_4_OH) in CH_2_Cl_2_) to afford **5** (82 mg, 64%) as a pale-yellow solid. ^1^H NMR (600 MHz, DMSO-*d*_6_): δ (ppm) 7.69 (s, 1H), 7.50 (s, 1H), 7.05 (s, 1H), 5.93 (s, 1H), 3.25 (app. s, 2H), 2.76 (s, 3H), 2.56 (s, 3H), 2.42 (s, 3H), 2.22 (t, *J* = 7.1 Hz, 2H), 2.15 (s, 3H), 2.07 (s, 6H), 1.63 (p, *J* = 7.1 Hz, 2H). ^13^C NMR (151 MHz, DMSO-*d*_6_): δ (ppm) 168.3, 163.4, 158.5, 154.1, 148.1, 135.4, 134.3, 132.7, 121.9, 120.0, 97.7, 56.8, 45.1, 38.2, 27.0, 23.3, 21.4, 21.1, 16.9. HRMS *m*/*z*: [M – H]^+^ calcd for C_21_H_30_N_7_^+^, 380.2557; found, 380.2559.

### N4-(3-(Dimethylamino)propyl)-N2-(6-methoxy-4-methylquinazolin-2-yl)-6-methylpyrimidine-2,4-diamine (**6**)

A vial was charged with **16d** (74 mg, 0.25 mmol) and PyAOP (169 mg, 0.325 mmol). Then, DMF (2.5 mL) and DBU (56 μL, 0.38 mmol) were added and the resulting solution was stirred for 1 h. Propylamine (79 μL, 0.63 mmol) was then added, and the solution was stirred at ambient temperature overnight. The solvent was removed under reduced pressure, and the crude mixture was diluted in water and extracted with IPA/CHCl_3_ (3 × 10 mL). The combined org. layer was then dried over Na_2_SO_4_, filtered, and concentrated under reduced pressure. Purification by flash column chromatography (eluent: 15 → 20% MeOH (1% NH_4_OH) in CH_2_Cl_2_) to afford **6** (40 mg, 42%) as a pale-yellow solid. ^1^H NMR (600 MHz, DMSO-*d*_6_): δ (ppm) 9.22 (s, 1H), 7.62 (d, *J* = 9.1 Hz, 1H), 7.48 (dd, *J* = 9.1, 2.7 Hz, 1H), 7.39 (d, *J* = 2.8 Hz, 1H), 7.13 (s, 1H), 3.91 (s, 3H), 3.41–3.29 (m, 2H), 2.80 (s, 3H), 2.36–2.24 (m, 2H), 2.14 (s, 3H), 2.12 (s, 6H), 1.71 (p, *J* = 7.0 Hz, 2H). ^13^C NMR (151 MHz, DMSO-*d*_6_): δ (ppm) 167.5, 163.3, 158.5, 155.6, 154.2, 146.3, 128.2, 125.7, 120.8, 103.9, 97.7, 56.7, 55.6, 45.0, 38.1, 26.9, 23.3, 21.5. HRMS *m*/*z*: [M – H]^+^ calcd for C_20_H_28_N_7_O^+^, 382.2350; found, 382.2351.

### N2-(6-Chloro-4-methylquinazolin-2-yl)-N4-(3-(dimethylamino)propyl)-6-methylpyrimidine-2,4-diamine (**7**)

A vial was charged with **16e** (75 mg, 0.25 mmol) and PyAOP (169 mg, 0.325 mmol). Then, DMF (2.5 mL) and DBU (56 μL, 0.38 mmol) were added and the resulting solution was stirred for 1 h. Propylamine (79 μL, 0.63 mmol) was then added, and the solution was stirred at ambient temperature overnight. The solvent was removed under reduced pressure, and the crude mixture was diluted in water and extracted with IPA/CHCl_3_ (3 × 10 mL). The combined org. layer was then dried over Na_2_SO_4_, filtered, and concentrated under reduced pressure. Purification by flash column chromatography (eluent: 15 → 20% MeOH (1% NH_4_OH) in CH_2_Cl_2_) to afford **7** (60 mg, 62%) as a pale-yellow solid. ^1^H NMR (600 MHz, DMSO-*d*_6_): δ (ppm) 9.53 (s, 1H), 8.17 (d, *J* = 2.3 Hz, 1H), 7.80 (dd, *J* = 8.9, 2.3 Hz, 1H), 7.65 (d, *J* = 8.9 Hz, 1H), 7.18 (s, 1H), 5.97 (s, 1H), 3.29–3.23 (m, 2H), 2.80 (s, 3H), 2.31 (t, *J* = 6.9 Hz, 2H), 2.15 (s, 3H), 2.14 (s, 6H), 1.72 (t, *J* = 7.1 Hz, 2H). ^13^C NMR (151 MHz, DMSO-*d*_6_): δ (ppm) 168.9, 163.4, 158.1, 155.8, 149.4, 134.1, 128.7, 127.8, 124.7, 120.9, 98.3, 56.6, 44.8, 38.1, 26.7, 23.3, 21.4. HRMS *m*/*z*: [M – H]^+^ calcd for C_19_H_25_ClN_7_^+^, 386.1854; found, 386.1853.

### Quinoxalin-2(1*H*)-one (**17**)

A flask was charged with *o*-phenylidendiamine (1.50 g, 13.9 mmol). Then, EtOH (6 mL) and ethyl glyoxalate (50% in PhCH_3_, 3.30 mL, 16.6 mmol) were added and the mixture was stirred at 60 °C overnight. The reaction was then allowed to cool to ambient temperature, and the solids were collected with suction filtration and washed with water to afford **17** (1.86 g, 92%) as a pale beige solid. ^1^H NMR (400 MHz, DMSO-*d*_6_): δ (ppm) 12.42 (s, 1H), 8.17 (s, 1H), 7.78 (dd, *J* = 8.4, 1.4 Hz, 1H), 7.60–7.49 (m, 1H), 7.35–7.26 (m, 2H). The data is consistent with that reported in the literature.^38^

### 2-Chloroquinoxaline (**18**)

To a RBF (25 mL) containing **17** (500 mg, 3.42 mmol) was added POCl_3_ (3.20 mL, 34.3 mmol), and the mixture was refluxed for 2 h. Then, the reaction was allowed to cool to ambient temperature and poured onto ice water (ca. 20 mL). The organic layer was then extracted with EtOAc (3 × 20 mL), and the combined org. layer was then washed with sat. NaHCO_3_ (20 mL), water (20 mL), and sat. NaCl sol. (20 mL). The resulting org. layer was dried over Na_2_SO_4_, filtered, and concentrated under reduced pressure to afford **18** (550 mg, 98%) as a pale-brown solid. ^1^H NMR (400 MHz, CDCl_3_): δ (ppm) 8.79 (s, 1H), 8.15–8.10 (m, 1H), 8.06–8.00 (m, 1H), 7.85–7.75 (m, 2H). The data is consistent with that reported in the literature.^39^

### 1-(Quinoxalin-2-yl)guanidine (**19**)

To a μ-wave vial (10–20 mL) were added **18** (300 mg, 1.82 mmol), guanidine·HCl (348 mg, 3.64 mmol), and K_2_CO_3_ (756 mg, 5.47 mmol). Then, CH_3_CN (12 mL) was added, and the reaction was heated using MWI (140 °C, 3 h). If **18** remained on TLC, the reaction time was extended by another 30 min. The mixture was then concentrated under reduced pressure and diluted with water ∼20 mL. The resulting AQ layer was extracted with EtOAc (20 mL × 4), and the combined org. layer was dried over Na_2_SO_4_, filtered, a concentrated under reduced pressure. **19** (247 mg, 72%) was then obtained as a yellow solid. ^1^H NMR (400 MHz, DMSO-*d*_6_): δ (ppm) 8.22 (s, 1H), 7.76 (d, *J* = 8.1 Hz, 1H), 7.58 (dd, *J* = 19.0, 7.6 Hz, 2H), 7.37 (t, *J* = 7.6 Hz, 1H). 7.29 (s, 4H, NH). ^13^C NMR (100 MHz, DMSO-*d*_6_): δ (ppm) 160.2, 157.8, 147.9, 140.4, 136.6, 129.2, 128.2, 125.8, 124.2.

### 6-Methyl-2-(quinoxalin-2-ylamino)pyrimidin-4(3*H*)-one (**20**)

A vial was charged with **19** (130 mg, 0.694 mmol). Then, DMF (1 mL), DIPEA (242 μL, 1.39 mmol), and ethyl acetoacetate (886 μL, 6.94 mmol) were added sequentially, and the solution was stirred at 120 °C for 24 h. The mixture was then diluted with Et_2_O, and the precipitate was collected with suction filtration. The solids were washed with more Et_2_O to afford **20** (80 mg, 46%) as a beige solid. ^1^H NMR (600 MHz, DMSO-*d*_6_): δ (ppm) 12.85 (s, 1H), 11.66 (s, 1H, NH), 8.77 (s, 1H, NH), 7.98 (d, *J* = 8.2 Hz, 1H), 7.83 (dd, *J* = 8.3, 1.4 Hz, 1H), 7.81–7.76 (m, 1H), 7.68–7.63 (m, 1H), 5.83 (s, 1H), 2.18 (s, 3H). ^13^C NMR (151 MHz, DMSO-*d*_6_): δ (ppm) 161.1, 151.4, 148.5, 140.9, 138.2, 137.9, 131.0, 128.8, 128.3, 127.3, 126.3, 104.4, 22.7.

### N4-(3-(Dimethylamino)propyl)-6-methyl-N2-(quinoxalin-2-yl)pyrimidine-2,4-diamine (**8**)

A vial was charged with **20** (60 mg, 0.24 mmol) and PyAOP (160 mg, 0.307 mmol). Then, DMF (1 mL) and DBU (53 μL, 0.36 mmol) were added and the resulting solution was stirred at ambient temperature for 1 h. Propylamine (88 μL, 0.59 mmol) was then added, and the solution was stirred at ambient temperature overnight. The solvent was removed under reduced pressure, and the mixture was diluted with water. The AQ layer was then extracted with CHCl_3_/IPA (3/1, 10 mL × 3), and the combined org. layer was dried over Na_2_SO_4_, filtered, and concentrated under reduced pressure. Purification by flash column chromatography (eluent: 6 → 12% MeOH (1% NH_4_OH) in CH_2_Cl_2_) afforded **8** (61 mg, 76%) as a yellow solid. ^1^H NMR (600 MHz, DMSO-*d*_6_): δ (ppm) 9.88 (s, 1H), 9.80 (s, 1H, NH), 7.95 (d, *J* = 8.2 Hz, 1H), 7.77 (d, *J* = 8.3 Hz, 1H), 7.70 (td, *J* = 7.5, 6.7, 1.5 Hz, 1H), 7.58 (td, *J* = 7.5, 6.6, 1.5 Hz, 1H), 7.32 (s, 1H, NH), 5.98 (s, 1H), 3.31–3.20 (m, 2H), 2.36 (t, *J* = 7.2 Hz, 2H), 2.18 (s, 9H (merged peaks)), 1.70 (p, *J* = 7.1 Hz, 2H). ^13^C NMR (151 MHz, DMSO-*d*_6_): δ (ppm) 163.2, 158.2, 149.0, 140.8, 140.8, 138.0, 130.0, 128.5, 126.7, 126.3, 98.0, 56.5, 44.9, 38.1, 26.6, 25.8. HRMS *m*/*z*: [M – H]^+^ calcd for C_18_H_24_N_7_^+^, 338.2088; found, 338.2085.

### 3-Methylquinoxalin-2(1*H*)-one (**21a**)

A RBF was charged with *o*-phenylidendiamine (1.62 g, 15.0 mmol) and sodium pyruvate (1.65 g, 15.0 mmol) in aq. acetic acid (20%, 25 mL). The reaction was stirred at ambient temperature for 3 h. The resulting precipitate was filtered off and washed with water to afford **21a** (1.95 g, 81%) as a pale-brown solid. ^1^H NMR (400 MHz, CDCl_3_): δ (ppm) 11.61 (s, 1H), 7.81 (d, *J* = 8.0 Hz, 1H), 7.49 (t, *J* = 7.8 Hz, 1H), 7.39–7.30 (m, 2H), 2.64 (s, 3H). The data is consistent with that reported in the literature.^40^

### 3,6,7-Trimethylquinoxalin-2(1*H*)-one (**21b**)

A RBF (50 mL) was charged with 4,5-dimethylbenzene-1,2-diamine (1.00 g, 7.34 mmol) and sodium pyruvate (808 mg, 7.34 mmol) in aq. acetic acid (20%, 25 mL). The reaction was stirred at ambient temperature for 3 h. The resulting precipitate was filtered off and washed with water to afford **21b** (1.13 g, 82%) as a pale-brown solid. ^1^H NMR (600 MHz, DMSO-*d*_6_): δ (ppm) 12.14 (s, 1H), 7.44 (s, 1H), 7.01 (s, 1H), 2.36 (s, 3H), 2.27 (s, 3H), 2.25 (s, 3H). ^13^C NMR (151 MHz, DMSO-*d*_6_): δ (ppm) 172.0, 157.7, 155.0, 138.6, 131.5, 130.2, 129.9, 127.8, 115.3, 20.5, 19.7, 18.9.

### 6,7-Dichloro-3-methylquinoxalin-2(1*H*)-one (**21c**)

A RBF (100 mL) was charged with 3,4-dichloro-*o*-phenylenediamine (2.00 g, 11.3 mmol) and sodium pyruvate (1.37 g, 12.4 mmol) followed by aqueous acetic acid (20%, 50 mL). The reaction was stirred at ambient temperature overnight. The resulting precipitate was filtered off, washed with water, and dried. **21c** (2.43 g, 94%) was obtained as a brown solid. ^1^H NMR (400 MHz, DMSO-*d*_6_): δ (ppm) 12.43 (s, 1H, NH), 7.94 (d, *J* = 5.3 Hz, 1H, H-2), 7.41 (d, *J* = 5.3 Hz, 1H, H-1), 2.39 (s, 3H, CH_3_). ^13^C NMR (100 MHz, DMSO-*d*_6_): δ (ppm) 161.4, 154.5, 131.9, 131.2, 131.1, 128.9, 124.7, 116.2, 20.6.

### 2-Chloro-3-methylquinoxaline (**22a**)

To a RBF containing **21a** (1.94 g, 12.1 mmol) was added POCl_3_ (9.70 mL, 104 mmol), and the mixture was stirred at 110 °C overnight. Then, the reaction was allowed to cool to ambient temperature and poured onto ice water (ca. 20 mL). The organic layer was then extracted with EtOAc (3 × 30 mL), and the combined org. layer was then washed with sat. NaHCO_3_ (20 mL), water (20 mL), and sat. NaCl sol. (20 mL). The resulting org. layer was dried over Na_2_SO_4_, filtered, and concentrated under reduced pressure to afford **22a** (1.0 g, 46%) as a pale-brown solid. ^1^H NMR (400 MHz, CDCl_3_): δ (ppm) 8.06–8.01 (m, 1H), 8.00–7.96 (m, 1H), 7.79–7.68 (m, 2H), 2.85 (s, 3H). ^13^C NMR (151 MHz, CDCl_3_): δ (ppm) 152.9, 148.0, 141.1, 141.0, 130.2, 130.1, 128.6, 128.3, 23.5.

### 2-Chloro-3,6,7-trimethylquinoxaline (**22b**)

To a RBF containing **21b** (500 mg, 2.66 mmol) was added POCl_3_ (2.50 mL, 26.8 mmol), and the mixture was stirred at 110 °C overnight. Then, the reaction was allowed to cool to ambient temperature and poured onto ice water (ca. 20 mL). The organic layer was then extracted with EtOAc (3 × 30 mL), and the combined org. layer was then washed with sat. NaHCO_3_ (20 mL), water (20 mL), and sat. NaCl sol. (20 mL). The resulting org. layer was dried over Na_2_SO_4_, filtered, and concentrated under reduced pressure to afford **22b** (402 mg, 73%) as a dark-brown solid. ^1^H NMR (600 MHz, DMSO-*d*_6_): δ (ppm) 7.78 (s, 1H), 7.73 (s, 1H), 2.70 (s, 3H), 2.43 (s, 6H). ^13^C NMR (151 MHz, DMSO-*d*_6_): δ (ppm) 151.4, 146.2, 140.8, 140.7, 139.2, 139.1, 127.0, 126.6, 22.8, 19.8, 19.7.

### 2,6,7-Trichloro-3-methylquinoxaline (**22c**)

To a RBF containing **21c** (790 mg, 3.45 mmol) was added POCl_3_ (3.20 mL, 34.3 mmol), and the mixture was stirred at 110 °C overnight. Then, the reaction was allowed to cool to ambient temperature, and the volatiles were removed under reduced pressure. The resulting crude mixture was diluted with sat. NaHCO_3_ (150 mL), and the aq. layer was then extracted with DCM (3 × 75 mL). If the organic layer is sluggish, filter once over Celite before the wash. The combined org. layer was then washed with sat. NaCl sol. (2 × 50 mL). The resulting org. layer was dried over Na_2_SO_4_, filtered over Celite, and concentrated under reduced pressure. **22c** (480 mg, 56%) was then obtained as a dark-brown solid without further purification. ^1^H NMR (400 MHz, DMSO-*d*_6_): δ (ppm) 8.38 (s, 1H), 8.36 (s, 1H), 2.75 (s, 3H). ^13^C NMR (100 MHz, DMSO-*d*_6_): δ (ppm) 154.8, 148.9, 139.3, 139.1, 133.1, 133.0, 129.1, 128.8, 23.1.

### 1-(3-Methylquinoxalin-2-yl)guanidine (**23a**)

To a μ-wave vial (10–20 mL) was added **22a** (500 mg, 2.80 mmol), guanidine·HCl (535 mg, 5.60 mmol), and K_2_CO_3_ (1.16 g, 8.40 mmol). Then, DMSO (15 mL) was added, and the reaction was heated using MWI (160 °C, 75 min). The mixture was then diluted with water and extracted with EtOAc (4 × 30 mL). The combined org. layer was washed with water (3 × 10 mL) and once with sat. NaCl sol. (1 × 15 mL) and dried over MgSO_4_. The solvent was removed under reduced pressure to afford **23a** (500 mg, 89%) as a yellow solid. ^1^H NMR (400 MHz, CDCl_3_): δ (ppm) 7.85 (dd, *J* = 8.1, 1.5 Hz, 1H), 7.68–7.59 (m, 1H), 7.50 (ddd, *J* = 8.3, 6.9, 1.5 Hz, 1H), 7.42 (ddd, *J* = 8.4, 7.0, 1.5 Hz, 1H), 2.71 (s, 3H). ^13^C NMR (100 MHz, CDCl_3_): δ (ppm) 158.4, 156.3, 154.9, 139.8, 137.4, 128.3, 127.8, 125.9, 125.5, 22.6.

### 1-(3,6,7-Trimethylquinoxalin-2-yl)guanidine (**23b**)

To a MW vial (2–5 mL) were added **22b** (200 mg, 0.968 mmol), guanidine·HCl (185 mg, 1.94 mmol), and K_2_CO_3_ (401 mg, 2.90 mmol). Then, DMSO (3 mL) was added, and the reaction was heated using MWI (160 °C, 75 min). The mixture was then diluted with water and extracted with EtOAc (4 × 20 mL). The organic layer was washed with water (3 × 20 mL), once with sat. NaCl sol. (20 mL), and dried over Na_2_SO_4_. The solvent was removed under reduced pressure to afford **23b** (161 mg, 73%) as a dark brown solid. ^1^H NMR (400 MHz, DMSO-*d*_6_): δ (ppm) 7.45 (s, 1H), 7.35 (s, 1H), 7.20 (s, 4H, NH), 2.50 (s, 3H) 2.33 (s, 3H), 2.32 (s, 3H). ^13^C NMR (100 MHz, DMSO-*d*_6_): δ (ppm) 159.3, 153.2, 138.2, 137.1, 134.8, 133.1, 127.1, 126.9, 125.0, 22.3, 19.7, 19.4.

### 1-(6,7-Dichloro-3-methylquinoxalin-2-yl)guanidine (**23c**)

To a MW vial (2–5 mL) were added **22c** (300 mg, 01.21 mmol), guanidine·HCl (232 mg, 2.43 mmol), and K_2_CO_3_ (503 mg, 3.64 mmol). Then, DMSO (5 mL) was added, and the reaction was heated using MWI (160 °C, 75 min). The mixture was then diluted with water and extracted with CHCl_3_:IPA (3:1, 3 × 15 mL). If the organic layer is sluggish, filter once over Celite with CH_2_Cl_2_before the wash. The organic layer was washed with sat. NaCl sol. (5 × 15 mL) and dried over Na_2_SO_4_, filtered, and concentrated under reduced pressure. **23c** [230 mg, 70% (residual DMSO calculated for)] was then obtained as a dark-brown solid. ^1^H NMR (400 MHz, DMSO-*d*_6_): δ (ppm) 7.87 (s, 1H), 7.84 (s, 1H), 7.36 (s, 4H, NH), 2.51 (s, 3H). ^13^C NMR (100 MHz, DMSO-*d*_6_): δ (ppm) 160.2, 157.1, 156.7, 139.7, 135.0, 130.0, 128.0, 125.9, 125.2, 22.4.

### 6-Methyl-2-((3-methylquinoxalin-2-yl)amino)pyrimidin-4(3*H*)-one (**24a**)

A vial was charged with **23a** (167 mg, 0.830 mmol). Then, DMF (2 mL), DIPEA (289 μL, 1.66 mmol), and ethyl acetoacetate (1.06 mL, 8.30 mmol) were added sequentially, and the solution was stirred at 120 °C for 24 h. The mixture was then diluted with Et_2_O, and the precipitate was collected with suction filtration. The solids were washed with more Et_2_O to afford **24a** (166 mg, 59%) as a beige solid. ^1^H NMR (400 MHz, DMSO-*d*_6_): δ (ppm) 13.17 (s, 1H), 12.19 (s, 1H), 7.85 (d, *J* = 7.9 Hz, 1H), 7.74 (d, *J* = 8.1 Hz, 1H), 7.65 (s, 1H), 7.56 (s, 1H), 5.64 (s, 1H), 2.68 (s, 3H), 2.21 (s, 3H). No carbon NMR could be obtained due to poor solubility.

### 6-Methyl-2-((3,6,7-trimethylquinoxalin-2-yl)amino)pyrimidin-4(3*H*)-one (**24b**)

A vial was charged with **23b** (200 mg, 0.872 mmol). Then, DMF (2 mL), DIPEA (304 μL, 1.75 mmol), and ethyl acetoacetate (1.11 mL, 8.72 mmol) were added sequentially, and the solution was stirred at 120 °C for 24 h. The mixture was then diluted with Et_2_O, and the precipitate was collected with suction filtration. The solids were washed with more Et_2_O to afford **24b** (152 mg, 59%) as a dark-brown solid. ^1^H NMR (400 MHz, DMSO-*d*_6_): δ (ppm) 13.23 (s, 1H, NH), 12.09 (s, 1H, NH), 7.61 (s, 1H), 7.52 (s, 1H), 5.65 (s, 1H), 2.65 (s, 3H), 2.40 (s, 3H), 2.38 (s, 3H), 2.20 (s, 3H). No carbon NMR could be obtained due to poor solubility.

### 2-((6,7-Dichloro-3-methylquinoxalin-2-yl)amino)-6-methylpyrimidin-4(3*H*)-one (**24c**)

A vial was charged with **23c** (260 mg, 0.963 mmol). Then, DMF (3 mL), DIPEA (335 μL, 1.92 mmol), and ethyl acetoacetate (1.23 mL, 9.64 mmol) were added sequentially, and the solution was stirred at 120 °C for 24 h. The mixture was then diluted with Et_2_O, and the precipitate was collected with suction filtration. The solids were washed with more Et_2_O to afford **24c** (152 mg, 59%) as a dark-brown solid. ^1^H NMR (400 MHz, DMSO-*d*_6_): δ (ppm) 12.73 (s, 1H), 12.15 (s, 1H), 8.00 (s, 1H), 7.92 (s, 1H), 5.65 (s, 1H), 2.60 (s, 3H), 2.21 (s, 3H). ^13^C NMR (151 MHz, DMSO-*d*_6_): δ (ppm) 162.3, 161.1, 156.1, 154.4, 150.8, 137.8, 136.1, 131.1, 128.4, 128.0, 126.4, 101.8, 22.0, 19.0.

### N4-(3-(Dimethylamino)propyl)-6-methyl-N2-(3-methylquinoxalin-2-yl)pyrimidine-2,4-diamine (**9**)

A vial was charged with **24a** (166 mg, 0.49 mmol) and PyAOP (331 mg, 0.634 mmol). Then, DMF (5 mL) and DBU (109 μL, 0.732 mmol) were added and the resulting solution was stirred at ambient temperature for 1 h. Propylamine (92 μL, 0.73 mmol) was then added, and the solution was stirred at ambient temperature overnight. The solvent was removed under reduced pressure, and the mixture was diluted with water. The AQ layer was then extracted with CHCl_3_/IPA (3/1, 15 mL × 3), and the combined org. layer was dried over Na_2_SO_4_, filtered, and concentrated under reduced pressure. Purification by flash column chromatography (eluent: 6 → 10% MeOH (1% NH_4_OH) in CH_2_Cl_2_) afforded **9** (78 mg, 46%) as a yellow solid. ^1^H NMR (400 MHz, DMSO-*d*_6_): δ (ppm) 9.26 (s, 1H), 7.93 (d, *J* = 7.9 Hz, 1H), 7.81 (d, *J* = 8.0 Hz, 1H), 7.67 (q, *J* = 8.8, 7.8 Hz, 2H), 7.19 (s, 1H), 5.90 (s, 1H), 3.30 (s, 2H), 3.17 (s, 2H), 2.59 (s, 3H), 2.37–2.31 (m, 6H), 2.12 (s, 3H), 1.67 (d, *J* = 9.8 Hz, 2H). ^13^C NMR (100 MHz, DMSO-*d*_6_): δ (ppm) 163.3, 159.7, 152.2, 148.8, 139.7, 138.8, 129.0, 128.9, 127.7, 127.4, 127.0, 97.4, 55.6, 43.5, 37.5, 25.5, 23.1, 22.2. HRMS *m*/*z*: [M – H]^+^ calcd for C_19_H_26_N_7_^+^, 352.2244; found, 352.2242.

### N4-(3-(Dimethylamino)propyl)-6-methyl-N2-(3,6,7-trimethylquinoxalin-2-yl)pyrimidine-2,4-diamine (**10**)

A vial was charged with **24b** (74 mg, 0.25 mmol) and PyAOP (169 mg, 0.322 mmol). Then, DMF (1.5 mL) and DBU (56 μL, 0.38 mmol) were added and the resulting solution was stirred at ambient temperature for 1 h. Propylamine (79 μL, 0.63 mmol) was then added, and the solution was stirred at ambient temperature overnight. The solvent was removed under reduced pressure, and the mixture was diluted with water. The AQ layer was then extracted with CHCl_3_/IPA (3/1, 15 mL × 3), and the combined org. layer was dried over Na_2_SO_4_, filtered, and concentrated under reduced pressure. Purification by flash column chromatography (eluent: 10 → 20% MeOH (1% NH_4_OH) in CH_2_Cl_2_) afforded **10** (40 mg, 42%) as a dark-yellow solid. ^1^H NMR (600 MHz, DMSO-*d*_6_): δ (ppm) 9.10 (s, 1H), 7.69 (s, 1H), 7.59 (s, 1H), 7.14 (s, 1H), 5.86 (s, 1H), 3.32 (s, 2H), 3.17–3.10 (m, 2H), 2.54 (s, 3H), 2.42 (d, *J* = 2.6 Hz, 6H), 2.26 (s, 6H), 2.10 (s, 3H), 1.67–1.59 (m, 2H). ^13^C NMR (151 MHz, DMSO-*d*_6_): δ 163.3, 160.0, 151.1, 147.8, 138.6, 138.0, 137.4, 126.9, 126.4, 97.0, 55.9, 44.0, 37.6, 25.9, 23.3, 22.0, 19.7, 19.7. HRMS *m*/*z*: [M – H]^+^ calcd for C_21_H_30_N_7_^+^, 380.2557; found, 380.2561.

### N2-(6,7-Dichloro-3-methylquinoxalin-2-yl)-N4-(3-(dimethylamino)propyl)-6-methylpyrimidine-2,4-diamine (**11**)

A vial was charged with **24c** (100 mg, 0.298 mmol) and PyAOP (202 mg, 0.387 mmol). Then, DMF (3 mL) and DBU (67 μL, 0.45 mmol) were added and the resulting solution was stirred at ambient temperature for 1 h. Propylamine (94 μL, 0.75 mmol) was then added, and the solution was stirred at ambient temperature overnight. The solvent was removed under reduced pressure, and the mixture was diluted with water. The AQ layer was then extracted with CHCl_3_/IPA (3/1, 15 mL × 3), and the combined org. layer was dried over Na_2_SO_4_, filtered, and concentrated under reduced pressure. Purification by flash column chromatography (eluent: 8 → 12% MeOH (1% NH_4_OH) in CH_2_Cl_2_) afforded **11** (44 mg, 35%) as a dark-brown solid. ^1^H NMR (600 MHz, DMSO-*d*_6_): δ (ppm) 9.40 (s, 1H), 8.15 (s, 1H), 8.01 (s, 1H), 7.25 (s, 1H), 5.93 (s, 1H), 3.23–3.10 (m, 2H), 2.59 (s, 3H), 2.24 (s, 2H), 2.16 (s, 3H), 2.11 (s, 6H), 1.63 (p, *J* = 7.1 Hz, 2H). ^13^C NMR (151 MHz, DMSO-*d*_6_): δ (ppm) 174.5, 163.2, 159.0, 153.0, 149.6, 139.1, 137.3, 131.1, 128.5, 127.6, 98.0, 56.5, 44.8, 44.7, 38.0, 26.5, 22.2. HRMS *m*/*z*: [M – H]^+^ calcd for C_19_H_24_Cl_2_N_7_^+^, 420.1465; found, 420.1460.

### *N*-(4,6-Dimethylpyrimidin-2-yl)-4-methylquinazolin-2-amine (**25**)

A vial was charged with **15a** (150 mg, 0.866 mmol). Then, DMF (3 mL), DIPEA (302 μL, 1.74 mmol), and acetylacetone (885 μL, 8.66 mmol) were added sequentially and the solution was stirred at 120 °C for 24 h. The solution was concentrated under reduced pressure and then diluted with water. The precipitate was collected with suction filtration. The solids were washed with more water and Et_2_O to afford **25** (71 mg, 31%) as a beige solid. ^1^H NMR (400 MHz, DMSO-*d*_6_): δ (ppm) 9.92 (s, 1H), 8.12 (d, *J* = 8.2 Hz, 1H), 7.81 (t, *J* = 7.7 Hz, 1H), 7.66 (d, *J* = 8.4 Hz, 1H), 7.46 (t, *J* = 7.5 Hz, 1H), 6.82 (s, 1H), 2.82 (s, 3H), 2.34 (s, 6H). ^13^C NMR (151 MHz, DMSO-*d*_6_): δ (ppm) 169.9, 167.6, 159.3, 155.8, 151.1, 134.3, 127.1, 126.2, 124.9, 121.0, 114.2, 40.4, 23.9, 21.8. HRMS *m*/*z*: [M – H]^+^ calcd for C_15_H_16_N_5_^+^, 266.1400; found, 266.1402.

### *N*-(4,6-Dimethylpyrimidin-2-yl)-4,6-dimethylquinazolin-2-amine (**26**)

A vial was charged with **15b** (200 mg, 0.929 mmol). Then, DMF (4 mL), DIPEA (324 μL, 1.86 mmol), and acetylacetone (949 μL, 9.29 mmol) were added sequentially and the solution was stirred at 120 °C for 24 h. The solution was concentrated under reduced pressure and then diluted with water. The precipitate was collected with suction filtration. The solids were washed with more water and Et_2_O to afford **26** (119 mg, 46%) as a beige solid. ^1^H NMR (400 MHz, DMSO-*d*_6_): δ (ppm) 9.84 (s, 1H), 7.90 (s, 1H), 7.66 (dd, *J* = 8.6, 1.9 Hz, 1H), 7.58 (d, *J* = 8.5 Hz, 1H), 6.79 (s, 1H), 2.79 (s, 3H), 2.48 (s, 3H), 2.33 (s, 6H). ^13^C NMR (151 MHz, DMSO-*d*_6_): δ (ppm) 168.6, 167.0, 158.9, 154.8, 149.0, 135.8, 133.8, 126.5, 124.4, 120.5, 113.5, 23.5, 21.3, 21.0. HRMS *m*/*z*: [M – H]^+^ calcd for C_16_H_18_N_5_^+^, 280.1557; found, 280.1551.

### *N*-(4,6-Dimethylpyrimidin-2-yl)-4,6,8-trimethylquinazolin-2-amine (**27**)

A vial was charged with **15c** (200 mg, 0.872 mmol). Then, DMF (4 mL), DIPEA (304 μL, 1.75 mmol), and acetylacetone (891 μL, 8.72 mmol) were added sequentially and the solution was stirred at 120 °C for 24 h. The solution was concentrated under reduced pressure and then diluted with water. The precipitate was collected with suction filtration. The solids were washed with more water and Et_2_O to afford **27** (124 mg, 49%) as a beige solid. ^1^H NMR (400 MHz, DMSO-*d*_6_): δ (ppm) 9.80 (s, 1H), 7.72 (s, 1H), 7.52 (s, 1H), 6.79 (s, 1H), 2.78 (s, 3H), 2.56 (s, 3H), 2.44 (s, 3H), 2.34 (s, 6H). ^13^C NMR (151 MHz, DMSO-*d*_6_): δ (ppm) 168.5, 166.9, 158.9, 153.8, 148.0, 135.4, 134.5, 133.0, 121.9, 120.3, 113.3, 23.5, 21.4, 21.1, 16.6. HRMS *m*/*z*: [M – H]^+^ calcd for C_17_H_20_N_5_^+^, 294.1713; found, 294.1711.

### *N*-(4,6-Dimethylpyrimidin-2-yl)-6-methoxy-4-methylquinazolin-2-amine (**28**)

A vial was charged with **15d** (200 mg, 0.865 mmol). Then, DMF (4 mL), DIPEA (301 μL, 1.73 mmol), and acetylacetone (884 μL, 8.65 mmol) were added sequentially and the solution was stirred at 120 °C for 24 h. The solution was concentrated under reduced pressure and then diluted with water. The precipitate was collected with suction filtration. The solids were washed with more water and Et_2_O to afford **28** (89 mg, 35%) as a beige solid. ^1^H NMR (400 MHz, DMSO-*d*_6_): δ (ppm) 9.77 (s, 1H), 7.63 (d, *J* = 9.1 Hz, 1H), 7.49 (dd, *J* = 9.1, 2.8 Hz, 1H), 7.40 (d, *J* = 2.8 Hz, 1H), 6.77 (s, 1H), 3.92 (s, 3H), 2.81 (s, 3H), 2.32 (s, 6H). ^13^C NMR (151 MHz, DMSO-*d*_6_): δ (ppm) 167.8, 167.0, 159.1, 155.8, 154.0, 146.3, 128.3, 125.8, 121.1, 113.3, 103.9, 55.6, 23.5, 21.5. HRMS *m*/*z*: [M – H]^+^ calcd for C_16_H_18_N_5_O^+^, 296.1506; found, 296.1509.

### 6-Chloro-*N*-(4,6-dimethylpyrimidin-2-yl)-4-methylquinazolin-2-amine (**29**)

A vial was charged with **15e** (200 mg, 0.849 mmol). Then, DMF (4 mL), DIPEA (296 μL, 1.70 mmol) and acetylacetone (867 μL, 8.49 mmol) were added sequentially and the solution was stirred at 120 °C for 24 h. The solution was concentrated under reduced pressure and then diluted with water. The precipitate was collected with suction filtration. The solids were washed with more water and Et_2_O to afford **29** (146 mg, 57%) as a beige solid. ^1^H NMR (400 MHz, DMSO-*d*_6_): δ (ppm) 10.08 (s, 1H), 8.18 (d, *J* = 2.4 Hz, 1H), 7.81 (dd, *J* = 8.9, 2.4 Hz, 1H), 7.66 (d, *J* = 9.0 Hz, 1H), 6.83 (s, 1H), 2.81 (s, 3H), 2.34 (s, 6H). ^13^C NMR (151 MHz, DMSO-*d*_6_): δ (ppm) 169.2, 167.2, 158.6, 155.6, 149.3, 134.2, 128.8, 128.2, 124.8, 121.1, 114.0, 23.5, 21.4. HRMS *m*/*z*: [M – H]^+^ calcd for C_15_H_15_ClN_5_^+^, 300.1010; found, 300.1008.

## Assays

### Folding of G4 Structures for FRET Study

Synthetic labeled oligonucleotides for the FRET study were purchased from Eurofins Genomics. Stock solutions were prepared in MQ-water at 100 μM concentration. The sequences used are listed in Supporting Information Table S1. All the oligonucleotides except Pu22 were prefolded in 10 mM lithium cacodylate buffer (pH 7.4), with 10 mM KCl and 90 mM LiCl by heating for 10 min at 95 °C and then cooling in fridge for at least 1 h. Pu22 was folded in 10 mM lithium cacodylate buffer (pH 7.4), with 2 mM KCl and 98 mM LiCl.

### FRET Melting assay

The FRET occurs between two dyes (5′-FAM as donor and 3′-TAMRA as acceptor) linked at both extremities of a DNA oligonucleotide. When the oligonucleotides are folded into G4 structures, the donor and acceptor are in proximity, which results in an energy transfer from the donor to the acceptor. This process can be detected by a reduction in the fluorescence emission of the donor. Fluorescence emission of the donor is recovered when the temperature increment triggers the thermal denaturation of the G4 structure. The experiments were performed in a Bio-Rad CFX96 real-time PCR device at temperatures from 10 to 95 °C at 1.5 °C/m heating rate using a 492 nm excitation wavelength and a 516 nm detection wavelength in 96-well plates. Each condition was tested in duplicate, and analysis of the data was carried out by using Excel and Origin 8 software. In each well, 0.2 μM of labeled oligonucleotide was heated in the presence or absence of the ligand (and with or without the competitor dsDNA) at the specified concentrations. Emission of 5′-FAM was normalized between 0 and 1, and the melting temperature (*T*_m_) is defined as the temperature at which 50% of the G4 structures are denatured (the temperature when the normalized emission was 0.5). The stabilization (Δ*T*_m_) is calculated from comparison of *T*_m_ of the fluorescently labeled oligonucleotide in the presence or absence of the ligand.

### Microscale Thermophoresis

5′-Cy5 labeled G4 DNAs for this study were purchased from Eurofins Genomics. Stock solutions were prepared in water at 100 μM concentration. The sequences used are listed in Supporting Information Table S1. The G4 DNA sequences were folded in KCl buffer (10 mM phosphate, 100 mM KCl, pH 7.4) by heating at 95 °C for 5 min and then cooling to room temperature. All the experiments were performed in 10 mM phosphate pH 7.4, 100 mM KCl, and 0.05% Tween20. The labeled DNA concentration is held constant at 25 nM, and ligand concentration is varied depending on ligands *K*_d_ (16 1:3 dilutions). The samples were loaded into standard MST graded glass capillaries and initial fluorescence intensity of the capillary was measured using Monolith NT.115 (Nano Temper, Germany) with 20% LED power. The change in fluorescence with ligand’s concentrations were plotted in Excel and fitted through nonlinear equation to obtain the binding constants.

### Compound Calculations

The calculations were performed in Maestro^41^ v. 11.9.011 for windows-64bit as a part of the Schrödinger package. The conformational searches for the compounds were conducted using MacroModel^42^ with the OPLS3e^43^ force field without solvent using a dielectric constant of 3. The Mixed Torsional/Low-Mode sampling (MTLMOD) was used, and maximum iterations were set to 5000, number of steps to 10,000, and RMSD cutoff to 0.5 Å. The ESP maps were generated using DFT geometry optimizations. The calculations were performed on the B3LYP-D3^44−46^ level of theory with the 6-31G** basis set as implemented in Jaguar.^47^ For compounds with aliphatic amines, the amine was protonated prior to any calculations.

### Isothermal Titration Calorimetry

ITC experiments were performed using a MicroCal ITC200 instrument (GE Healthcare). A buffer containing 10 mM potassium phosphate and 100 mM KCl of pH 7.4 was prepared. The sample cell was filled with a 20 μM ligand, and the syringe with 120 μM prefolded G4 DNA. The G4 DNA was titrated to the ligand during 20 injections (titrating the ligand to the G4 DNA did not give conclusive results). The following settings were applied: temperature 19 °C, reference power 7 μcal/s, initial delay 300 s, stirring speed 1000 rpm, spacing 120 s, filter 5 s, first injection 0.5 μL for 1 s then 2 μL for 4 s for the subsequent injections, and high-feedback mode. The data was analyzed in MICROCAL PEAQ-ITC analysis software using a one-site binding mode.

### Nuclear Magnetic Resonance Titrations

The G4 DNA stock solutions were prepared by folding 100 μM *c-MYC* Pu22 in 10 mM potassium phosphate buffer (pH = 7.4) and 35 mM KCl by heating at 95 °C for 10 min and cooling to ambient temperature overnight. 10% D_2_O was added to the DNA stock solutions, yielding a final DNA concentration of 90 μM. NMR samples were prepared by sequential addition of **5** or **27** from 2 or 10 mM DMSO-*d*_6_ stock solutions to 200 μL of the DNA solution which was then transferred to 3 mm NMR tubes. Control samples with Pu22 *c-MYC* G4 DNA with and without 10% DMSO-*d*_6_ was also performed to verify that DMSO did not have a significant effect on the DNA structure. All spectra were recorded at 298 K on a Bruker 850 MHz Avance III HD spectrometer equipped with a 5 mm TCI cryoprobe. Excitation sculpting was used in the 1D 1H experiments, and 256 scans were recorded. Processing of spectra was performed in MestreNova 10.0.2.

### Primer Extension Assay

Templates for the primer extension assay were created by annealing 25 nt TET labeled primer (1 μM final concentration) to either PEPu24T or a mutated sequence not forming a G4, PEPu24T mut, (1.25 μM final concentration) in 100 mM KCl by heating to 95 °C for 5 min and slowly cooling to room temperature. The reaction mixture (10 μL) contained 1× Taq buffer with KCl (10 mM Tris–HCl pH 8.8, 50 mM KCl, detergent), 25 mM MgCl_2_, 0.05 U Taq DNA polymerase, and 40 nM of template DNA. Compounds were added in the indicated concentration and incubated on ice for 10 min before starting the reaction by adding dNTPs (100 μM final concentration) to the reaction and transferring it to 37 °C. Reactions were stopped after 15 min by addition of 10 μL of formamide loading buffer (0.5% SDS, 25 mM EDTA, 95% v/v formamide and xylene-cyanol). Samples were then loaded on a 12% polyacrylamide gel containing 7 M urea and 25% formamide and imaged using an Amersham Typhoon. The images were quantified using ImageJ 1.53e software, and the percentage of full-length product compared to a sample without compound was calculated.

### Cell Viability

Cell viability was measured using the PrestoBlue cell viability reagent (Invitrogen) according to the manufacturer’s recommendations.

For HeLa cells, 5000 cells/well were seeded in complete medium on 96-wells, black walls plate the day before the treatment. Compounds were dissolved in medium at the indicated concentrations and added to cells. Five technical replicates were performed for each treatment. 48 h after treatment, 8 μL of PrestoBlue was added to each well and the cells were incubated at 37 °C for 15 min. Fluorescence (excitation 560 nm, emission 590 nm, 10 nm bandwidth) was recorded using a FLUOstar Omega Microplate Reader (BMG).

For IHTLCP, 1850 cells/well were seeded in complete Prigrow III medium (abm) in 384-wells, black walls plate (PerkinElmer) the day before the treatment. Compounds were titrated 1/2 in DMSO, dissolved in medium, and transferred to the cells to generate the indicated final concentrations (final volume 60 μL/well). 72 h after treatment, 6 μL of PrestoBlue was added per well. Cells were incubated in PrestoBlue for 1 h at 37 °C after which fluorescence (excitation 535 nm, emission 590 nm, 20 nm bandwidth) was measured using a Synergy H4Microplate Reader. Percent viability of IHTLCP cells was calculated as FLU_cmp_ – FLU_bg_/FLU_DMSO_ – FLU_bg_ (*n* = 3).

THP1 cell viability was measured according to a previously published procedure.^48^

### BG4 Immunostaining

The pSANG10-3F-BG4 construct, for expression and purification of recombinant BG4 (BG4), was a gift from Shankar Balasubramanian (Addgene plasmid # 55756). BG4 was purified as previously described.^49^

BG4 immunostaining was performed as previously described.^50^ Briefly, 60,000 HeLa cells were seeded on 13 mm glass coverslips the day before treatment. Cells were treated for 24 h with the compounds at the indicated concentrations. After treatment, cells were fixed in 2% paraformaldehyde and permeabilized in 0.1% Triton X-100 at room temperature. Cells were blocked in 2% nonfat milk followed by incubation with BG4–FLAG (1/3000), Rabbit anti-FLAG M2 (1/800, Sigma), and Goat anti Rabbit IgG Alexa Fluor594 (1/1000-Life Technologies) conjugated antibody. Each incubation was for 1 h at 37 °C in a humidified chamber. All washes and incubations were performed in 1× PBS buffer. Cell nuclei were stained with 0.2 μg/mL diamidino-2-phenylindole (DAPI) solution prior to mounting the coverslips on glass slides with DAKO mounting medium (Agilent Technologies). Cells were imaged with a LEICA SP8 FALCON confocal microscope equipped with a 63× oil objective (NA 1.40) using identical acquisition settings. Cell nuclei were focused on the DAPI channel, and BG4-positive foci were counted in a semiautomatic mode using a customized Cell Profiler (Broad Institute) pipeline. All images were processed using ImageJ software. Statistical analysis was performed as previously described.^50^

## Supporting Information Available

Experimental procedures and data; assay data, computational results, and all cell data; NMR (^1^H and ^13^C) spectra for all compounds (with the exception of a few intermediates); and HPLC UV traces for the final compounds (PDF)
Molecular formula strings and *K*_d_ values for binding to the Pu24T c-MYC G4 structure (CSV)

## Abbreviations

G-tetrad: also known as G-quartet, is one assembly in a G4 structure typically composed of 4 coordinated guanine bases
*c-MYC*: MYC proto-oncogene
*c-KIT*: c-KIT proto-oncogene
*KRAS*: Kirsten rat sarcoma virus gene
*BCL-2*: B-cell lymphoma 2 gene
ESP: electrostatic potential
MM: molecular mechanics
S_N_Ar: nucleophilic aromatic substitution
DMSO: dimethtylsulfoxide
DMF: dimethylformamide
PyAOP: ((7-azabenzotriazol-1-yloxy)tripyrrolidinophosphonium hexafluorophosphate)
FRET: Förster resonance energy transfer
MST: microscale thermophoresis
*K*_d_: dissociation constant or binding affinity
ITC: isothermal titration calorimetry
NMR: nuclear magnetic resonance
ppm: parts per million
SD: standard deviation
Caco-2: cancer coli
THP-1: human monocytic cell line
IHTLCP: (immortalized human total liver cell population)
BG4: monoclonal antibody that binds G4 structures
PDS: pyridostatin
