# Supplementary material for: Exploring the Dispersion and Electrostatic Components in Arene–Arene Interactions between Ligands and G4 DNA to Develop G4-Ligands
Source: J Med Chem. 2024 Jan 19;67(3):2202–19. doi: 10.1021/acs.jmedchem.3c02127 (PMC10860144; doi:10.1021/acs.jmedchem.3c02127)
Supplement: Supplementary file 1 — jm3c02127_si_001.pdf [file jm3c02127_si_001.pdf]

Supporting Information

Exploring the Dispersion and Electrostatic Components in Arene-Arene Interactions Between Ligands and G4 DNA to Develop G4-Ligands

Måns Andreasson[a], Maxime Donzel[a], Alva Abrahamsson[a], Andreas Berner[b], Mara Doimo[b, c], Anna Quiroga[b], Anna Eriksson[a], Yu-Kai Chao[d], Jeroen Overman[d], Nils Pemberton[e], Sjoerd Wanrooij[b], Erik Chorell*[a]

[a] Department of Chemistry, Umeå University, 901 87, Umeå, Sweden
[b] Departments of Medical Biochemistry and Biophysics, Umeå University, Umeå 90736, Sweden
[c] Clinical Genetics Unit, Department of Women and Children's Health, Padua University, 35128 Padua, Italy
[d] Mechanistic and Structural Biology, Discovery Sciences, R&D, AstraZeneca, Cambridge CB2 0AA, UK
[e] Medicinal Chemistry, Research and EarlyDevelopment, Respiratory and Immunology (R&I), BioPharmaceuticals R&D, AstraZeneca, Gothenburg SE-43183, Sweden

*Corresponding author: erik.chorell@umu.se

Table of Contents

Table S1. Oligonucleotides used in this study. 3

Folding of G4 structures for FRET study 4

FRET melting assay 4
- **Figure S1.** 4
- **Figure S2.** 5

Microscale Thermophoresis (MST) 5
- **Figure S3.** 6
- **Figure S4.** 6

Compound Calculations 7
- **Figure S5.** 7
- **Figure S6.** 7
- **Figure S7** 7
- **Figure S8.** 8
- **Figure S9.** 8
- **Figure S10.** 8
- **Figure S11.** 9
- **Figure S12.** 9
- **Figure S13.** 9
- **Figure S14.** 10
- **Figure S15.** 10
- **Figure S16.** 10
- **Figure S17.** 11
- **Figure S18.** 11
- **Figure S19.** 11

Isothermal titration calorimetry (ITC) **12**
- **Figure S20.** 12

Nuclear Magnetic Resonance (NMR) Titrations 12

Primer Extension Assay 13
- **Figure S21.** 13

Physicochemical properties 14
- **Table S2.** Pharmacokinetic Properties 14
- **Table S3.** 15

Cell Viability 15
- **Figure S22.** 16
- **Figure S23.** 16

BG4 Immunostaining 16
- **Figure S24.** 17

General Experimental 18

NMR spectra of Synthesized compounds 37

HPLC traces of tested compounds 81

References 85

Table S1. Oligonucleotides used in this study.

Sequence name	Sequence (5’-3’)	Technic	Function
FPu24TT	Fam-TGAG$_3$TG$_2$TGAG$_3$TG$_4$A$_2$G$_2$-Tamra	FRET melting	Labelled G4 (DNA)
FPu22T	Fam- TGAG$_3$TG$_3$TAG$_3$TG$_3$TA$_2$ -Tamra	FRET melting	Labelled G4 (DNA)
Fc-KIT2T	Fam-G$_3$CG$_3$CGCGAG$_3$AG$_4$-Tamra	FRET melting	Labelled G4 (DNA)
F25cebT	Fam- AG$_3$TG$_3$TGTAAGTGTG$_3$TG$_3$T –Tamra	FRET melting	Labelled G4 (DNA)
Fbom17T	Fam- G$_2$TTAG$_2$TTAG$_2$TTG$_2$ -Tamra	FRET melting	Labelled G4 (DNA)
FBcl2T	Fam-G$_3$CGCG$_3$AG$_2$AATTG$_3$CG$_3$-Tamra	FRET melting	Labelled G4 (DNA)
F21GT	Fam- G$_3$TTAG$_3$TTAG$_3$TTAG$_3$ -Tamra	FRET melting	Labelled G4 (DNA)
FtbaT	Fam- G$_2$TTG$_2$TGT G$_2$TTG-Tamra	FRET melting	Labelled G4 (DNA)
ds26	CAATCGGATCGAATTCGATCCGATTG	FRET melting	Competitor (DNA)
Pu22	TGAG$_3$TG$_3$TAG$_3$TG$_3$TA$_2$	NMR	G4 DNA
5’cy5-Pu24T	Cy5-TGAG$_3$TG$_2$TGAG$_3$TG$_4$A$_2$G$_2$	MST	Labelled G4 (DNA)
5’cy5-Pu22	Cy5-TGAG$_3$TG$_3$TAG$_3$TG$_3$TA$_2$	MST	Labelled G4 (DNA)
5’Cy5-ds26	Cy5-CAATCGGATCGAATTCGATCCGATTG	MST	Labelled non-G4 (DNA)

Folding of G4 structures for FRET study

Synthetic labelled oligonucleotides for FRET study were purchased from Eurofins Genomics. Stock solutions were prepared in MQ-water at 100 µM concentration. The sequences used are listed in supplementary Table S1. All the oligonucleotides except Pu22 were pre-folded in 10 mM lithium cacodylate buffer (pH 7.4), with 10 mM KCl and 90 mM LiCl by heating for 10 min at 95 °C and then cooling in fridge for at least 1 h. Pu22 was folded in 10 mM lithium cacodylate buffer (pH 7.4), with 2 mM KCl and 98 mM LiCl.

FRET melting assay

The fluorescence resonance energy transfer (FRET) occurs between two dyes (5'-FAM as donor and 3'- TAMRA as acceptor) linked at both extremities of a DNA oligonucleotide. When the oligonucleotides are folded into G4 structures, the donor and acceptor are in proximity, which results in an energy transfer from the donor to the acceptor. This process can be detected by a reduction in the fluorescence emission of the donor. Fluorescence emission of the donor is recovered when the temperature increment triggers the thermal denaturation of the G4 structure. The experiments were performed in a Bio-Rad CFX96 real-time PCR device at temperatures from 10 to 95 °C at 1.5 °C/m heating rate using a 492-nm excitation wavelength and a 516-nm detection wavelength in 96-well plates. Each condition was tested in duplicate, and analysis of the data was carried out by using Excel and Origin 8 software. In each well, 0.2 µM of labelled oligonucleotide was heated in the presence or absence of the ligand (and with or without the competitor dsDNA) at the specified concentrations. Emission of 5'-FAM was normalized between 0 and 1, and the melting temperature (T_m) is defined as the temperature at which 50% of the G4 structures are denatured (the temperature when the normalized emission was 0.5). The stabilization (ΔT_m) is calculated from comparison of T_m of the fluorescently labelled oligonucleotide in the presence or absence of the ligand.

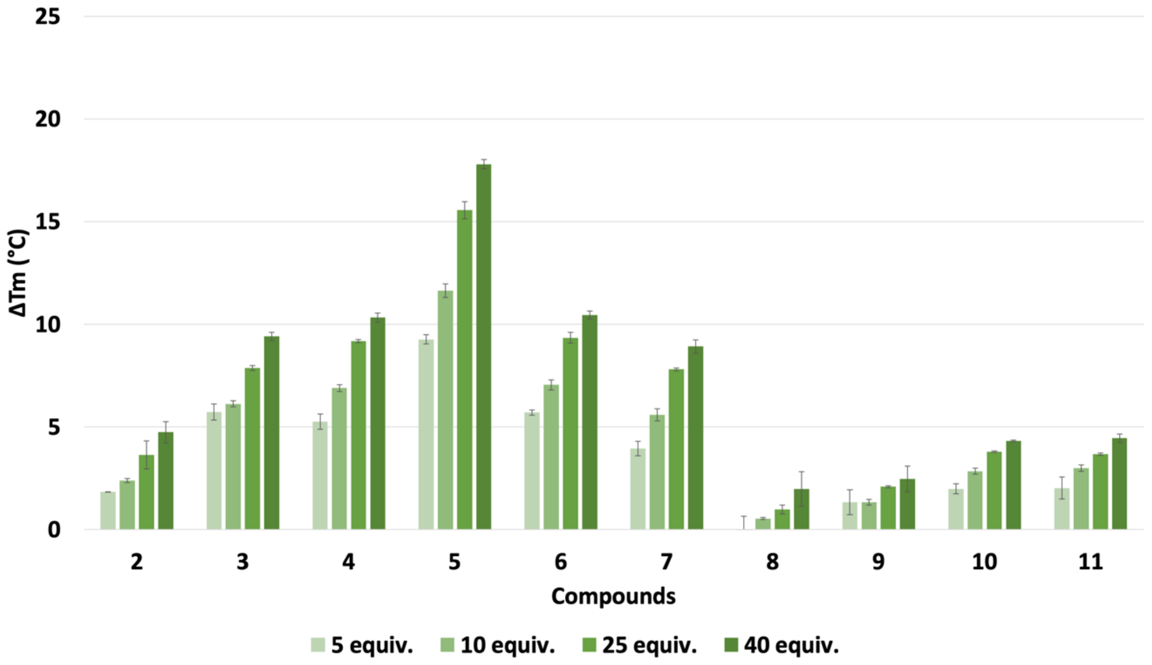

Figure S1. The FRET melting assay with the synthesized compounds (**2**-**11**) at 5, 10, 25, and 40 equivalents of added compound for Pu24T (0.2 µM), showing the ability of the compounds to affect the thermal stability of the G4 structure. Error bars correspond to the SD of six independent experiments.

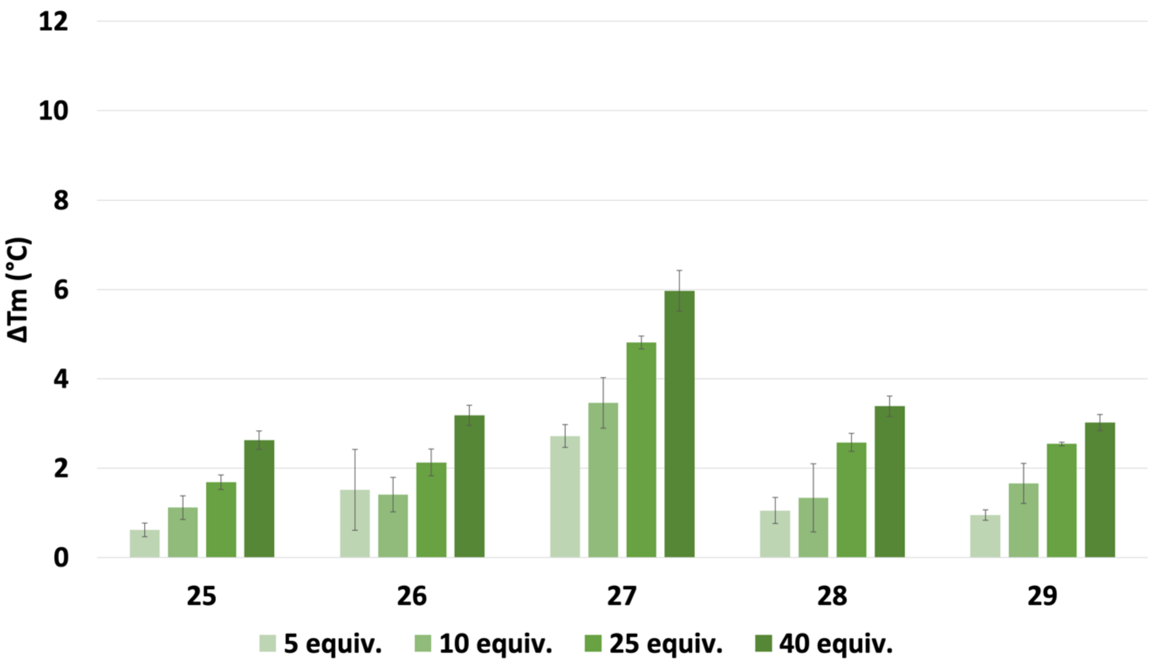

Figure S2. The FRET melting assay with the synthesized compounds (**25**-**29**) at 5, 10, 25, and 40 equivalents of added compound for Pu24T (0.2 µM), showing the ability of the compounds to affect the thermal stability of the G4 structure. Error bars correspond to the SD of six independent experiments.

Microscale Thermophoresis (MST)

5'- Cy5 labelled G4 DNAs for this study were purchased from Eurofins Genomics. Stock solutions were prepared in water at 100 mM concentration. The sequences used are listed in supplementary Table S1. The G4 DNA sequences were folded in KCl buffer (10 mM phosphate, 100 mM KCl, pH 7.4) by heating at 95 °C for 5 min and then cooling to room temperature. All the experiments were performed in 10 mM phosphate pH 7.4, 100 mM KCl, 0.05% Tween20. The labelled DNA concentration is held constant at 25 nM and ligand concentration is varied depending on ligands K_d (sixteen 1:3 dilutions). The samples were loaded into standard MST graded glass capillaries and initial fluorescence intensity of the capillary were measured using Monolith NT.115 (Nano Temper, Germany) with 20% LED power. The change in fluorescence with ligand's concentrations were plotted in Excel and fitted through non-linear equation to obtain the binding constants.

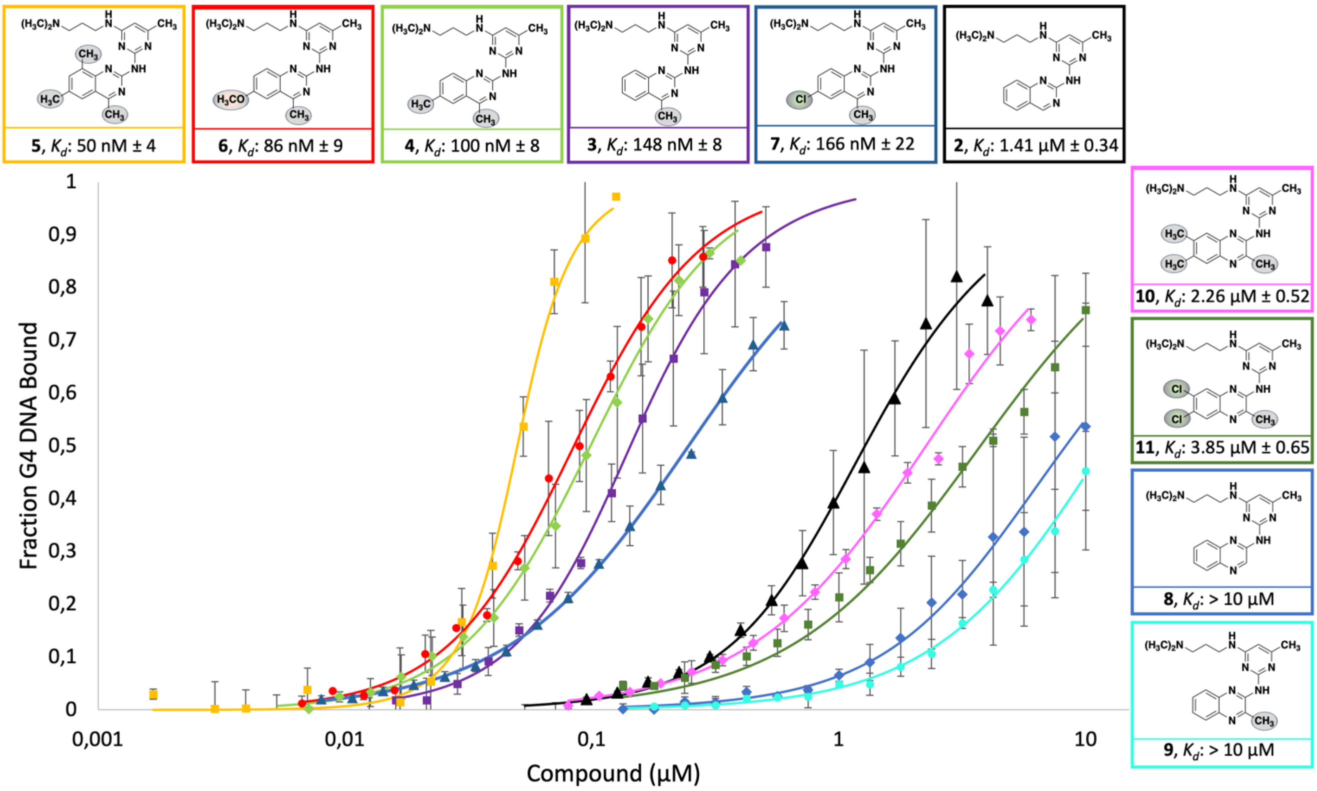

Figure S3. MST binding curves of each compound (**2**-**11**) at varying concentrations (dilution factor: 1:3) with Pu22 G4 DNA (0.025 mM). The error bars correspond to the SD of two independent experiments. Kd for each compound is written out in the graph and colour coded accordingly.

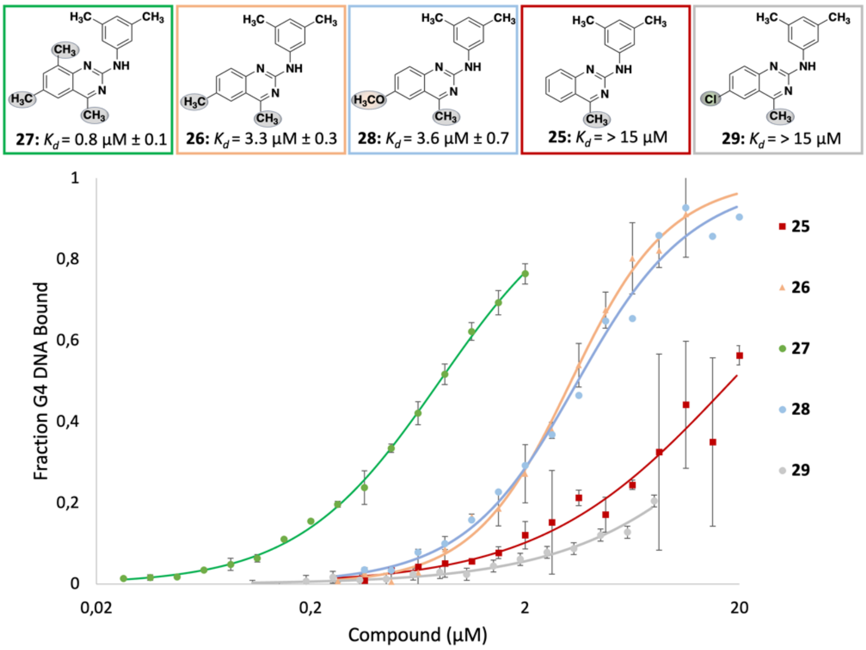

Figure S4. MST binding curves of each compound (**12**-**16**) at varying concentrations (dilution factor: 1:3) with *c-MYC* Pu22 G4 DNA (25 nM). The error bars correspond to the SD of two independent experiments. K_d for each compound is written out in the graph and color coded accordingly.

Compound Calculations

The calculations were performed in Maestro[1] v. 11.9.011 for windows-64bit as a part of the Schrödinger package. The conformational searches for the compounds were conducted using MacroModel[2] with the OPLS3e[3] force field without solvent using a dielectric constant of 3. The Mixed Torsional/Low-Mode sampling (MTLMOD) was used, and maximum iterations were set to 5 000, number of steps to 10 000, and RMSD cut-off to 0.5 Å. The ESP maps were generated using DFT geometry optimizations. The calculations were performed on the B3LYP-D3[4–6] level of theory with the 6-31G** basis set as implemented in Jaguar.[7] For compounds with aliphatic amines the amine was protonated prior to any calculations.

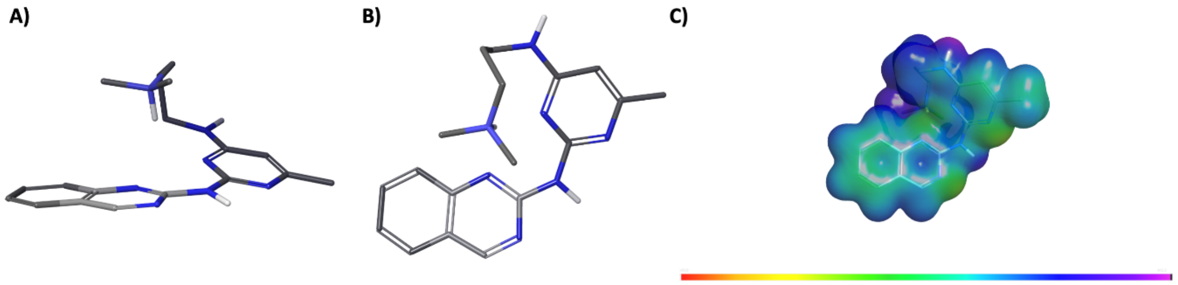

Figure S5. Lowest energy and most populated conformational state of **2** viewed from the **A)** side and **B)** top. **C)** ESP map of **2** shown with an ISO-value of 0.005 and an energy span of -40-140 kcal/mol. The color span represents different energy levels going from yellow/green (lowest negative, -40 kcal/mol) to purple (highest positive, 140 kcal/mol).

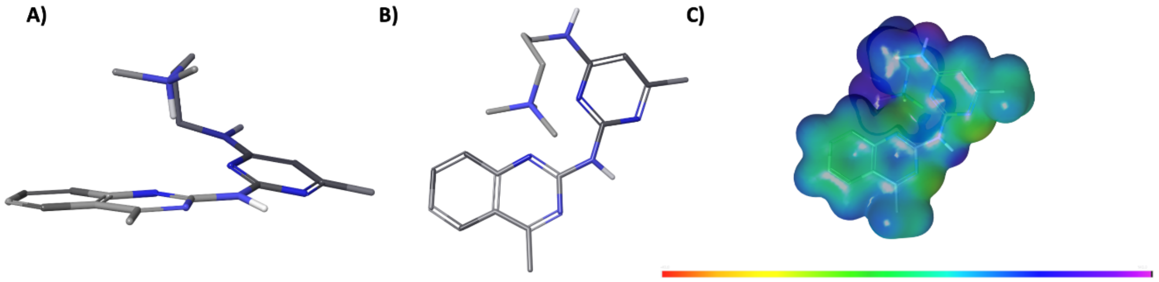

Figure S6. Lowest energy and most populated conformational state of **3** viewed from the **A)** side and **B)** top. **C)** ESP map of **3** shown with an ISO-value of 0.005 and an energy span of -40-140 kcal/mol. The color span represents different energy levels going from yellow/green (lowest negative, -40 kcal/mol) to purple (highest positive, 140 kcal/mol).

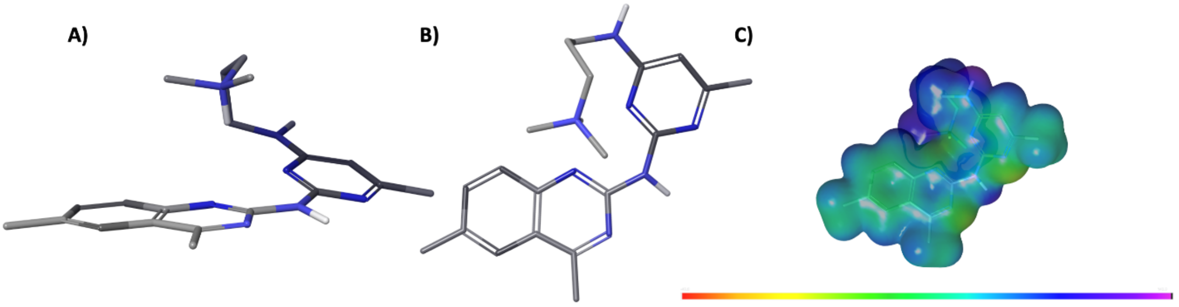

Figure S7 Lowest energy and most populated conformational state of **4** viewed from the **A)** side and **B)** top. **C)** ESP map of **4** shown with an ISO-value of 0.005 and an energy span of -40-140 kcal/mol. The color span represents different energy levels going from yellow/green (lowest negative, -40 kcal/mol) to purple (highest positive, 140 kcal/mol).

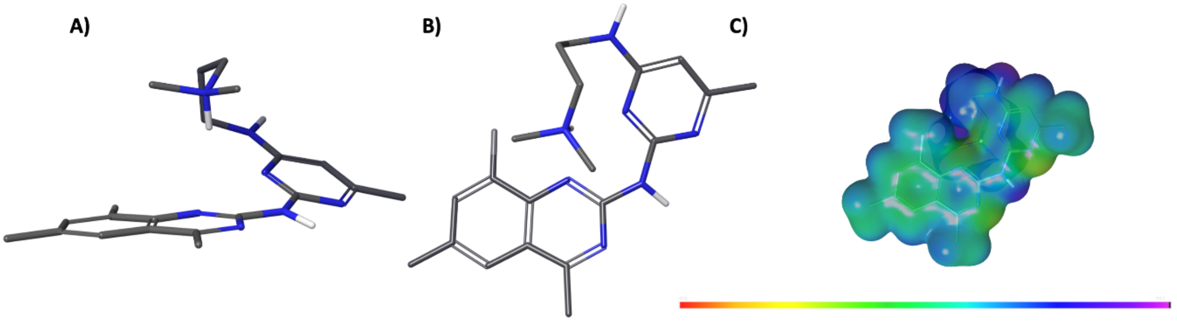

Figure S8. Lowest energy and most populated conformational state of **5** viewed from the **A)** side and **B)** top. **C)** ESP map of **5** shown with an ISO-value of 0.005 and an energy span of -40-140 kcal/mol. The color span represents different energy levels going from yellow/green (lowest negative, -40 kcal/mol) to purple (highest positive, 140 kcal/mol).

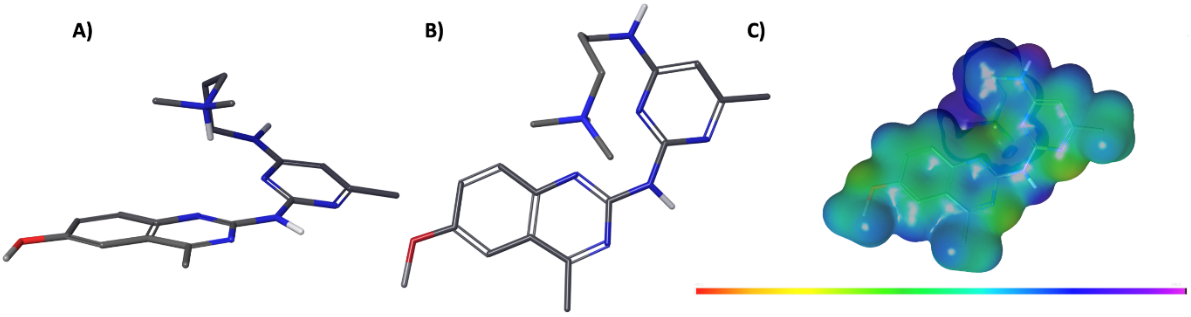

Figure S9. Lowest energy and most populated conformational state of **6** viewed from the **A)** side and **B)** top. **C)** ESP map of **6** shown with an ISO-value of 0.005 and an energy span of -40-140 kcal/mol. The color span represents different energy levels going from yellow/green (lowest negative, -40 kcal/mol) to purple (highest positive, 140 kcal/mol).

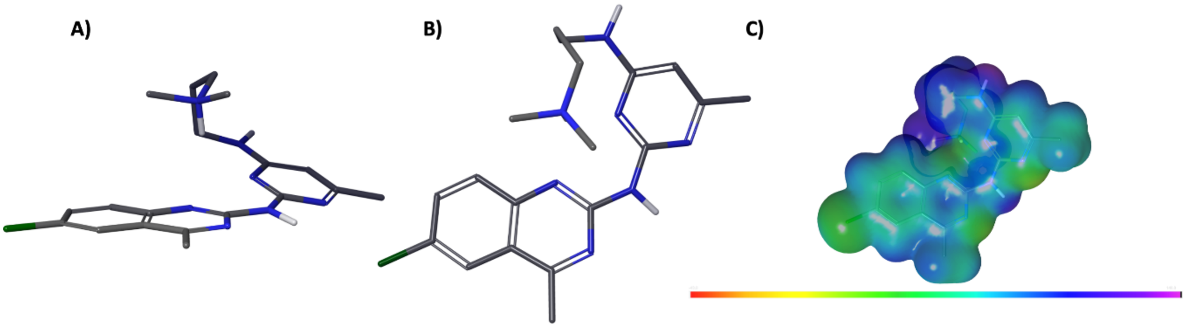

Figure S10. Lowest energy and most populated conformational state of **7** viewed from the **A)** side and **B)** top. **C)** ESP map of **7** shown with an ISO-value of 0.005 and an energy span of -40-140 kcal/mol. The color span represents different energy levels going from yellow/green (lowest negative, -40 kcal/mol) to purple (highest positive, 140 kcal/mol).

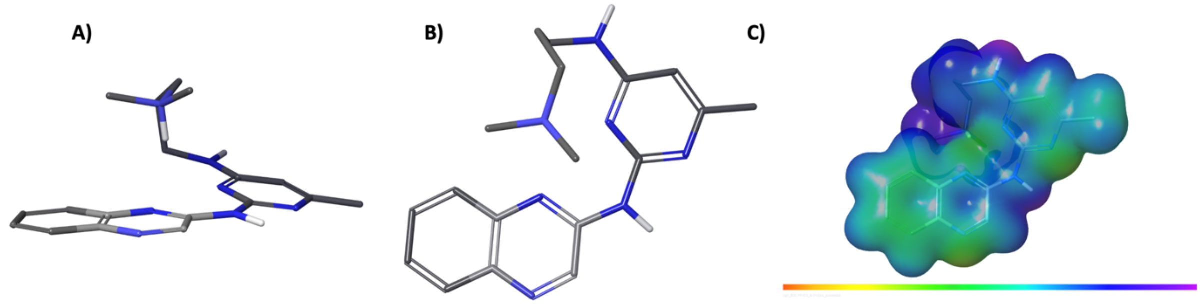

Figure S11. Lowest energy and most populated conformational state of **8** viewed from the **A)** side and **B)** top. **C)** ESP map of **8** shown with an ISO-value of 0.005 and an energy span of -40-140 kcal/mol. The color span represents different energy levels going from yellow/green (lowest negative, -40 kcal/mol) to purple (highest positive, 140 kcal/mol).

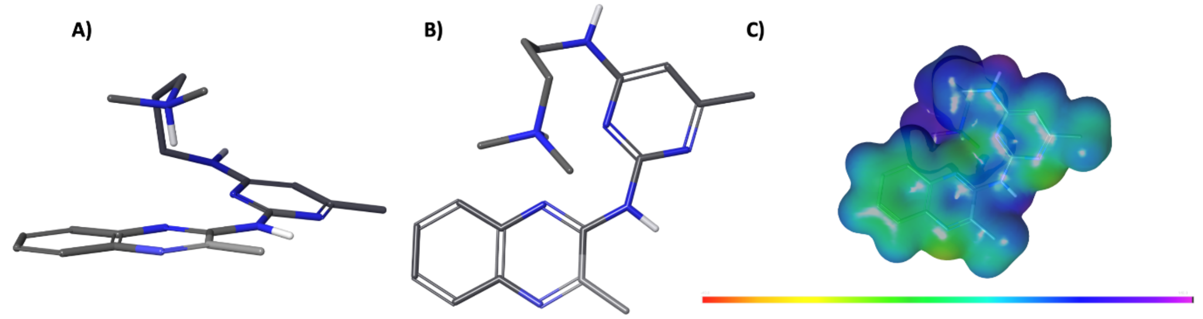

Figure S12. Lowest energy and most populated conformational state of **9** viewed from the **A)** side and **B)** top. **C)** ESP map of **9** shown with an ISO-value of 0.005 and an energy span of -40-140 kcal/mol. The color span represents different energy levels going from yellow/green (lowest negative, -40 kcal/mol) to purple (highest positive, 140 kcal/mol).

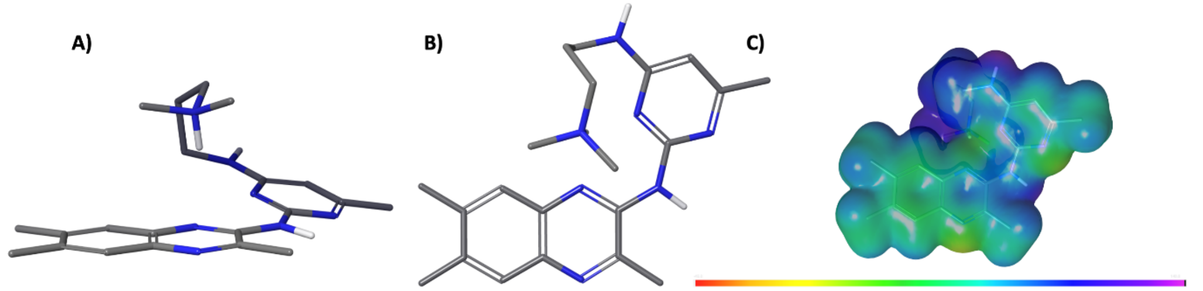

Figure S13. Lowest energy and most populated conformational state of **10** viewed from the **A)** side and **B)** top. **C)** ESP map of **10** shown with an ISO-value of 0.005 and an energy span of -40-140 kcal/mol. The color span represents different energy levels going from yellow/green (lowest negative, -40 kcal/mol) to purple (highest positive, 140 kcal/mol).

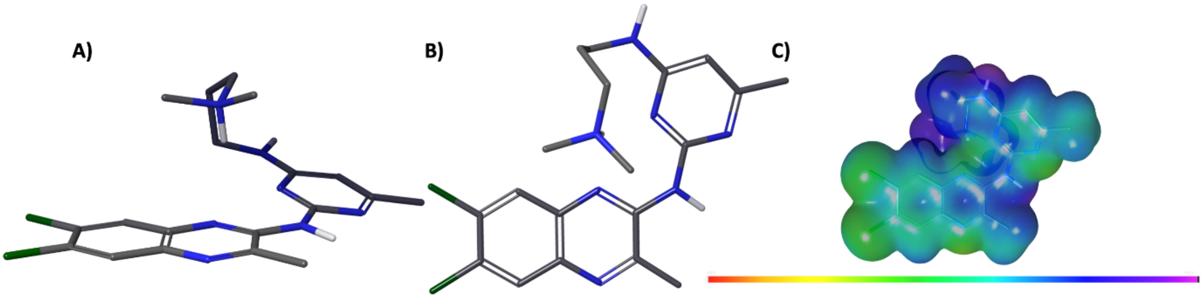

Figure S14. Lowest energy and most populated conformational state of **11** viewed from the **A)** side and **B)** top. **C)** ESP map of **11** shown with an ISO-value of 0.005 and an energy span of -40-140 kcal/mol. The color span represents different energy levels going from yellow/green (lowest negative, -40 kcal/mol) to purple (highest positive, 140 kcal/mol).

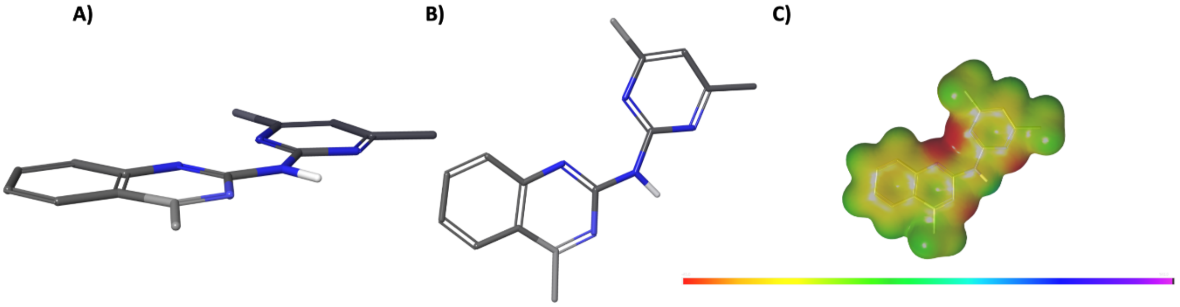

Figure S15. Lowest energy and most populated conformational state of **25** viewed from the **A)** side and **B)** top. **C)** ESP map of **25** shown with an ISO-value of 0.005 and an energy span of -40-140 kcal/mol. The color span represents different energy levels going from yellow/green (lowest negative, -40 kcal/mol) to purple (highest positive, 140 kcal/mol).

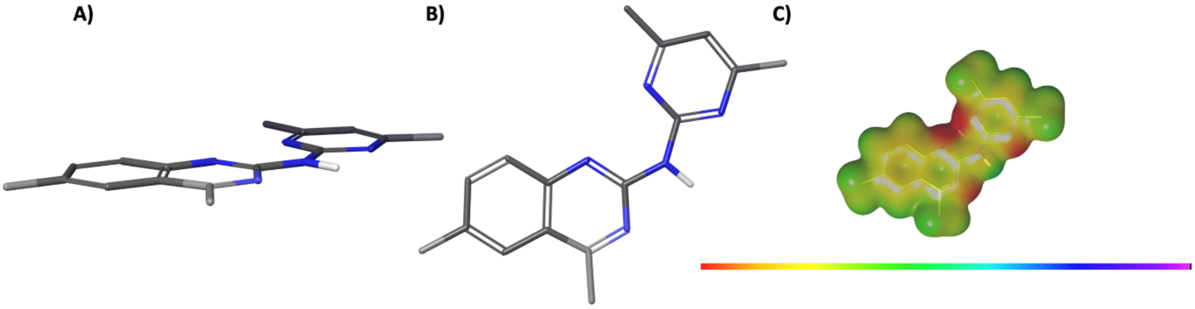

Figure S16. Lowest energy and most populated conformational state of **26** viewed from the **A)** side and **B)** top. **C)** ESP map of **26** shown with an ISO-value of 0.005 and an energy span of -40-140 kcal/mol. The color span represents different energy levels going from yellow/green (lowest negative, -40 kcal/mol) to purple (highest positive, 140 kcal/mol).

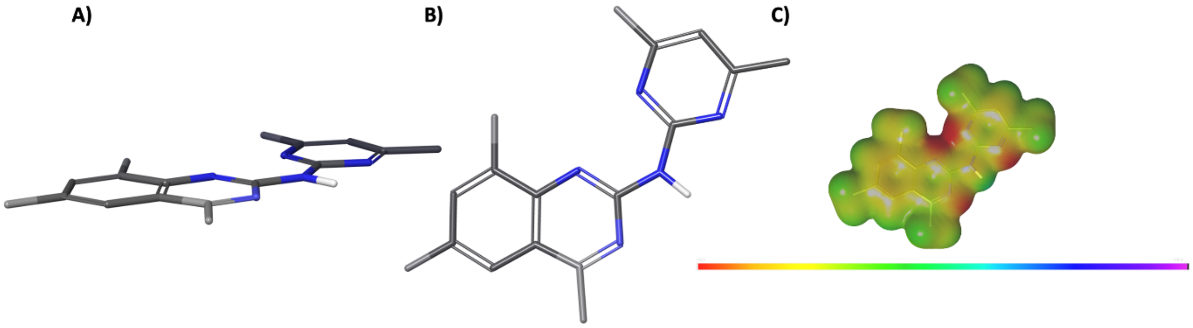

Figure S17. Lowest energy and most populated conformational state of **27** viewed from the **A)** side and **B)** top. **C)** ESP map of **27** shown with an ISO-value of 0.005 and an energy span of -40-140 kcal/mol. The color span represents different energy levels going from yellow/green (lowest negative, -40 kcal/mol) to purple (highest positive, 140 kcal/mol).

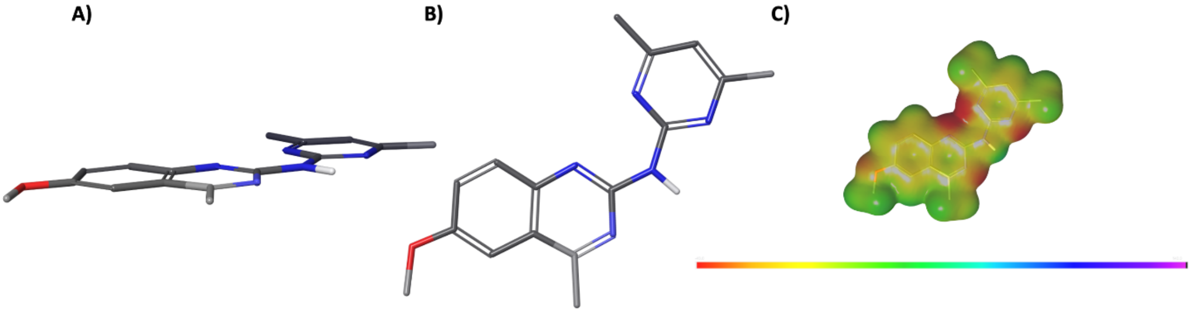

Figure S18. Lowest energy and most populated conformational state of **28** viewed from the **A)** side and **B)** top. **C)** ESP map of **28** shown with an ISO-value of 0.005 and an energy span of -40-140 kcal/mol. The color span represents different energy levels going from yellow/green (lowest negative, -40 kcal/mol) to purple (highest positive, 140 kcal/mol).

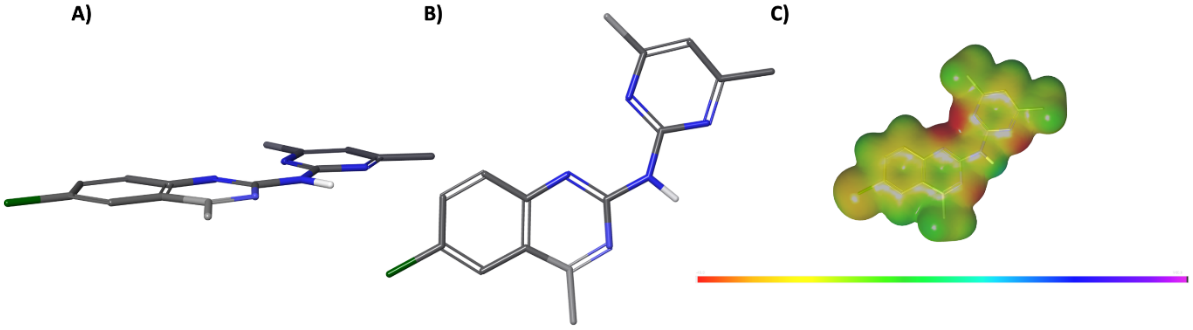

Figure S19. Lowest energy and most populated conformational state of **29** viewed from the **A)** side and **B)** top. **C)** ESP map of **29** shown with an ISO-value of 0.005 and an energy span of -40-140 kcal/mol. The color span represents different energy levels going from yellow/green (lowest negative, -40 kcal/mol) to purple (highest positive, 140 kcal/mol).

Isothermal titration calorimetry (ITC)

ITC experiments were performed using a MicroCal ITC200 instrument (GE Healthcare). A buffer containing 10 mM potassium phosphate and 100 mM KCl of pH 7.4 was prepared. The sample cell was filled with 20 µM ligand and the syringe with 120 µM prefolded G4 DNA. The G4 DNA was titrated to the ligand during 20 injections (titrating the ligand to the G4 DNA did not give conclusive results). The following settings were applied: temperature 19 °C, reference power 7 µcal/sec, initial delay 300 sec, stirring speed 1000 RPM, spacing 120 sec, filter 5 sec, first injection 0.5 µL for 1 sec then 2 µL for 4 sec for the subsequent injections, and high-feedback mode. The data was analyzed in MICROCAL PEAQ-ITC analysis software using a one-site binding mode.

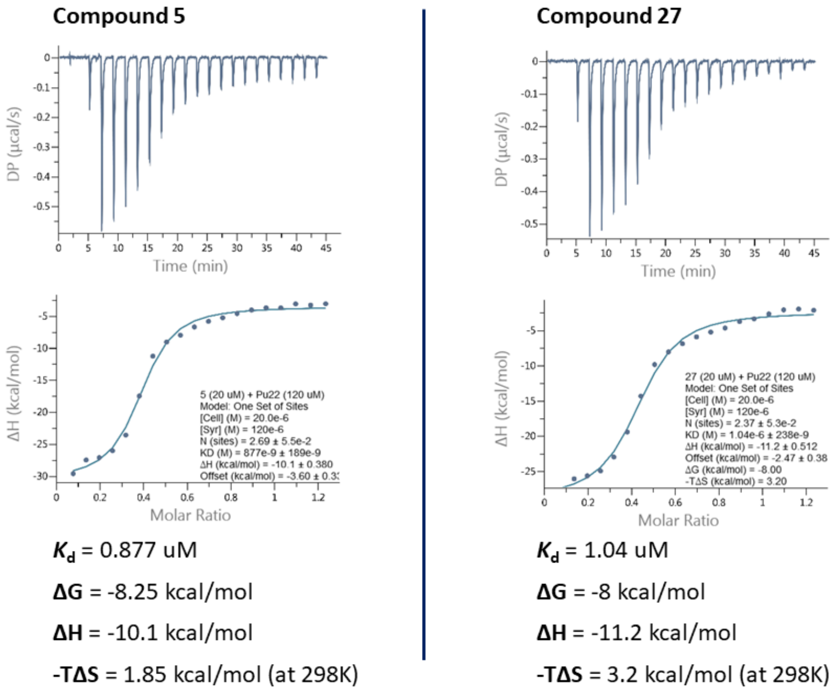

Figure S20. Binding curves with their associated ΔG-, ΔH-, and -TΔS-values for compounds **5** and **27** for G4 DNA (Pu22) measured by ITC with a one-site binding model.

Nuclear Magnetic Resonance (NMR) Titrations

The G4 DNA stock solutions were prepared by folding 100 µM *c-MYC* Pu22 in 10 mM potassium phosphate buffer (pH = 7.4) and 35 mM KCl by heating at 95 °C for 10 min and cooling to ambient temperature overnight. 10% D_2O was added to the DNA stock solutions, yielding a final DNA concentration of 90 µM. NMR samples were prepared by sequential addition of **5** or **27** from 2 or 10 mM DMSO-d_6 stock solutions to 200 µL of the DNA solution which was then transferred to 3 mm NMR tubes. Control samples with Pu22 *c-MYC* G4 DNA with and without 10% DMSO-d_6 was also performed to verify that DMSO did not have a significant effect on the DNA structure. All spectra were recorded at 298 K on a Bruker 850 MHz Avance III HD spectrometer equipped with a 5 mm TCI cryoprobe. Excitation sculpting was used in the 1D 1H experiments, and 256 scans were recorded. Processing of spectra was performed in MestreNova 10.0.2.

Primer Extension Assay

Templates for the primer extension assay were created by annealing 25nt TET labelled primer (1 µM final concentration) to either PEPu24T or a mutated sequence not forming a G4, PEPu24T mut, (1.25 µM final concentration) in 100 mM KCl by heating to 95 °C for 5 min and slowly cooling to room temperature. The reaction mixture (10 µl) contained 1X Taq Buffer with KCl (10 mM Tris-HCl pH 8.8, 50 mM KCl, detergent), 25 mM $MgCl_2$, 0.05 U Taq DNA Polymerase and 40 nM of template DNA. Compounds were added in the indicated concentration and incubated on ice for 10 min before starting the reaction by adding dNTPs (100 µM final concentration) to the reaction and transferring it to 37 °C. Reactions were stopped after 15 min by addition of 10 µl of formamide loading buffer (0,5% SDS, 25 mM EDTA, 95% v/v formamide and xylene-cyanol). Samples were then loaded on a 12% polyacrylamide gel containing 7 M urea and 25% formamide and imaged using an Amersham™ Typhoon™. The images were quantified using ImageJ 1.53e software and the percentage of full-length product compared to a sample without compound was calculated.

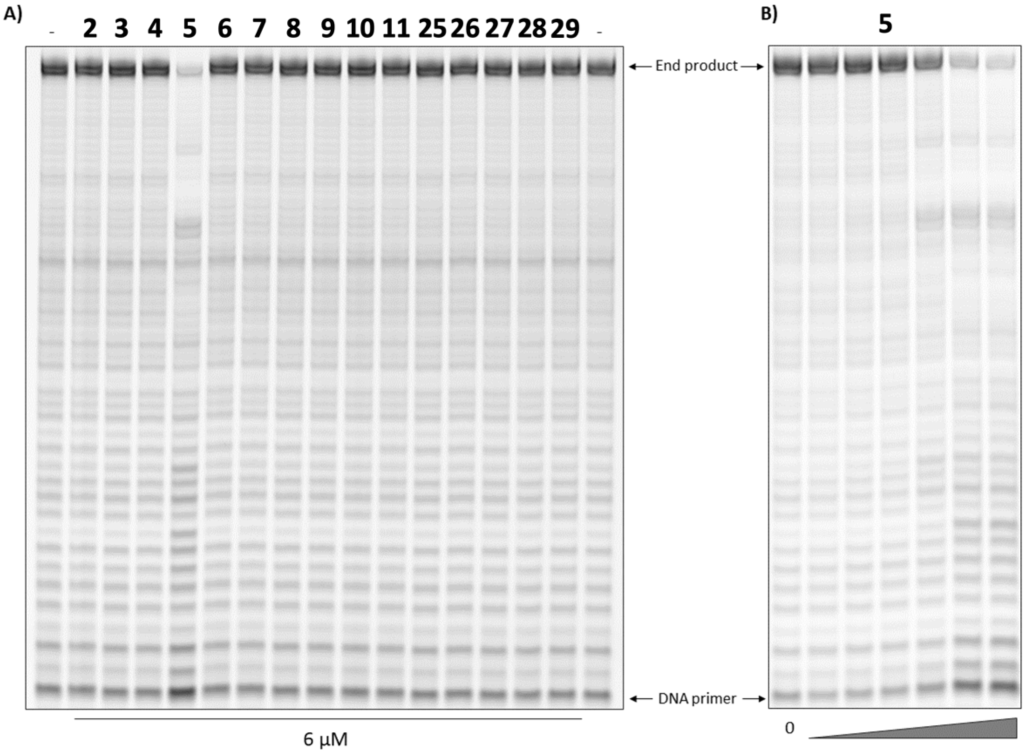

Figure S21. Primer extension assay on a template not forming a G4 structure. **A)** No compound (-) or 6 µM of compounds (**2-11, 25-29**) was added to the reaction. **B)** increasing concentration (0, 1, 2, 3, 4, 5, 6 µM) of compound **5** were added to the reaction. DNA primer and full-length product are indicated.

Physicochemical properties

The Physicochemical profiling (LogD, Solubility, Caco-2 cell permeability) was performed as previously described.[8] Cell viability was performed as described below.

Table S2. Pharmacokinetic Properties

Compound	Structure	logD, pH 7.4	Solubility, pH 7.4 (μM)	Rat heps[a]	Human mics [b]
**2**	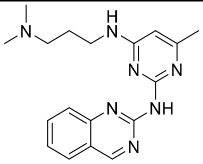	-0.1	>1000	8	<3
**3**	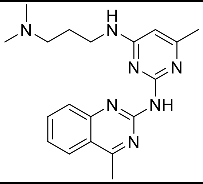	0	858	4	<3
**4**	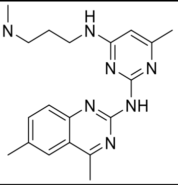	0.4	>1000	<1	<3
**5**	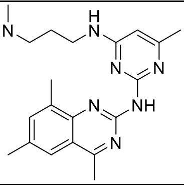	0.6	962	1	<3
**6**	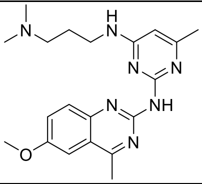	0.1	>1000	<1	<3
**7**	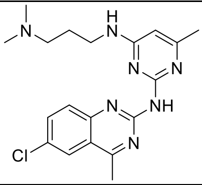	0.9	990	1	<3
**8**	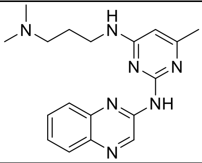	1.4	>1000	38	8
**9**	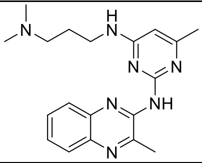	0.5	871	17	<3
**10**	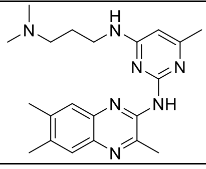	1.4	838	19	<3

**11**	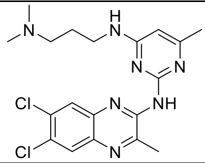	2.3	646	6	<3
**25**	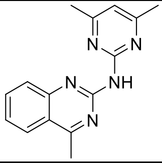	1.8	>1000	17	71
**26**	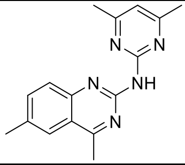	2.2	951	20	84
**27**	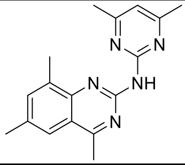	2.9	264	27	140
**28**	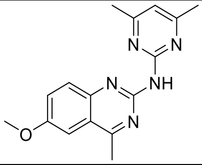	1.9	271	34	22
**29**	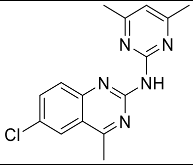	2.4	134	9	56

[a] Intrinsic clearance in rat hepatocytes ((µL/min)/10^6 cells).[b] Intrinsic clearance in human microsomes (µL/min/mg).

Table S3. Cell permeability and cell viability for **5** and **27**

Compound	Cell-permeability ($X \times 10^{-6}$ cm/s)	Cell viability IC_{50} (THP1) (µM)	Cell viability IC_{50} (HeLa) (µM)	Cell viability IC_{50} (IHTLCP) (µM)
**5**	<0.13	2.89	0.88-1.1	0.64
**27**	70	>50	19-34	35

Cell Viability

Cell viability was measured using the PrestoBlue cell viability reagent (Invitrogen) according to the manufacturer's recommendations.

For HeLa cells, 5000 cells/well were seeded in complete medium on 96-wells, black walls plate the day before the treatment. Compounds were dissolved in medium at the indicated concentrations and added to cells. Five technical replicates were performed for each treatment. 48 h after treatment, 8 µL of PrestoBlue was added to each well and the cells were incubated at 37 °C for 15 minutes. Fluorescence (excitation 560 nm, emission 590 nm, 10 nm bandwidth) was recorded using a FLUOstar Omega Microplate Reader (BMG).

For Immortalized Human Total Liver Cell Population (IHTLCP), 1850 cells/well were seeded in complete Prigrow III medium (abm) in 384-wells, black walls plate (Perkin Elmer) the day before the treatment. Compounds were titrated 1:2 in DMSO, dissolved in medium and transferred to the cells to generate the indicated final concentrations (final volume 60 µL/well). 72 h after treatment, 6 µL of PrestoBlue was added per well. Cells were incubated in PrestoBlue for 1h at 37 °C after which fluorescence (excitation 535 nm, emission 590 nm, 20 nm bandwidth) was measured using a Synergy H4 Microplate Reader. Percent viability of IHTLCP cells was calculated as FLU_{cmp} -FLU_{bg}/ FLU_{DMSO} -FLU_{bg} (n=3).

THP1 cell viability was measured according to a previously published procedure.[9]

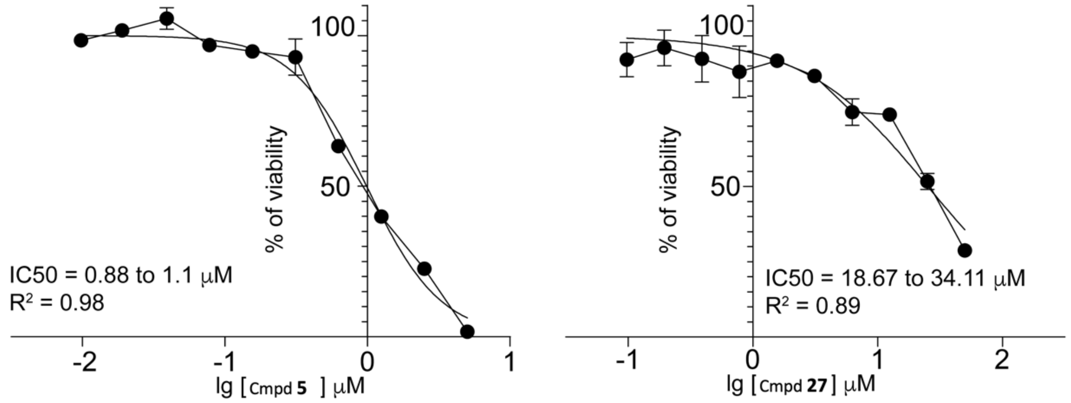

Figure S22. Cell viability assay of HeLa cells treated for 48h with A) **5** or B) **27** at the indicated concentrations. Data represent the mean of two biological replicates ± absolute error.

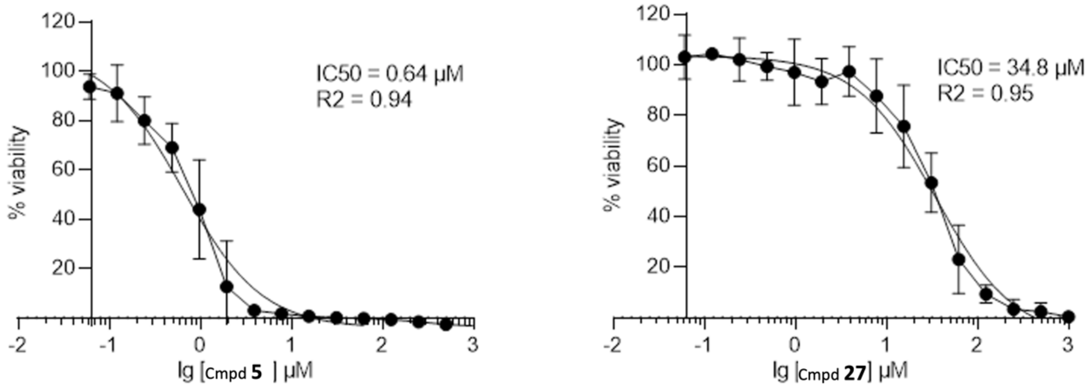

Figure S23. Toxicity of **5** and **27** calculated as % viability of IHTLCP cells treated for 72h at the indicated concentrations compared to non-treated cells. Data represent mean of three independent experiments ± stdev (n=3). IC50 and R2 determined with Graph Pad Prism is indicated.

BG4 Immunostaining

pSANG10-3F-BG4 construct, for expression and purification of recombinant BG4 (BG4), was a gift from Shankar Balasubramanian (Addgene plasmid # 55756). BG4 was purified as previously described.[10]
BG4 immunostaining was performed as previously described.[11] Briefly, 60.000 HeLa cells were seeded on 13 mm glass coverslips the day before treatment. Cells were treated for 24h with the compounds at the indicated concentrations. After treatment, cells were fixed in 2% paraformaldehyde and permeabilized in 0.1% Triton X-100 at room temperature. Cells were blocked in 2% non-fat milk followed by incubation with BG4-FLAG (1:3000), Rabbit anti-FLAG M2 (1:800, Sigma), and Goat anti Rabbit IgG Alexa Fluor594 (1:1000- Life Technologies) conjugated antibody. Each incubation was for 1 h at 37 °C in a humidified chamber. All washes and incubations were performed in 1× PBS buffer. Cell nuclei were stained with 0.2 μg/mL diamidino-2-phenylindole (DAPI) solution prior to mounting the coverslips on glass slides with DAKO mounting medium (Agilent Technologies). Cells were imaged with a LEICA SP8 FALCON confocal microscope equipped with a 63× oil objective (NA 1.40) using identical acquisition settings. Cell nuclei were focused on the DAPI channel, and BG4-positive foci were counted in a semiautomatic mode using a customized Cell Profiler (Broad Institute) pipeline. All images were processed using ImageJ software. Statistical analysis was performed as previously described.[11]

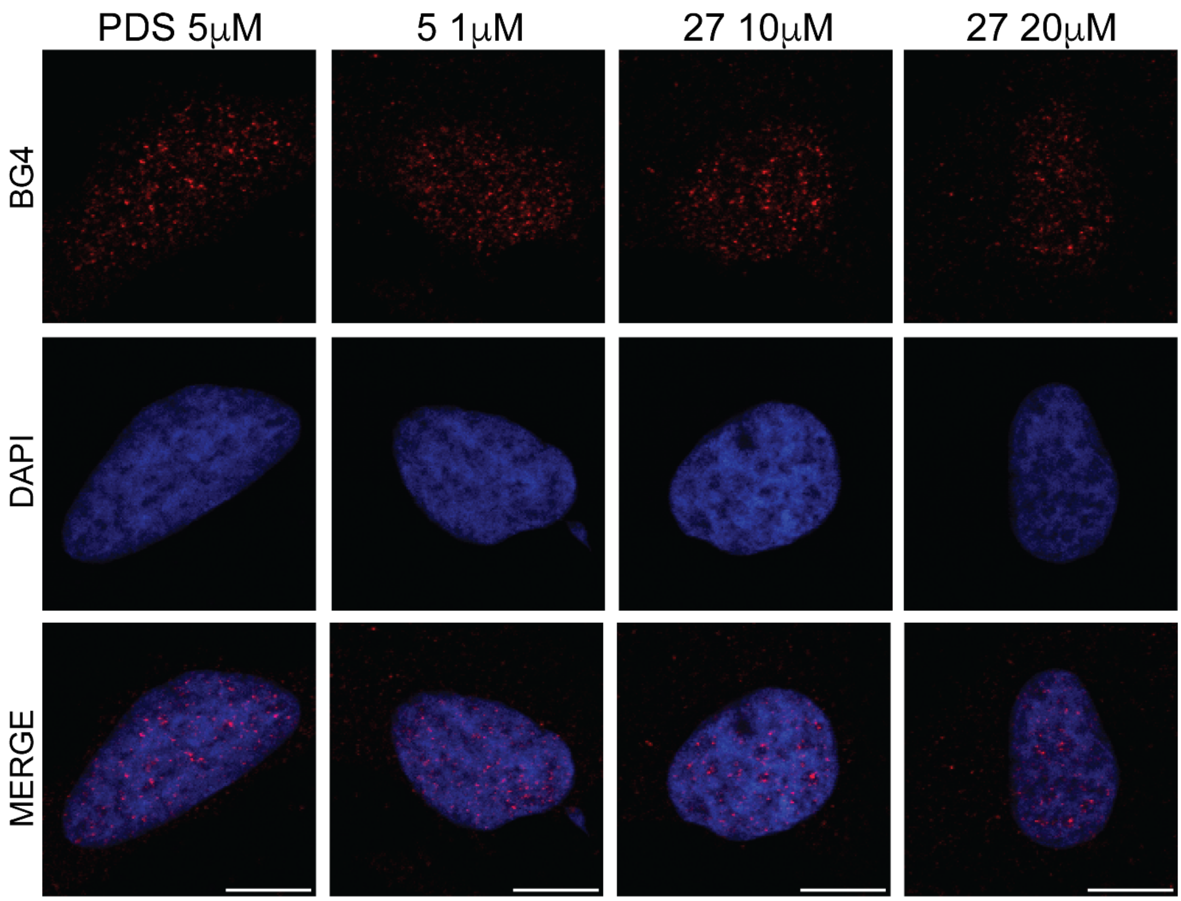

Figure S24. Representative images of HeLa cells stained with the BG4 antibody after treatment for 24 h. Scale bar = 10 μM. See Figure 8B for quantification.

General Experimental

All reagents and solvents were used as received from commercial suppliers unless stated otherwise. TLC was performed on aluminum backed silica gel plates (median pore size 60 Å, fluorescent indicator 254 nm) and detected with UV light. Flash column chromatography was performed using silica gel with an average particle diameter of 50 µm (range 40−65 µm, pore diameter 53 Å), eluents are given in brackets. ^{1}H and ^{13}C NMR spectra were recorded on a Bruker 400 MHz or 600 MHz spectrometer at 298 K, calibrated by using the residual peak of the solvents as the internal standard ($CDCl_3$: δ (ppm) H = 7.26; δ (ppm) C = 77.16. DMSO-d_6: δ (ppm) H = 2.50; δ (ppm) C = 39.50. *There is always one carbon which is not visible (or merged with another carbon) in the ^{13}C NMR for compounds (**2-11**), even if more than 4000 scans with concentrated samples were measured at 150 MHz*. All tested compounds showed a purity of ≥95% determined by the UV chromatogram by LC-MS analysis. LC-MS was performed on an Agilent 6150 Series Quadrupole LC/MS system. Microwave reactions were carried out in an Initiator + microwave instrument from Biotage, using sealed 0.2–0.5 mL, 2-5 mL, or 10-20 mL process vials. Reaction times refer to irradiation time at the target temperature, not the total irradiation time. The temperature was measured with an IR sensor.

1-(quinazolin-2-yl)guanidine (12).

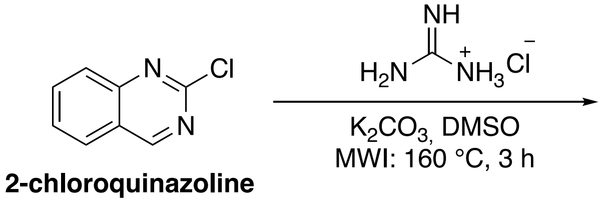

A microwave vial (2-5 mL) was charged with 2-chloroquinazoline (200 mg, 1.22 mmol), guanidine hydrochloride (232 mg, 2.43 mmol), and K_2CO_3 (504 mg, 3.65 mmol). Then, DMSO (4 mL) was added, and the mixture was reacted using MWI (160 °C, 3 h). The mixture was then cooled to ambient temperature. Water was added and the mixture was sonicated and then cooled over ice. The solids were then collected with suction filtration and washed with small portions off water and Et_2O to afford **12** (118 mg, 52%) as a yellow solid. ^{1}H NMR (400 MHz, DMSO-d_6) δ (ppm) 9.41 (s, 1H), 7.99 (dd, J = 8.1, 1.3 Hz, 1H), 7.88 (ddd, J = 8.5, 6.8, 1.3 Hz, 1H), 7.80 (dd, J = 8.5, 1.3 Hz, 1H), 7.50 (ddd, J = 8.1, 6.8, 1.3 Hz, 1H). ^{13}C NMR (151 MHz, DMSO-d_6) δ (ppm) 162.7, 158.7, 157.4, 149.3, 134.7, 127.8, 125.6, 124.9, 120.8.

6-methyl-2-(quinazolin-2-ylamino)pyrimidin-4(3H)-one (13).

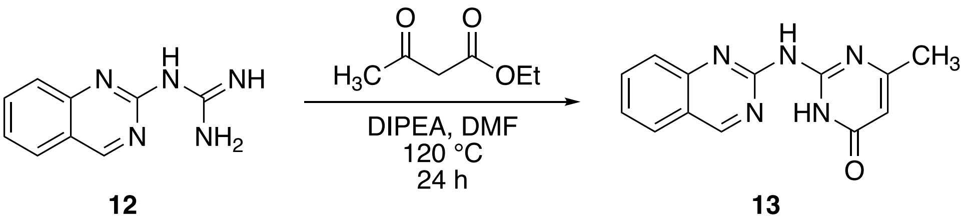

12 13

A vial was charged with **12** (117 mg, 0.625 mmol)**.** Then, DMF (3 mL), DIPEA (218 mL, 1.25 mmol), and ethyl acetoacetate (797 mL, 6.25 mmol) were added sequentially, and the solution was stirred at 120 °C for 24 h. The mixture was then diluted with Et_2O, and the precipitate was collected with suction filtration and the solids were washed with more Et_2O. Purification by flash column chromatography (eluent: 3% → 3.5% MeOH in CH_2Cl_2) afforded **13** (64 mg, 40%) as a pale-brown solid. ^{1}H NMR (400 MHz, DMSO-d_6) δ (ppm) 13.24 (s, 1H, NH), 11.51 (s, 1H, NH), 9.53 (s, 1H), 8.09 (d, J = 8.1 Hz, 1H), 7.98 (t, J = 7.7 Hz, 1H), 7.79 (d, J = 8.6 Hz, 1H), 7.60 (t, J = 7.5 Hz, 1H), 5.84 (s, 1H), 2.17 (s, 3H, CH_3). ^{13}C NMR (100 MHz, DMSO-d_6) δ (ppm) 163.6, 161.1, 155.6, 151.4, 149.2, 148.5, 135.8, 128.4, 125.9, 125.3, 121.2, 104.7, 23.5.

N4-(3-(dimethylamino)propyl)-6-methyl-N2-(quinazolin-2-yl)pyrimidine-2,4-diamine (2).

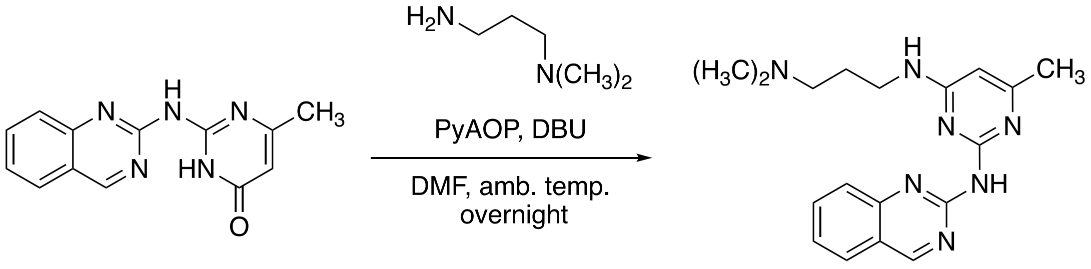

A vial was charged with **13** (60 mg, 0.24 mmol) and PyAOP (161 mg, 0.309 mmol). Then, DMF (1.5 mL) and DBU (53 mL, 0.35 mmol) were added, and the resulting solution was stirred at ambient temperature for 1 h. Propylamine (75 mL, 0.60 mmol) was then added, and the solution was stirred overnight. The reaction solution was then concentrated under reduced pressure and diluted with water. The AQ layer was then extracted with $CHCl_3$:IPA (3:1, 15 mL × 3). The combined org. layer was then dried over Na_2SO_4, filtered, and concentrated under reduced pressure. Purification by flash column chromatography (eluent: 15% → 20% MeOH (1% NH_4OH) in CH_2Cl_2) afforded **2** (18 mg, 23%) as a dark-yellow solid. ^{1}H NMR (400 MHz, DMSO-d_6) δ (ppm) 9.37 (s, 1H), 7.98 (d, J = 8.1 Hz, 1H), 7.86 (ddd, J = 8.5, 6.9, 1.5 Hz, 1H), 7.71 (d, J = 8.5 Hz, 1H), 7.48 (t, J = 7.5 Hz, 1H), 7.37 (s, 1H) 6.01 (s, 1H), 3.29 (s, 2H), 2.72 – 2.60 (m, 2H), 2.37 (s, 6H), 2.18 (s, 3H), 1.84 (app. dt, J = 12.2, 7.5 Hz, 2H). ^{13}C NMR (151 MHz, DMSO-d_6) δ (ppm) 163.4, 162.0, 157.9, 156.2, 150.7, 134.4, 127.8, 126.0, 124.5, 121.1, 98.3, 55.6, 47.6, 43.7, 25.7, 23.2. HRMS: m/z: $[M-H]^+$ calcd for $C_{18}H_{24}N_7^+$ 338.2099; Found 338.2108.

2,2,4-trimethyl-1,2-dihydroquinoline (14a).

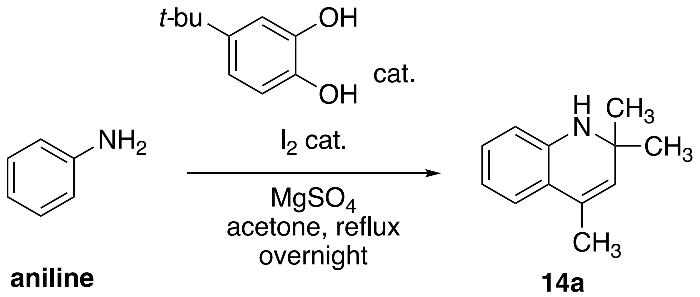

A round-bottom flask (RBF) was charged with $MgSO_4$ (6.46 g, 53.7 mmol), catechol 53.6 mg, 0.322 mmol), and I_2 (136 mg, 0.537 mmol). Then, aniline (979 mL, 10.7 mmol) and acetone (20 mL) were added, and the mixture was stirred at reflux overnight. After cooling down to ambient temperature, the mixture was filtered over a plug of celite with EtOAc and the resulting org. solution was concentrated under reduced pressure. Purification by flash column chromatography (eluent: 5% EtOAc in n-heptane) afforded **14a** (1.15 g, 62%) as an orange oil. ^{1}H NMR (400 MHz, $CDCl_3$) δ (ppm) 7.06 (dd, J = 7.6, 1.5 Hz, 1H), 6.98 (td, J = 7.6, 1.5 Hz, 1H), 6.64 (td, J = 7.5, 1.2 Hz, 1H), 6.46 (dd, J = 7.9, 1.2 Hz, 1H), 5.31 (d, J = 1.5 Hz, 1H), 3.87 (s, 1H), 1.99 (d, J = 1.4 Hz, 3H), 1.28 (s, 6H). ^{13}C NMR (100 MHz, $CDCl_3$), δ (ppm) 143.2, 128.6, 128.4, 128.4, 123.6, 121.6, 117.2, 113.0, 51.8, 31.0, 18.6.

2,2,4,6-tetramethyl-1,2-dihydroquinoline (14b).

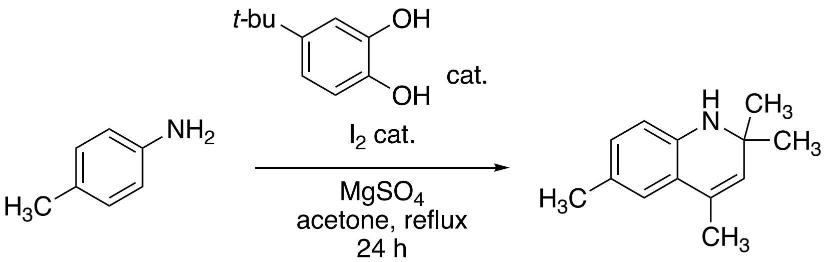

4-methylaniline **14b**

A RBF (100 mL) was charged with $MgSO_4$ (11.2 g, 93.3 mmol), catechol (93 mg, 0.56 mmol), and I_2 (236 mg, 0.930 mmol). Then, 4-methylaniline (2.00 g, 18.7 mmol) and acetone (50 mL) were added, and the mixture was refluxed for 24 h. After cooling down the mixture was filtered over a plug of celite with EtOAc and the resulting org. solution was concentrated under reduced pressure. Purification by flash column chromatography (eluent: 2% EtOAc in n-heptane) afforded **14b** (2.11 g, 60%) as a pink oil. ^{1}H NMR (400 MHz, $CDCl_3$) δ (ppm) 6.88 (d, J = 2.0 Hz, 1H), 6.84 – 6.77 (m, 1H), 6.38 (d, J = 7.9 Hz, 1H), 5.32 (d, J = 1.7 Hz, 1H), 3.56 (s, 1H, NH), 2.23 (s, 3H), 1.99 (d, J = 1.7 Hz, 3H), 1.26 (s, 6H, $(CH_3)_2$). ^{13}C NMR (100 MHz, $CDCl_3$) δ (ppm) 141.1, 128.9, 128.8, 128.7, 126.3, 124.3, 121.8, 113.1, 51.8, 30.9, 20.8, 18.8.

2,2,4,6,8-pentamethyl-1,2-dihydroquinoline (14c).

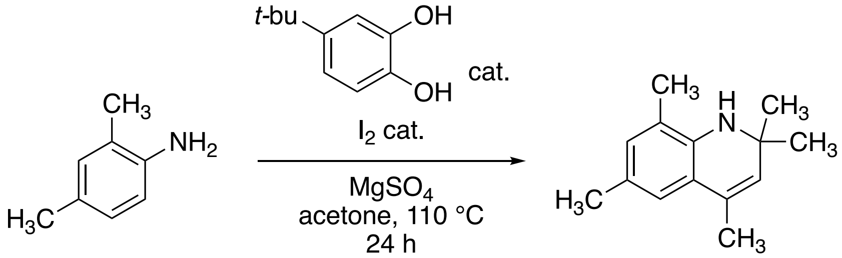

2,4-dimethylaniline **14c**

The reaction was carried out in two batches. A m-wave vial (10-20 mL) were charged with $MgSO_4$ (5.00 g, 41.5 mmol), catechol (41 mg, 0.25 mmol), and I_2 (105 mg, 0.412 mmol) each. Then, 2,4-dimethylaniline (1.02 mL, 8.25 mmol) and acetone (20 mL) were added, and the mixture was heated at 110 °C in the sealed tube for 24 h. After cooling down the mixture was filtered over a plug of celite with EtOAc and the resulting org. solution was concentrated under reduced pressure. Purification by flash column chromatography (eluent: 2.5% EtOAc in n-heptane) afforded the **14c** (1.43 g, 43%) as a brown oil. ^{1}H NMR (400 MHz, $CDCl_3$) δ (ppm) 6.81 (d, J = 2.2 Hz, 1H), 6.75 (d, J = 2.2 Hz, 1H), 5.32 (d, J = 1.5 Hz, 1H), 3.46 (s, 1H, NH), 2.22 (s, 3H), 2.08 (s, 3H), 2.00 (d, J = 1.5 Hz, 3H), 1.28 (s, 6H, $(CH_3)_2$). ^{13}C NMR (100 MHz, $CDCl_3$) δ (ppm) 139.0, 130.5, 129.0, 128.3, 125.4, 122.3, 121.2, 119.9, 51.8, 31.2, 20.8, 19.1, 17.0.

6-methoxy-2,2,4-trimethyl-1,2-dihydroquinoline (14d).

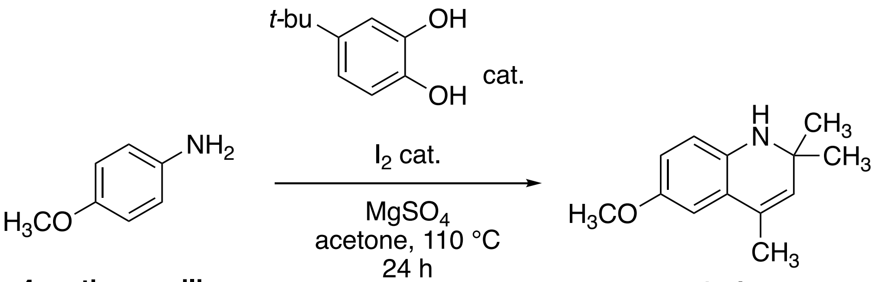

4-methoxyaniline **14d**

A RBF (100 mL) was charged with $MgSO_4$ (9.80 g, 81.2 mmol), catechol (81 mg, 0.49 mmol), and I_2 (206 mg, 0.812 mmol). Then, 4-methoxyaniline (2.00 g, 16.2 mmol) and acetone (50 mL) were added, and the mixture was refluxed for 24 h. After cooling down the mixture was filtered over a plug of celite with EtOAc and the resulting org. solution was concentrated under reduced pressure. Purification by flash column chromatography (eluent: 3% EtOAc in n-heptane) afforded **14d** (2.34 g, 71%) as a brown oil. ^{1}H NMR (400 MHz, $CDCl_3$) δ (ppm) 6.69 (s, 1H), 6.61 (dd, J = 8.4, 2.8 Hz, 1H), 6.41 (s, 1H), 5.37 (s, 1H), 3.75 (s, 3H), 3.47 (s, 1H), 1.98 (s, 3H), 1.25 (s, 6H). ^{13}C NMR (100 MHz, $CDCl_3$) δ (ppm) 152.0, 137.5, 129.8, 128.5, 123.0, 113.7, 113.5, 110.1, 55.9, 51.7, 30.4, 18.6.

6-chloro-2,2,4-trimethyl-1,2-dihydroquinoline (14e).

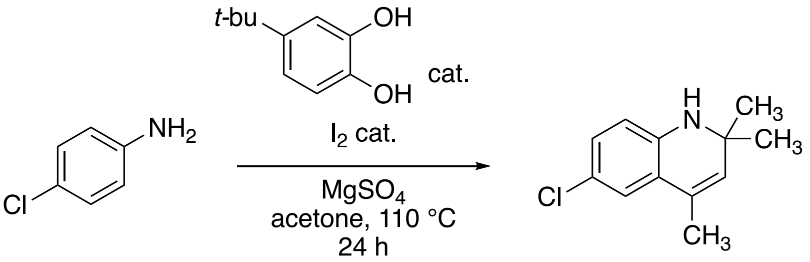

4-chloroaniline **14e**

A m-wave vial was charged with $MgSO_4$ (6.02 g, 50.0 mmol), catechol (50 mg, 0.30 mmol), and I_2 (127 mg, 0.500 mmol). Then, 4-chloroaniline (1.3 g, 10 mmol) and acetone (20 mL) were added, the tube was sealed, and the mixture was stirred at 110 °C 24 h. After cooling down was the mixture was filtered over a plug of celite with EtOAc and the resulting org. solution was concentrated under reduced pressure. Purification by flash column chromatography (eluent: 2% EtOAc in n-heptane) afforded **14e** (1.3 g, 63%) product as a yellow oil. ^{1}H NMR (400 MHz, $CDCl_3$) δ (ppm) 7.00 (d, J = 2.3 Hz, 1H), 6.92 (dd, J = 8.4, 2.4 Hz, 1H), 6.36 (d, J = 8.4 Hz, 1H), 5.35 (s, 1H), 3.67 (s, 1H), 1.96 (d, J = 1.5 Hz, 3H), 1.27 (s, 6H). ^{13}C NMR (151 MHz, $CDCl_3$) δ (ppm) 141.7, 129.6, 128.0, 127.9, 123.6, 123.2, 122.0, 114.1, 52.2, 31.0, 18.6.

1-(4-methylquinazolin-2-yl)guanidine (15a).

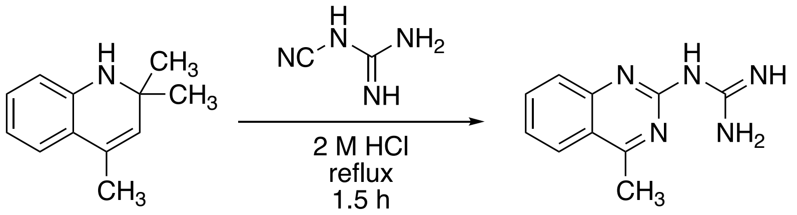

14a **15a**

To a vial containing **14a** (1.11 g, 6.43 mmol) was added 2-cyanoguanidine (1.08 g, 12.8 mmol) followed by HCl (2 M, 3.54 mL), and the mixture was refluxed for 1.5 h. Then, the mixture was allowed to cool to ambient temperature, and NaOH (15%, 2.06 mL) was added. The formed precipitate was collected with suction filtration and washed with water. The solid was sonicated 30 min in CH_2Cl_2 and the filtered again to afford **15a** (1.18 g, 91%) as a pale-beige solid. ^{1}H NMR (400 MHz, Chloroform-*d*) δ (ppm) 8.07 (dd, J = 8.3, 1.3 Hz, 1H), 7.79 (ddd, J = 8.3, 6.8, 1.4 Hz, 1H), 7.69 (d, J = 7.8 Hz, 1H), 7.41 (ddd, J = 8.2, 6.8, 1.2 Hz, 1H), 2.79 (s, 3H). ^{13}C NMR (100 MHz, DMSO-*d6*) δ (ppm) 169.6, 161.5, 159.3, 150.1, 134.0, 126.3, 126.0, 124.0, 119.9, 22.0.

1-(4,6-dimethylquinazolin-2-yl)guanidine (15b).

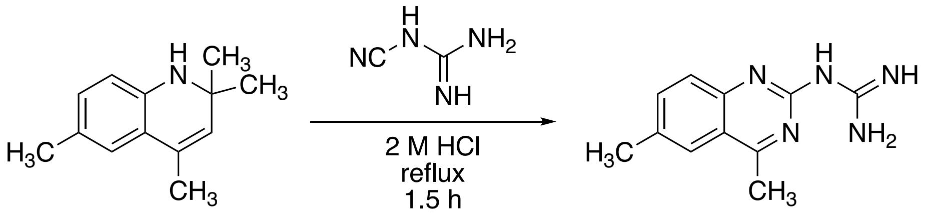

A RBF (50 mL) containing **14b** (2.10 g, 11.2 mmol) was charged with 2-cyanoguanidine (1.90 g, 22.6 mmol). Then, HCl (2 M, 6.2 mL) was added, and the mixture was stirred at 110 °C for 1.5 h. The mixture was then allowed to cool to ambient temperature and basified with NaOH (15%, 3.7 mL) and diluted with more water. The mixture was then sonicated, and the formed precipitate was then collected with suction filtration and washed with more water and Et_2O to afford **15b** (2.16 g, 90%) as a white solid. ^1H NMR (400 MHz, DMSO-d_6) δ (ppm) 7.93 (s, 1H, H), 7.72 (s, 2H), 2.82 (s, 3H), 2.49 (s, 3H). *No carbon could be obtained due to poor solubility.*

1-(4,6,8-trimethylquinazolin-2-yl)guanidine (15c).

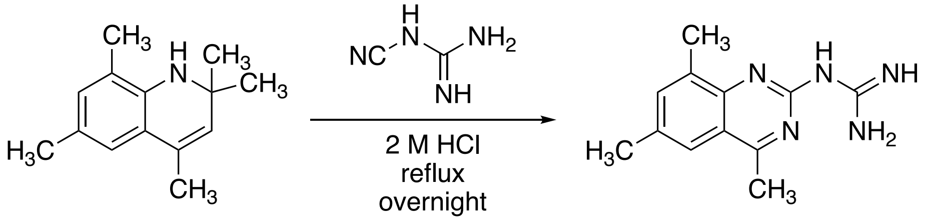

A RBF (25 mL) containing **14c** (1.43 g, 7.10 mmol) was charged with 2-cyanoguanidine (1.20 g, 14.3 mmol). Then, HCl (2 M, 3.9 mL) was added, and the mixture was stirred at 110 °C overnight. The mixture was then allowed to cool to ambient temperature and basified with NaOH (15%, 2.3 mL) and diluted with more water. The mixture was then sonicated, and the formed precipitate was then collected with suction filtration and washed with more water and Et_2O to afford **15c** (1.36 g, 84%) as a white solid. ^1H NMR (400 MHz, DMSO-d_6) δ (ppm) 7.60 (s, 1H), 7.43 (s, 1H), 7.13 (s, 4H, NH), 2.68 (s, 3H), 2.45 (s, 3H), 2.39 (s, 3H). *No carbon could be obtained due to poor solubility.*

1-(6-methoxy-4-methylquinazolin-2-yl)guanidine (15d).

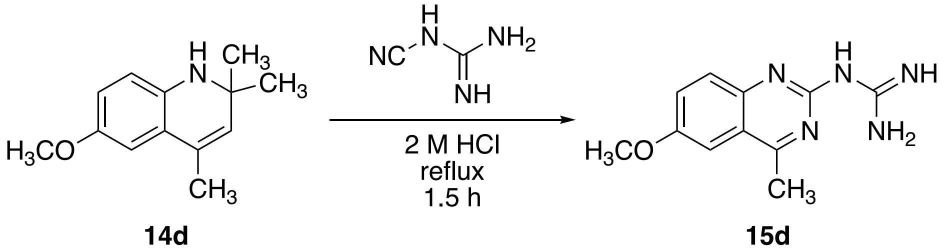

A vial containing **14d** (1.145 g, 5.63 mmol) was charged with 2-cyanoguanidine (947 mg, 11.27 mmol). Then, HCl (2 M, 3.1 mL) was added, and the mixture was stirred at 110 °C for overnight. The mixture was then allowed to cool to ambient temperature and basified with NaOH (15%, 1.8 mL) and diluted with more water. The mixture was then sonicated, and the formed precipitate was then collected with suction filtration and washed with more water and Et_2O to afford **15d** (1.22 g, 94%) as a white solid. ^1H NMR (400 MHz, DMSO-d_6) δ (ppm) 7.55 (d, J = 9.1 Hz, 1H), 7.39 (dd, J = 9.1, 2.8 Hz, 1H), 7.30 (d, J = 2.8 Hz, 1H), 3.87 (s, 3H), 2.72 (s, 3H). ^{13}C NMR (100 MHz, DMSO-*d6*), δ (ppm) 170.8, 157.5, 155.7, 152.2, 144.5, 128.7, 127.7, 122.3, 104.7, 56.3, 22.2.

1-(6-chloro-4-methylquinazolin-2-yl)guanidine (15e).

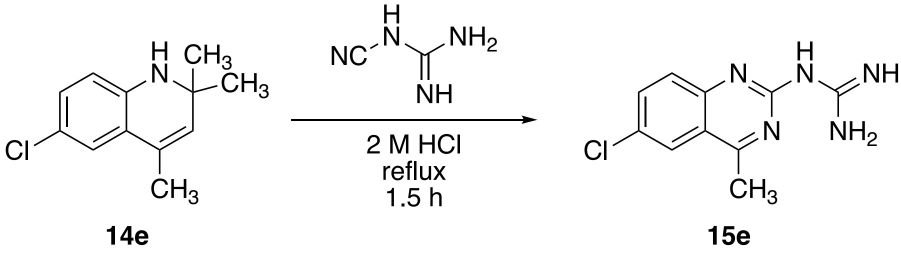

A vial containing **14e** (1.3 g, 6.3 mmol) was charged with 2-cyanoguanidine (1.05 g, 12.5 mmol). Then, HCl (2 M, 3.44 mL) was added, and the mixture was stirred at 110 °C for 1.5 h. The mixture was then allowed to cool to ambient temperature and basified with NaOH (15%, 2 mL) and diluted with more water. The mixture was then sonicated, and the formed precipitate was then collected with suction filtration and washed with more water and Et_2O to afford **15e** (780 mg, 53%) as a white solid. ^1H NMR (600 MHz, DMSO-d_6) δ (ppm) 8.20 (d, J = 2.3 Hz, 1H), 7.87 (dd, J = 8.9, 2.3 Hz, 1H), 7.81 (d, J = 8.9 Hz, 1H), 2.82 (s, 3H). ^{13}C NMR (100 MHz, DMSO-d_6) δ (ppm) 168.4, 162.0, 159.4, 148.5, 133.7, 127.9, 126.9, 124.5, 119.8, 21.6.

6-methyl-2-((4-methylquinazolin-2-yl)amino)pyrimidin-4(3H)-one (16a).

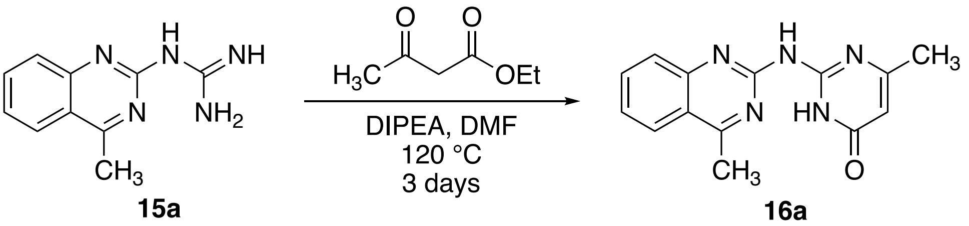

To a vial containing **15a** (1.18 g, 5.84 mmol) was added DMF (5 mL) ethyl acetoacetate (7.45 mL, 58.4 mmol) and DIPEA (2.03 mL, 11.7 mmol), the vial was sealed, and the mixture was stirred at 120 °C for 3 days. The mixture was then diluted with Et_2O, and the precipitate was collected with suction filtration and washed with more Et_2O. **16a** (1 g, 65%) was then collected as a beige solid. ^1H NMR (400 MHz, DMSO-d_6) δ (ppm) 13.38 (s, 1H), 11.33 (s, 1H), 8.23 (dd, J = 8.3, 1.4 Hz, 1H), 7.95 (ddd, J = 8.4, 6.9, 1.4 Hz, 1H), 7.76 (d, J = 8.3 Hz, 1H), 7.58 (ddd, J = 8.2, 6.9, 1.2 Hz, 1H), 5.83 (s, 1H), 2.90 (s, 3H), 2.16 (s, 3H). *No carbon could be obtained due to poor solubility.*

2-((4,6-dimethylquinazolin-2-yl)amino)-6-methylpyrimidin-4(3H)-one (16b).

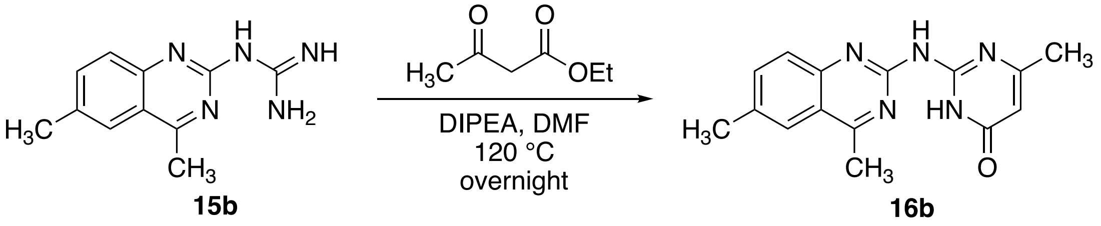

A vial (2-5 mL) was charged with **15b (**250 mg, 1.16 mmol). Then, DMF (2 mL), DIPEA (405 mL, 2.33 mmol), and ethyl acetoacetate (1.48 mL, 11.6 mmol) were added sequentially, and the solution was stirred at 120 °C overnight. The mixture was then diluted with Et_2O and sonicated. The precipitate was collected with suction filtration and washed with more Et_2O to afford **16b** (240 mg, 74%) as a pale beige solid. ^1H NMR (400 MHz, DMSO-d_6) δ (ppm) 13.33 (s, 1H, NH), 11.21 (s, 1H, NH), 7.96 (d, J = 2.2 Hz, 1H), 7.76 (dd, J = 8.5, 2.2 Hz, 1H), 7.63 (d, J = 8.5 Hz, 1H), 5.80 (s, 1H), 2.85 (s, 3H), 2.48 (s, 3H), 2.15 (s, 3H). ^{13}C NMR (100 MHz, DMSO-d_6) δ (ppm) 170.9, 165.6, 161.1, 154.1, 151.5, 146.4, 137.1, 135.3, 125.5, 125.0, 120.4, 104.5, 23.6, 21.5, 21.0.

6-methyl-2-((4,6,8-trimethylquinazolin-2-yl)amino)pyrimidin-4(3H)-one (16c).

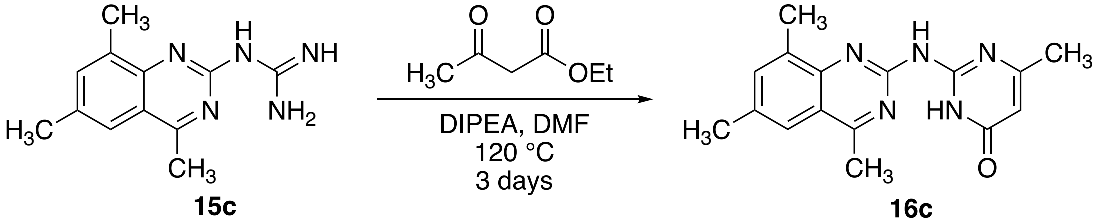

15c **16c**

A vial (2-5 mL) was charged with **15c (**250 mg, 1.09 mmol). Then, DMF (2 mL), DIPEA (380 mL, 2.18 mmol), and ethyl acetoacetate (1.4 mL, 11 mmol) were added sequentially, and the solution was stirred at 120 °C for 3 days. The mixture was then diluted with Et_2O and sonicated. The precipitate was collected with suction filtration and washed with more Et_2O to afford **16c** (295 mg, 92%) as a pale beige solid. ^{1}H NMR (400 MHz, DMSO-d_6) δ (ppm) 13.61 (s, 1H, NH), 11.27 (s, 1H, NH), 7.83 (s, 1H), 7.66 (s, 1H), 5.82 (s, 1H), 2.85 (s, 3H), 2.59 (s, 3H), 2.46 (s, 3H), 2.16 (s, 3H). ^{13}C NMR (151 MHz, DMSO-d_6) δ (ppm) 171.3, 165.7, 161.0, 153.4, 151.7, 145.4, 136.9, 134.6, 133.1, 122.6, 120.2, 104.4, 23.7, 21.7, 21.0, 17.1.

2-((6-methoxy-4-methylquinazolin-2-yl)amino)-6-methylpyrimidin-4(3H)-one (16d).

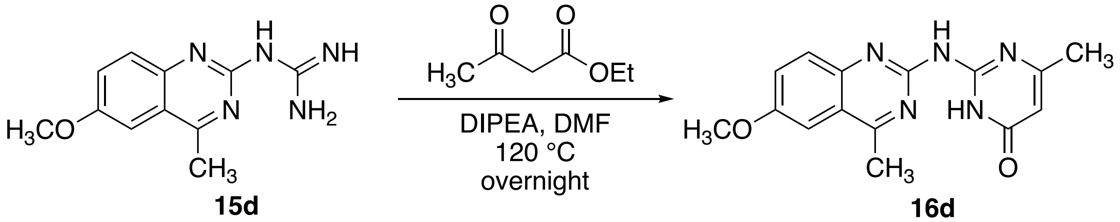

15d **16d**

A vial (2-5 mL) was charged with **15d** (231 mg, 1.00 mmol). Then, DMF (3 mL), DIPEA (348 mL, 2.00 mmol), and ethyl acetoacetate (1.28 mL, 10.2 mmol) were added sequentially, and the solution was stirred at 120 °C for 40 h. The mixture was then diluted with Et_2O and sonicated. The precipitate was collected with suction filtration and washed with more Et_2O to afford **16d** (210 mg, 71%) as a pale beige solid. ^{1}H NMR (600 MHz, DMSO-d_6) δ (ppm) 13.31 (s, 1H), 11.20 (s, 1H), 7.72 (d, J = 9.1 Hz, 1H), 7.61 (dd, J = 9.1, 2.8 Hz, 1H), 7.50 (d, J = 2.8 Hz, 1H), 5.80 (s, 1H), 3.94 (s, 3H), 2.89 (s, 3H), 2.15 (s, 3H). ^{13}C NMR (151 MHz, DMSO-d_6) δ (ppm) 170.0, 165.7, 161.1, 156.5, 153.2, 151.6, 143.8, 127.4, 127.0, 121.2, 104.6, 104.3, 55.8, 23.7, 21.8.

2-((6-chloro-4-methylquinazolin-2-yl)amino)-6-methylpyrimidin-4(3H)-one (16e).

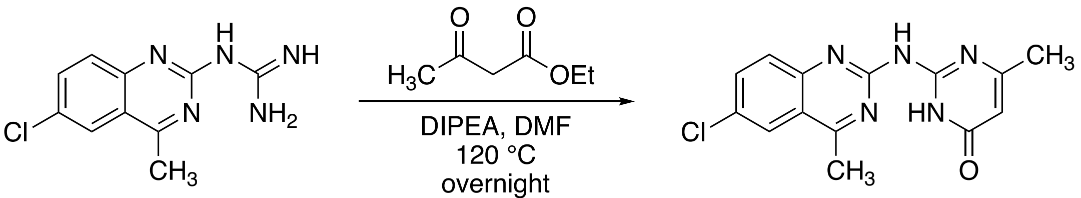

15e **16e**

A vial (2-5 mL) was charged with **15e** (236 mg, 1.01 mmol). Then, DMF (3 mL), DIPEA (348 mL, 2.00 mmol), and ethyl acetoacetate (1.28 mL, 10.2 mmol) were added sequentially, and the solution was stirred at 120 °C overnight. The mixture was then diluted with Et_2O and sonicated. The precipitate was collected with suction filtration and washed with more Et_2O to afford **16e** (175 mg, 58%) as a pale beige solid. ^{1}H NMR (600 MHz, DMSO-d_6) δ (ppm) 13.20 (s, 1H), 11.42 (s, 1H), 8.32 (d, J = 2.5 Hz, 1H), 7.95 (dd, J = 8.9, 2.4 Hz, 1H), 7.79 (d, J = 8.9 Hz, 1H), 5.84 (s, 1H), 2.89 (s, 3H), 2.16 (s, 3H). ^{13}C NMR (151 MHz, DMSO-d_6) δ (ppm) 171.5, 165.5, 161.0, 154.8, 151.3, 146.9, 135.5, 129.5, 128.0, 125.4, 121.2, 104.8, 23.6, 21.7.

N4-(3-(dimethylamino)propyl)-6-methyl-N2-(4-methylquinazolin-2-yl)pyrimidine-2,4-diamine (3).

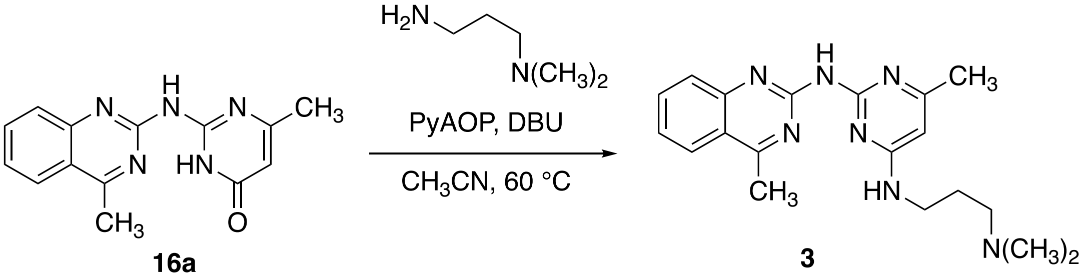

To a vial containing **16a** (267 mg, 1.00 mmol) in CH_3CN (4 mL), PyAOP (573 mg, 1.10 mmol) and DBU (224 mL, 1.50 mmol) were added successively, and the mixture was stirred at 60 °C overnight. Propylamine (252 mL, 2.00 mmol) was then added and the solution was stirred for 3 h at 60 °C. The reaction solution concentrated under reduced pressure and then diluted with water and the AQ layer was then extracted with $CHCl_3$:IPA (3:1, 15 mL × 3), and the combined org. layer was dried over Na_2SO_4, filtered, and concentrated under reduced pressure. Purification by flash column chromatography (eluent: 15%→20% MeOH (1% NH_4OH) in CH_2Cl_2) afforded **3** (75 mg, 21%) as a pale-yellow solid. ^1H NMR (400 MHz, DMSO-d_6) δ (ppm) 9.44 (s, 1H), 8.10 (dd, J = 8.3, 1.3 Hz, 1H), 7.80 (ddd, J = 8.3, 6.8, 1.4 Hz, 1H), 7.65 (d, J = 8.3 Hz, 1H), 7.43 (ddd, J = 8.1, 6.8, 1.2 Hz, 1H), 7.17 (s, 1H), 3.65 – 3.14 (m, 2H), 2.81 (s, 3H), 2.31 (t, J = 7.0 Hz, 2H), 2.16 (s, 3H), 2.13 (s, 6H), 1.73 (p, J = 7.1 Hz, 2H). ^{13}C NMR (100 MHz, DMSO-d_6) δ (ppm) 169.2, 163.4, 161.7, 158.3, 155.5, 150.6, 133.8, 133.7, 126.6, 125.6, 124.1, 120.3, 56.6, 44.9, 38.2, 26.7, 23.2, 21.3. HRMS: m/z: [M-H]$^+$ calcd for $C_{19}H_{26}N_7^+$ 352.2244; Found 352.2244.

N4-(3-(dimethylamino)propyl)-N2-(4,6-dimethylquinazolin-2-yl)-6-methylpyrimidine-2,4-diamine (4).

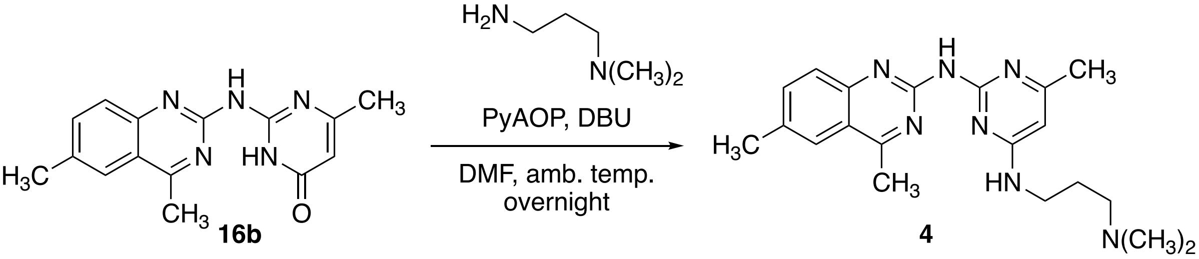

A vial was charged with **16b** (100 mg, 0.356 mmol) and PyAOP (241 mg, 0.462 mmol). Then, DMF (2 mL) and DBU (80 mL, 0.54 mmol) were added and the resulting solution was stirred for 1 h. Propylamine (112 mL, 0.890 mmol) was then added and the solution was stirred at ambient temperature overnight. The solvent was removed under reduced pressure and the crude mixture was diluted in water and extracted with IPA:$CHCl_3$ (3 × 10 mL). The combined org. layer was then dried over Na_2SO_4, filtered, and concentrated under reduced pressure. Purification by flash column chromatography (eluent: 15% → 20% MeOH (1% NH_4OH) in CH_2Cl_2) to afford **4** (85 mg, 65%) as a pale-yellow solid. ^1H NMR (600 MHz, DMSO-d_6) δ (ppm) 7.87 (s, 1H), 7.64 (dd, J = 8.5, 1.8 Hz, 1H), 7.57 (d, J = 8.5 Hz, 1H), 7.14 (s, 1H), 5.94 (s, 1H), 3.27 – 3.21 (m, 2H), 2.78 (s, 3H), 2.47 (s, 3H), 2.28 (t, J = 7.1 Hz, 2H), 2.15 (s, 3H), 2.11 (s, 6H), 1.71 (p, J = 7.1 Hz, 2H). ^{13}C NMR (151 MHz, DMSO-d_6) δ (ppm) 168.3, 163.4, 158.4, 155.0, 149.1, 135.6, 133.5, 126.4, 124.4, 120.2, 97.9, 56.7, 45.0, 38.1, 26.8, 23.3, 21.3, 21.0. HRMS: m/z: [M-H]$^+$ calcd for $C_{20}H_{28}N_7^+$ 366.2401; Found 366.2425.

N4-(3-(dimethylamino)propyl)-6-methyl-N2-(4,6,8-trimethylquinazolin-2-yl)pyrimidine-2,4-diamine (5).

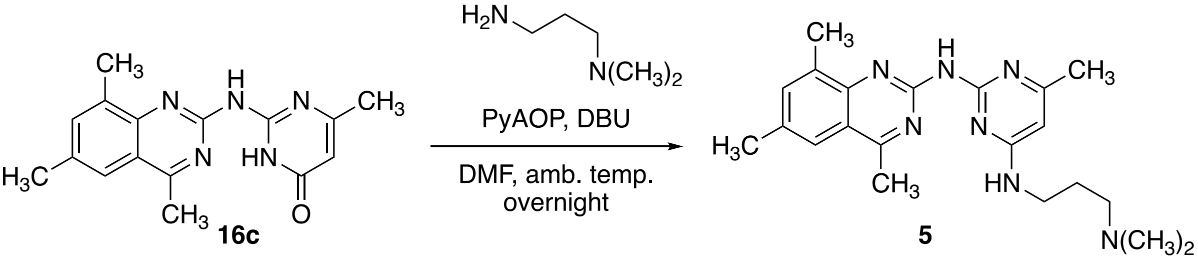

A vial was charged with **16c** (100 mg, 0.339 mmol) and PyAOP (230 mg, 0.441 mmol). Then, DMF (2 mL) and DBU (76 mL, 0.51 mmol) were added and the resulting solution was stirred for 1 h. Propylamine (107 mL, 0.850 mmol) was then added and the solution was stirred at ambient temperature overnight. The solvent was removed under reduced pressure and the crude mixture was diluted in water and extracted with IPA:$CHCl_3$ (3 × 10 mL). The combined org. layer was then dried over Na_2SO_4, filtered, and concentrated under reduced pressure. Purification by flash column chromatography (eluent: 15% → 25% MeOH (1% NH_4OH) in CH_2Cl_2) to afford **5** (82 mg, 64%) as a pale-yellow solid. ^{1}H NMR (600 MHz, DMSO-d_6) δ (ppm) 7.69 (s, 1H), 7.50 (s, 1H), 7.05 (s, 1H), 5.93 (s, 1H), 3.25 (app. s, 2H), 2.76 (s, 3H), 2.56 (s, 3H), 2.42 (s, 3H), 2.22 (t, *J* = 7.1 Hz, 2H), 2.15 (s, 3H), 2.07 (s, 6H), 1.63 (p, *J* = 7.1 Hz, 2H). ^{13}C NMR (151 MHz, DMSO-d_6) δ (ppm) 168.3, 163.4, 158.5, 154.1, 148.1, 135.4, 134.3, 132.7, 121.9, 120.0, 97.7, 56.8, 45.1, 38.2, 27.0, 23.3, 21.4, 21.1, 16.9. HRMS: *m/z*: [M-H]$^+$ calcd for $C_{21}H_{30}N_7^+$ 380.2557; Found 380.2559.

N4-(3-(dimethylamino)propyl)-N2-(6-methoxy-4-methylquinazolin-2-yl)-6-methylpyrimidine-2,4-diamine (6).

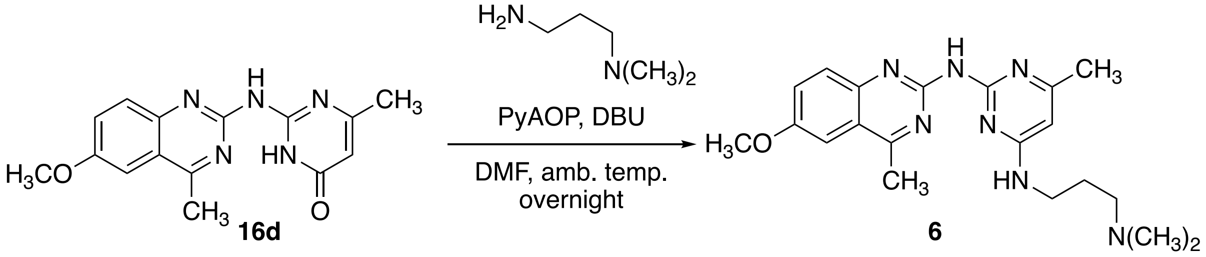

A vial was charged with **16d** (74 mg, 0.25 mmol) and PyAOP (169 mg, 0.325 mmol). Then, DMF (2.5 mL) and DBU (56 mL, 0.38 mmol) were added and the resulting solution was stirred for 1 h. Propylamine (79 mL, 0.63 mmol) was then added and the solution was stirred at ambient temperature overnight. The solvent was removed under reduced pressure and the crude mixture was diluted in water and extracted with IPA:$CHCl_3$ (3 × 10 mL). The combined org. layer was then dried over Na_2SO_4, filtered, and concentrated under reduced pressure. Purification by flash column chromatography (eluent: 15% → 20% MeOH (1% NH_4OH) in CH_2Cl_2) to afford **6** (40 mg, 42%) as a pale-yellow solid. ^{1}H NMR (600 MHz, DMSO-d_6) δ (ppm) 9.22 (s, 1H), 7.62 (d, *J* = 9.1 Hz, 1H), 7.48 (dd, *J* = 9.1, 2.7 Hz, 1H), 7.39 (d, *J* = 2.8 Hz, 1H), 7.13 (s, 1H), 3.91 (s, 3H), 3.41 – 3.29 (m, 2H), 2.80 (s, 3H), 2.36 – 2.24 (m, 2H), 2.14 (s, 3H), 2.12 (s, 6H), 1.71 (p, *J* = 7.0 Hz, 2H). ^{13}C NMR (151 MHz, DMSO-d_6) δ (ppm) 167.5, 163.3, 158.5, 155.6, 154.2, 146.3, 128.2, 125.7, 120.8, 103.9, 97.7, 56.7, 55.6, 45.0, 38.1, 26.9, 23.3, 21.5. HRMS: *m/z*: [M-H]$^+$ calcd for $C_{20}H_{28}N_7O^+$ 382.2350 ; Found 382.2351.

N2-(6-chloro-4-methylquinazolin-2-yl)-N4-(3-(dimethylamino)propyl)-6-methylpyrimidine-2,4-diamine (7).

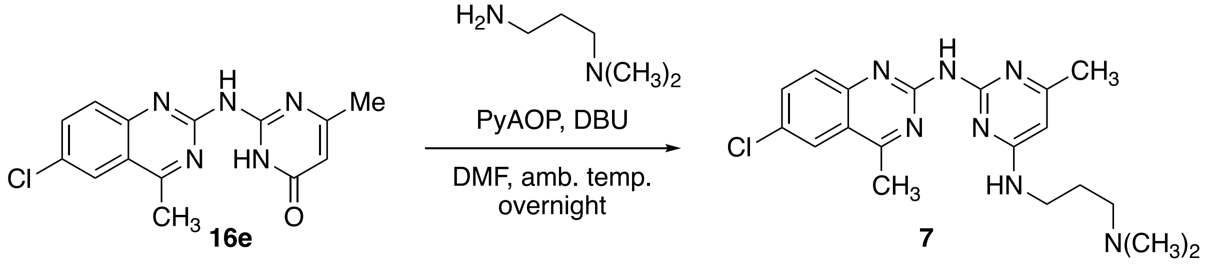

A vial was charged with **16e** (75 mg, 0.25 mmol) and PyAOP (169 mg, 0.325 mmol). Then, DMF (2.5 mL) and DBU (56 mL, 0.38 mmol) were added and the resulting solution was stirred for 1 h. Propylamine (79 mL, 0.63 mmol) was then added and the solution was stirred at ambient temperature overnight. The solvent was removed under reduced pressure and the crude mixture was diluted in water and extracted with IPA:$CHCl_3$ (3 × 10 mL). The combined org. layer was then dried over Na_2SO_4, filtered, and concentrated under reduced pressure. Purification by flash column chromatography (eluent: 15% → 20% MeOH (1% NH_4OH) in CH_2Cl_2) to afford **7** (60 mg, 62%) as a pale-yellow solid. ^{1}H NMR (600 MHz, DMSO-d_6) δ (ppm) 9.53 (s, 1H), 8.17 (d, *J* = 2.3 Hz, 1H), 7.80 (dd, *J* = 8.9, 2.3 Hz, 1H), 7.65 (d, *J* = 8.9 Hz, 1H), 7.18 (s, 1H), 5.97 (s, 1H), 3.29 – 3.23 (m, 2H), 2.80 (s, 3H), 2.31 (t, *J* = 6.9 Hz, 2H), 2.15 (s, 3H), 2.14 (s, 6H), 1.72 (t, *J* = 7.1 Hz, 2H). ^{13}C NMR (151 MHz, DMSO-d_6) δ (ppm) 168.9, 163.4, 158.1,

155.8, 149.4, 134.1, 128.7, 127.8, 124.7, 120.9, 98.3, 56.6, 44.8, 38.1, 26.7, 23.3, 21.4. HRMS: *m/z*: $[M-H]^+$ calcd for $C_{19}H_{25}ClN_7^+$ 386.1854; Found 386.1853.

quinoxalin-2(1H)-one (17).

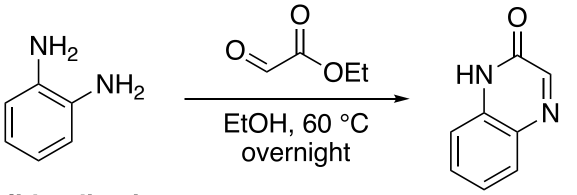

***o*-phenylidendiamine** **17**

A flask was charged with *o*-phenylidendiamine (1.50 g, 13.9 mmol). Then, EtOH (6 mL) and ethyl glyoxalate (50% in $PhCH_3$, 3.30 mL, 16.6 mmol) were added and the mixture was stirred at 60 °C overnight. The reaction was then allowed to cool to ambient temperature and the solids were collected with suction filtration and washed with water to afford **17** (1.86 g, 92%) as a pale beige solid. ^1H NMR (400 MHz, DMSO-d_6) δ (ppm) 12.42 (s, 1H), 8.17 (s, 1H), 7.78 (dd, J = 8.4, 1.4 Hz, 1H), 7.60 – 7.49 (m, 1H), 7.35 – 7.26 (m, 2H). *The data is consistent with that reported in the literature* [12]

2-chloroquinoxaline (18).

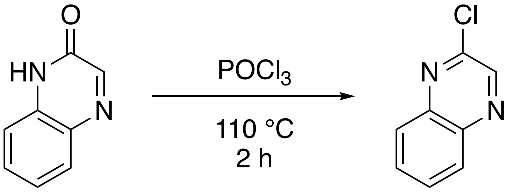

17 **18**

To a RBF (25 mL) containing **17** (500 mg, 3.42 mmol) was added $POCl_3$ (3.20 mL, 34.3 mmol) and the mixture was refluxed for 2 h. Then, the reaction was allowed to cool to ambient temperature and poured onto ice water (ca 20 mL). The organic layer was then extracted with EtOAc (3 × 20 mL), and the combined org. layer was then washed with sat. $NaHCO_3$ (20 mL), water (20 mL), and sat. NaCl sol. (20 mL). The resulting org. layer was dried over Na_2SO_4, filtered, and concentrated under reduced pressure to afford **18** (550 mg, 98%) as a pale-brown solid. ^1H NMR (400 MHz, $CDCl_3$) δ (ppm) 8.79 (s, 1H), 8.15 – 8.10 (m, 1H), 8.06 – 8.00 (m, 1H), 7.85 – 7.75 (m, 2H). *The data is consistent with that reported in the literature*[13]

1-(quinoxalin-2-yl)guanidine (19).

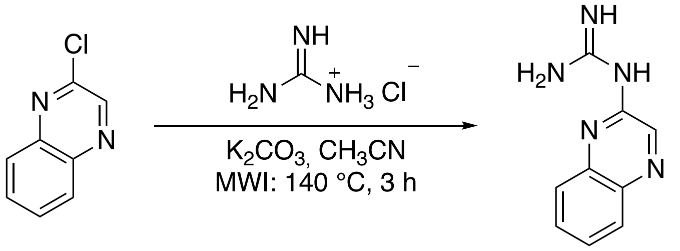

18 **19**

To a μ-wave vial (10-20 mL) was added **18** (300 mg, 1.82 mmol), guanidine·HCl (348 mg, 3.64 mmol), and K_2CO_3 (756 mg, 5.47 mmol). Then, CH_3CN (12 mL) was added, and the reaction was heated using MWI (140 °C, 3 h). *If **18** remained on TLC, the reaction time was extended by another 30 min*. The mixture was then concentrated under reduced pressure and diluted with water ~20 mL. The resulting AQ layer was extracted with EtOAc (20 mL × 4), and the combined org. layer was dried over Na_2SO_4, filtered, a concentrated under reduced pressure. **19** (247 mg, 72%) was then obtained as a yellow solid. ^1H NMR (400 MHz, DMSO-d_6) δ (ppm) 8.22 (s, 1H), 7.76 (d, J = 8.1 Hz, 1H), 7.58 (dd, J = 19.0, 7.6 Hz, 2H), 7.37 (t, J = 7.6 Hz, 1H). 7.29 (s, 4H, NH). ^{13}C NMR (100 MHz, DMSO-d_6) δ (ppm) 160.2, 157.8, 147.9, 140.4, 136.6, 129.2, 128.2, 125.8, 124.2.

6-methyl-2-(quinoxalin-2-ylamino)pyrimidin-4(3H)-one (20).

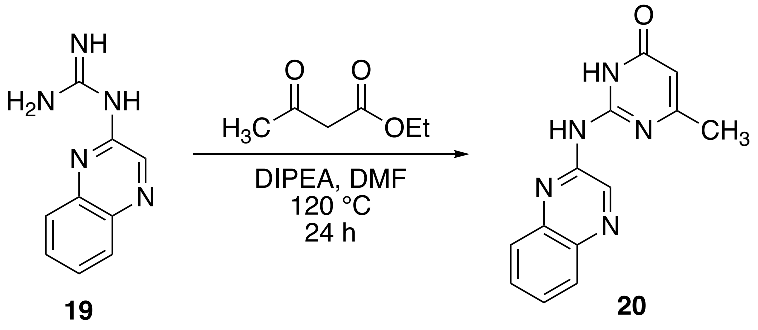

A vial was charged with **19** (130 mg, 0.694 mmol)**.** Then, DMF (1 mL), DIPEA (242 µL, 1.39 mmol), and ethyl acetoacetate (886 µL, 6.94 mmol) were added sequentially, and the solution was stirred at 120 °C for 24 h. The mixture was then diluted with Et_2O, and the precipitate was collected with suction filtration. The solids were washed with more Et_2O to afford **20** (80 mg, 46%) as a beige solid. ^{1}H NMR (600 MHz, DMSO-d_6) δ (ppm) 12.85 (s, 1H), 11.66 (s, 1H, NH), 8.77 (s, 1H, NH), 7.98 (d, J = 8.2 Hz, 1H), 7.83 (dd, J = 8.3, 1.4 Hz, 1H), 7.81 – 7.76 (m, 1H), 7.68 – 7.63 (m, 1H), 5.83 (s, 1H), 2.18 (s, 3H). ^{13}C NMR (151 MHz, DMSO-d_6) δ (ppm) 161.1, 151.4, 148.5, 140.9, 138.2, 137.9, 131.0, 128.8, 128.3, 127.3, 126.3, 104.4, 22.7.

N4-(3-(dimethylamino)propyl)-6-methyl-N2-(quinoxalin-2-yl)pyrimidine-2,4-diamine (8).

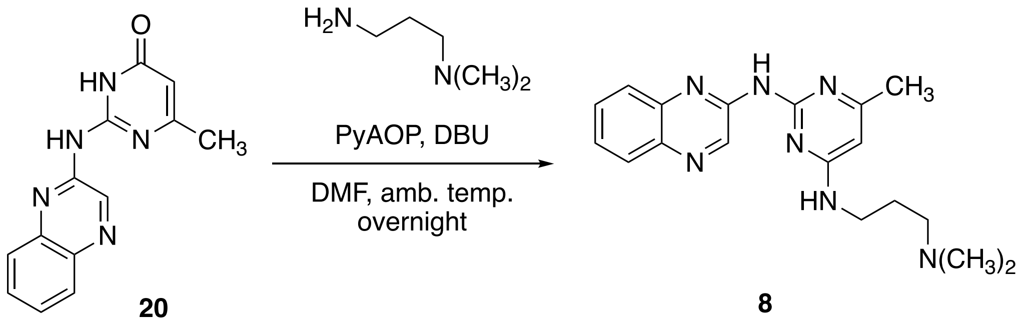

A vial was charged with **20** (60 mg, 0.24 mmol) and PyAOP (160 mg, 0.307 mmol). Then, DMF (1 mL) and DBU (53 µL, 0.36 mmol) were added and the resulting solution was stirred at ambient temperature for 1 h. Propylamine (88 µL, 0.59 mmol) was then added and the solution was stirred at ambient temperature overnight. The solvent was removed under reduced pressure and the mixture was diluted with water. The AQ layer was then extracted with $CHCl_3$:IPA (3:1, 10 mL × 3) and the combined org. layer was dried over Na_2SO_4, filtered, and concentrated under reduced pressure. Purification by flash column chromatography (eluent: 6% → 12% MeOH (1% NH_4OH) in CH_2Cl_2) afforded **8** (61 mg, 76%) as a yellow solid. ^{1}H NMR (600 MHz, DMSO-d_6) δ (ppm) 9.88 (s, 1H), 9.80 (s, 1H, NH), 7.95 (d, J = 8.2 Hz, 1H), 7.77 (d, J = 8.3 Hz, 1H), 7.70 (td, J = 7.5, 6.7, 1.5 Hz, 1H), 7.58 (td, J = 7.5, 6.6, 1.5 Hz, 1H), 7.32 (s, 1H, NH), 5.98 (s, 1H), 3.31 – 3.20 (m, 2H), 2.36 (t, J = 7.2 Hz, 2H), 2.18 (s, 9H (merged peaks)), 1.70 (p, J = 7.1 Hz, 2H). ^{13}C NMR (151 MHz, DMSO-d_6) δ (ppm) 163.2, 158.2, 149.0, 140.8, 140.8, 138.0, 130.0, 128.5, 126.7, 126.3, 98.0, 56.5, 44.9, 38.1, 26.6, 25.8. HRMS: m/z: $[M-H]^+$ calcd for $C_{18}H_{24}N_7^+$ 338.2088; Found 338.2085.

3-methylquinoxalin-2(1H)-one (21a).

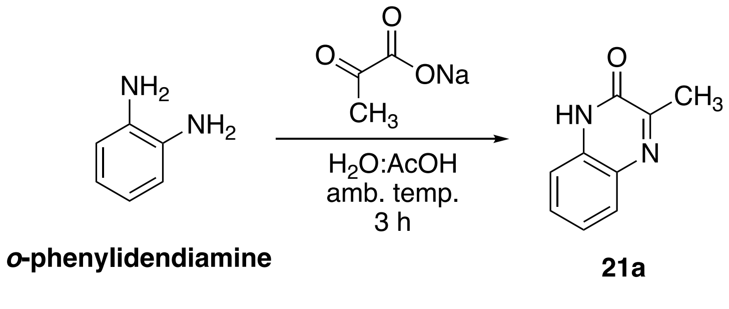

A RBF was charged with *o*-phenylidendiamine (1.62 g, 15.0 mmol) and sodium pyruvate (1.65 g, 15.0 mmol) in aq. acetic acid (20%, 25 mL). The reaction was stirred at ambient temperature for 3 hours. The resulting precipitate was filtered off and washed with water to afford **21a** (1.95 g, 81%) as a pale-brown solid. ^{1}H NMR (400 MHz, $CDCl_3$) δ (ppm) 11.61 (s, 1H), 7.81 (d, J = 8.0 Hz, 1H), 7.49 (t, J = 7.8 Hz, 1H), 7.39 – 7.30 (m, 2H), 2.64 (s, 3H). *The data is consistent with that reported in the literature*[14]

3,6,7-trimethylquinoxalin-2(1H)-one (21b).

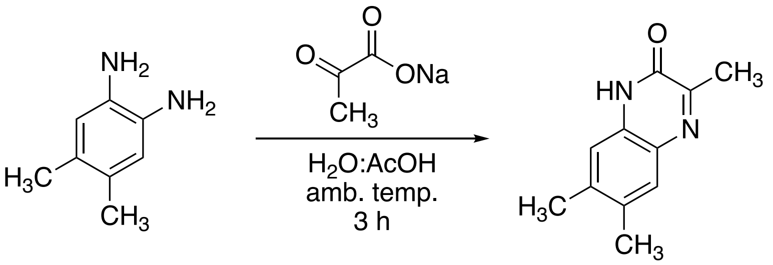

4,5-dimethylbenzene-1,2-diamine **21b**

A RBF (50 mL) was charged with 4,5-dimethylbenzene-1,2-diamine (1.00 g, 7.34 mmol) and sodium pyruvate (808 mg, 7.34 mmol) in aq. acetic acid (20%, 25 mL). The reaction was stirred at ambient temperature for 3 hours. The resulting precipitate was filtered off and washed with water to afford **21b** (1.13 g, 82%) as a pale-brown solid. ^{1}H NMR (600 MHz, DMSO-d_6) δ (ppm) 12.14 (s, 1H), 7.44 (s, 1H), 7.01 (s, 1H), 2.36 (s, 3H), 2.27 (s, 3H), 2.25 (s, 3H). ^{13}C NMR (151 MHz, DMSO-d_6) δ (ppm) 172.0, 157.7, 155.0, 138.6, 131.5, 130.2, 129.9, 127.8, 115.3, 20.5, 19.7, 18.9.

6,7-dichloro-3-methylquinoxalin-2(1H)-one (21c).

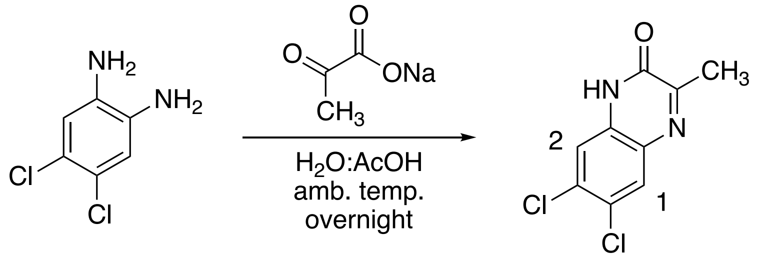

4,5-dichlorobenzene-1,2-diamine **21c**

A RBF (100 mL) was charged with 3,4-dichloro-ophenylenediamine (2.00 g, 11.3 mmol) and sodium pyruvate (1.37 g, 12.4 mmol) followed by aqueous acetic acid (20%, 50 mL). The reaction was stirred at ambient temperature overnight. The resulting precipitate was filtered off, wash with water, and dried. **21c** (2.43 g, 94%) was obtained as a brown solid. ^{1}H NMR (400 MHz, DMSO-d_6) δ (ppm) 12.43 (s, 1H, NH), 7.94 (d, J = 5.3 Hz, 1H, H-2), 7.41 (d, J = 5.3 Hz, 1H, H-1), 2.39 (s, 3H, CH_3). ^{13}C NMR (100 MHz, DMSO-d_6) δ (ppm) 161.4, 154.5, 131.9, 131.2, 131.1, 128.9, 124.7, 116.2, 20.6.

2-chloro-3-methylquinoxaline (22a).

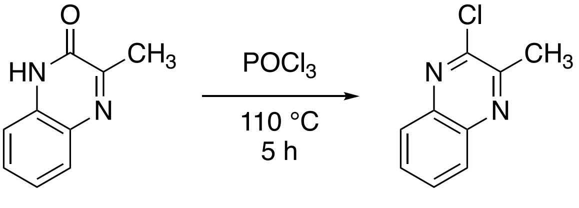

21a **22a**

To a RBF containing **21a** (1.94 g, 12.1 mmol) was added $POCl_3$ (9.70 mL, 104 mmol) and the mixture was stirred at 110 °C overnight. Then, the reaction was allowed to cool to ambient temperature and poured onto ice water (ca 20 mL). The organic layer was then extracted with EtOAc (3 × 30 mL), and the combined org. layer was then

washed with sat. $NaHCO_3$ (20 mL), water (20 mL), and sat. NaCl sol. (20 mL). The resulting org. layer was dried over Na_2SO_4, filtered, and concentrated under reduced pressure to afford **22a** (1.0 g, 46%) as a pale-brown solid. ^1H NMR (400 MHz, $CDCl_3$) δ (ppm) 8.06 – 8.01 (m, 1H), 8.00 – 7.96 (m, 1H), 7.79 – 7.68 (m, 2H), 2.85 (s, 3H). ^{13}C NMR (151 MHz, $CDCl_3$) δ (ppm) 152.9, 148.0, 141.1, 141.0, 130.2, 130.1, 128.6, 128.3, 23.5.

2-chloro-3,6,7-trimethylquinoxaline (22b).

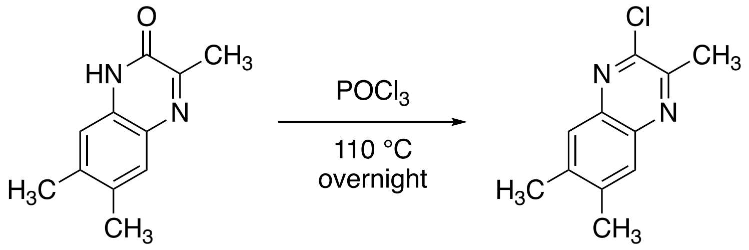

21b **22b**

To a RBF containing **21b** (500 mg, 2.66 mmol) was added $POCl_3$ (2.50 mL, 26.8 mmol) and the mixture was stirred at 110 °C overnight. Then, the reaction was allowed to cool to ambient temperature and poured onto ice water (ca 20 mL). The organic layer was then extracted with EtOAc (3 × 30 mL), and the combined org. layer was then washed with sat. $NaHCO_3$ (20 mL), water (20 mL), and sat. NaCl sol. (20 mL). The resulting org. layer was dried over Na_2SO_4, filtered, and concentrated under reduced pressure to afford **22b** (402 mg, 73%) as a dark-brown solid. ^1H NMR (600 MHz, DMSO-d_6) δ (ppm) 7.78 (s, 1H), 7.73 (s, 1H), 2.70 (s, 3H), 2.43 (s, 6H). ^{13}C NMR (151 MHz, DMSO-d_6) δ (ppm) 151.4, 146.2, 140.8, 140.7, 139.2, 139.1, 127.0, 126.6, 22.8, 19.8, 19.7.

2,6,7-trichloro-3-methylquinoxaline (22c).

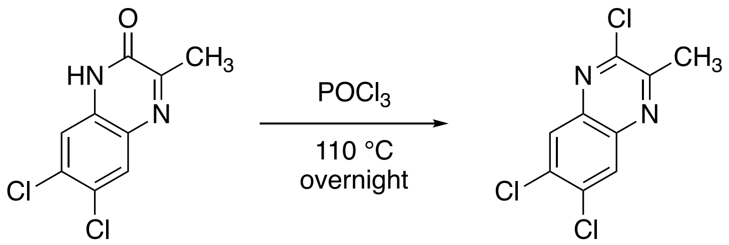

21c **22c**

To a RBF containing **21c** (790 mg, 3.45 mmol) was added $POCl_3$ (3.20 mL, 34.3 mmol) and the mixture was stirred at 110 °C overnight. Then, the reaction was allowed to cool to ambient temperature, and the volatiles were removed under reduced pressure. The resulting crude mixture was diluted with sat. $NaHCO_3$ (150 mL) and the aq. layer was then extracted with DCM (3 × 75 mL). *If the organic layer is sluggish, filter once over Celite before the wash.* The combined org. layer was then washed with sat. NaCl sol. (2 x 50 mL). The resulting org. layer was dried over Na_2SO_4, filtered over Celite, and concentrated under reduced pressure. **22c** (480 mg, 56%) was then obtained as a dark-brown solid without further purification. ^1H NMR (400 MHz, DMSO-d_6) δ (ppm) 8.38 (s, 1H), 8.36 (s, 1H), 2.75 (s, 3H). ^{13}C NMR (100 MHz, DMSO-d_6) δ (ppm) 154.8, 148.9, 139.3, 139.1, 133.1, 133.0, 129.1, 128.8, 23.1.

1-(3-methylquinoxalin-2-yl)guanidine (23a).

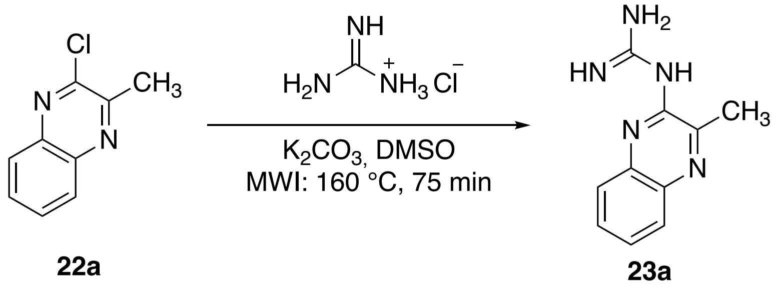

22a **23a**

To a μ-wave vial (10-20 mL) was added **22a** (500 mg, 2.80 mmol), guanidine·HCl (535 mg, 5.60 mmol), and K_2CO_3 (1.16 g, 8.40 mmol). Then, DMSO (15 mL) was added, and the reaction was heated using MWI (160 °C, 75 min). The mixture was then diluted with water and extracted with EtOAc (4 X 30 mL). The combined org. layer was washed with water (3 X 10 mL) and once with sat. NaCl sol. (1 X 15 mL) and dried over $MgSO_4$. The solvent was removed under reduced pressure to afford **23a** (500 mg, 89%) as a yellow solid. ^{1}H NMR (400 MHz, $CDCl_3$) δ (ppm) 7.85 (dd, J = 8.1, 1.5 Hz, 1H), 7.68 – 7.59 (m, 1H), 7.50 (ddd, J = 8.3, 6.9, 1.5 Hz, 1H), 7.42 (ddd, J = 8.4, 7.0, 1.5 Hz, 1H), 2.71 (s, 3H). ^{13}C NMR (100 MHz, $CDCl_3$) δ (ppm) 158.4, 156.3, 154.9, 139.8, 137.4, 128.3, 127.8, 125.9, 125.5, 22.6.

1-(3,6,7-trimethylquinoxalin-2-yl)guanidine (23b).

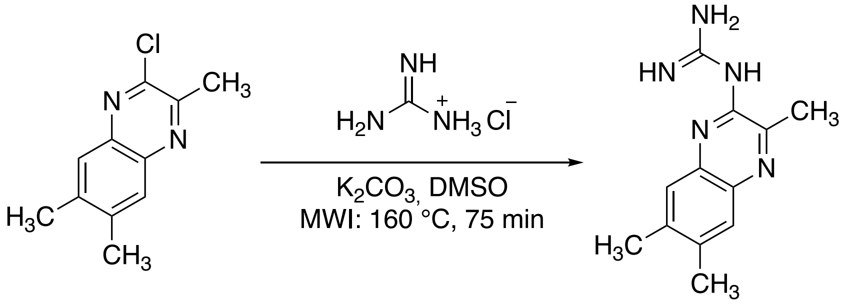

22b **23b**

To a MW vial (2-5 mL) was added **22b** (200 mg, 0.968 mmol), guanidine·HCl (185 mg, 1.94 mmol), and K_2CO_3 (401 mg, 2.90 mmol). Then, DMSO (3 mL) was added, and the reaction was heated using MWI (160 °C, 75 min). The mixture was then diluted with water and extracted with EtOAc (4 ´ 20 mL). The organic layer was washed with water (3 ´ 20 mL), once with sat. NaCl sol. (20 mL), and dried over Na_2SO_4. The solvent was removed under reduced pressure to afford **23b** (161 mg, 73%) as a dark brown solid. ^{1}H NMR (400 MHz, DMSO-d_6) δ (ppm) 7.45 (s, 1H), 7.35 (s, 1H), 7.20 (s, 4H, NH), 2.50 (s, 3 H) 2.33 (s, 3H), 2.32 (s, 3H). ^{13}C NMR (100 MHz, DMSO-d_6) δ (ppm) 159.3, 153.2, 138.2, 137.1, 134.8, 133.1, 127.1, 126.9, 125.0, 22.3, 19.7, 19.4.

1-(6,7-dichloro-3-methylquinoxalin-2-yl)guanidine (23c).

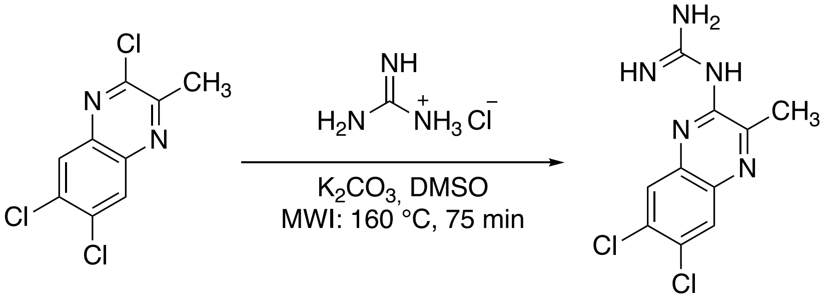

22c **23c**

To a MW vial (2-5 mL) was added **22c** (300 mg, 01.21 mmol), guanidine·HCl (232 mg, 2.43 mmol), and K_2CO_3 (503 mg, 3.64 mmol). Then, DMSO (5 mL) was added, and the reaction was heated using MWI (160 °C, 75 min). The mixture was then diluted with water and extracted with $CHCl_3$:IPA (3:1, 3 x 15 mL). *If the organic layer is sluggish, filter once over Celite with CH_2Cl_2 before the wash.* The organic layer was washed with sat. NaCl sol. (5 x 15 mL) and dried over Na_2SO_4, filtered, and concentrated under reduced pressure. **23c** (230 mg, 70% (residual DMSO calculated for)) was then obtained as a dark-brown solid. ^{1}H NMR (400 MHz, DMSO-d_6) δ (ppm) 7.87 (s, 1H), 7.84 (s, 1H), 7.36 (s, 4H, NH), 2.51 (s, 3H). ^{13}C NMR (100 MHz, DMSO-d_6) δ (ppm) 160.2, 157.1, 156.7, 139.7, 135.0, 130.0, 128.0, 125.9, 125.2, 22.4.

6-methyl-2-((3-methylquinoxalin-2-yl)amino)pyrimidin-4(3H)-one (24a).

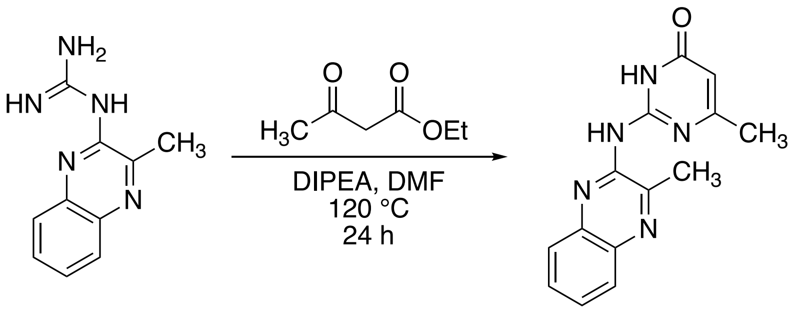

23a **24a**

A vial was charged with **23a** (167 mg, 0.830 mmol)**.** Then, DMF (2 mL), DIPEA (289 µL, 1.66 mmol), and ethyl acetoacetate (1.06 mL, 8.30 mmol) were added sequentially, and the solution was stirred at 120 °C for 24 h. The mixture was then diluted with Et_2O, and the precipitate was collected with suction filtration. The solids were washed with more Et_2O to afford **24a** (166 mg, 59%) as a beige solid. ^1H NMR (400 MHz, DMSO-d_6) δ (ppm) 13.17 (s, 1H), 12.19 (s, 1H), 7.85 (d, J = 7.9 Hz, 1H), 7.74 (d, J = 8.1 Hz, 1H), 7.65 (s, 1H), 7.56 (s, 1H), 5.64 (s, 1H), 2.68 (s, 3H), 2.21 (s, 3H). *No carbon NMR could be obtained due to poor solubility.*

6-methyl-2-((3,6,7-trimethylquinoxalin-2-yl)amino)pyrimidin-4(3H)-one (24b).

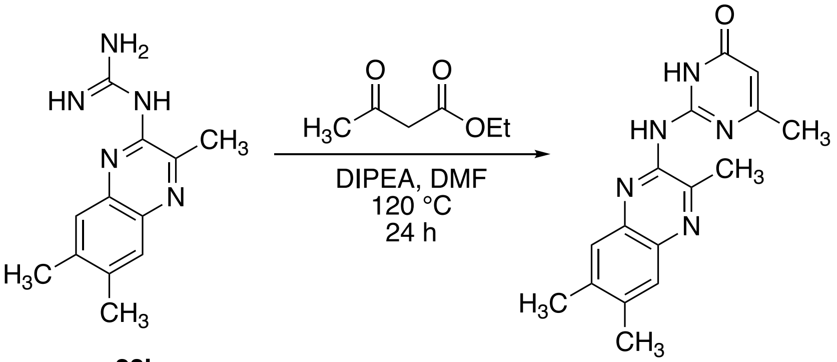

23b **24b**

A vial was charged with **23b** (200 mg, 0.872 mmol)**.** Then, DMF (2 mL), DIPEA (304 µL, 1.75 mmol), and ethyl acetoacetate (1.11 mL, 8.72 mmol) were added sequentially, and the solution was stirred at 120 °C for 24 h. The mixture was then diluted with Et_2O, and the precipitate was collected with suction filtration. The solids were washed with more Et_2O to afford **24b** (152 mg, 59%) as a dark-brown solid. ^1H NMR (400 MHz, DMSO-d_6) δ (ppm) 13.23 (s, 1H, NH), 12.09 (s, 1H, NH), 7.61 (s, 1H), 7.52 (s, 1H), 5.65 (s, 1H), 2.65 (s, 3H), 2.40 (s, 3H), 2.38 (s, 3H), 2.20 (s, 3H). *No carbon NMR could be obtained due to poor solubility.*

2-((6,7-dichloro-3-methylquinoxalin-2-yl)amino)-6-methylpyrimidin-4(3H)-one (24c).

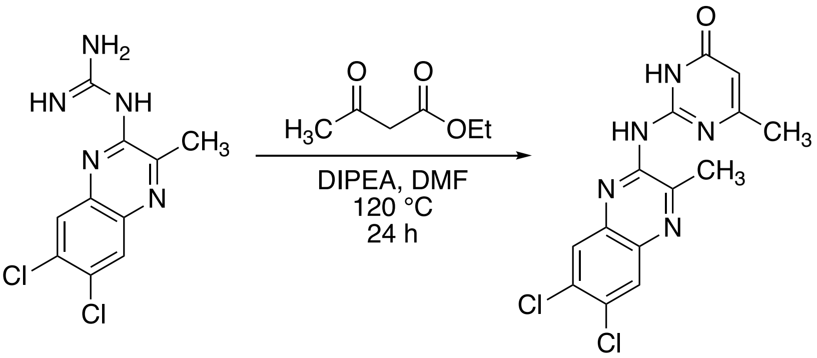

23c **24c**

A vial was charged with **23c** (260 mg, 0.963 mmol)**.** Then, DMF (3 mL), DIPEA (335 µL, 1.92 mmol), and ethyl acetoacetate (1.23 mL, 9.64 mmol) were added sequentially, and the solution was stirred at 120 °C for 24 h. The mixture was then diluted with Et_2O, and the precipitate was collected with suction filtration. The solids were washed with more Et_2O to afford **24c** (152 mg, 59%) as a dark-brown solid. ^{1}H NMR (400 MHz, DMSO-d_6) δ (ppm) 12.73 (s, 1H), 12.15 (s, 1H), 8.00 (s, 1H), 7.92 (s, 1H), 5.65 (s, 1H), 2.60 (s, 3H), 2.21 (s, 3H). ^{13}C NMR (151 MHz, DMSO-d_6) δ (ppm) 162.3, 161.1, 156.1, 154.4, 150.8, 137.8, 136.1, 131.1, 128.4, 128.0, 126.4, 101.8, 22.0, 19.0.

N4-(3-(dimethylamino)propyl)-6-methyl-N2-(3-methylquinoxalin-2-yl)pyrimidine-2,4-diamine (9).

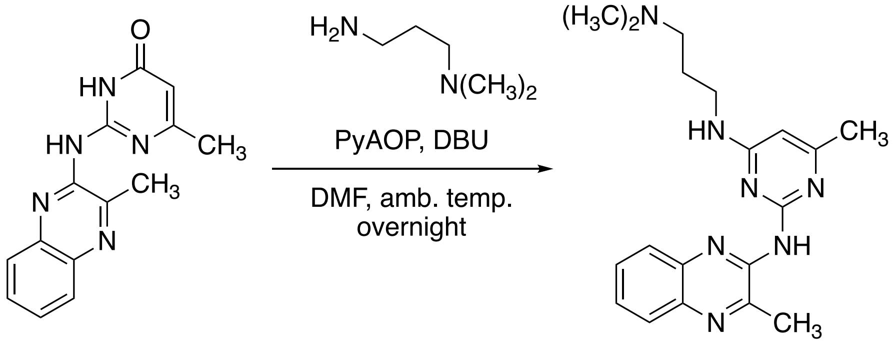

24a **9**

A vial was charged with **24a** (166 mg, 0.49 mmol) and PyAOP (331 mg, 0.634 mmol). Then, DMF (5 mL) and DBU (109 µL, 0.732 mmol) were added and the resulting solution was stirred at ambient temperature for 1 h. Propylamine (92 µL, 0.73 mmol) was then added and the solution was stirred at ambient temperature overnight. The solvent was removed under reduced pressure and the mixture was diluted with water. The AQ layer was then extracted with $CHCl_3$:IPA (3:1, 15 mL × 3) and the combined org. layer was dried over Na_2SO_4, filtered, and concentrated under reduced pressure. Purification by flash column chromatography (eluent: 6% → 10% MeOH (1% NH_4OH) in CH_2Cl_2) afforded **9** (78 mg, 46%) as a yellow solid. ^{1}H NMR (400 MHz, DMSO-d_6) δ (ppm) 9.26 (s, 1H), 7.93 (d, J = 7.9 Hz, 1H), 7.81 (d, J = 8.0 Hz, 1H), 7.67 (q, J = 8.8, 7.8 Hz, 2H), 7.19 (s, 1H), 5.90 (s, 1H), 3.30 (s, 2H), 3.17 (s, 2H), 2.59 (s, 3H), 2.37 – 2.31 (m, 6H), 2.12 (s, 3H), 1.67 (d, J = 9.8 Hz, 2H). ^{13}C NMR (100 MHz, DMSO-d_6) δ (ppm) 163.3, 159.7, 152.2, 148.8, 139.7, 138.8, 129.0, 128.9, 127.7, 127.4, 127.0, 97.4, 55.6, 43.5, 37.5, 25.5, 23.1, 22.2. HRMS: *m/z*: [M-H]$^+$ calcd for $C_{19}H_{26}N_7^+$ 352.2244; Found 352.2242.

N4-(3-(dimethylamino)propyl)-6-methyl-N2-(3,6,7-trimethylquinoxalin-2-yl)pyrimidine-2,4-diamine (10).

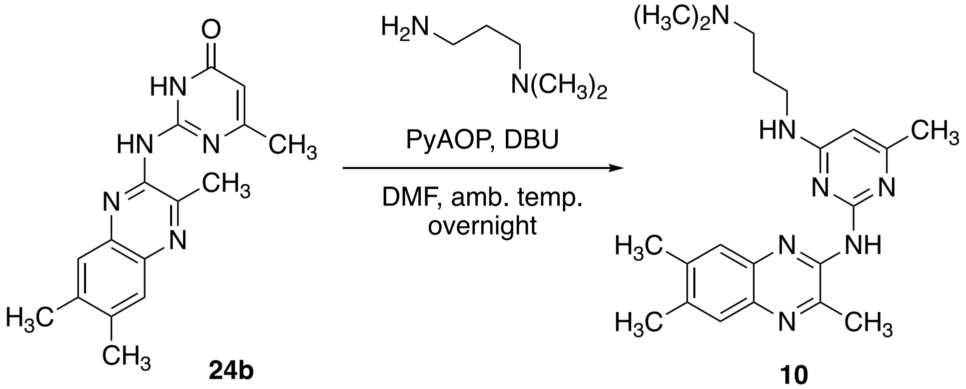

24b **10**

A vial was charged with **24b** (74 mg, 0.25 mmol) and PyAOP (169 mg, 0.322 mmol). Then, DMF (1.5 mL) and DBU (56 µL, 0.38 mmol) were added and the resulting solution was stirred at ambient temperature for 1 h. Propylamine (79 µL, 0.63 mmol) was then added and the solution was stirred at ambient temperature overnight. The solvent was removed under reduced pressure and the mixture was diluted with water. The AQ layer was then extracted with $CHCl_3$:IPA (3:1, 15 mL × 3) and the combined org. layer was dried over Na_2SO_4, filtered, and concentrated under reduced pressure. Purification by flash column chromatography (eluent: 10% → 20% MeOH (1% NH_4OH) in CH_2Cl_2) afforded **10** (40 mg, 42%) as a dark-yellow solid. ^{1}H NMR (600 MHz, DMSO-d_6) δ (ppm) 9.10 (s, 1H), 7.69 (s, 1H), 7.59 (s, 1H), 7.14 (s, 1H), 5.86 (s, 1H), 3.32 (s, 2H), 3.17 – 3.10 (m, 2H), 2.54 (s, 3H), 2.42

(d, J = 2.6 Hz, 6H), 2.26 (s, 6H), 2.10 (s, 3H), 1.67 – 1.59 (m, 2H). ^{13}C NMR (151 MHz, DMSO-d_6) δ 163.3, 160.0, 151.1, 147.8, 138.6, 138.0, 137.4, 126.9, 126.4, 97.0, 55.9, 44.0, 37.6, 25.9, 23.3, 22.0, 19.7, 19.7. HRMS: *m/z*: [M-H]$^+$ calcd for $C_{21}H_{30}N_7^+$ 380.2557; Found 380.2561.

N2-(6,7-dichloro-3-methylquinoxalin-2-yl)-N4-(3-(dimethylamino)propyl)-6-methylpyrimidine-2,4-diamine (11).

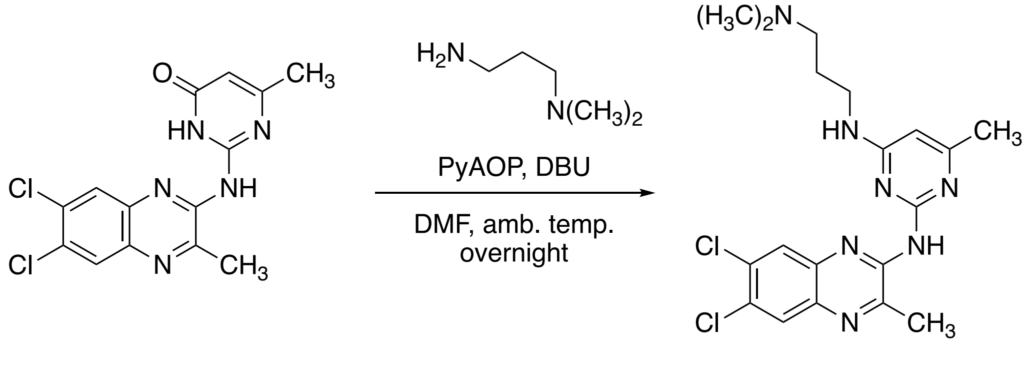

24c **11**

A vial was charged with **24c** (100 mg, 0.298 mmol) and PyAOP (202 mg, 0.387 mmol). Then, DMF (3 mL) and DBU (67 µL, 0.45 mmol) were added and the resulting solution was stirred at ambient temperature for 1 h. Propylamine (94 µL, 0.75 mmol) was then added and the solution was stirred at ambient temperature overnight. The solvent was removed under reduced pressure and the mixture was diluted with water. The AQ layer was then extracted with $CHCl_3$:IPA (3:1, 15 mL × 3) and the combined org. layer was dried over Na_2SO_4, filtered, and concentrated under reduced pressure. Purification by flash column chromatography (eluent: 8% → 12% MeOH (1% NH_4OH) in CH_2Cl_2) afforded **11** (44 mg, 35%) as a dark-brown solid. ^{1}H NMR (600 MHz, DMSO-d_6) δ (ppm) 9.40 (s, 1H), 8.15 (s, 1H), 8.01 (s, 1H), 7.25 (s, 1H), 5.93 (s, 1H), 3.23 – 3.10 (m, 2H), 2.59 (s, 3H), 2.24 (s, 2H), 2.16 (s, 3H), 2.11 (s, 6H), 1.63 (p, J = 7.1 Hz, 2H). ^{13}C NMR (151 MHz, DMSO-d_6) δ (ppm) 174.5, 163.2, 159.0, 153.0, 149.6, 139.1, 137.3, 131.1, 128.5, 127.6, 98.0, 56.5, 44.8, 44.7, 38.0, 26.5, 22.2. HRMS: *m/z*: [M-H]$^+$ calcd for $C_{19}H_{24}Cl_2N_7^+$ 420.1465; Found 420.1460.

N-(4,6-dimethylpyrimidin-2-yl)-4-methylquinazolin-2-amine (25).

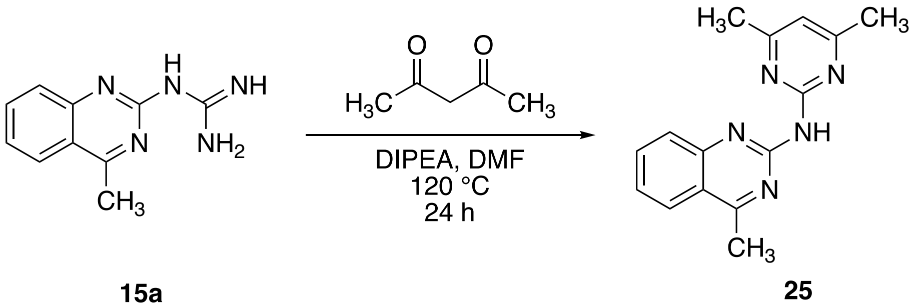

15a **25**

A vial was charged with **15a** (150 mg, 0.866 mmol). Then, DMF (3 mL), DIPEA (302 µL, 1.74 mmol) and acetylacetone (885 µL, 8.66 mmol) were added sequentially and the solution was stirred at 120 °C for 24 h. The solution was concentrated under reduced pressure and then diluted with water. The precipitate was collected with suction filtration. The solids were washed with more water and Et_2O to afford **25** (71 mg, 31%) as a beige solid. ^{1}H NMR (400 MHz, DMSO-d_6) δ (ppm) 9.92 (s, 1H), 8.12 (d, J = 8.2 Hz, 1H), 7.81 (t, J = 7.7 Hz, 1H), 7.66 (d, J = 8.4 Hz, 1H), 7.46 (t, J = 7.5 Hz, 1H), 6.82 (s, 1H), 2.82 (s, 3H), 2.34 (s, 6H). ^{13}C NMR (151 MHz, DMSO-d_6) δ (ppm) 169.9, 167.6, 159.3, 155.8, 151.1, 134.3, 127.1, 126.2, 124.9, 121.0, 114.2, 40.4, 23.9, 21.8. HRMS: *m/z*: [M-H]$^+$ calcd for $C_{15}H_{16}N_5^+$ 266.1400; Found 266.1402.

N-(4,6-dimethylpyrimidin-2-yl)-4,6-dimethylquinazolin-2-amine (26).

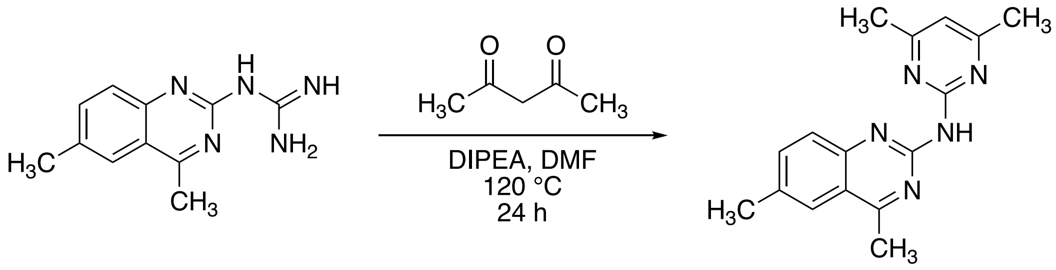

15b **26**

A vial was charged with **15b** (200 mg, 0.929 mmol). Then, DMF (4 mL), DIPEA (324 µL, 1.86 mmol) and acetylacetone (949 µL, 9.29 mmol) were added sequentially and the solution was stirred at 120 °C for 24 h. The solution was concentrated under reduced pressure and then diluted with water. The precipitate was collected with suction filtration. The solids were washed with more water and Et_2O to afford **26** (119 mg, 46%) as a beige solid. ^1H NMR (400 MHz, DMSO-d_6) δ (ppm) 9.84 (s, 1H), 7.90 (s, 1H), 7.66 (dd, J = 8.6, 1.9 Hz, 1H), 7.58 (d, J = 8.5 Hz, 1H), 6.79 (s, 1H), 2.79 (s, 3H), 2.48 (s, 3H), 2.33 (s, 6H). ^{13}C NMR (151 MHz, DMSO-d_6) δ (ppm) 168.6, 167.0, 158.9, 154.8, 149.0, 135.8, 133.8, 126.5, 124.4, 120.5, 113.5, 23.5, 21.3, 21.0. HRMS: m/z: $[M-H]^+$ calcd for $C_{16}H_{18}N_5^+$ 280.1557; Found 280.1551.

N-(4,6-dimethylpyrimidin-2-yl)-4,6,8-trimethylquinazolin-2-amine (27).

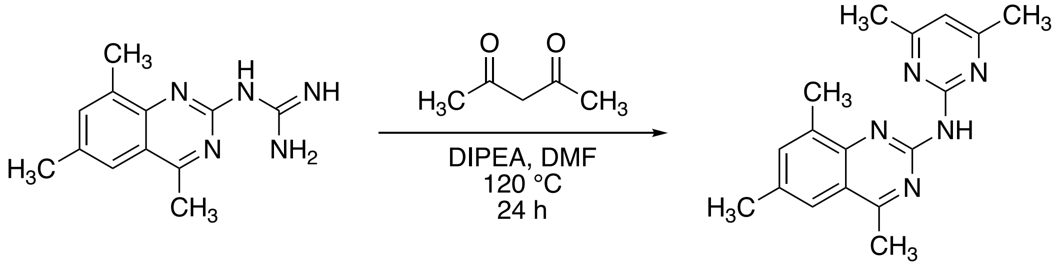

15c **27**

A vial was charged with **15c** (200 mg, 0.872 mmol). Then, DMF (4 mL), DIPEA (304 µL, 1.75 mmol) and acetylacetone (891 µL, 8.72 mmol) were added sequentially and the solution was stirred at 120 °C for 24 h. The solution was concentrated under reduced pressure and then diluted with water. The precipitate was collected with suction filtration. The solids were washed with more water and Et_2O to afford **27** (124 mg, 49%) as a beige solid. ^1H NMR (400 MHz, DMSO-d_6) δ (ppm) 9.80 (s, 1H), 7.72 (s, 1H), 7.52 (s, 1H), 6.79 (s, 1H), 2.78 (s, 3H), 2.56 (s, 3H), 2.44 (s, 3H), 2.34 (s, 6H). ^{13}C NMR (151 MHz, DMSO-d_6) δ (ppm) 168.5, 166.9, 158.9, 153.8, 148.0, 135.4, 134.5, 133.0, 121.9, 120.3, 113.3, 23.5, 21.4, 21.1, 16.6. HRMS: m/z: $[M-H]^+$ calcd for $C_{17}H_{20}N_5^+$ 294.1713; Found 294.1711.

N-(4,6-dimethylpyrimidin-2-yl)-6-methoxy-4-methylquinazolin-2-amine (28).

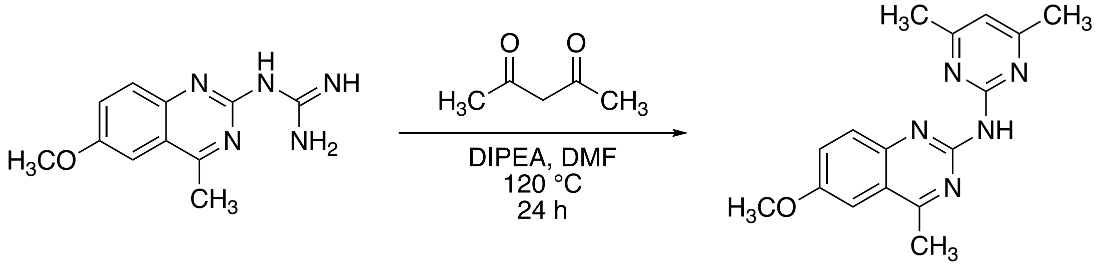

15d **28**

A vial was charged with **15d** (200 mg, 0.865 mmol). Then, DMF (4 mL), DIPEA (301 µL, 1.73 mmol) and acetylacetone (884 µL, 8.65 mmol) were added sequentially and the solution was stirred at 120 °C for 24 h. The solution was concentrated under reduced pressure and then diluted with water. The precipitate was collected with suction filtration. The solids were washed with more water and Et_2O to afford **28** (89 mg, 35%) as a beige solid. ^1H NMR (400 MHz, DMSO-d_6) δ (ppm) 9.77 (s, 1H), 7.63 (d, J = 9.1 Hz, 1H), 7.49 (dd, J = 9.1, 2.8 Hz, 1H), 7.40 (d, J = 2.8 Hz, 1H), 6.77 (s, 1H), 3.92 (s, 3H), 2.81 (s, 3H), 2.32 (s, 6H). ^{13}C NMR (151 MHz, DMSO-d_6) δ (ppm) 167.8, 167.0, 159.1, 155.8, 154.0, 146.3, 128.3, 125.8, 121.1, 113.3, 103.9, 55.6, 23.5, 21.5. HRMS: *m/z*: $[M-H]^+$ calcd for $C_{16}H_{18}N_5O^+$ 296.1506; Found 296.1509.

6-chloro-N-(4,6-dimethylpyrimidin-2-yl)-4-methylquinazolin-2-amine (29).

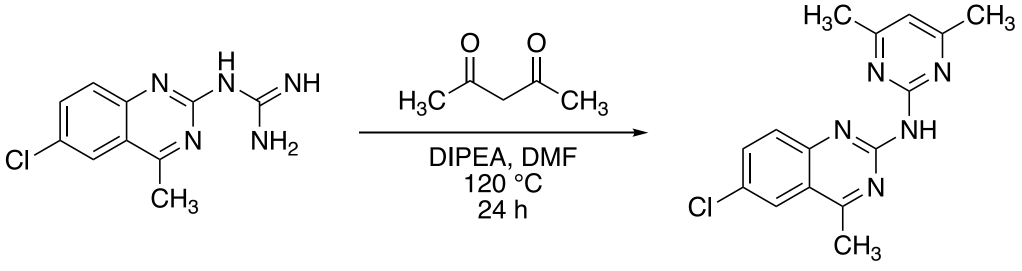

15e **29**

A vial was charged with **15e** (200 mg, 0.849 mmol). Then, DMF (4 mL), DIPEA (296 µL, 1.70 mmol) and acetylacetone (867 µL, 8.49 mmol) were added sequentially and the solution was stirred at 120 °C for 24 h. The solution was concentrated under reduced pressure and then diluted with water. The precipitate was collected with suction filtration. The solids were washed with more water and Et_2O to afford **29** (146 mg, 57%) as a beige solid. ^1H NMR (400 MHz, DMSO-d_6) δ (ppm) 10.08 (s, 1H), 8.18 (d, J = 2.4 Hz, 1H), 7.81 (dd, J = 8.9, 2.4 Hz, 1H), 7.66 (d, J = 9.0 Hz, 1H), 6.83 (s, 1H), 2.81 (s, 3H), 2.34 (s, 6H). ^{13}C NMR (151 MHz, DMSO-d_6) δ (ppm) 169.2, 167.2, 158.6, 155.6, 149.3, 134.2, 128.8, 128.2, 124.8, 121.1, 114.0, 23.5, 21.4. HRMS: *m/z*: $[M-H]^+$ calcd for $C_{15}H_{15}ClN_5^+$ 300.1010; Found 300.1008.

NMR spectra of Synthesized compounds

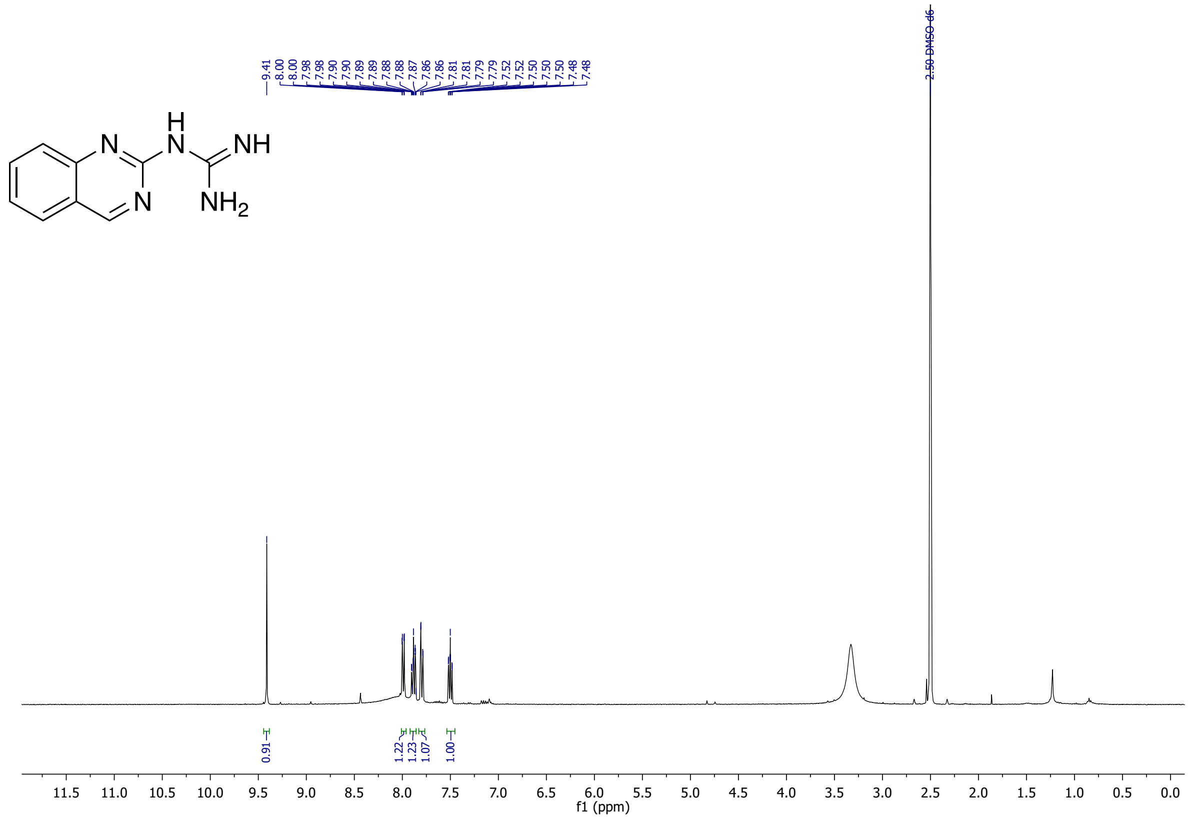

^{1}H NMR spectrum of **12** in DMSO-d_6 measured at 400 MHz.

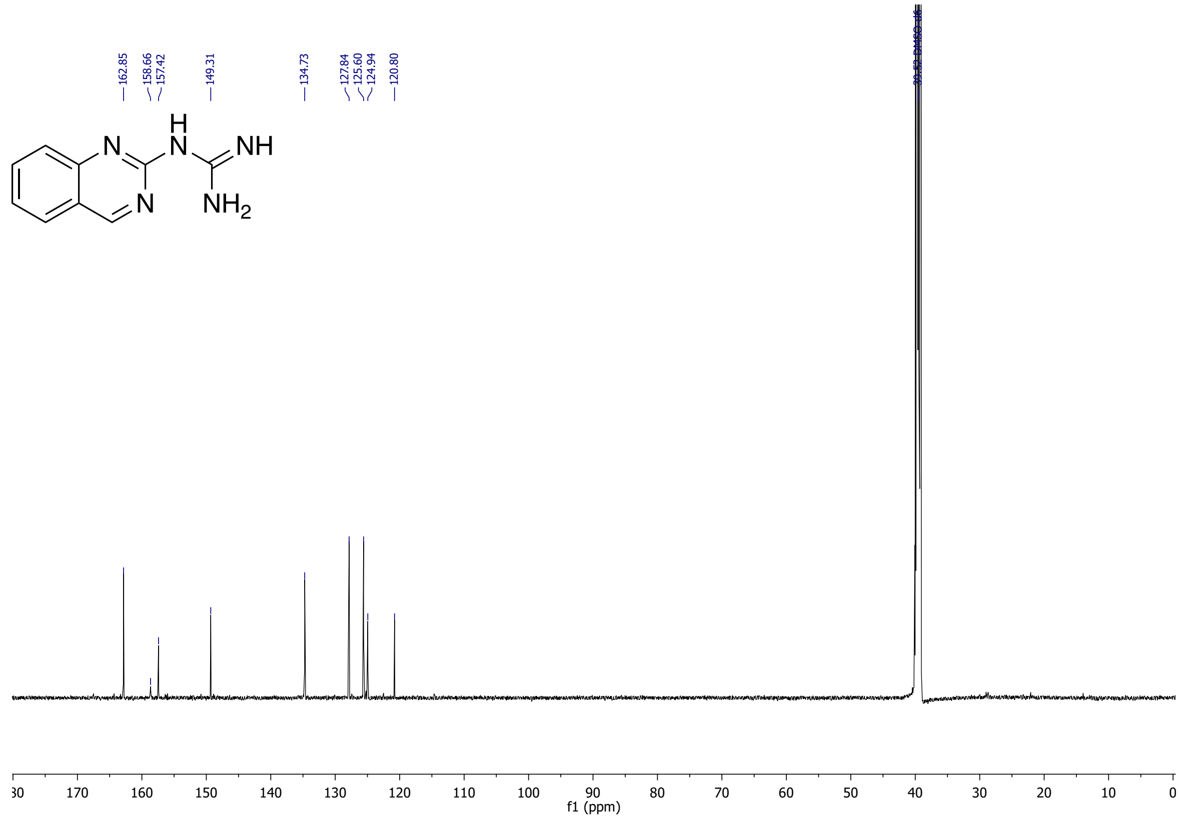

^{13}C NMR spectrum of **12** in DMSO-d_6 measured at 151 MHz.

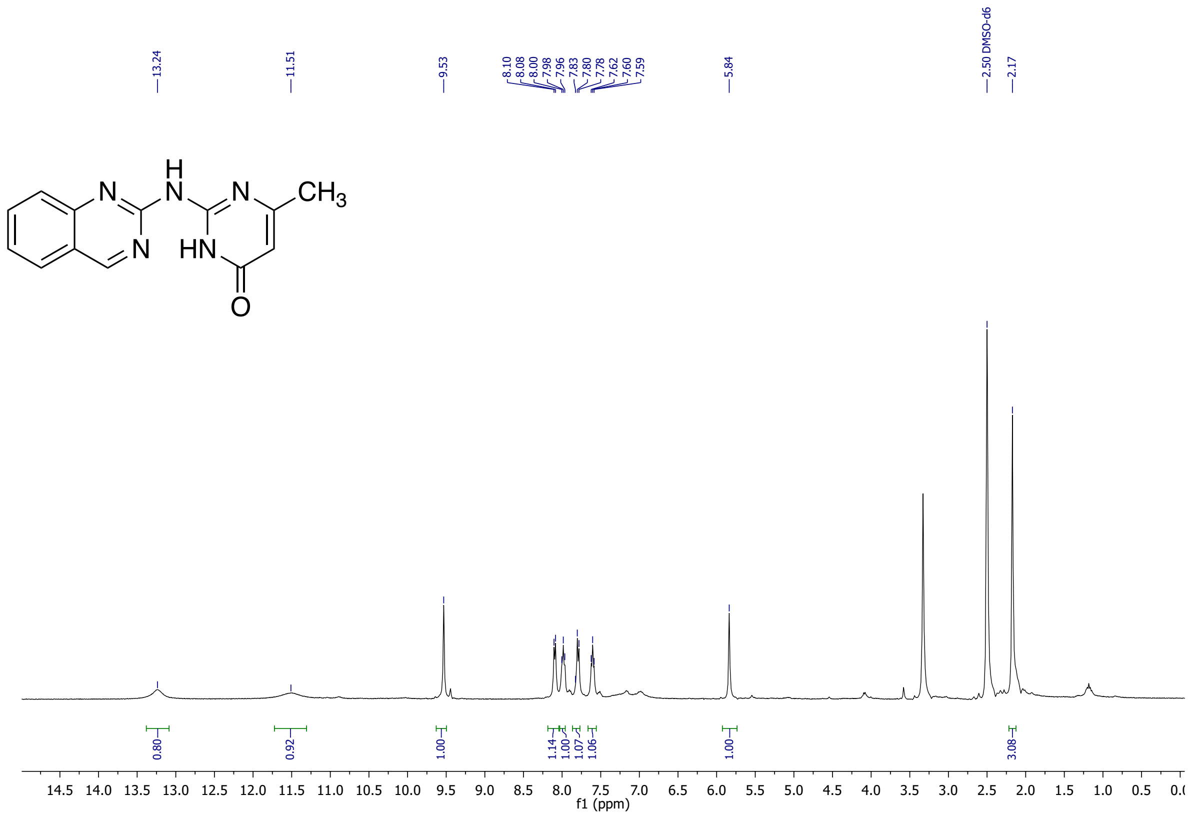

^{1}H NMR spectrum of **13** in DMSO-d_6 measured at 400 MHz.

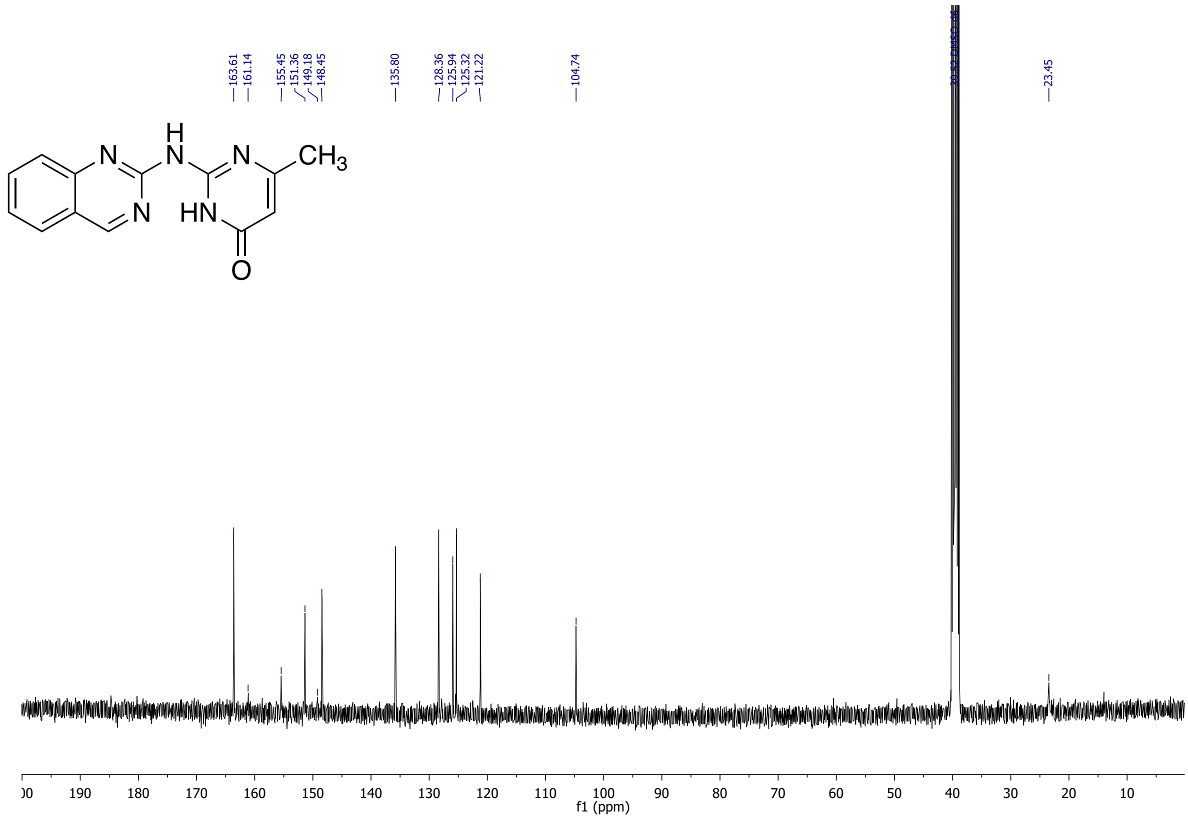

^{13}C NMR spectrum of **13** in DMSO-d_6 measured at 100 MHz.

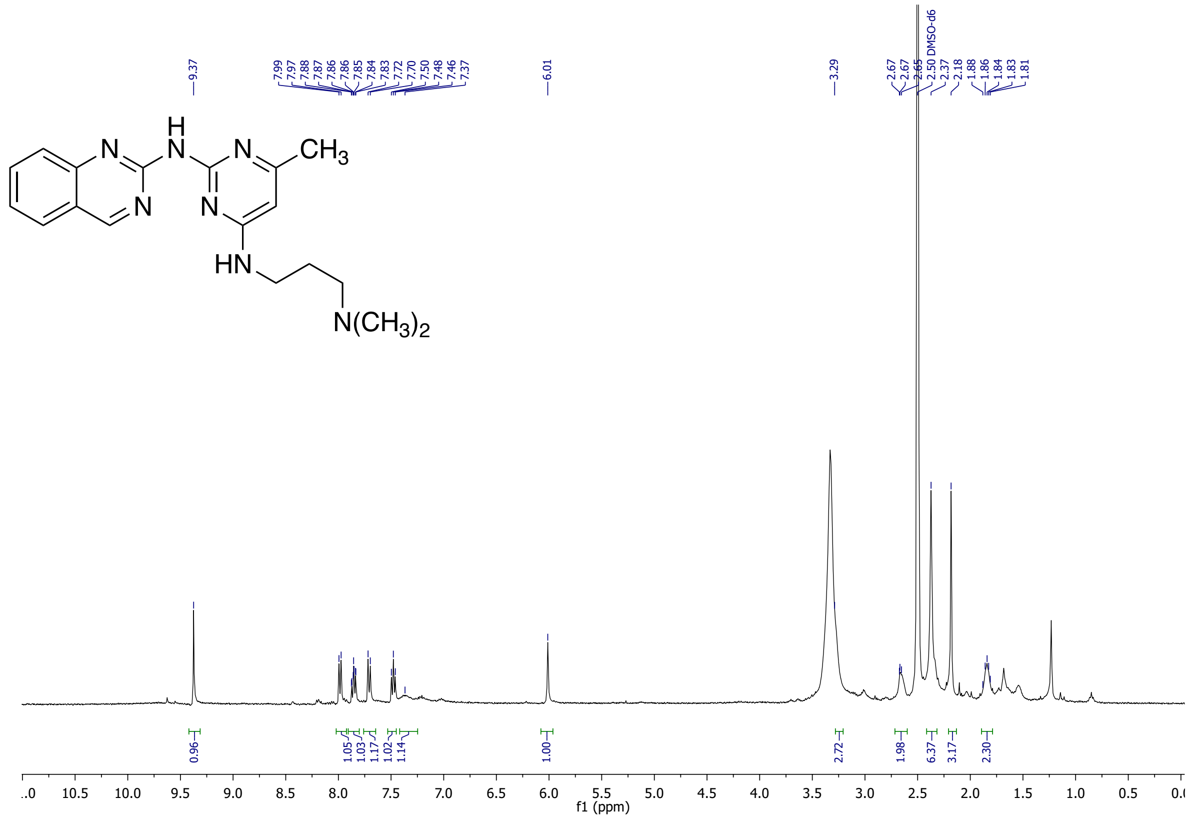

^{1}H NMR spectrum of **2** in DMSO-d_6 measured at 400 MHz.

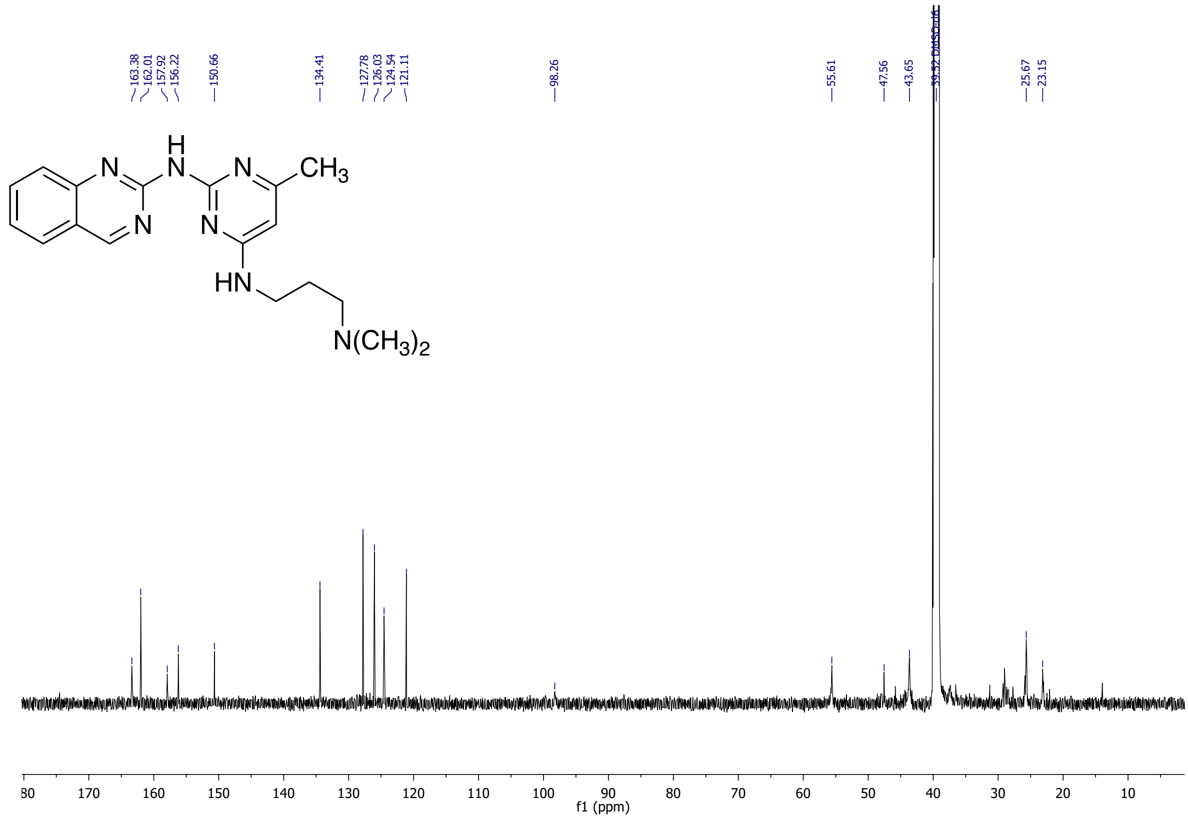

^{13}C NMR spectrum of **2** in DMSO-d_6 measured at 151 MHz.

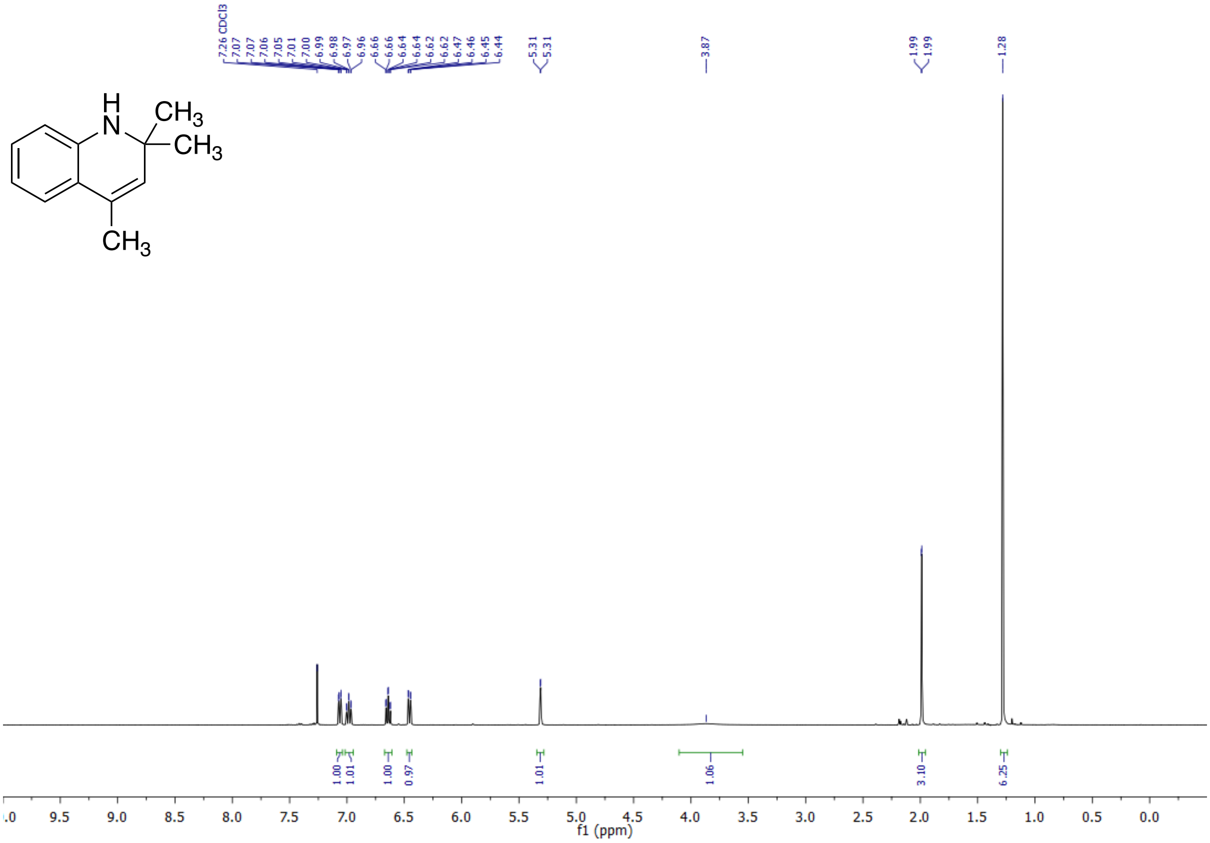

^{1}H NMR spectrum of **14a** in $CDCl_3$ measured at 400 MHz.

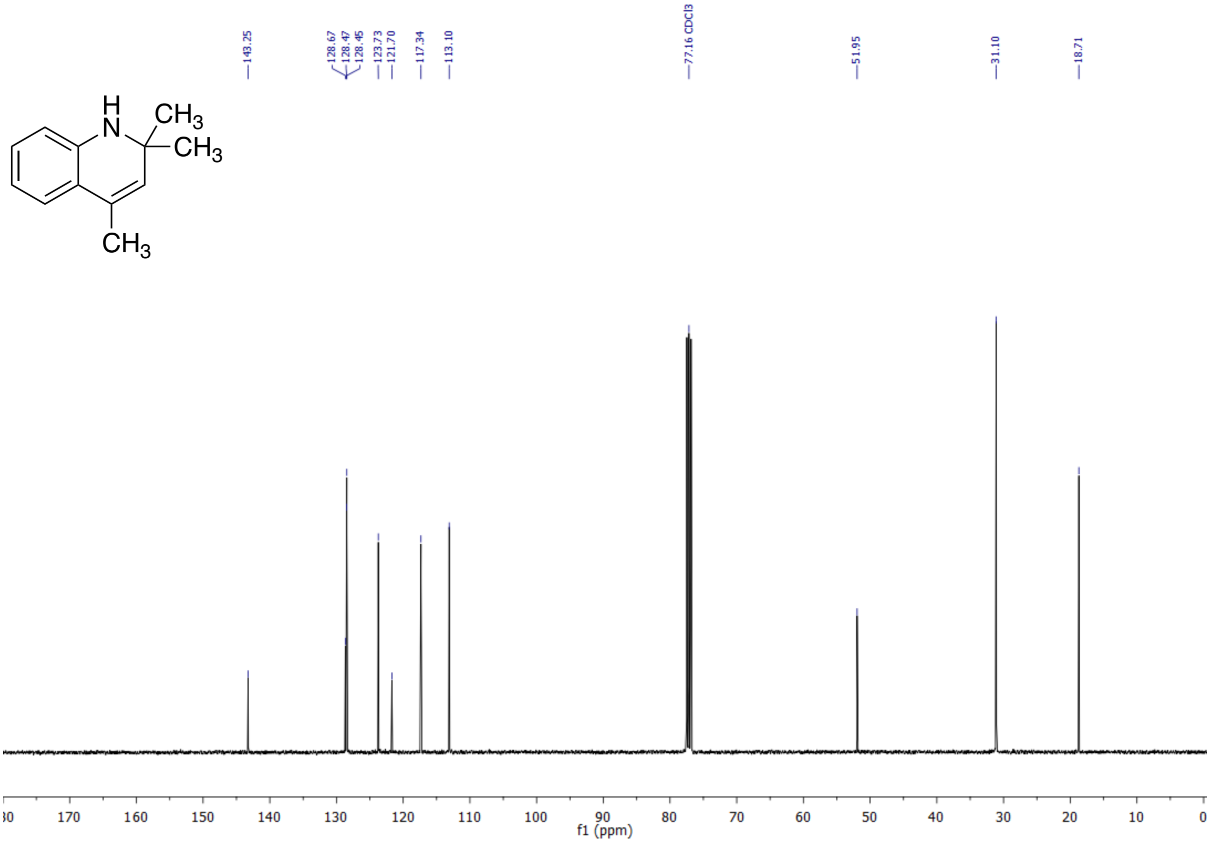

^{13}C NMR spectrum of **14a** in $CDCl_3$ measured at 100 MHz.

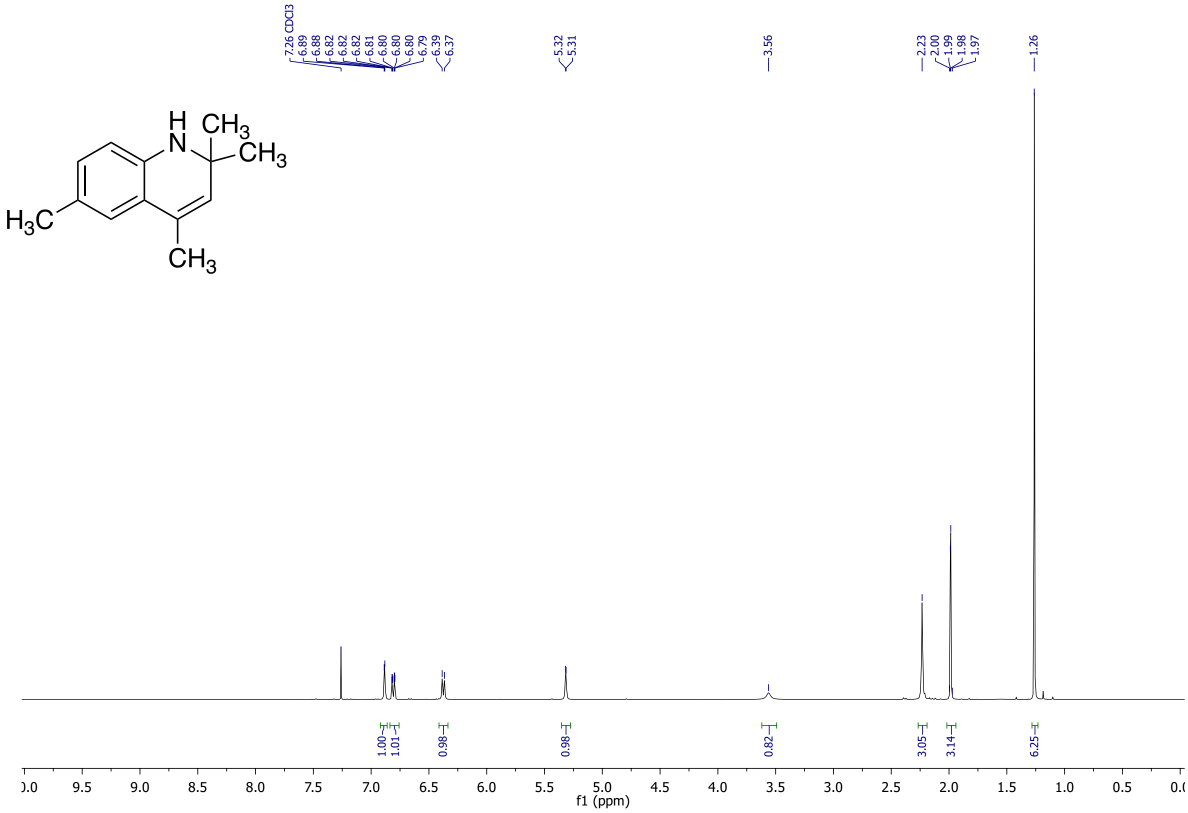

^{1}H NMR spectrum of **14b** in $CDCl_3$ measured at 400 MHz.

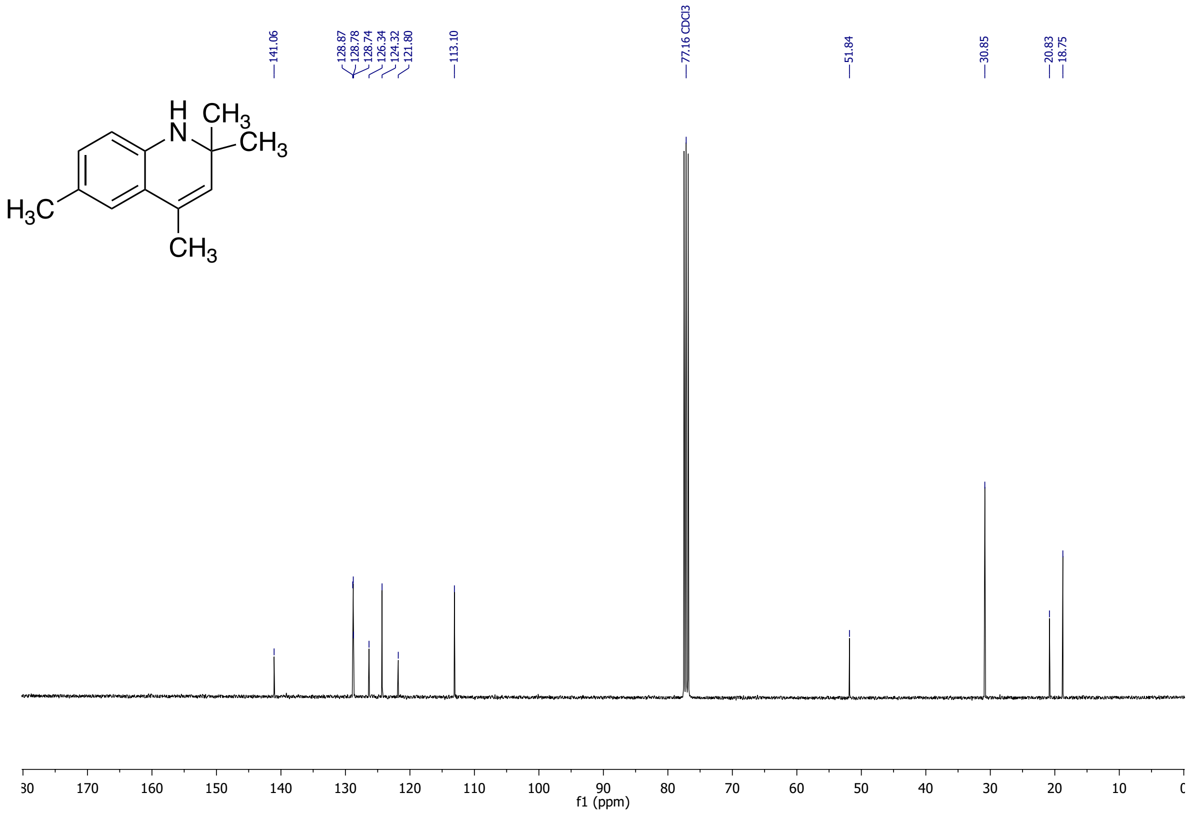

^{13}C NMR spectrum of **14b** in $CDCl_3$ measured at 100 MHz.

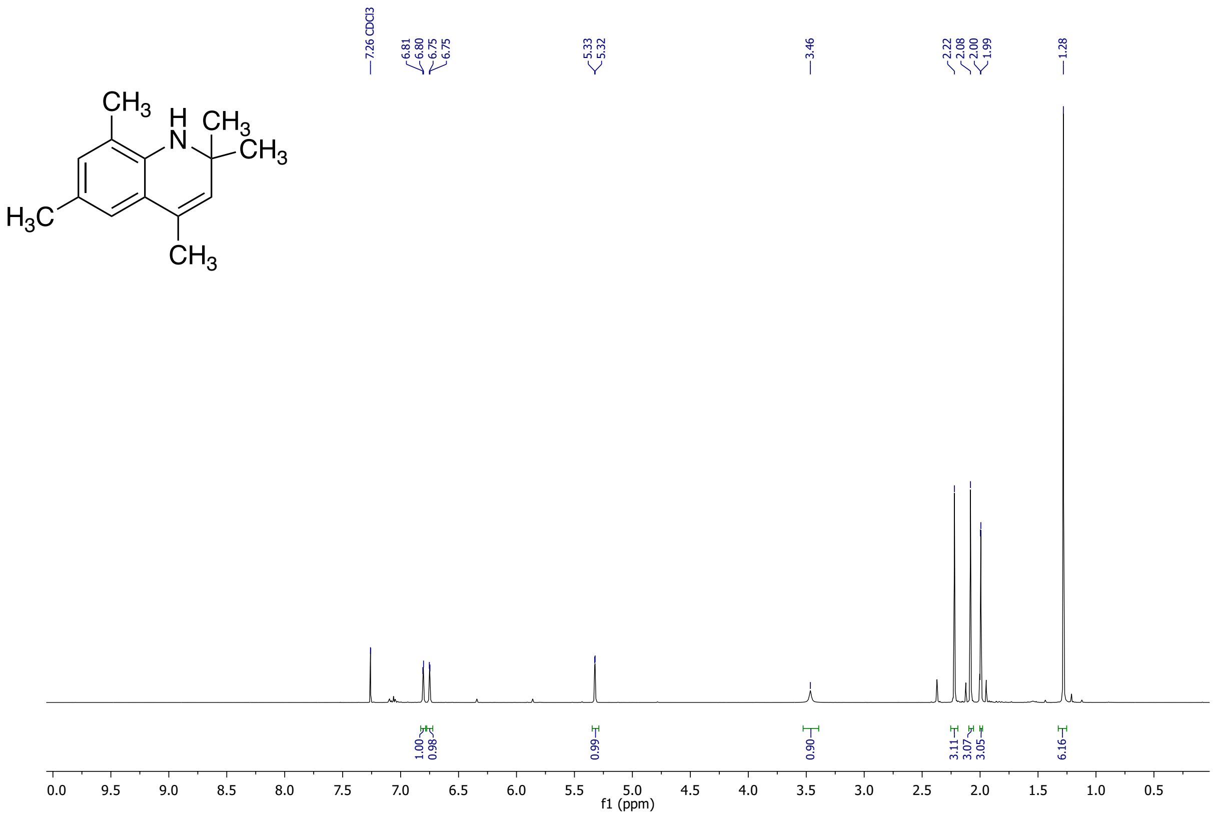

^{1}H NMR spectrum of **14c** in $CDCl_3$ measured at 400 MHz.

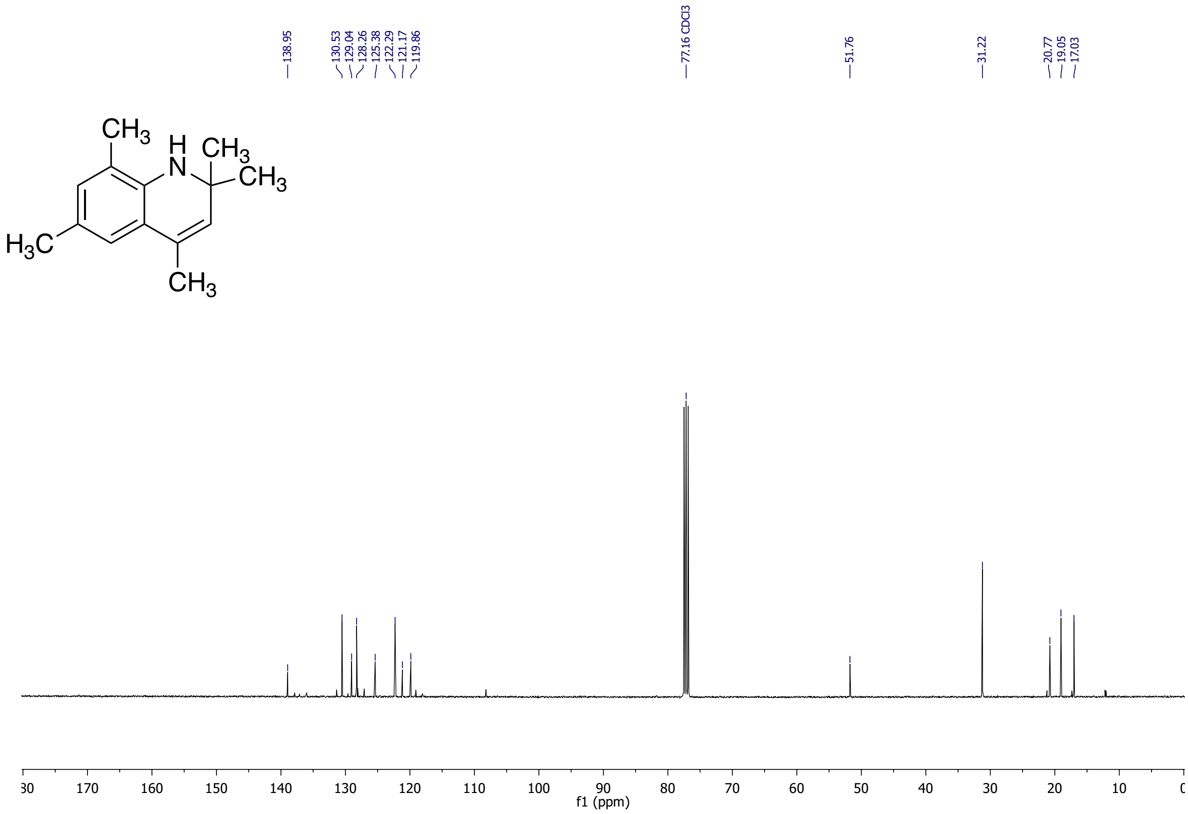

^{13}C NMR spectrum of **14c** in $CDCl_3$ measured at 100 MHz.

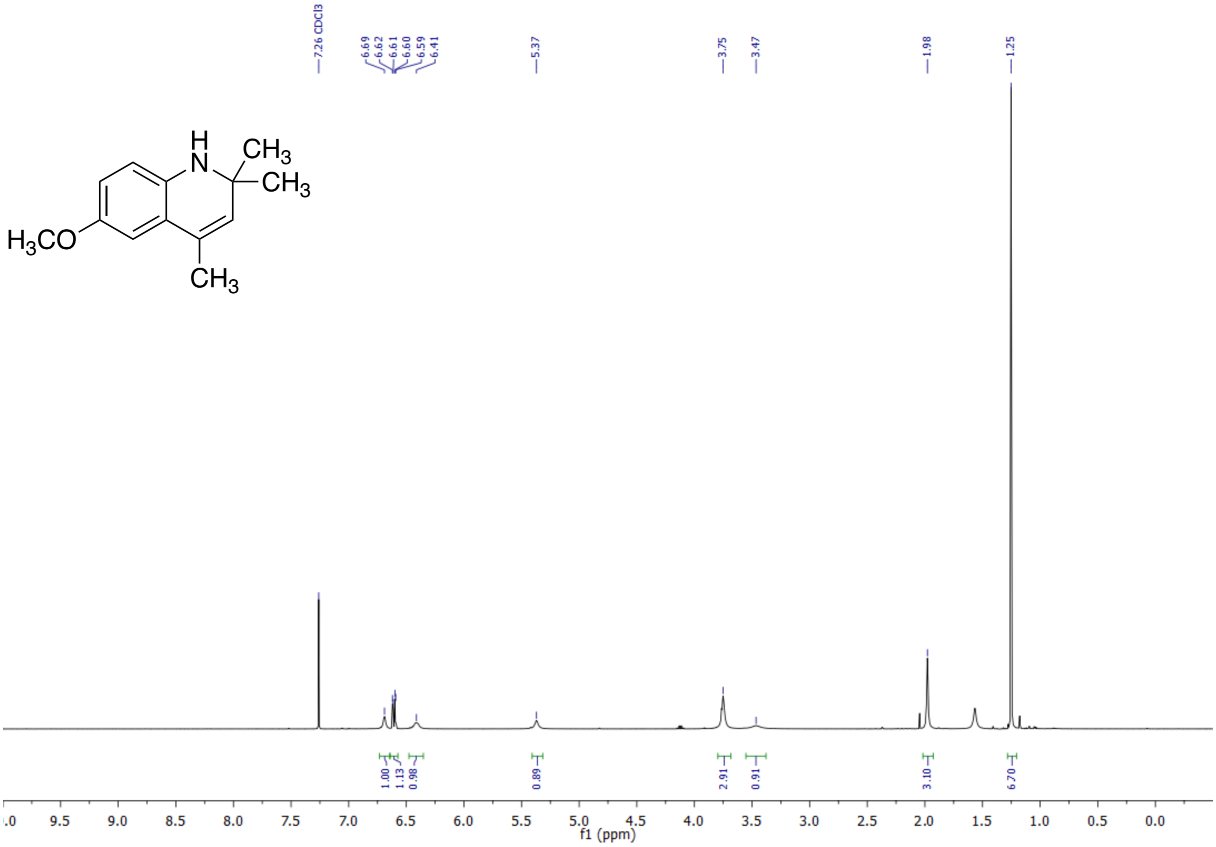

^{1}H NMR spectrum of **14d** in $CDCl_3$ measured at 400 MHz.

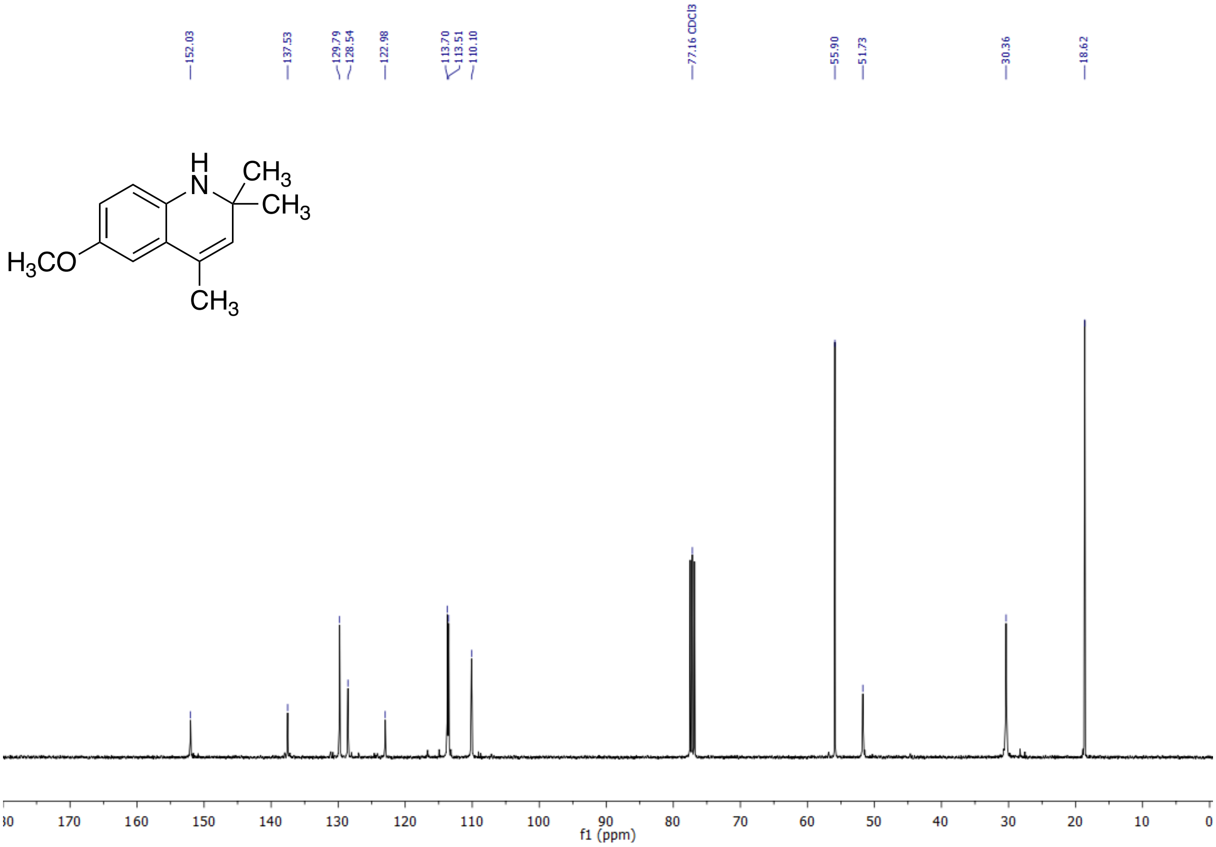

^{13}C NMR spectrum of **14d** in $CDCl_3$ measured at 100 MHz.

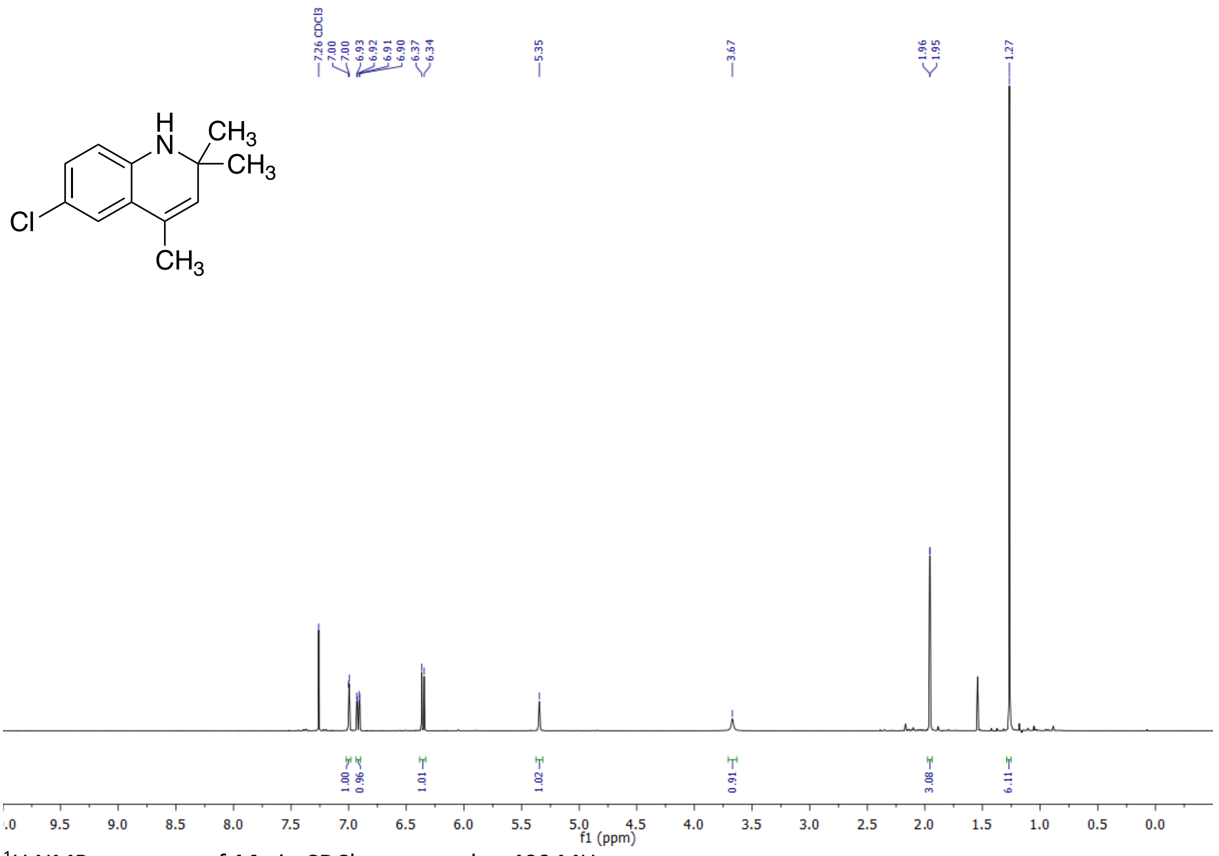

^{1}H NMR spectrum of **14e** in $CDCl_3$ measured at 400 MHz.

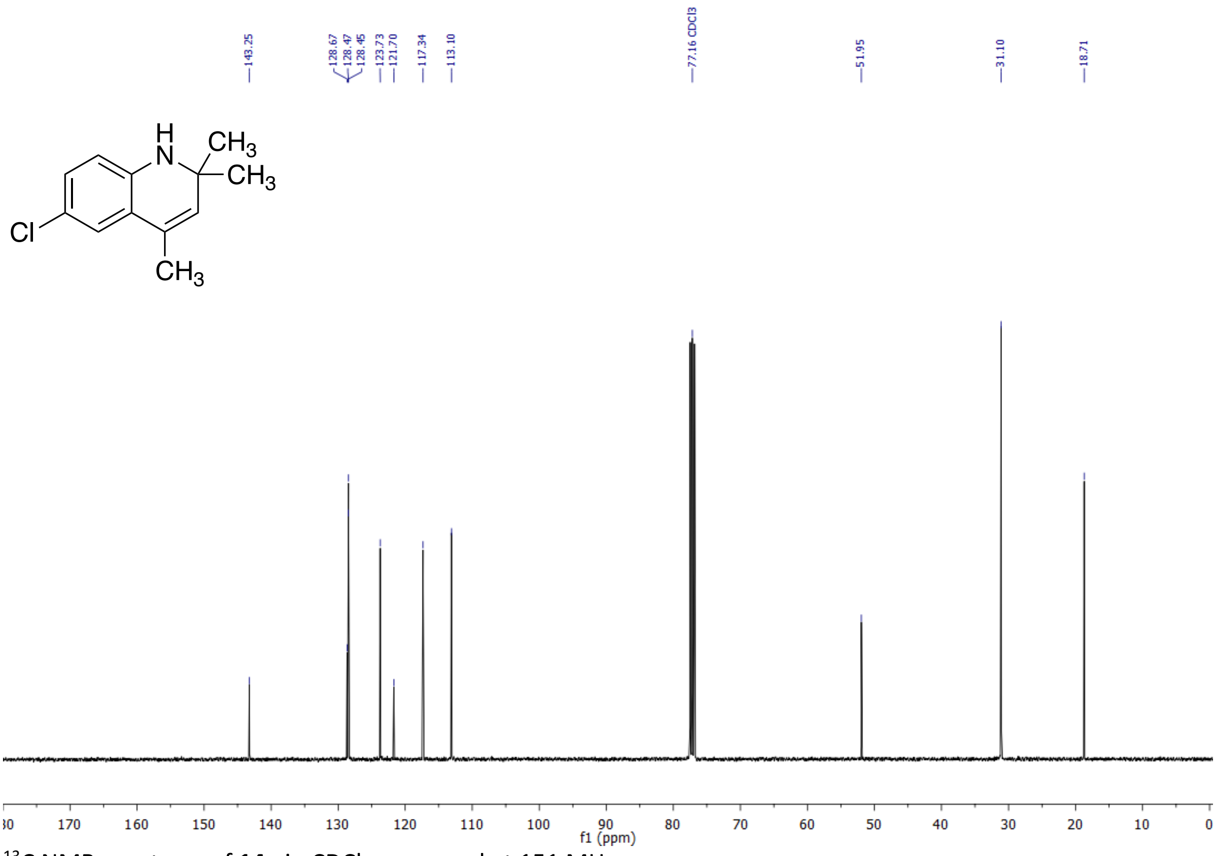

^{13}C NMR spectrum of **14e** in $CDCl_3$ measured at 151 MHz.

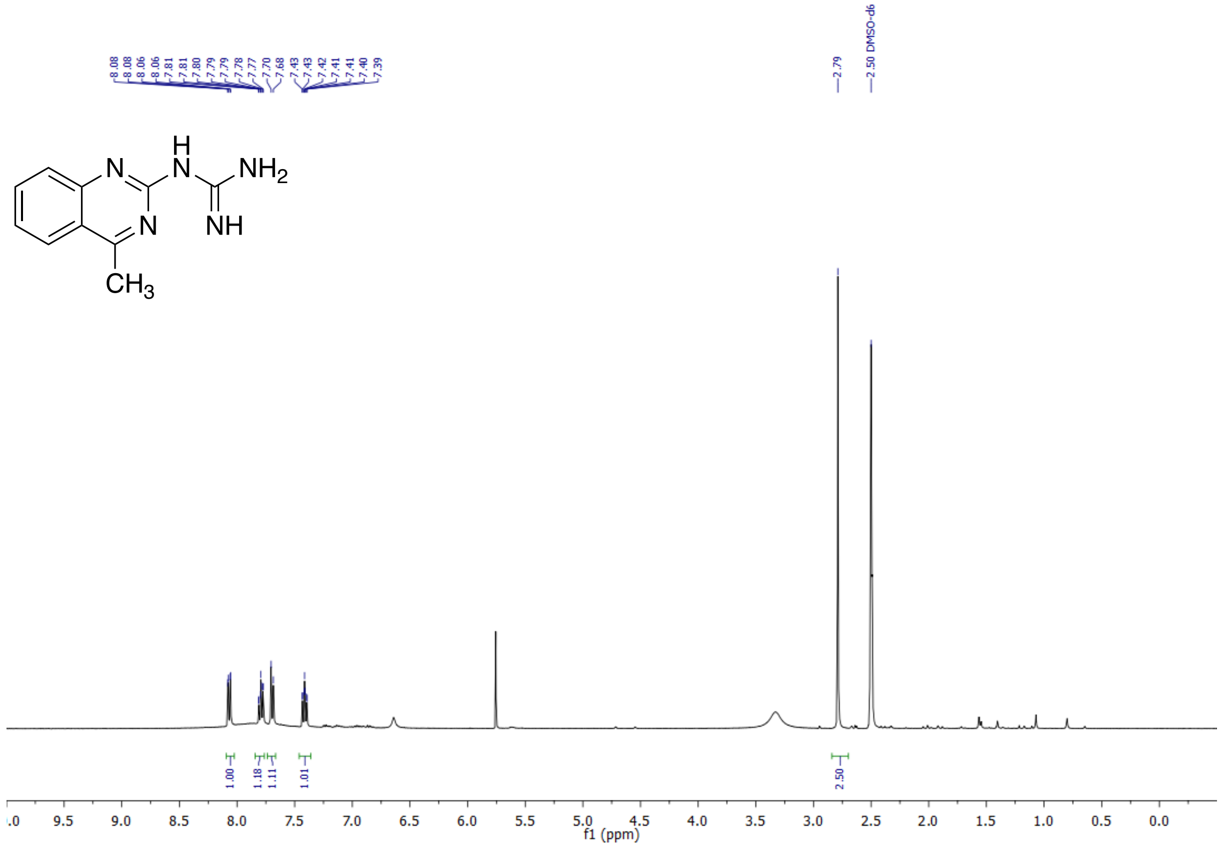

^{1}H NMR spectrum of **15a** in DMSO-d_6 measured at 400 MHz.

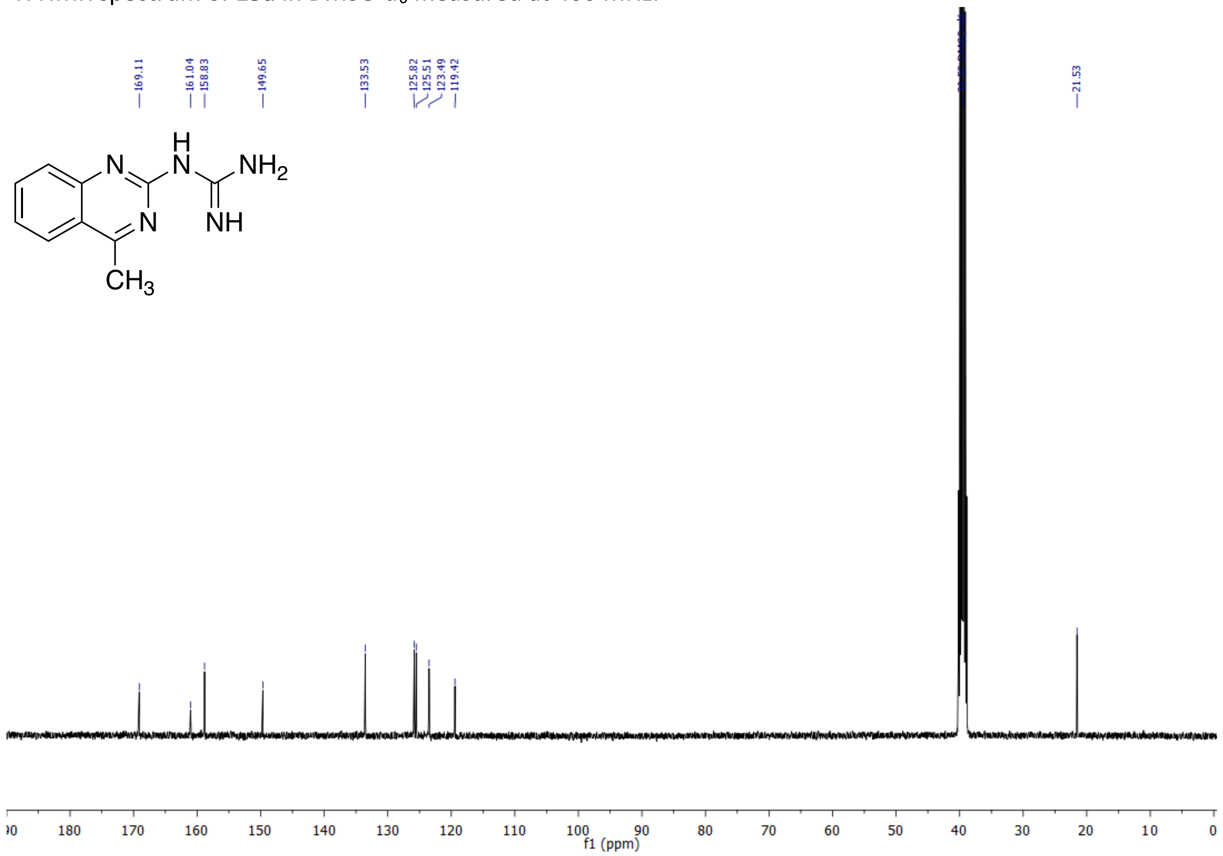

^{13}C NMR spectrum of **15a** in DMSO-d_6 measured at 100 MHz.

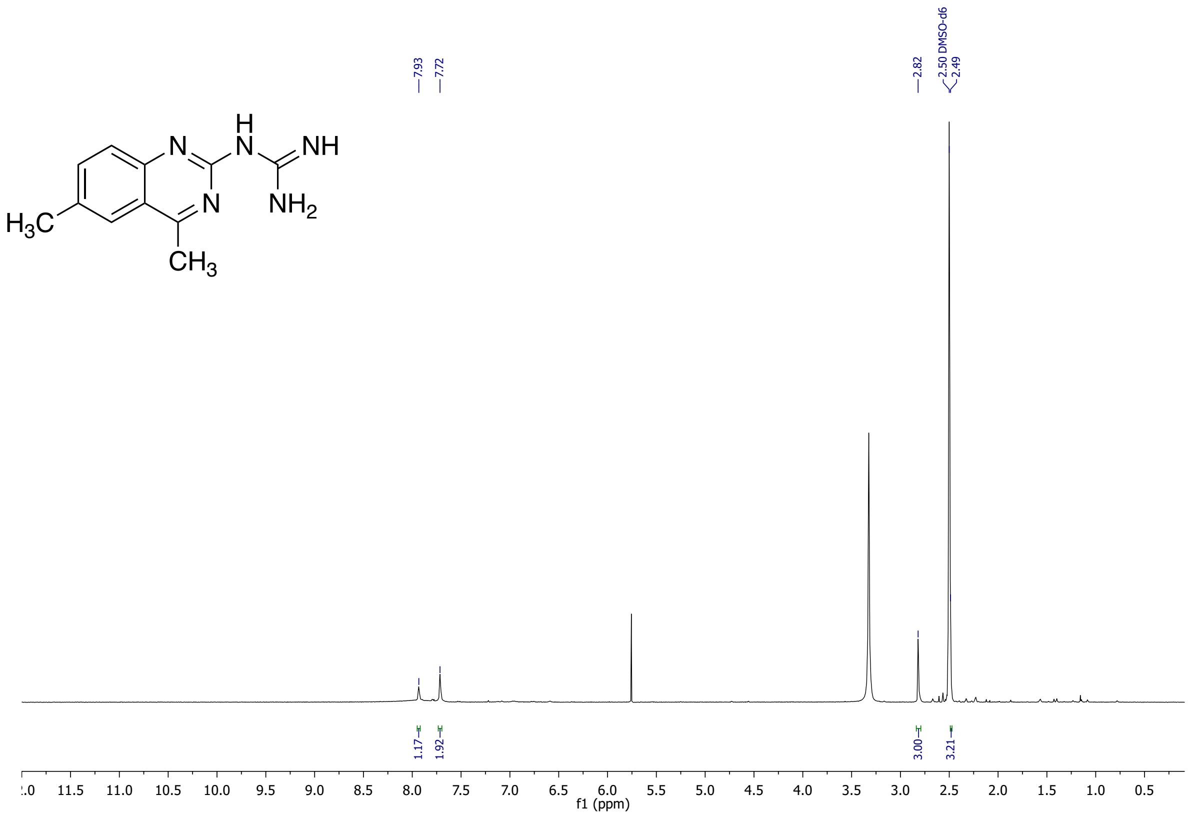

^{1}H NMR spectrum of **15b** in DMSO-d_6 measured at 400 MHz.

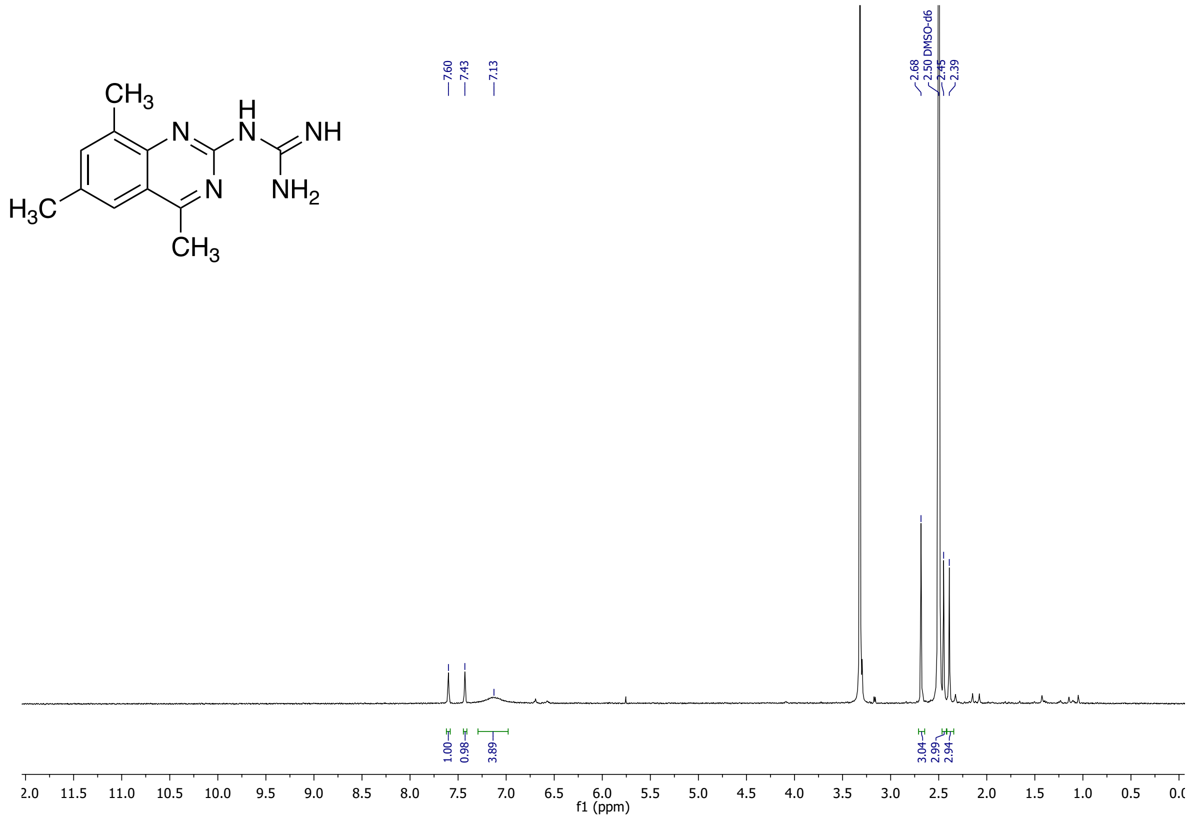

^{1}H NMR spectrum of **15c** in DMSO-d_6 measured at 400 MHz.

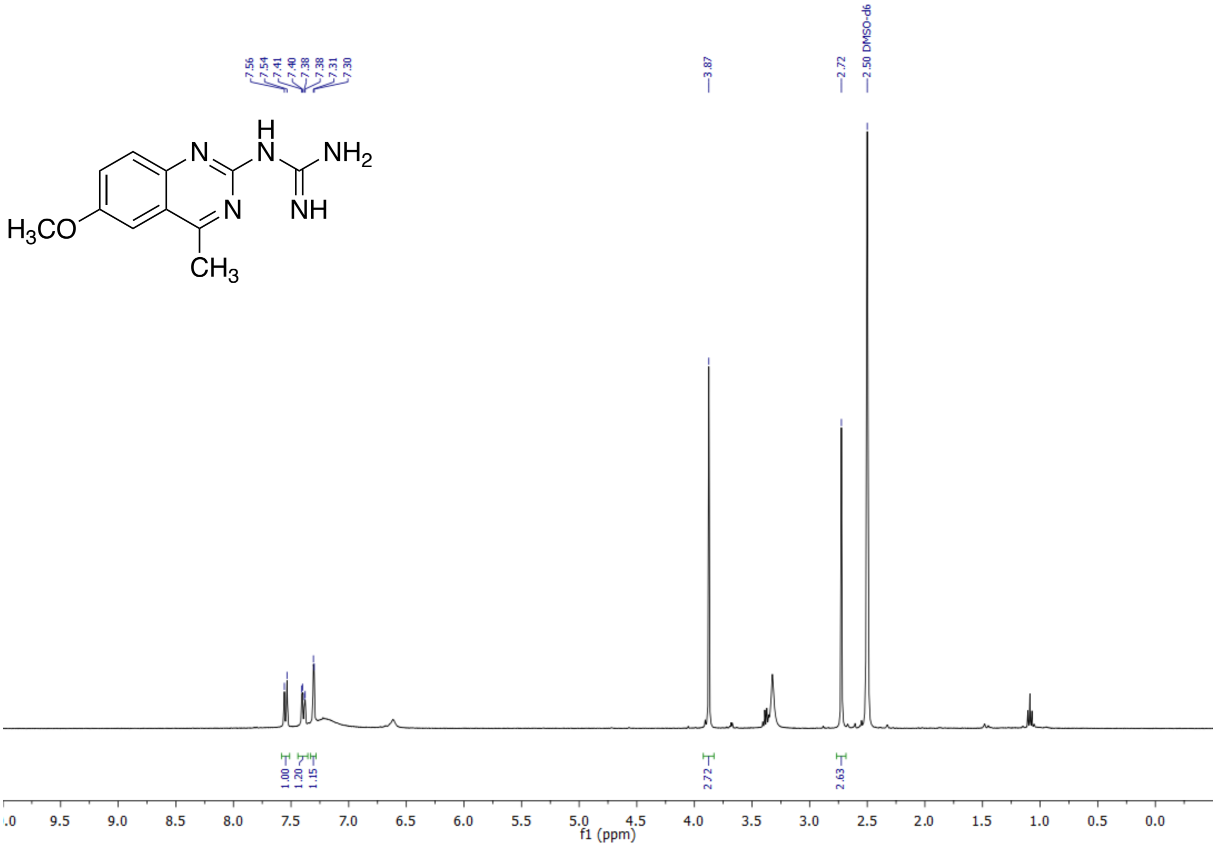

^{1}H NMR spectrum of **15d** in DMSO-d_6 measured at 400 MHz.

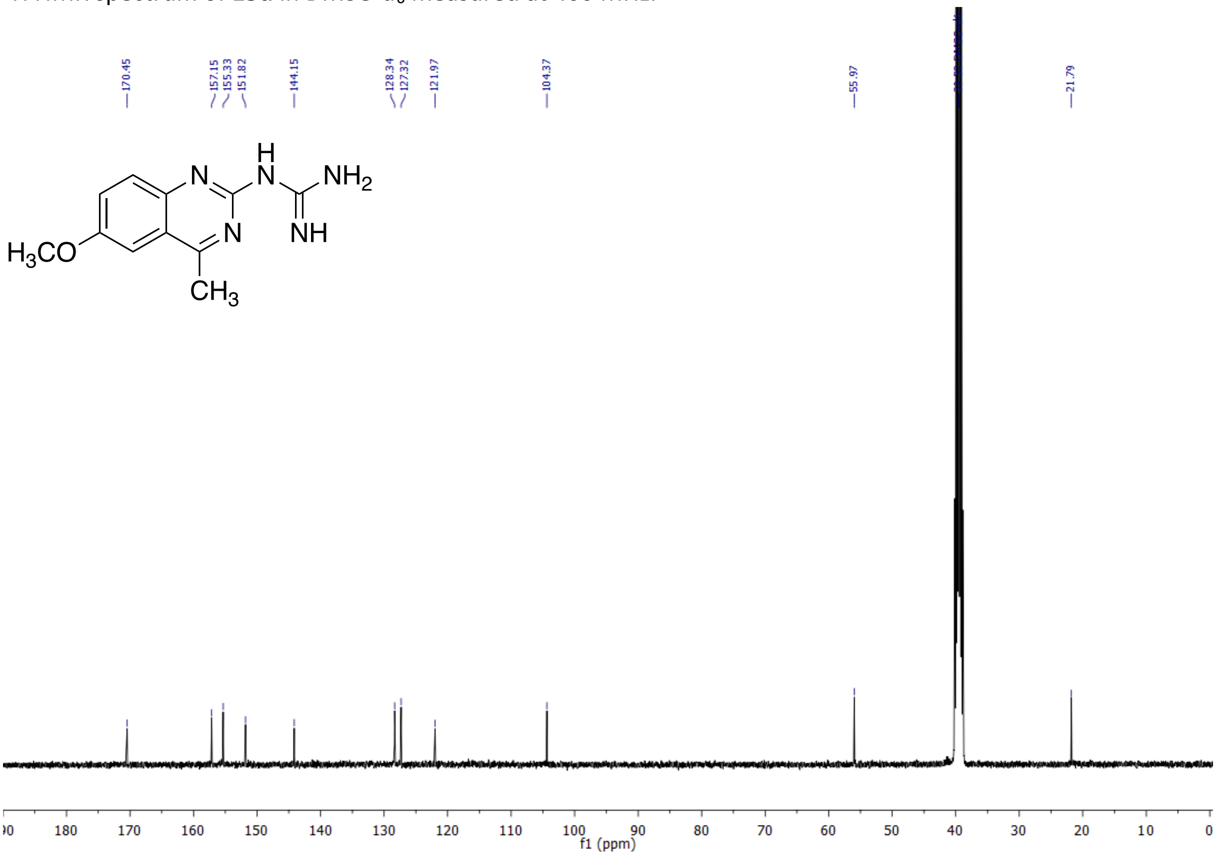

^{13}C NMR spectrum of **15d** in DMSO-d_6 measured at 100 MHz.

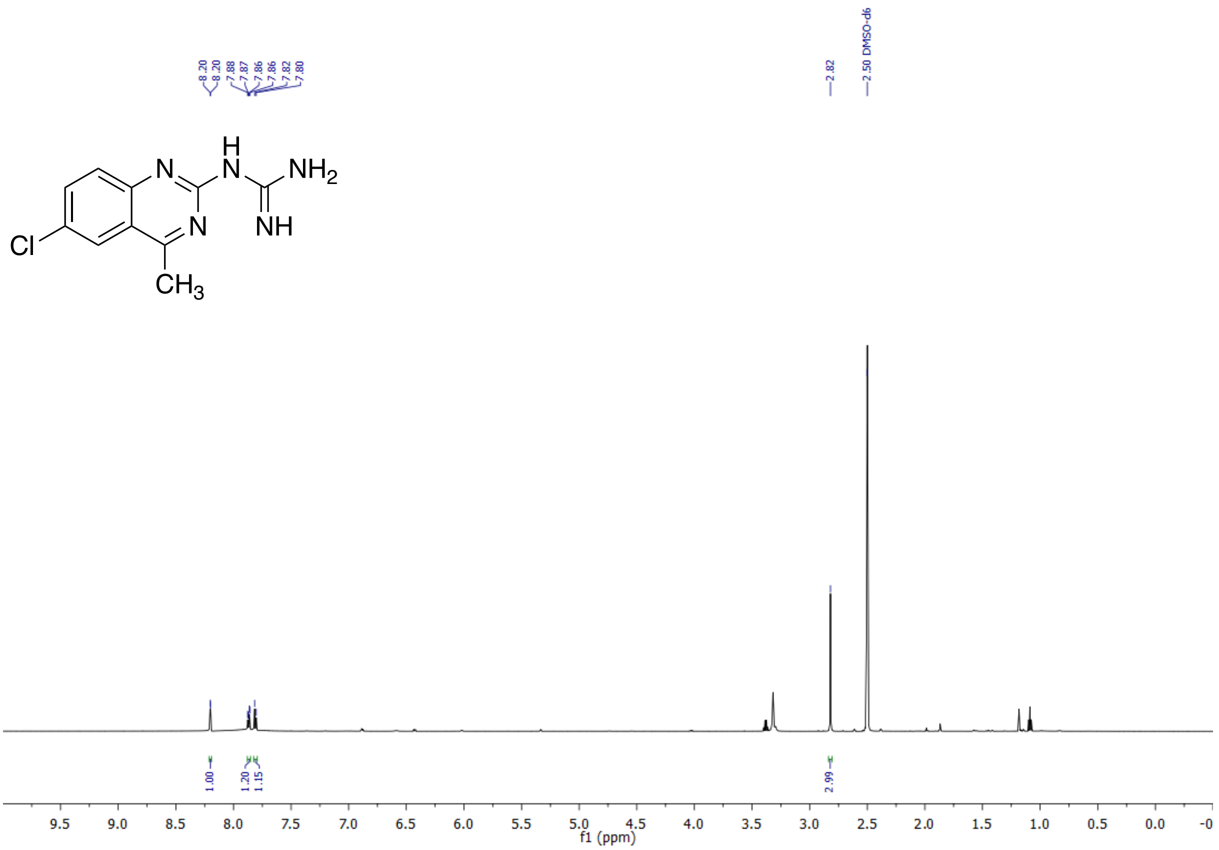

^{1}H NMR spectrum of **15e** in DMSO-d_6 measured at 600 MHz.

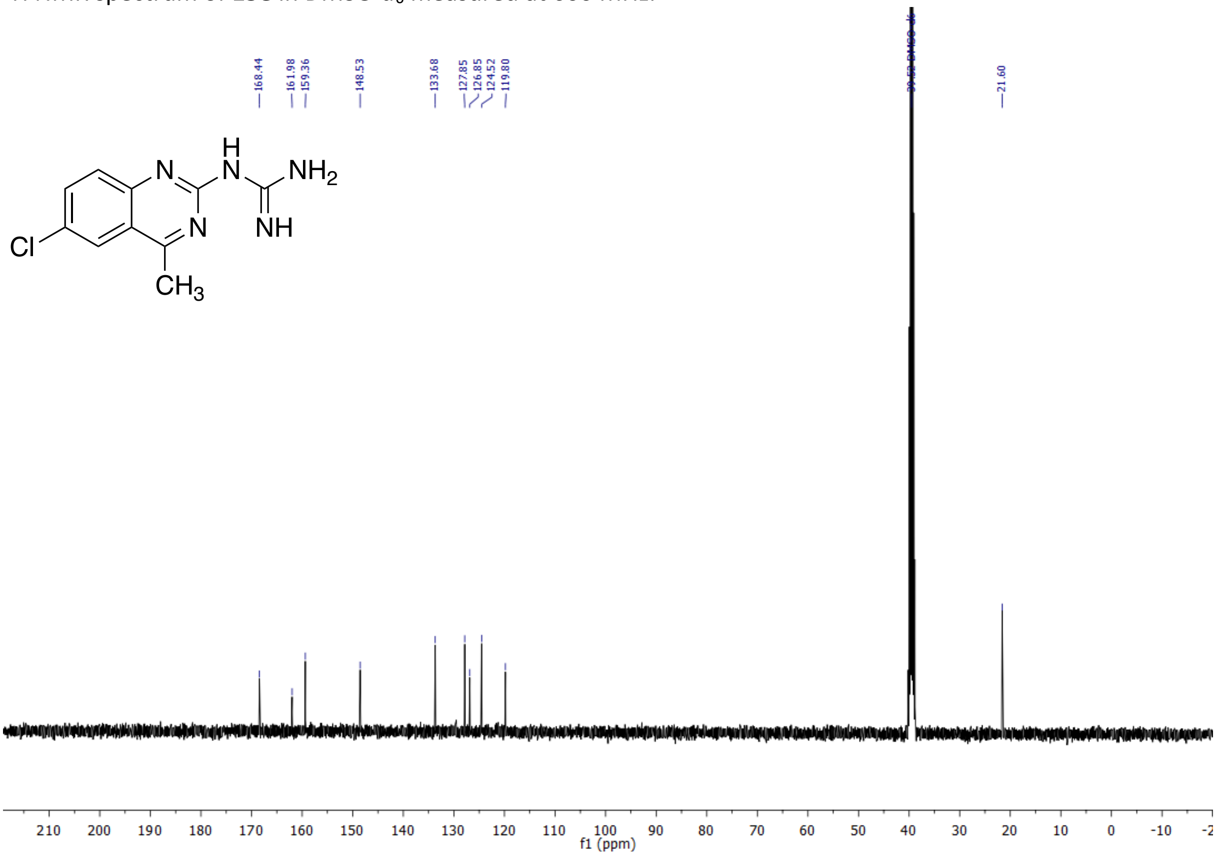

^{13}C NMR spectrum of **15e** in DMSO-d_6 measured at 100 MHz.

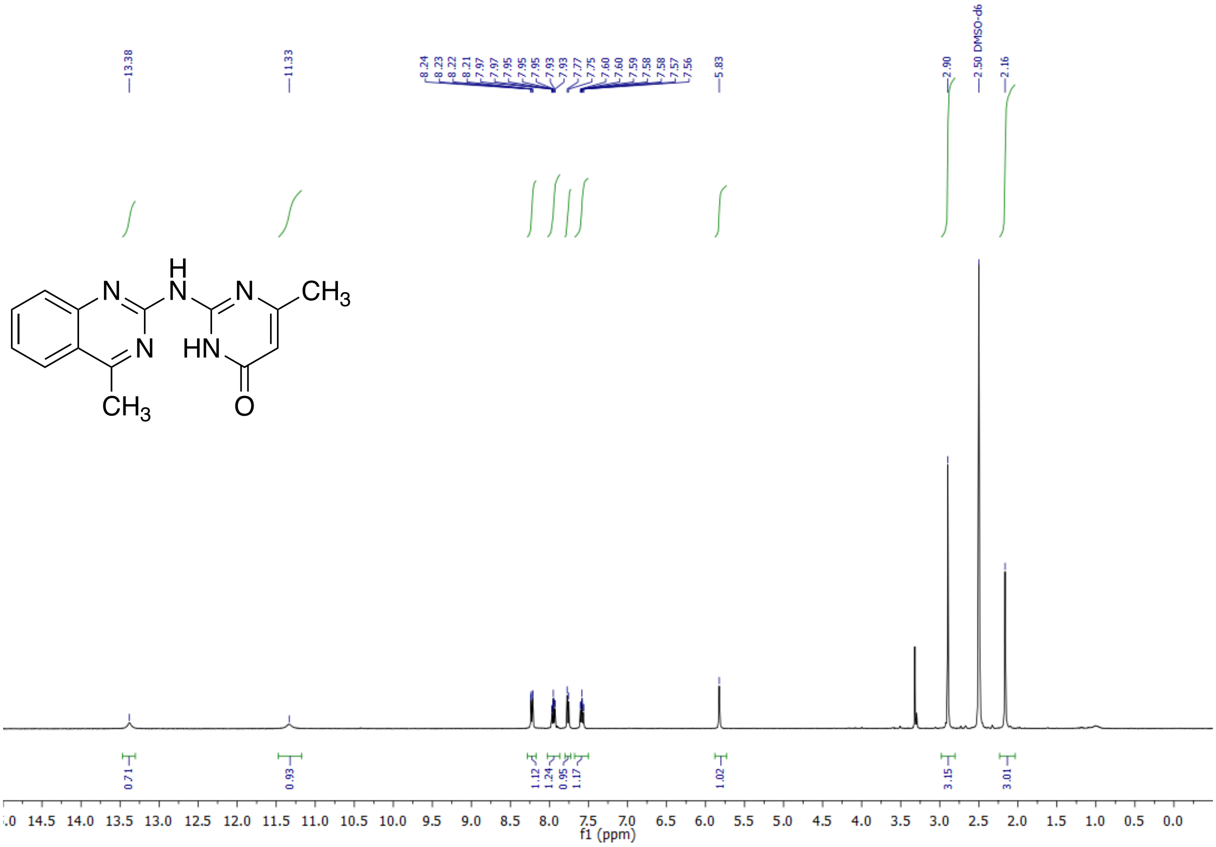

^{1}H NMR spectrum of **16a** in DMSO-d_6 measured at 400

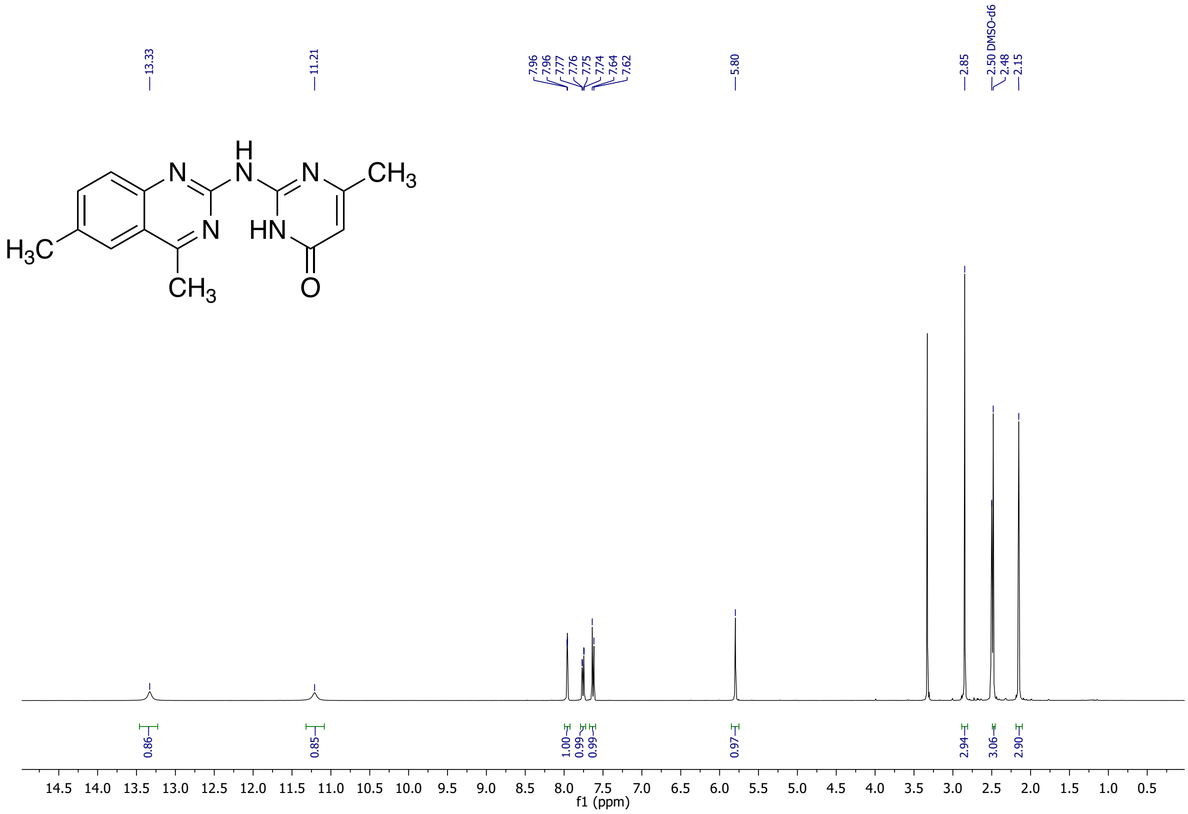

^{1}H NMR spectrum of **16b** in DMSO-d_6 measured at 400 MHz.

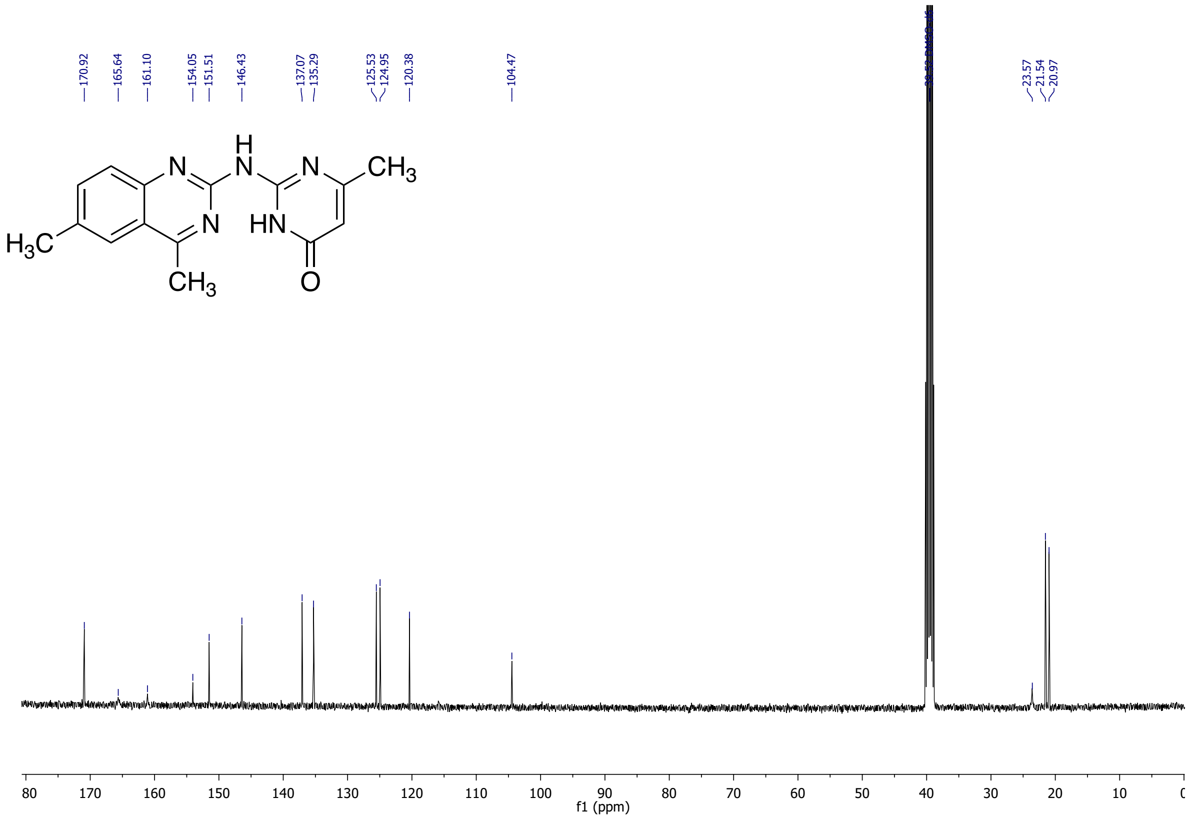

^{13}C NMR spectrum of **16b** in DMSO-d_6 measured at 100 MHz.

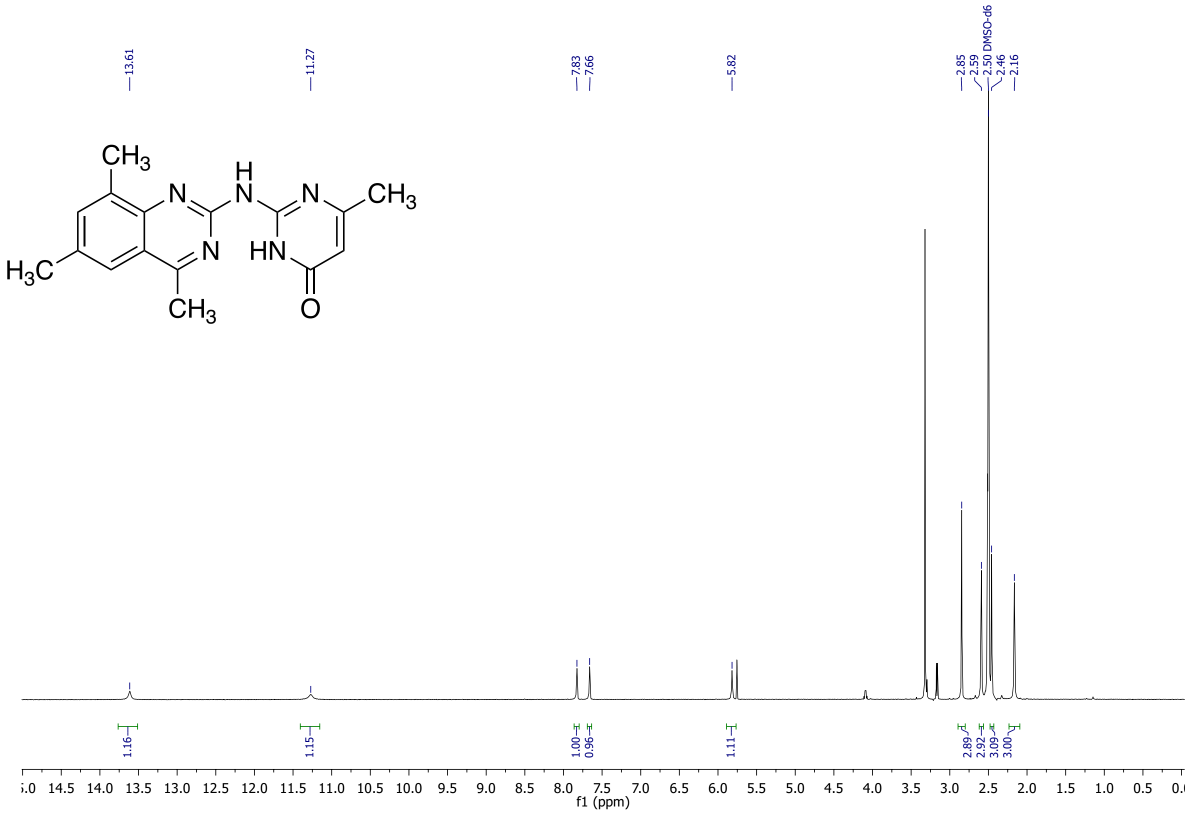

^{1}H NMR spectrum of **16c** in DMSO-d_6 measured at 400 MHz.

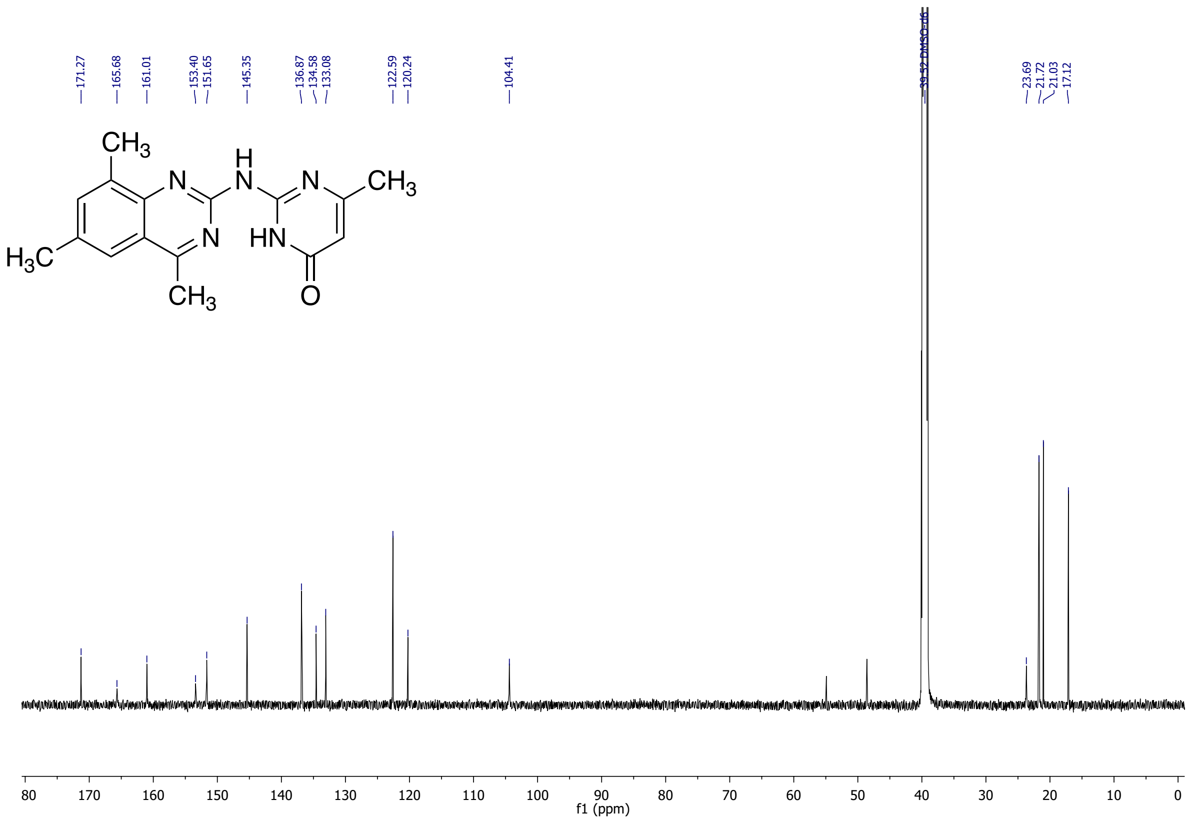

^{13}C NMR spectrum of **16c** in DMSO-d_6 measured at 151 MHz.

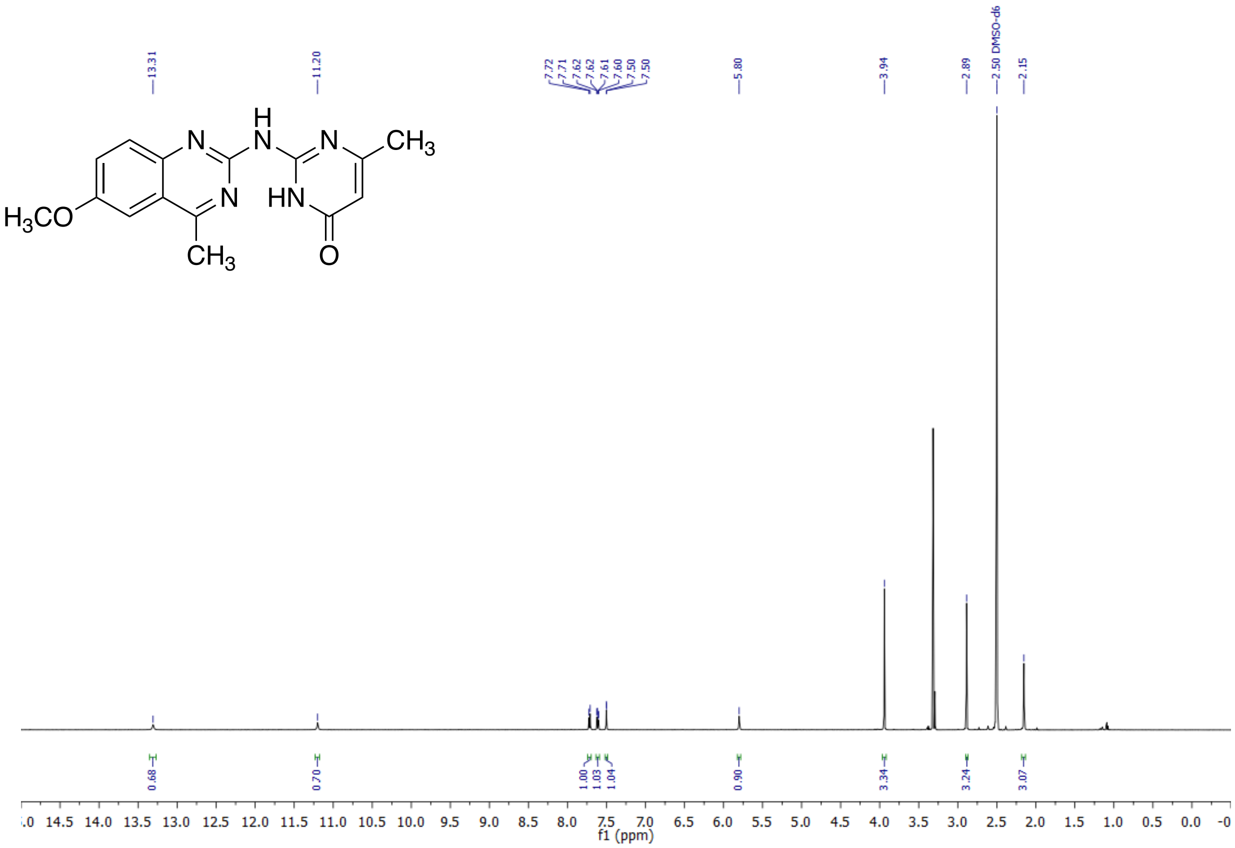

^{1}H NMR spectrum of **16d** in DMSO-d_6 measured at 600 MHz.

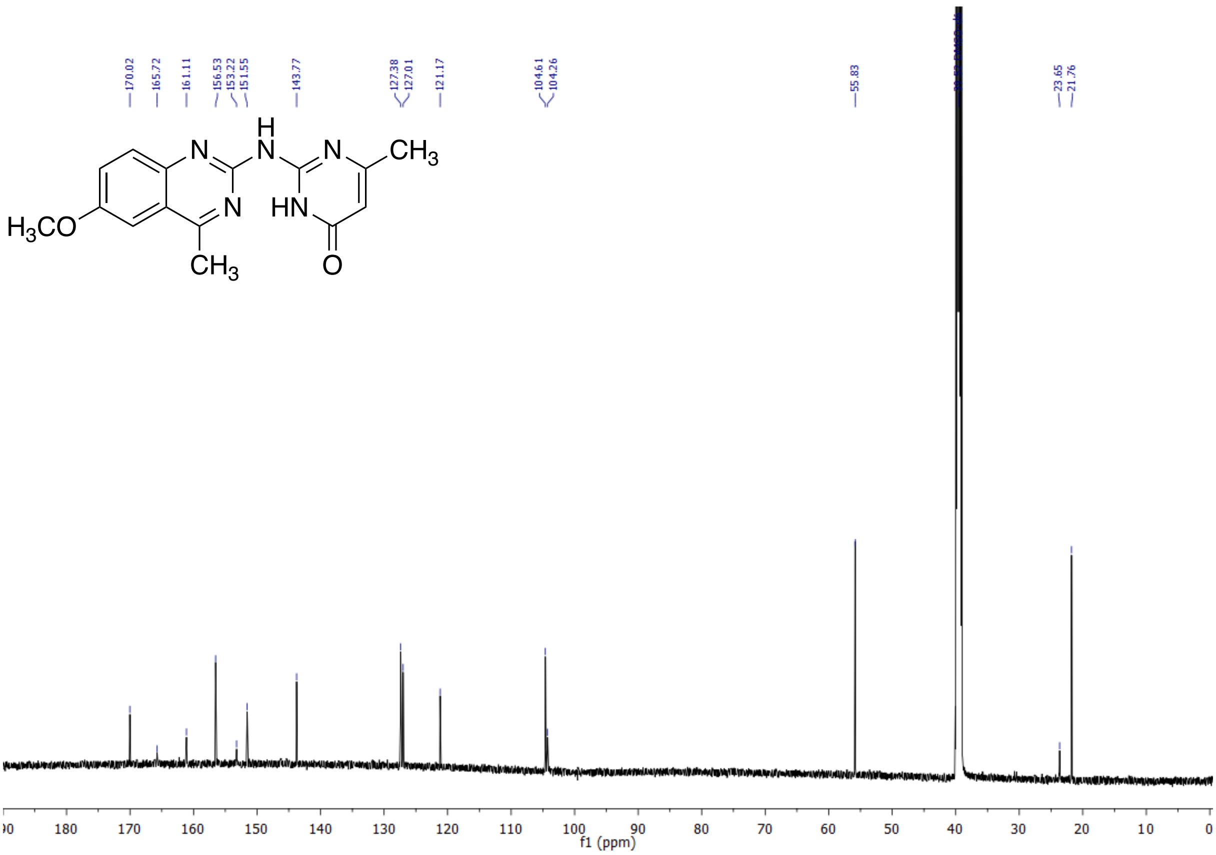

^{13}C NMR spectrum of **16d** in DMSO-d_6 measured at 151 MHz.

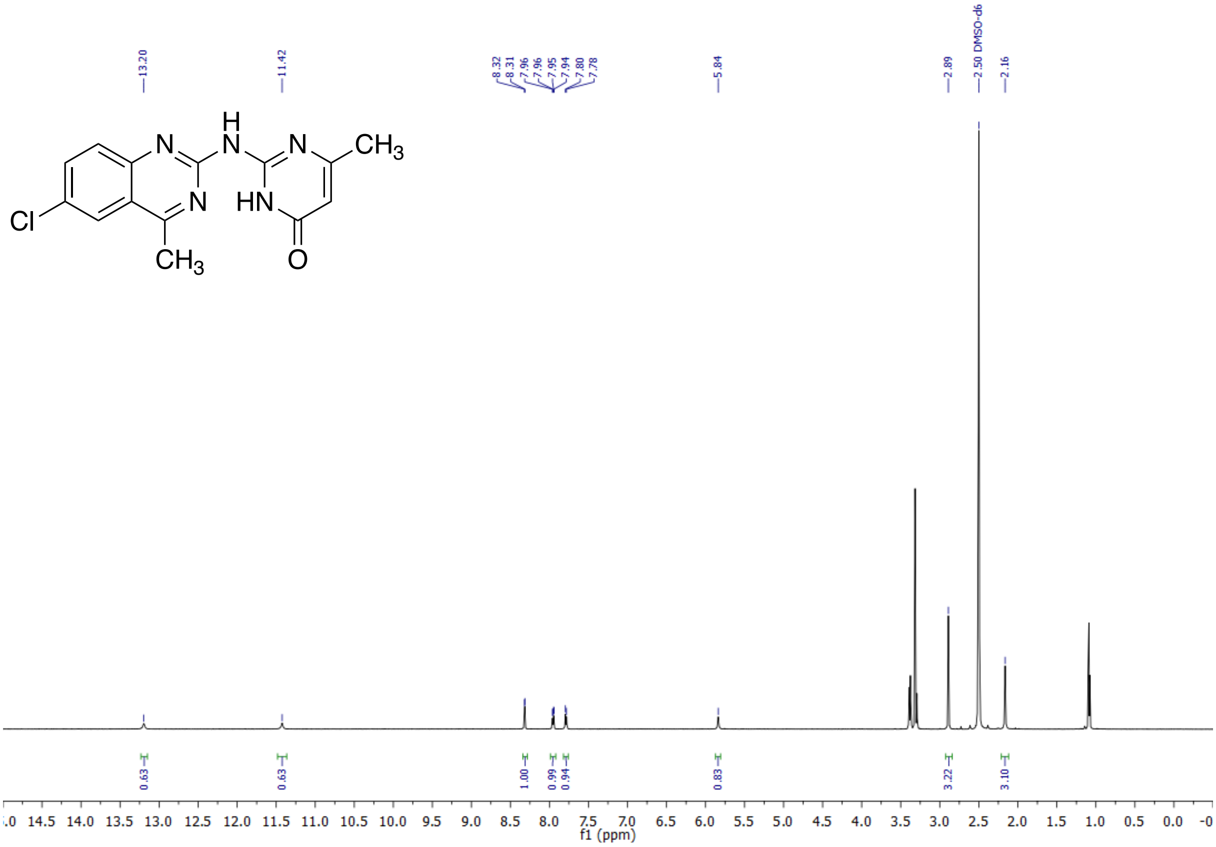

^{1}H NMR spectrum of **16e** in DMSO-d_6 measured at 600 MHz.

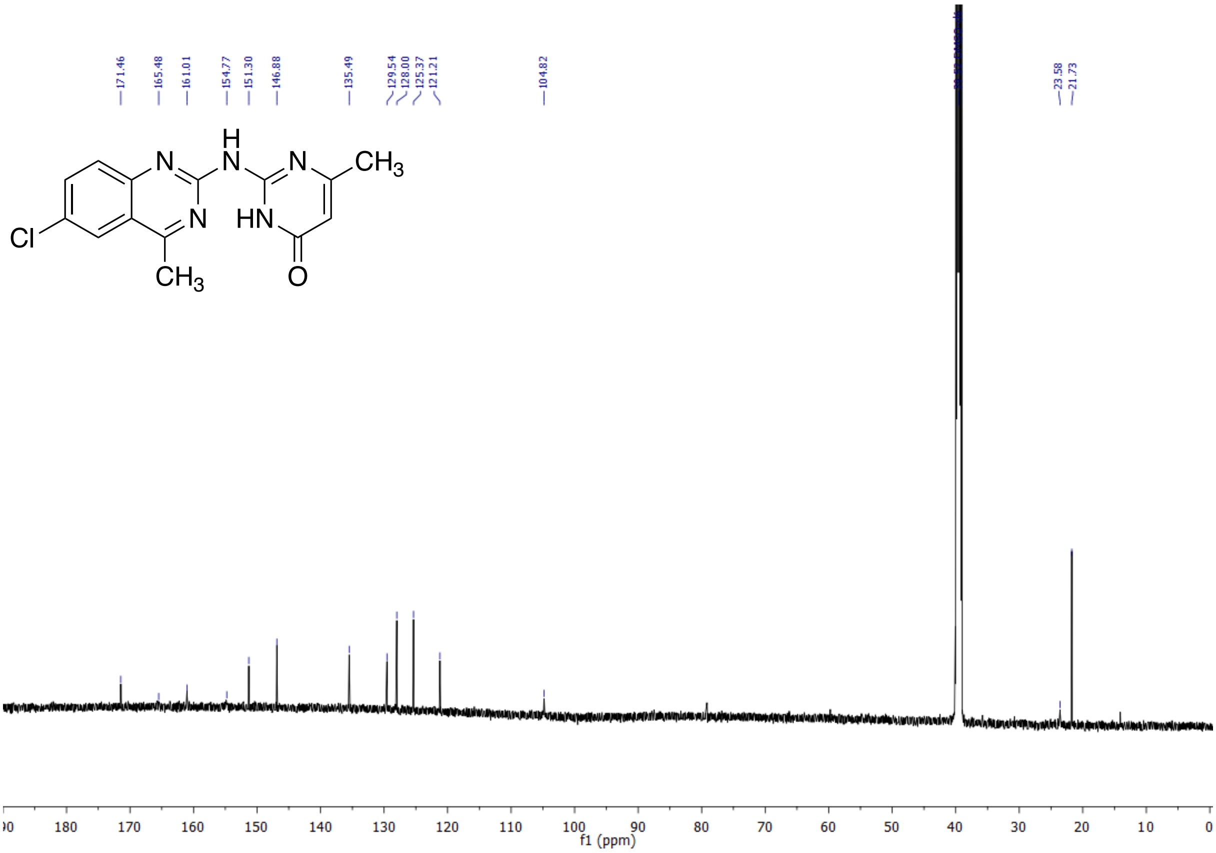

^{13}C NMR spectrum of **16e** in DMSO-d_6 measured at 151 MHz.

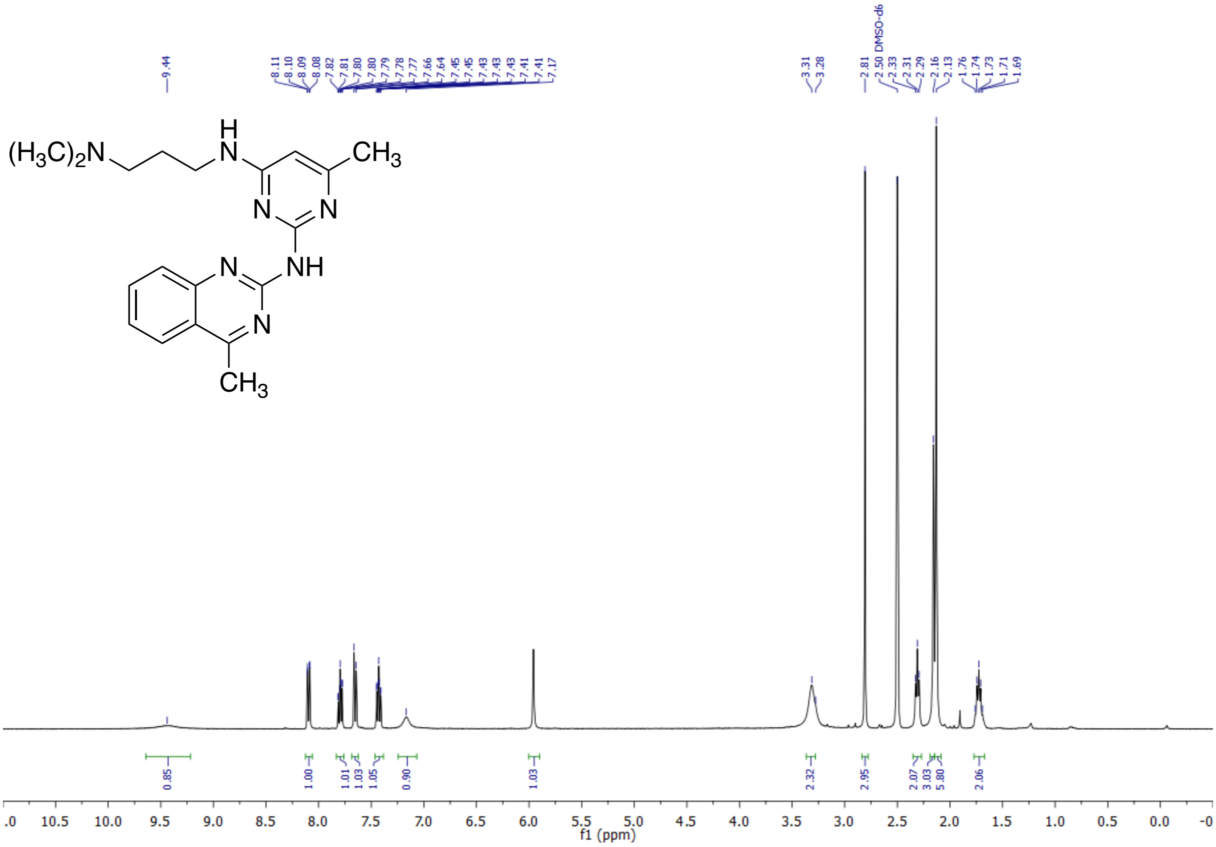

^{1}H NMR spectrum of **3** in DMSO-d_6 measured at 400 MHz.

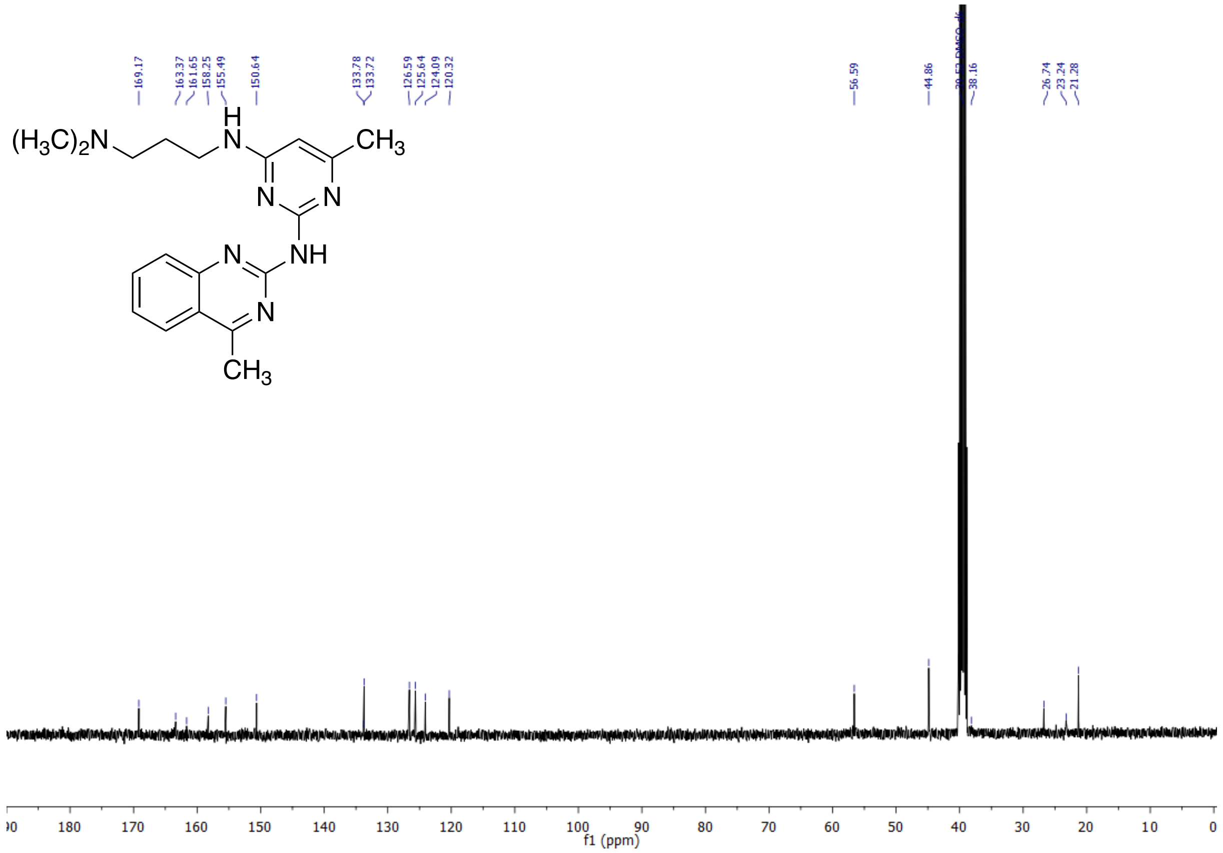

^{13}C NMR spectrum of **3** in DMSO-d_6 measured at 100 MHz.

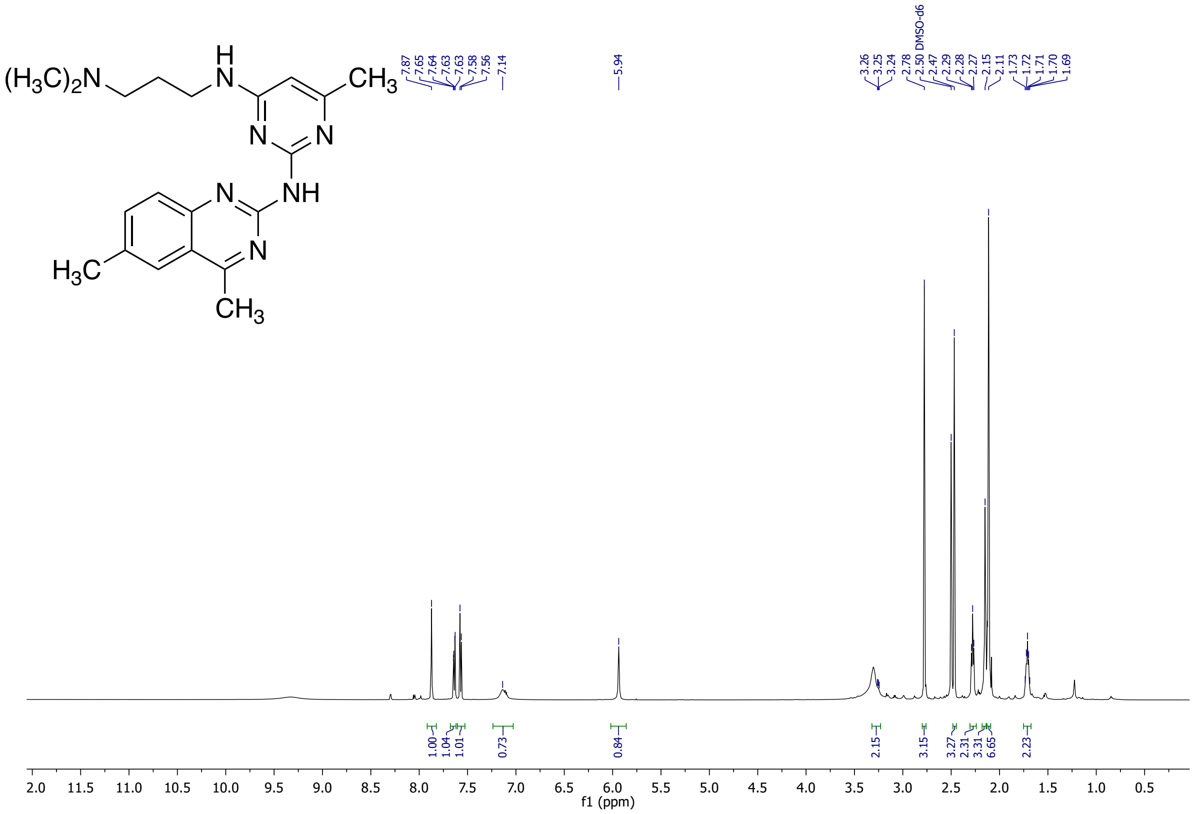

^{1}H NMR spectrum of **4** in DMSO-d_6 measured at 600 MHz.

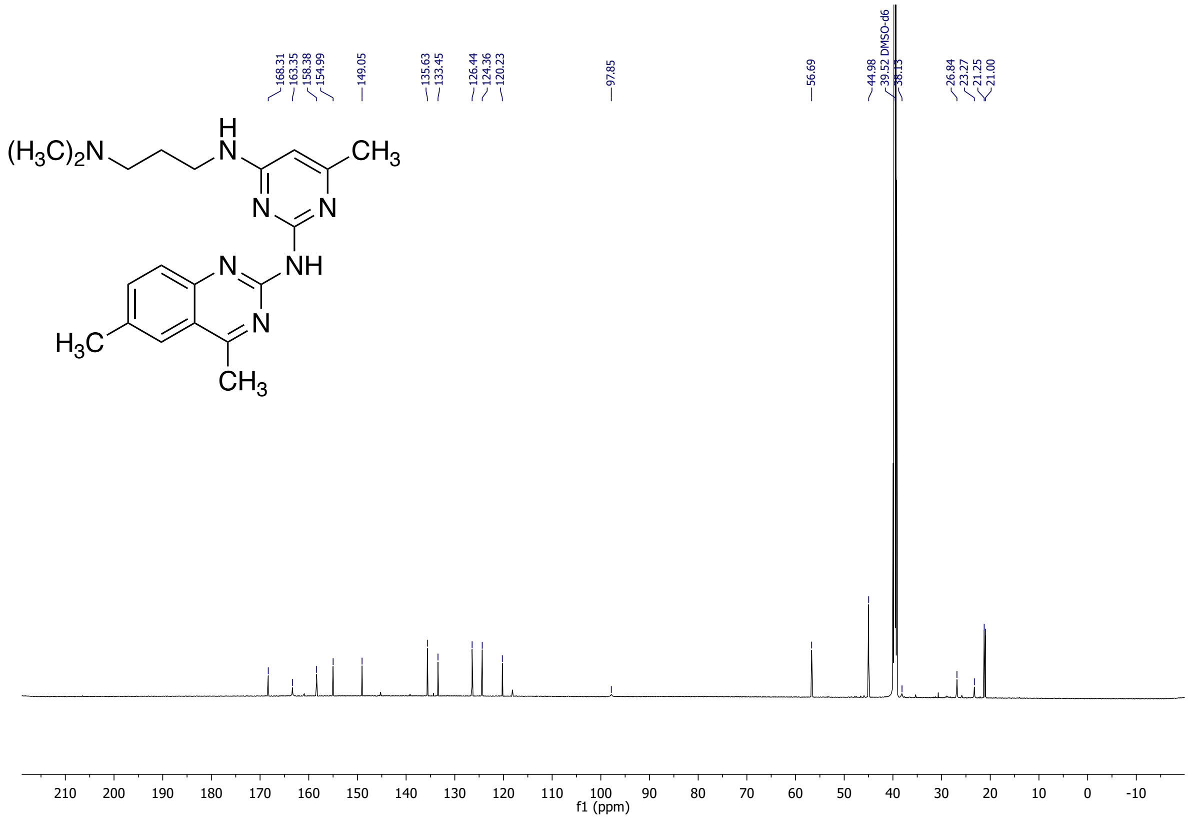

^{13}C NMR spectrum of **4** in DMSO-d_6 measured at 151 MHz.

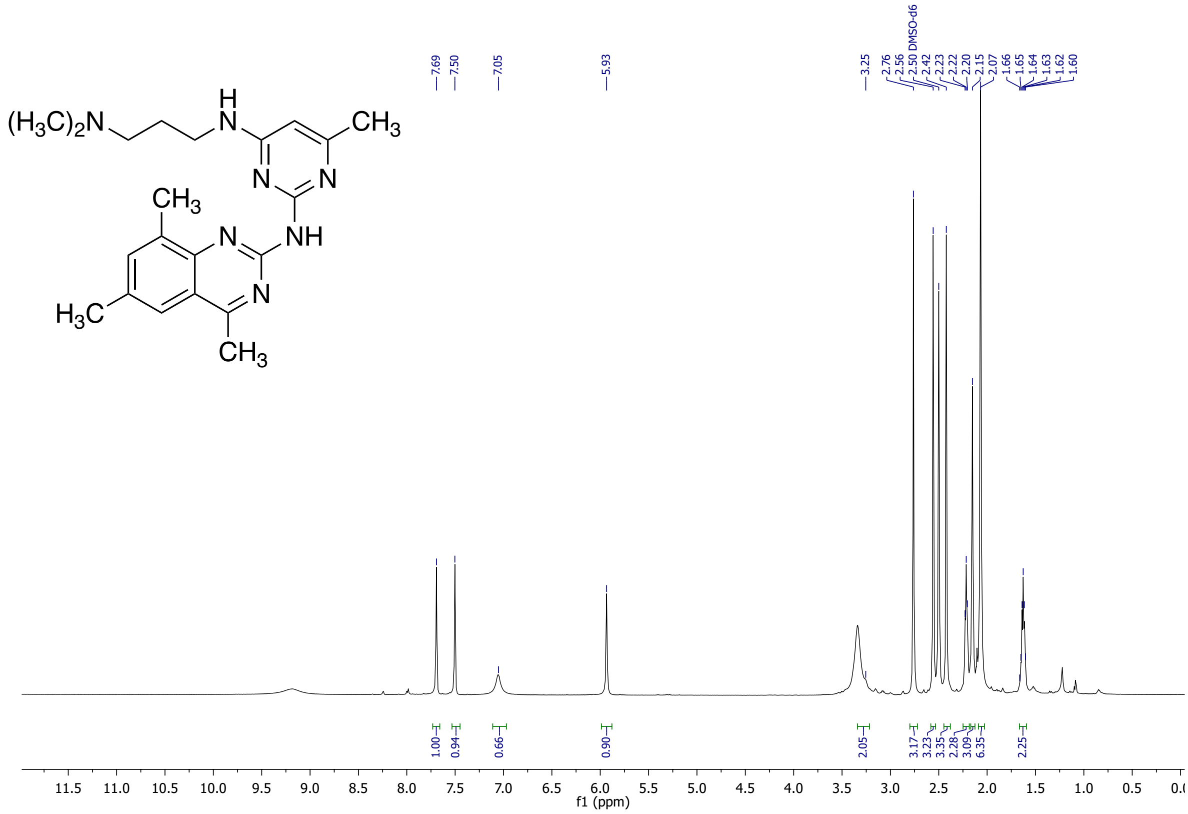

^{1}H NMR spectrum of **5** in DMSO-d_6 measured at 600 MHz.

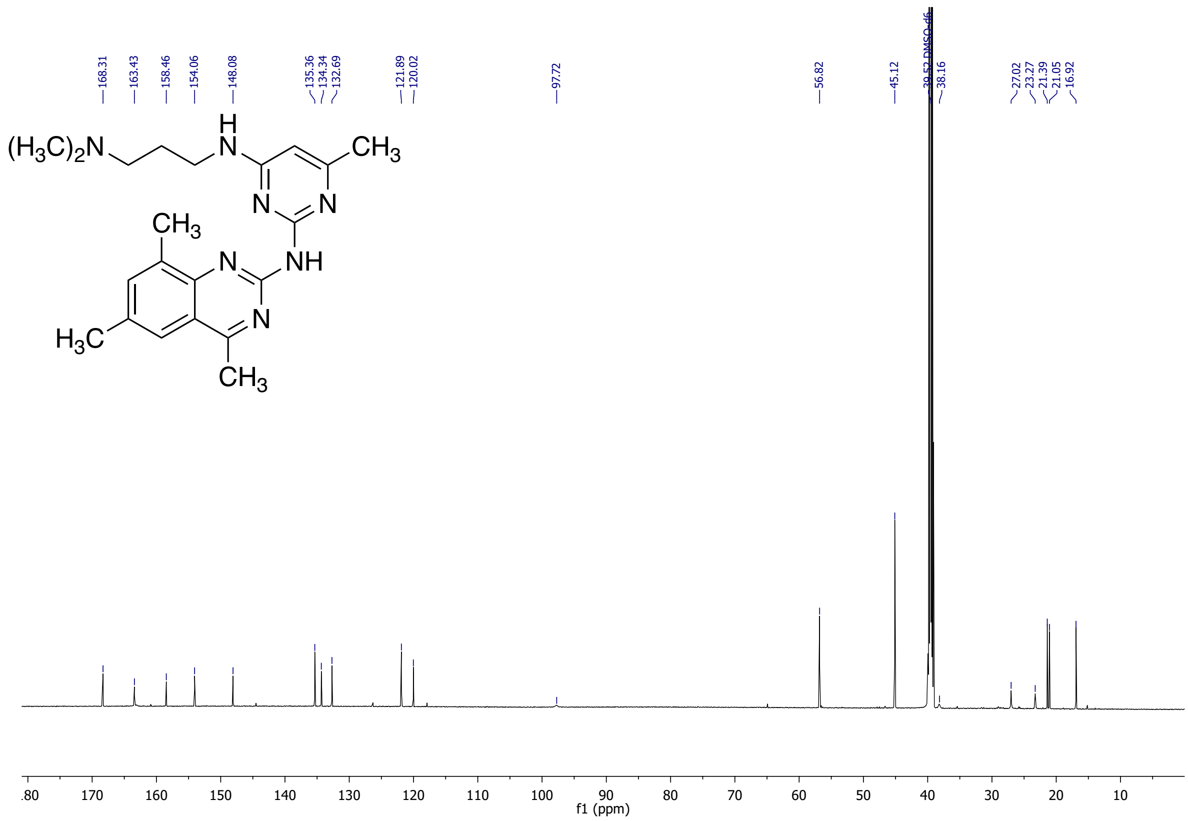

^{13}C NMR spectrum of **5** in DMSO-d_6 measured at 151 MHz.

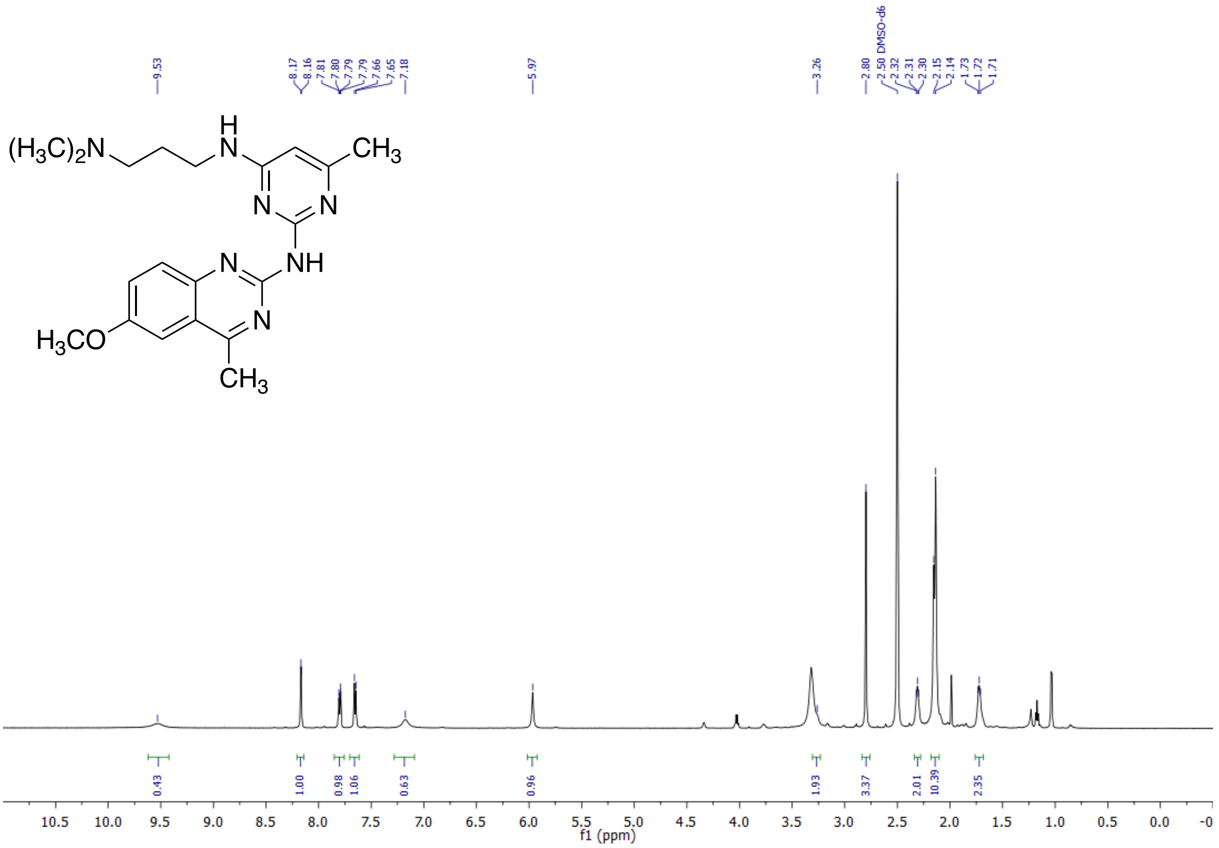

^{1}H NMR spectrum of **6** in DMSO-d_6 measured at 600 MHz.

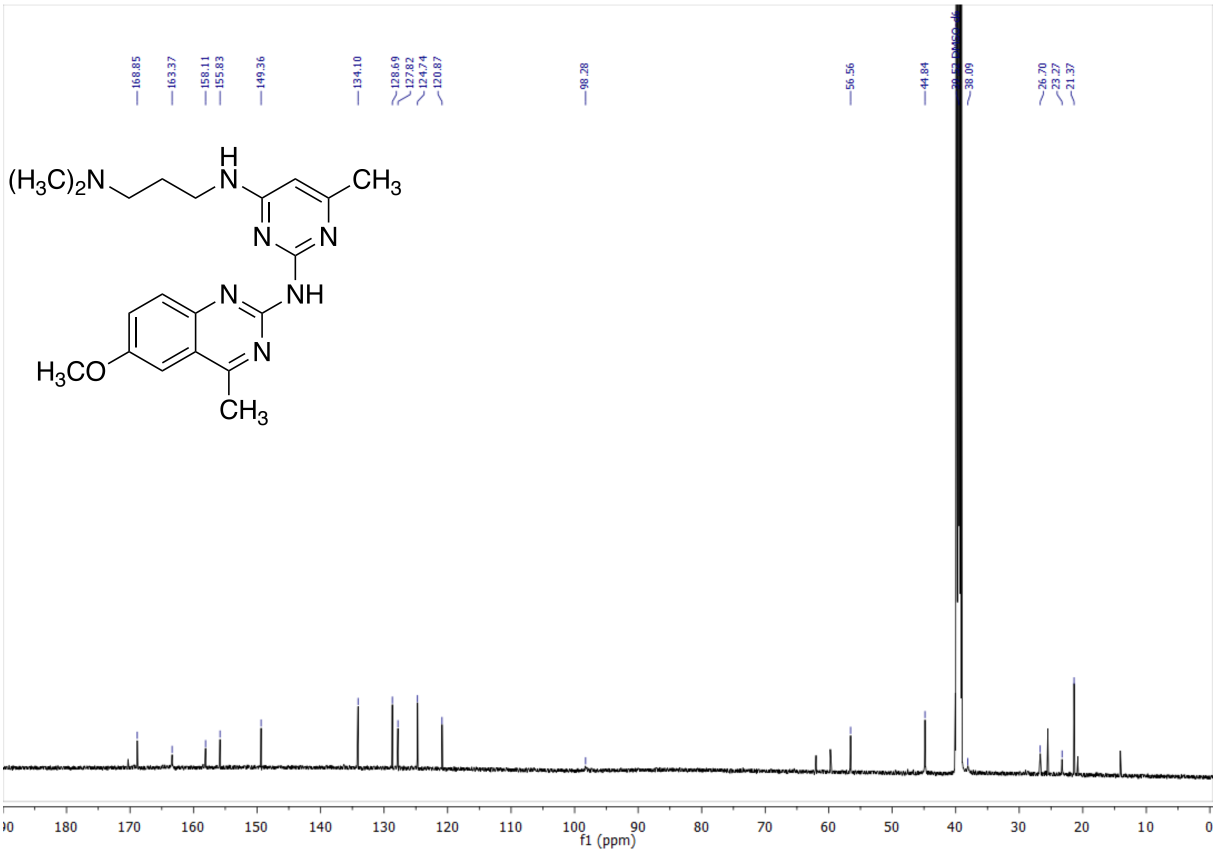

^{13}C NMR spectrum of **6** in DMSO-d_6 measured at 151 MHz.

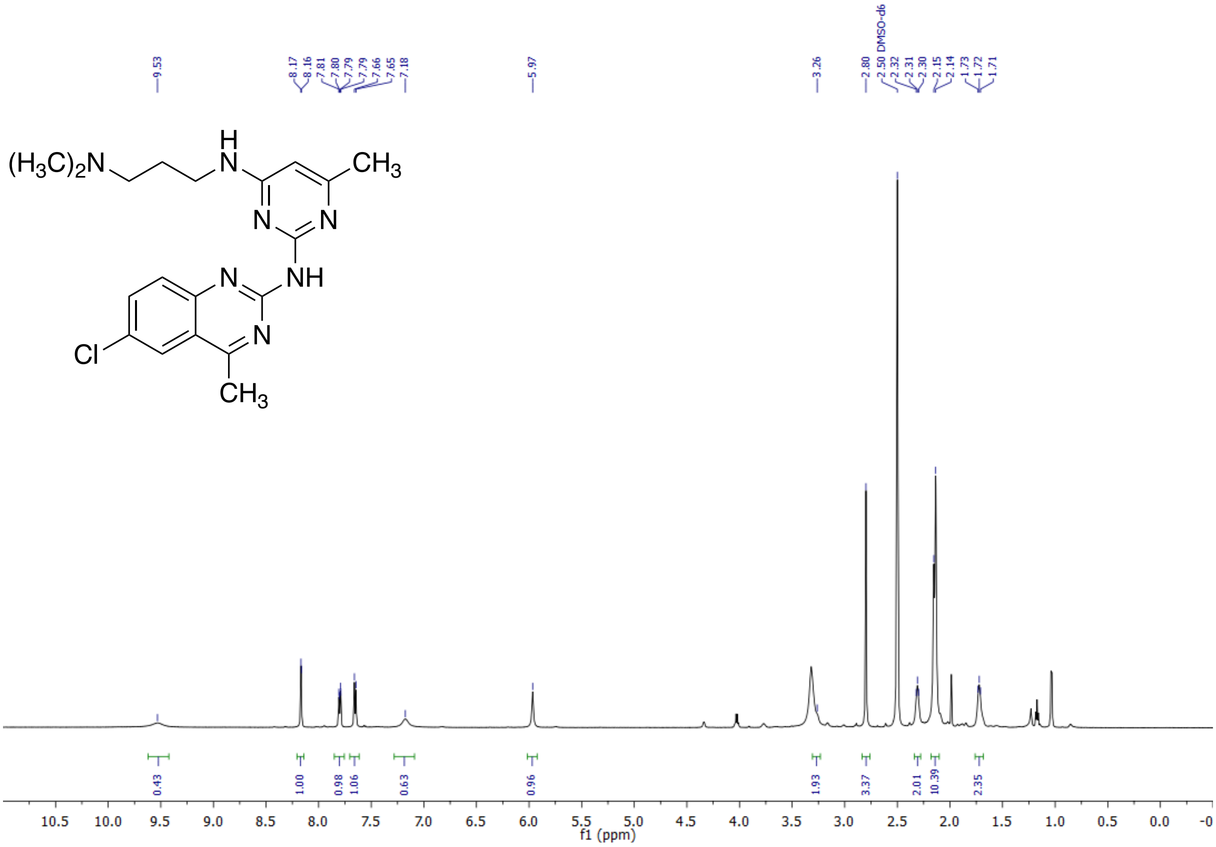

^{1}H NMR spectrum of **7** in DMSO-d_6 measured at 600 MHz.

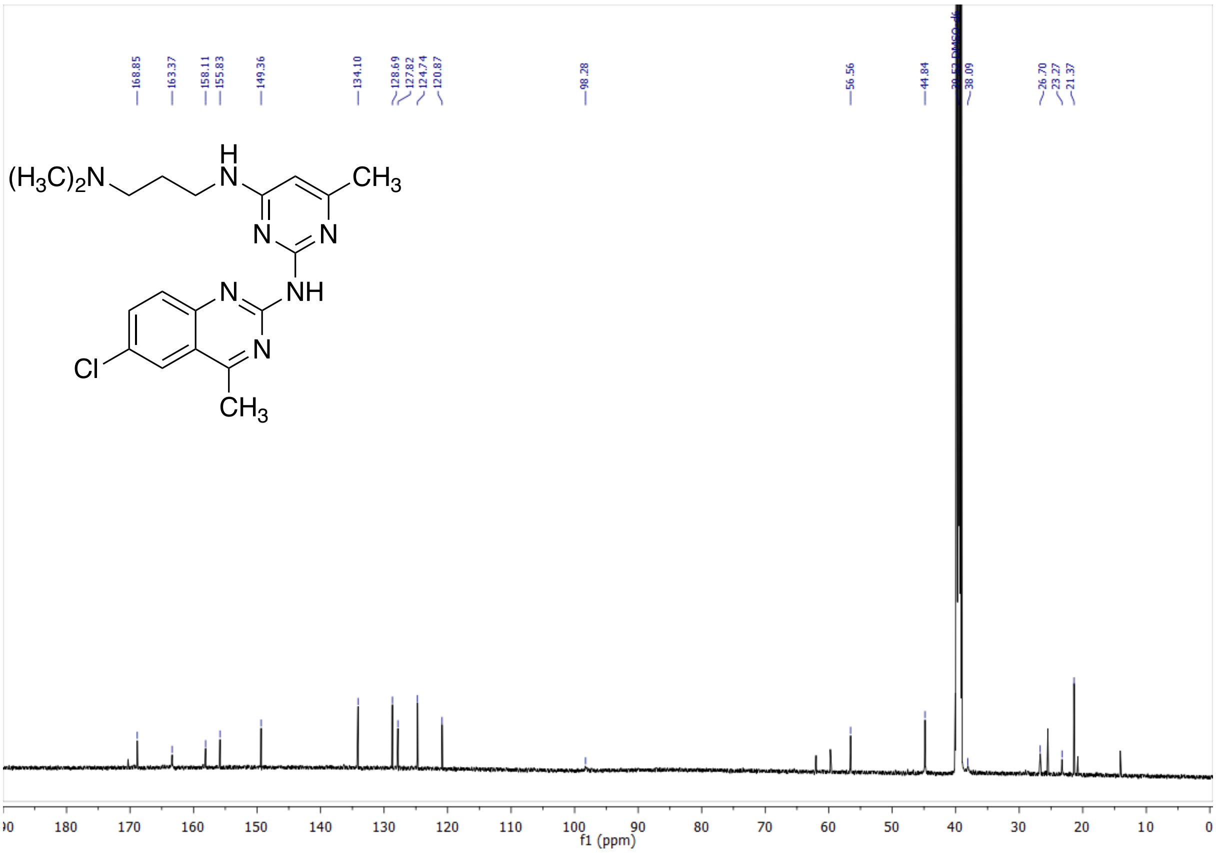

^{13}C NMR spectrum of **X** in DMSO-d_6 measured at 151 MHz.

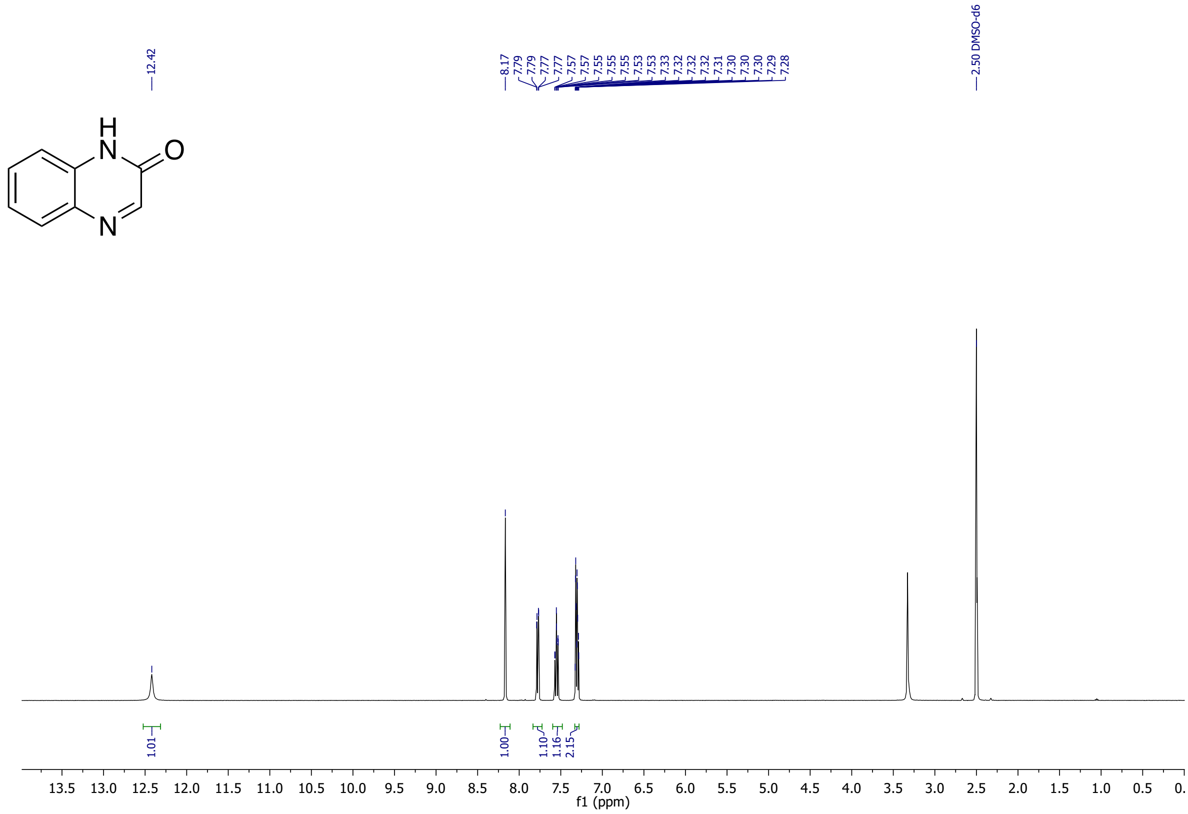

^{1}H NMR spectrum of **17** in DMSO-d_6 measured at 400 MHz.

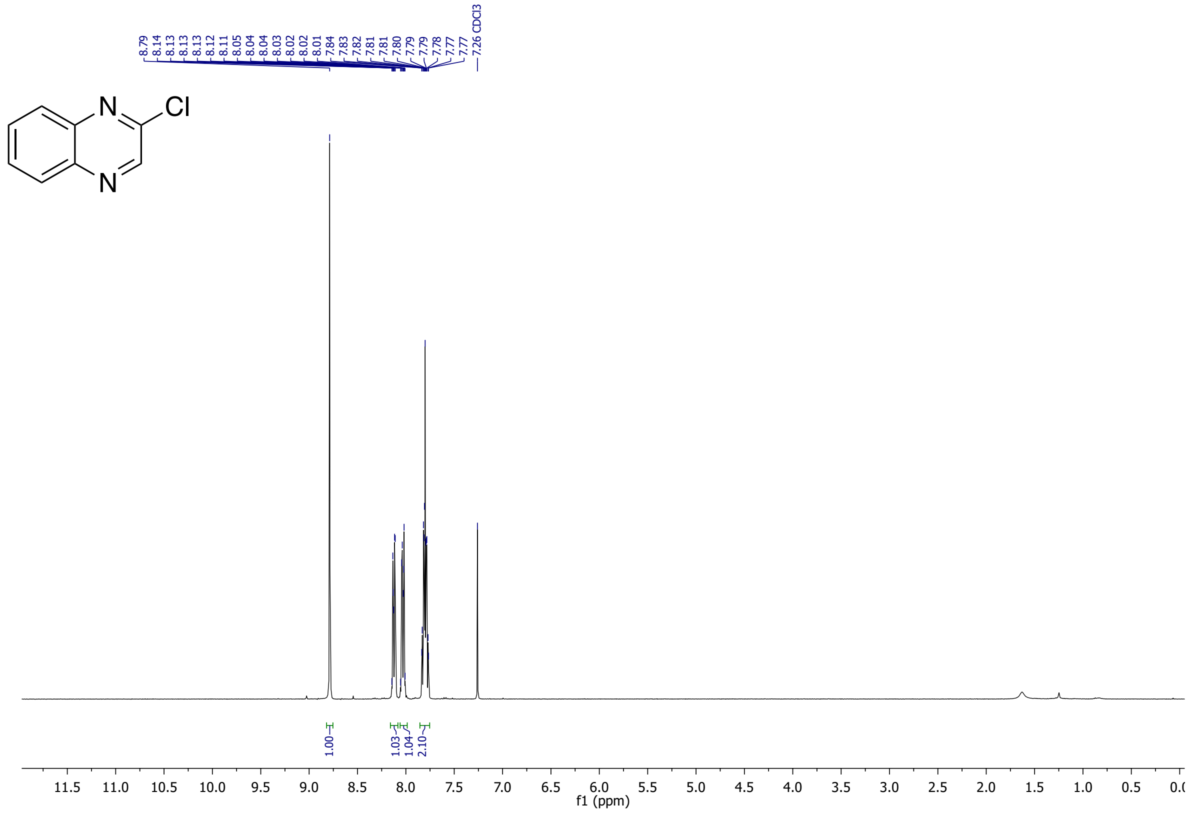

^1H NMR spectrum of **18** in $CDCl_3$ measured at 400 MHz.

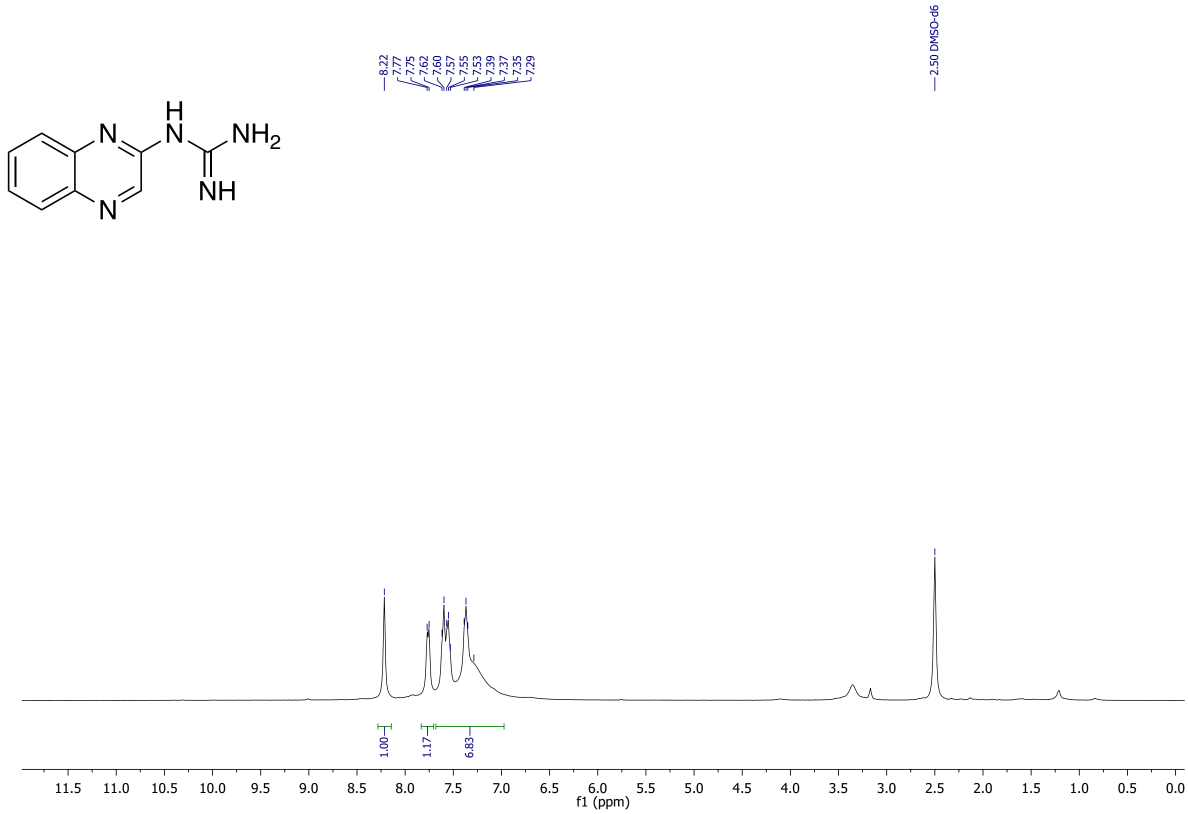

^1H NMR spectrum of **19** in DMSO-d_6 measured at 400 MHz.

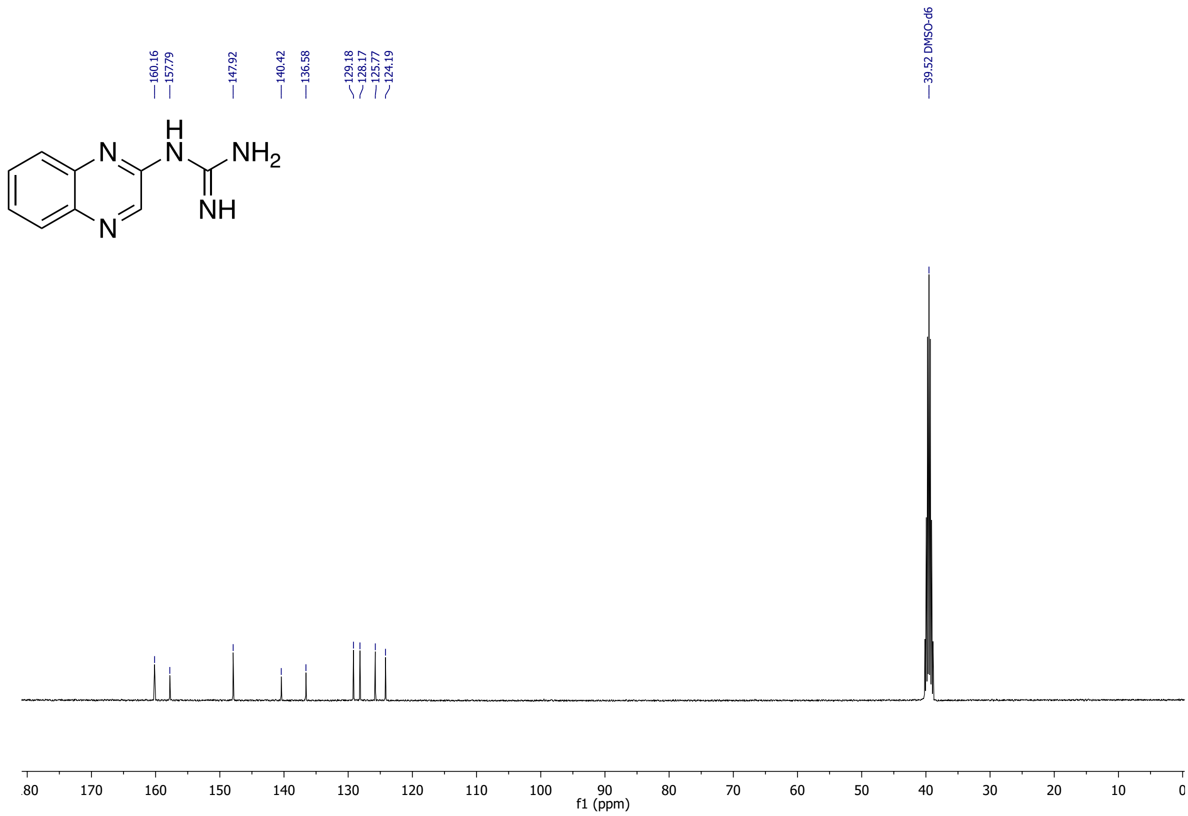

^{13}C NMR spectrum of **19** in DMSO-d_6 measured at 100 MHz.

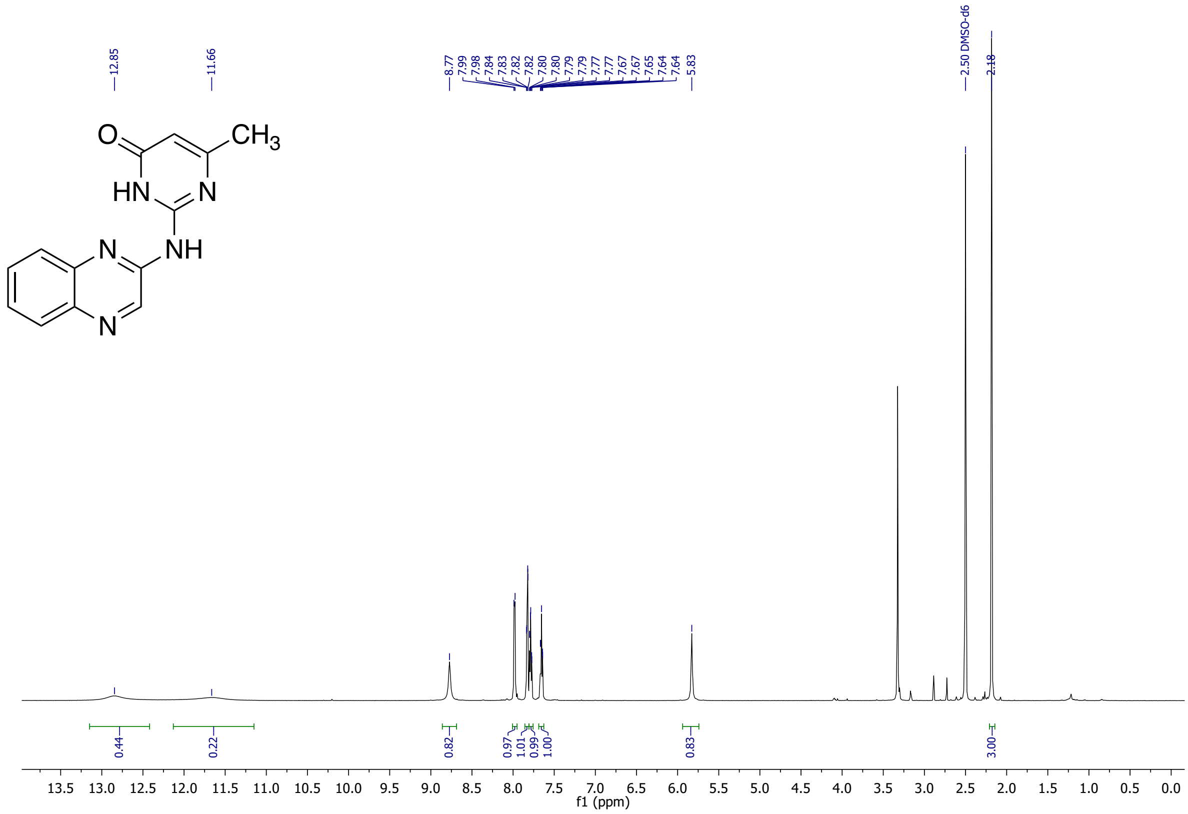

^{1}H NMR spectrum of **20** in DMSO-d_6 measured at 400 MHz.

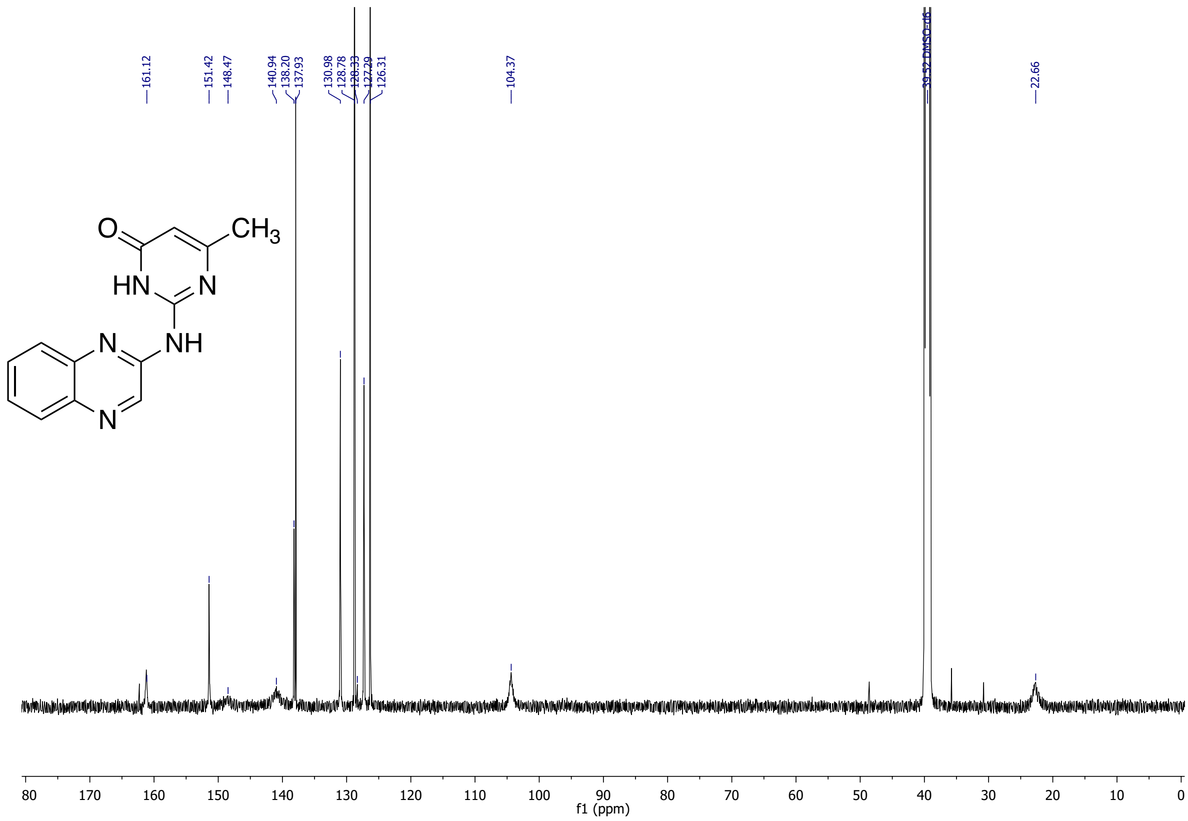

^{13}C NMR spectrum of **20** in DMSO-d_6 measured at 600 MHz.

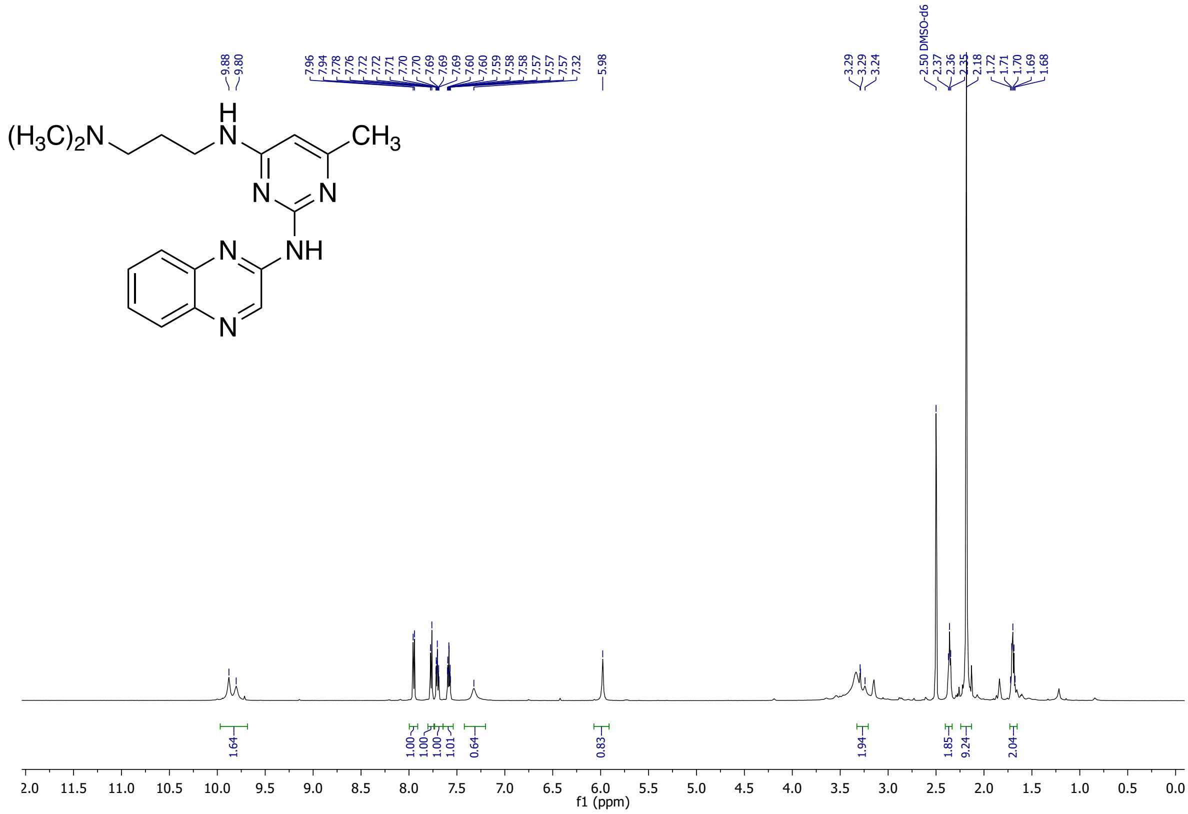

^{1}H NMR spectrum of **8** in DMSO-d_6 measured at 600 MHz.

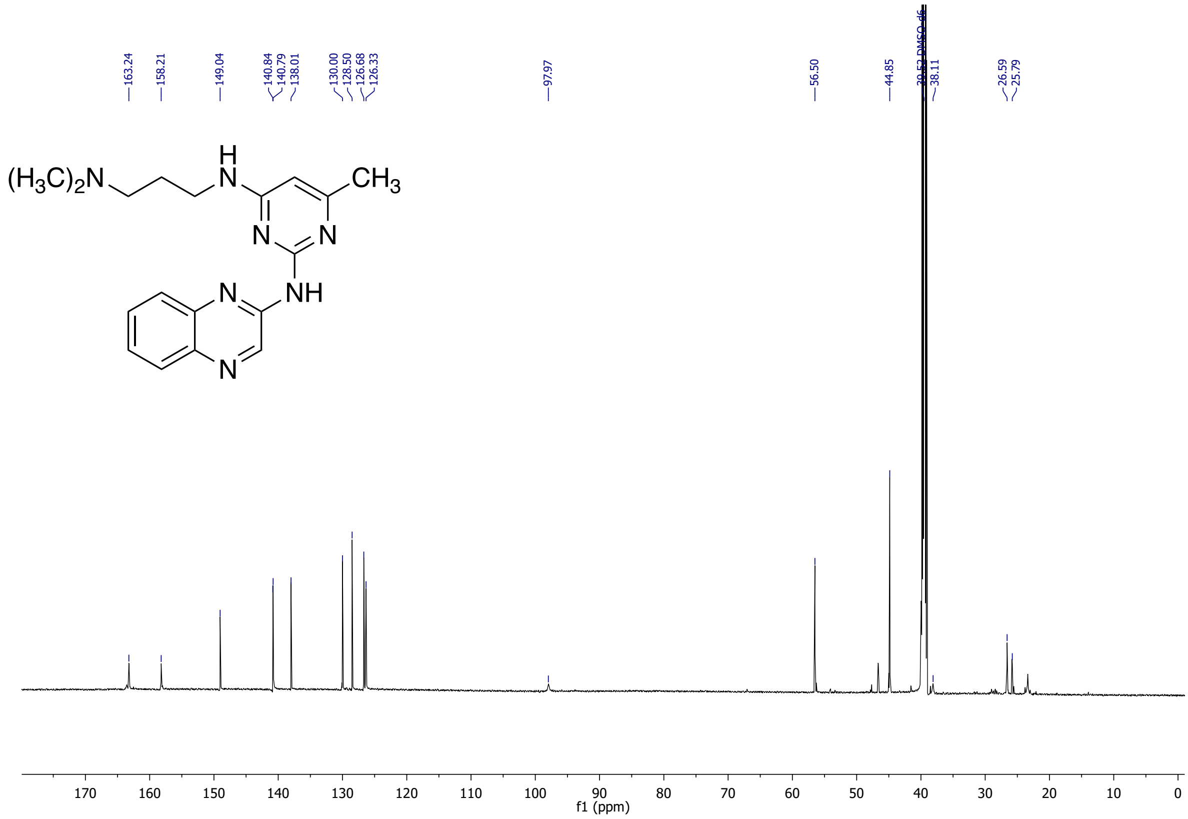

^{13}C NMR spectrum of **8** in DMSO-d_6 measured at 150 MHz.

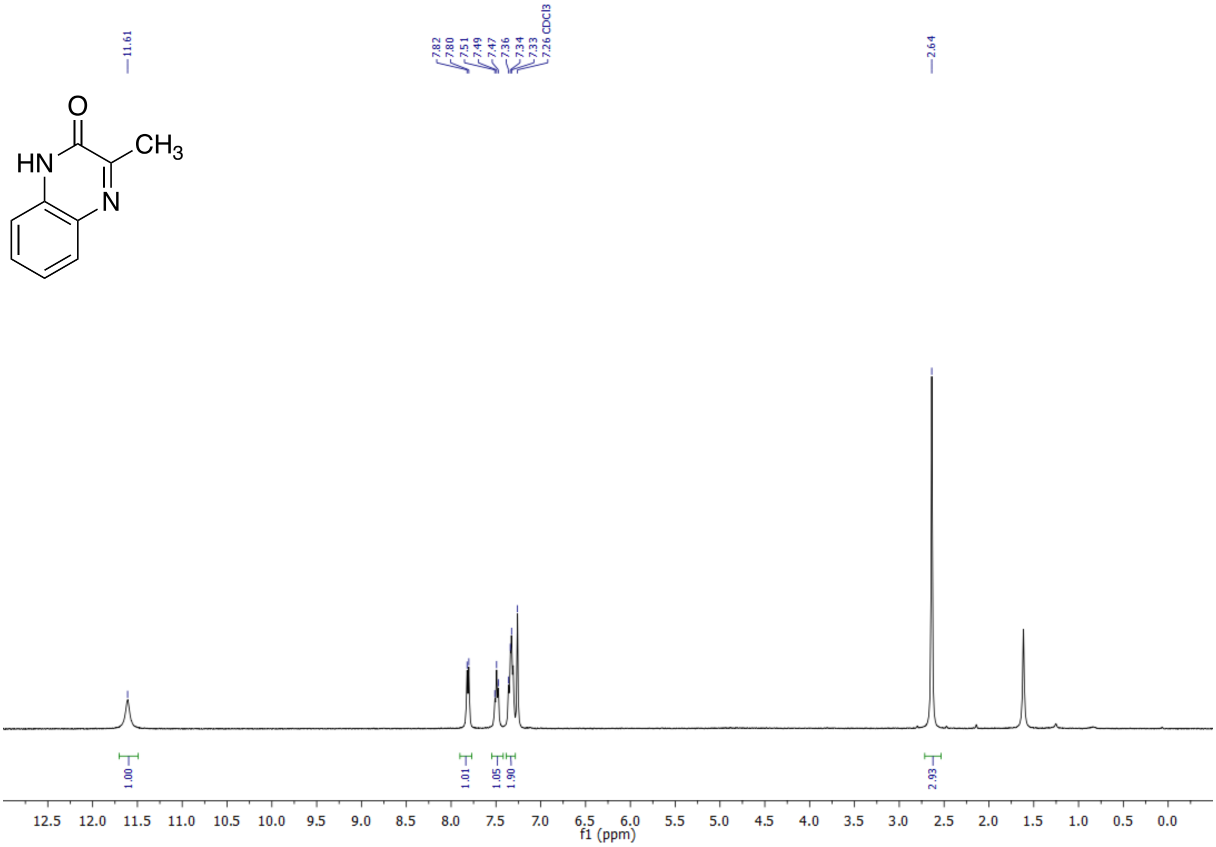

^{1}H NMR spectrum of **21a** in $CDCl_3$ measured at 400 MHz.

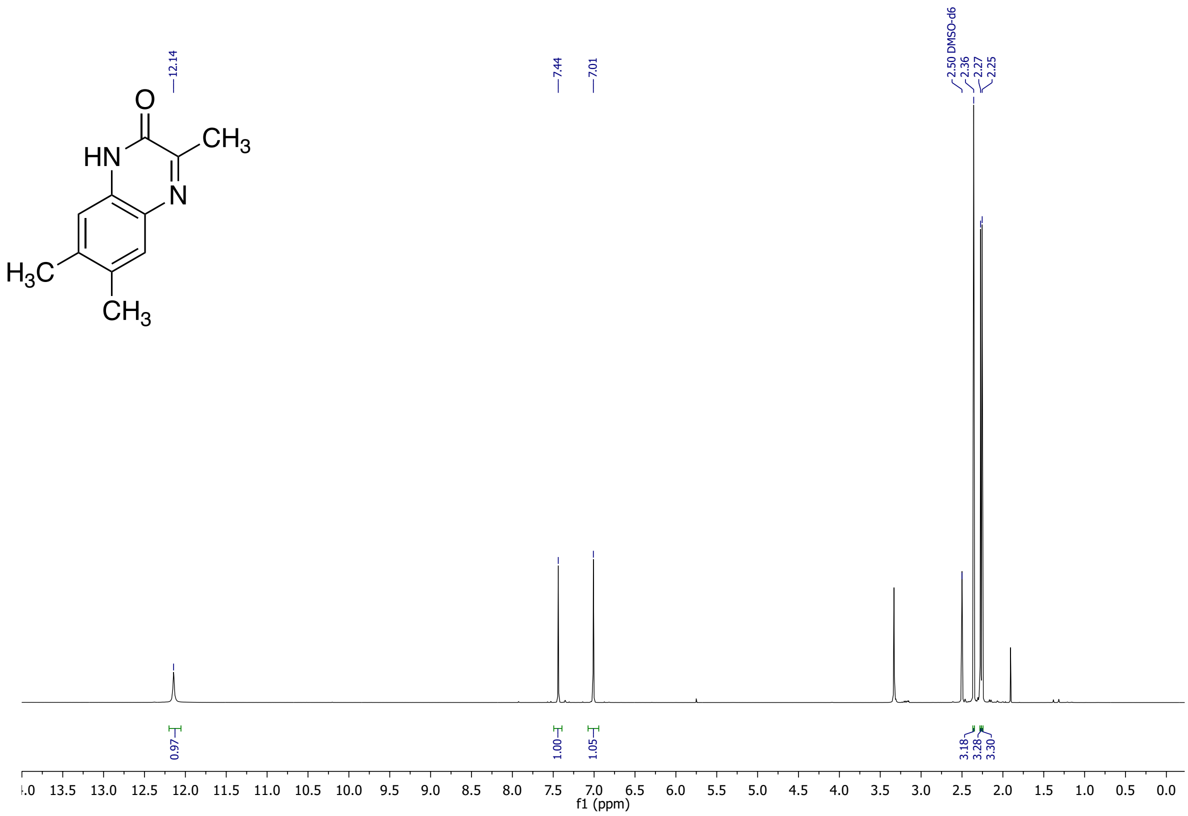

^{1}H NMR spectrum of **21b** in DMSO-d_6 measured at 600 MHz.

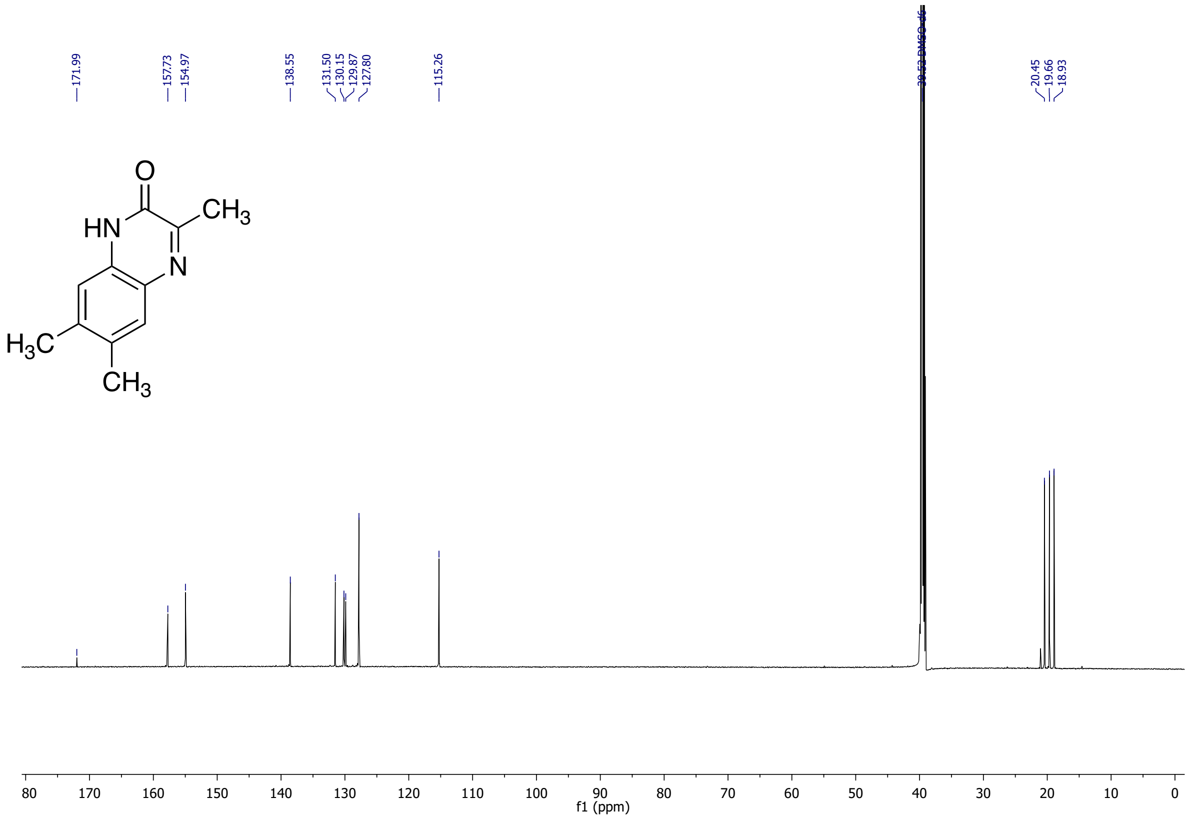

^{13}C NMR spectrum of **21b** in DMSO-d_6 measured at 151 MHz.

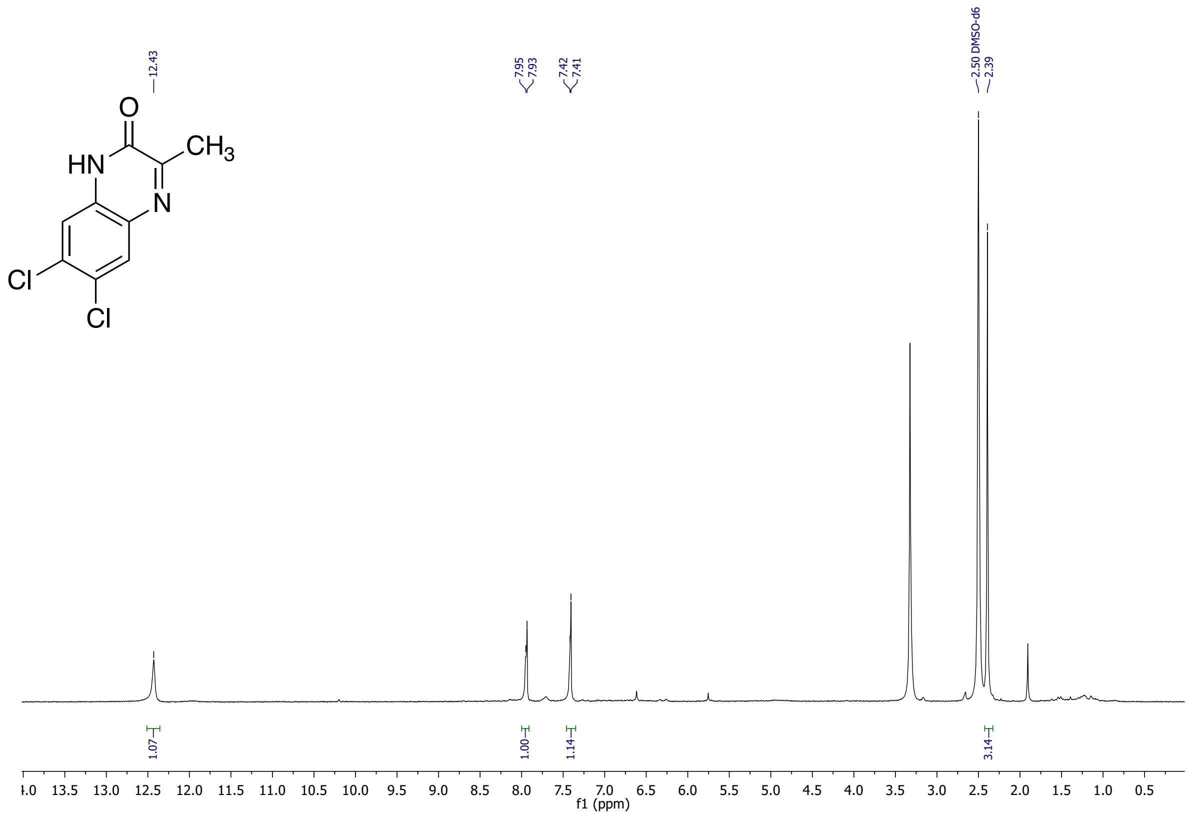

^{1}H NMR spectrum of **21c** in DMSO-d_6 measured at 400 MHz.

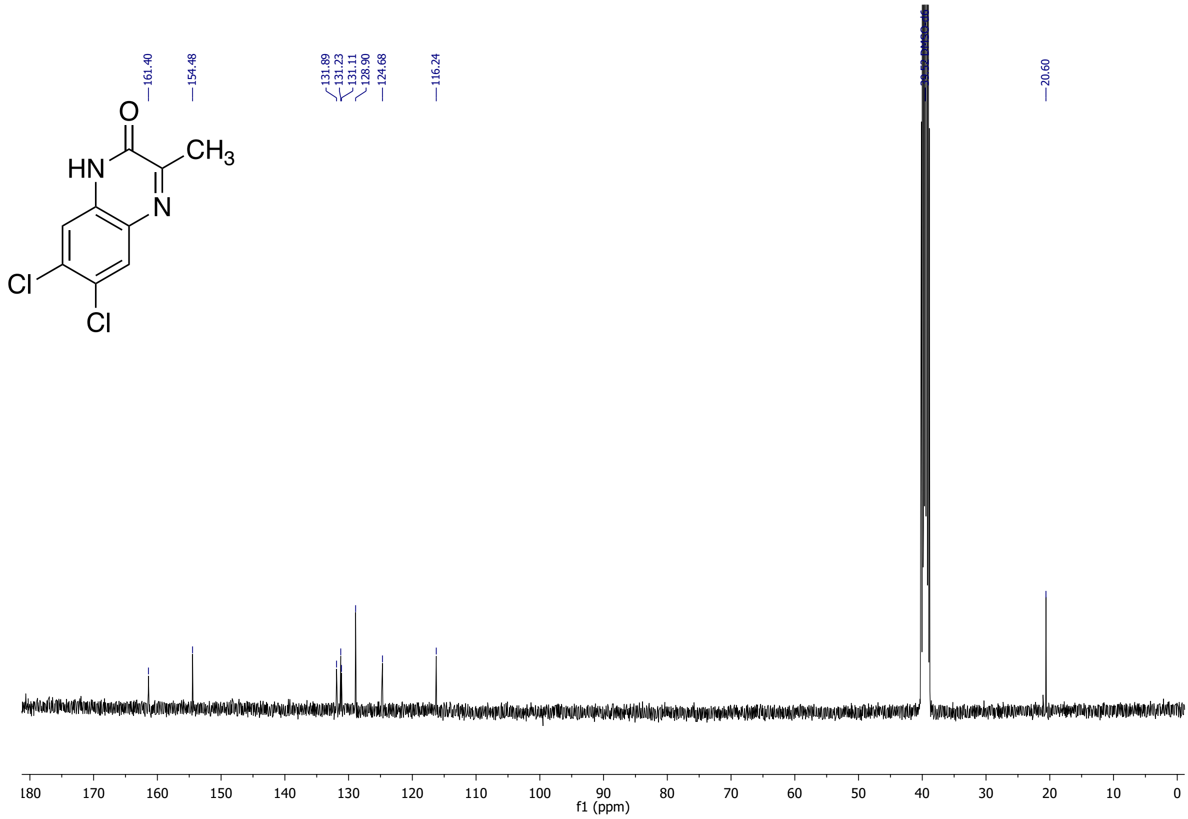

^{13}C NMR spectrum of **21c** in DMSO-d_6 measured at 100 MHz.

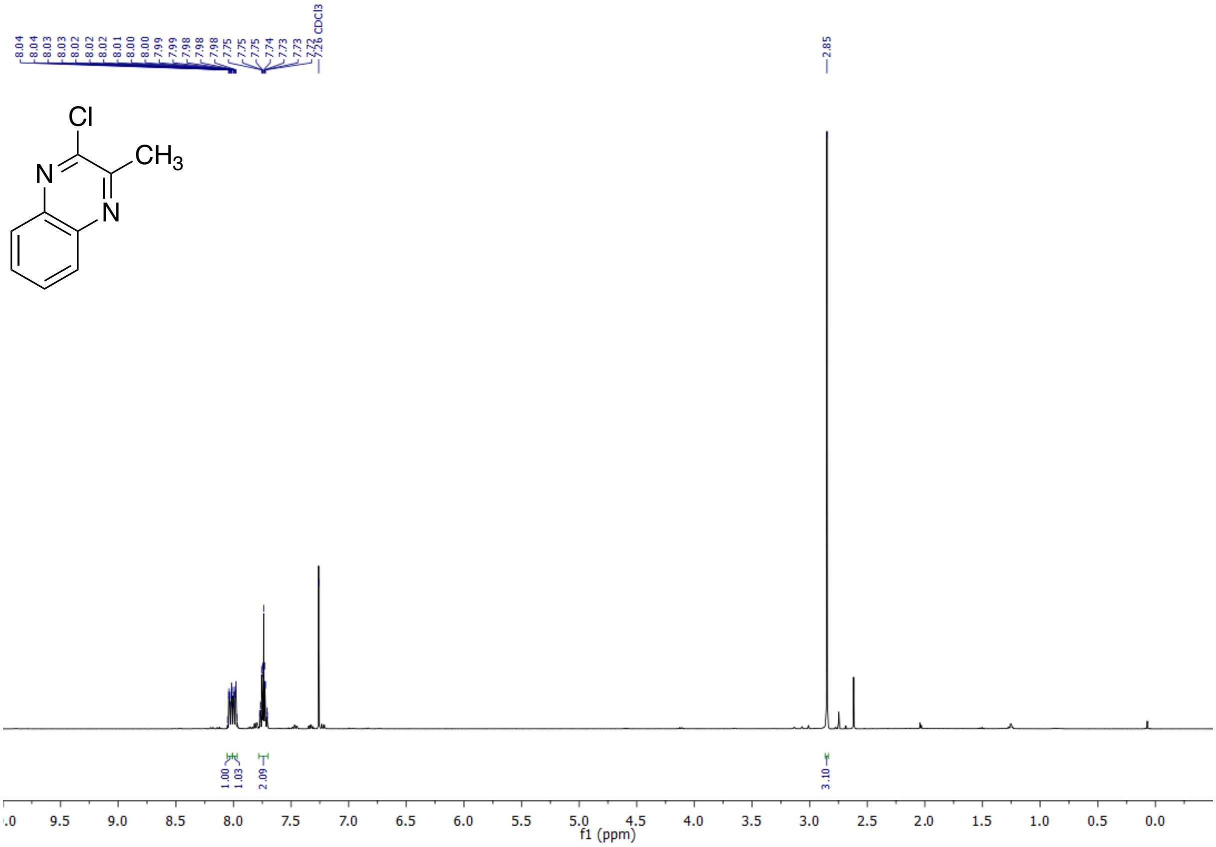

^{1}H NMR spectrum of **22a** in $CDCl_3$ measured at 400 MHz.

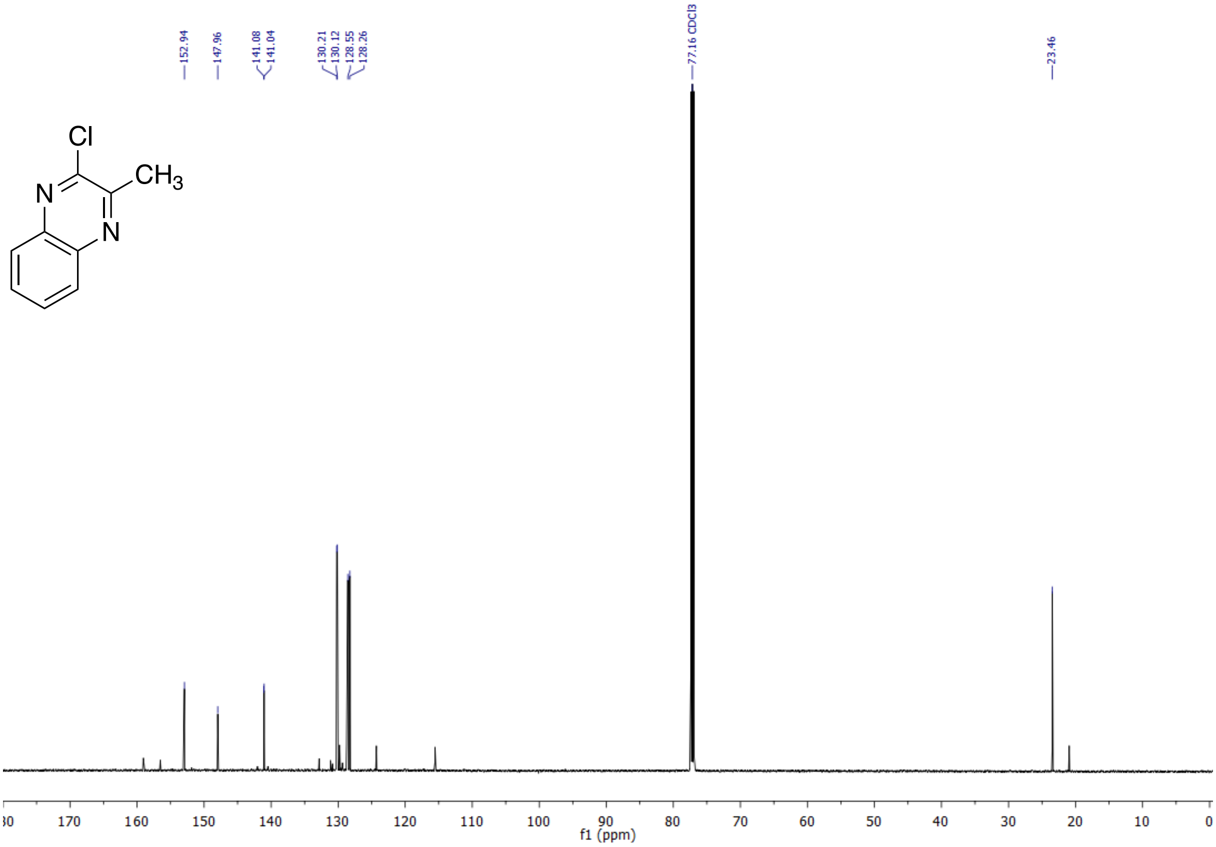

^{13}C NMR spectrum of **22a** in $CDCl_3$ measured at 151 MHz.

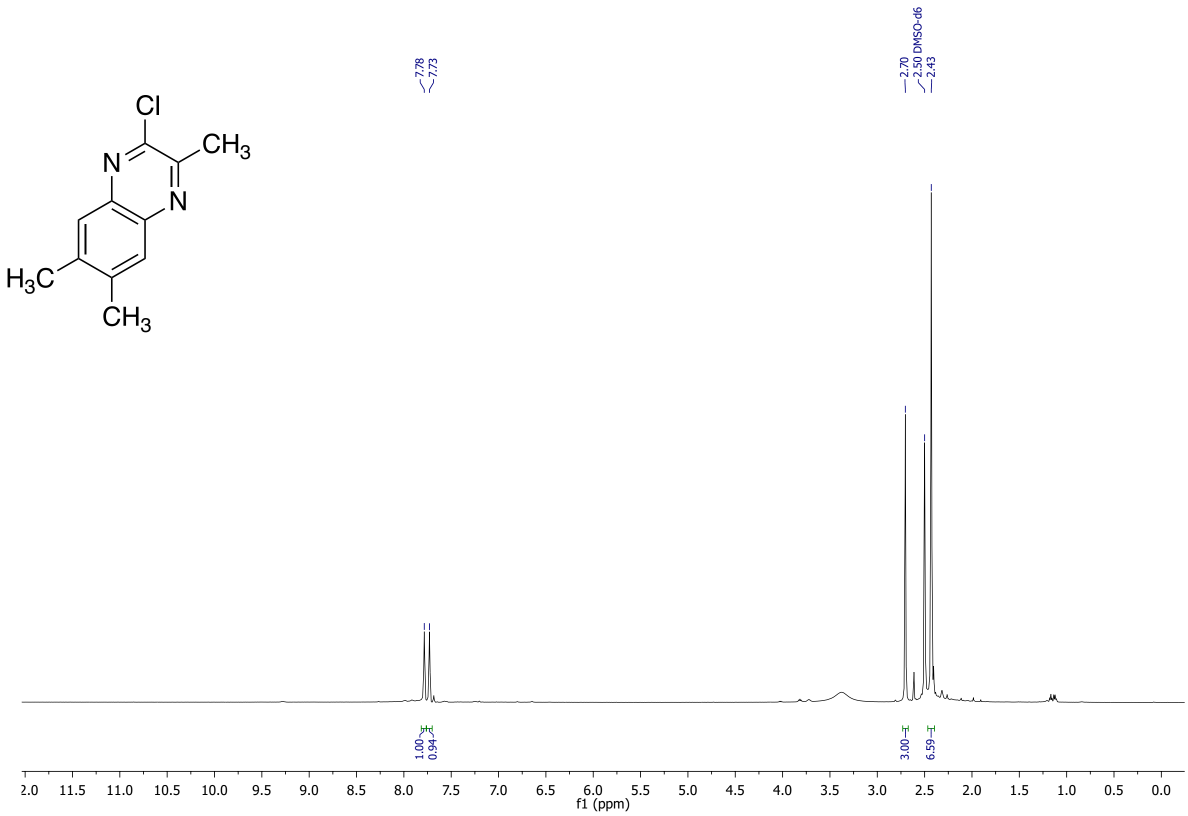

^{1}H NMR spectrum of **22b** in DMSO-d_6 measured at 600 MHz.

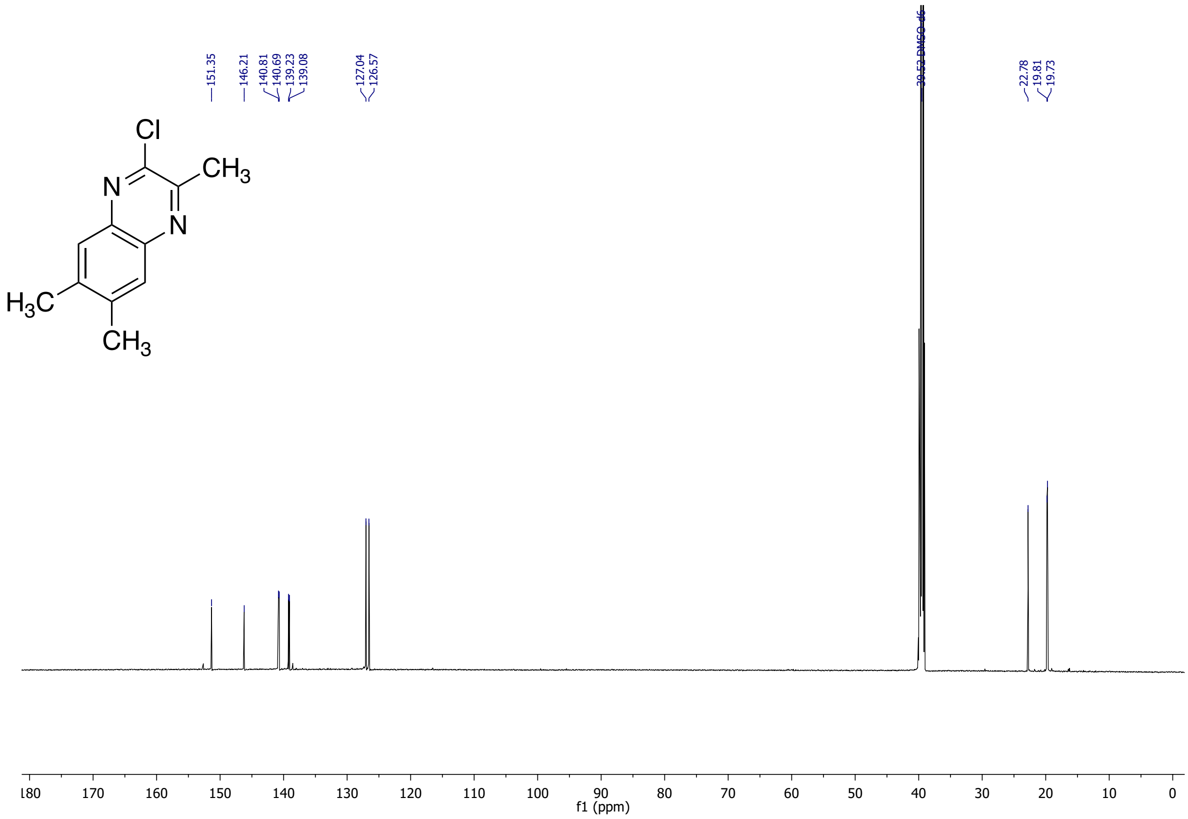

^{13}C NMR spectrum of **22b** in DMSO-d_6 measured at 151 MHz.

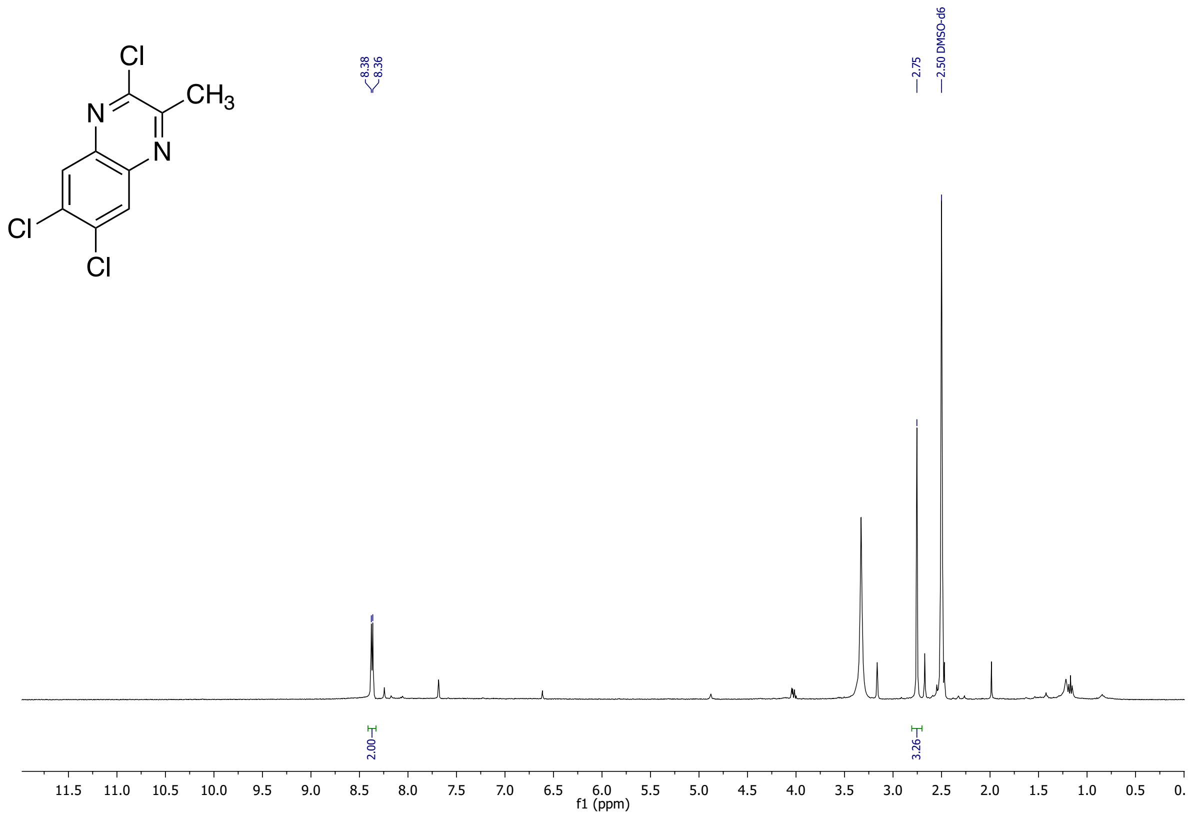

^{1}H NMR spectrum of **22c** in DMSO-d_6 measured at 400 MHz.

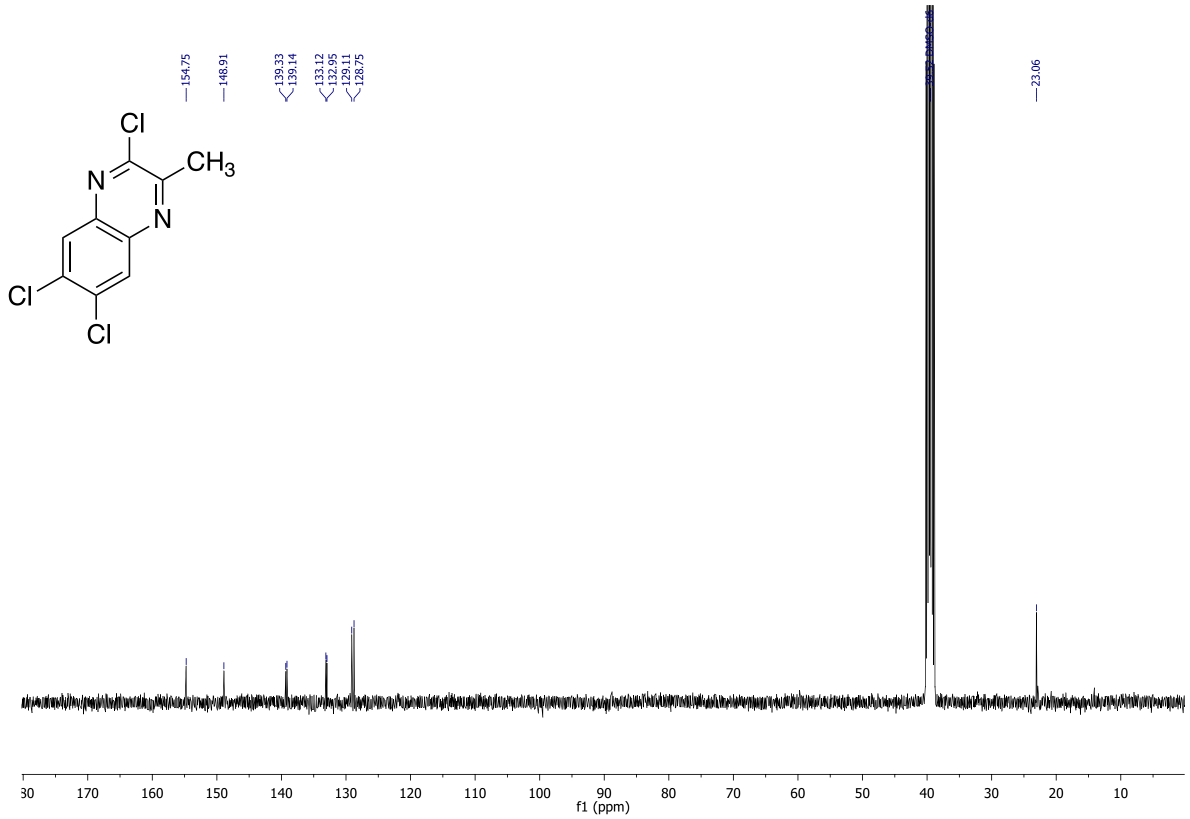

^{13}C NMR spectrum of **22c** in DMSO-d_6 measured at 100 MHz.

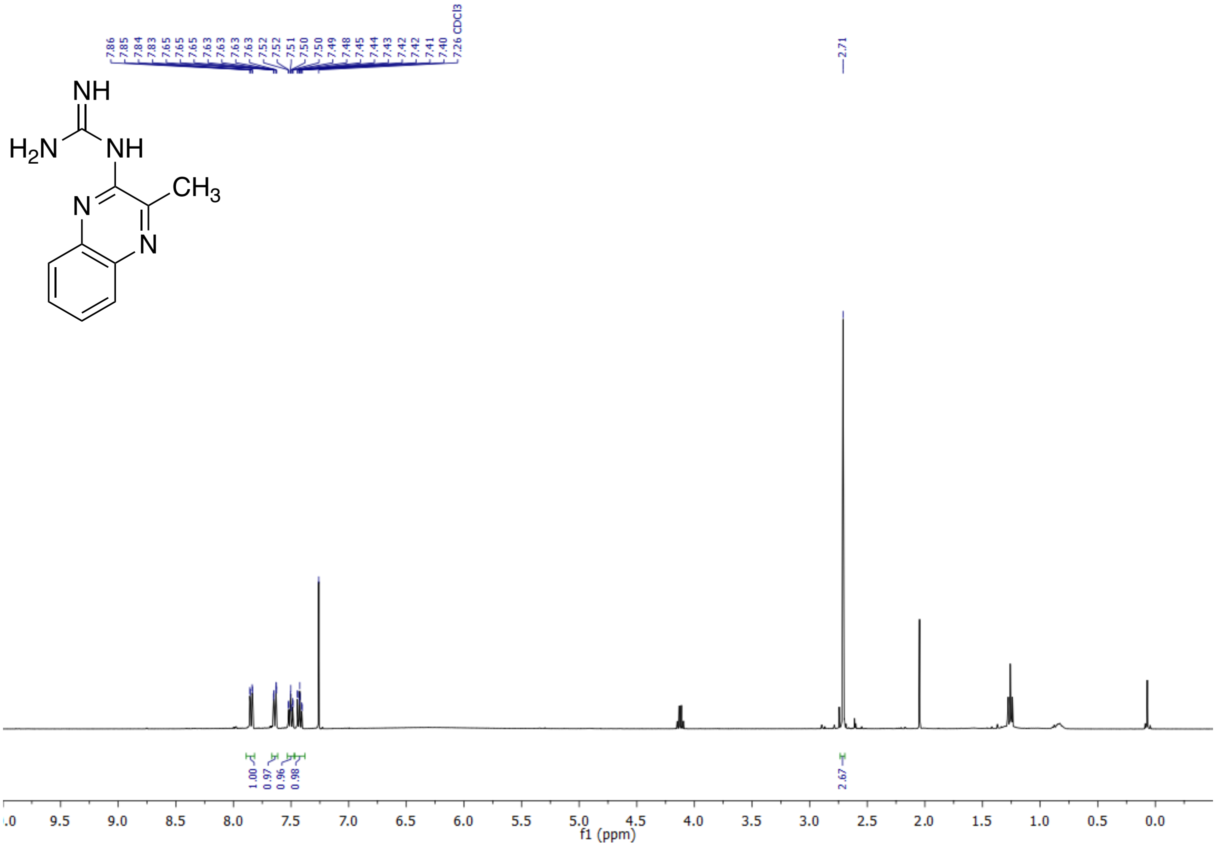

^{1}H NMR spectrum of **23a** in DMSO-d_6 measured at 400 MHz.

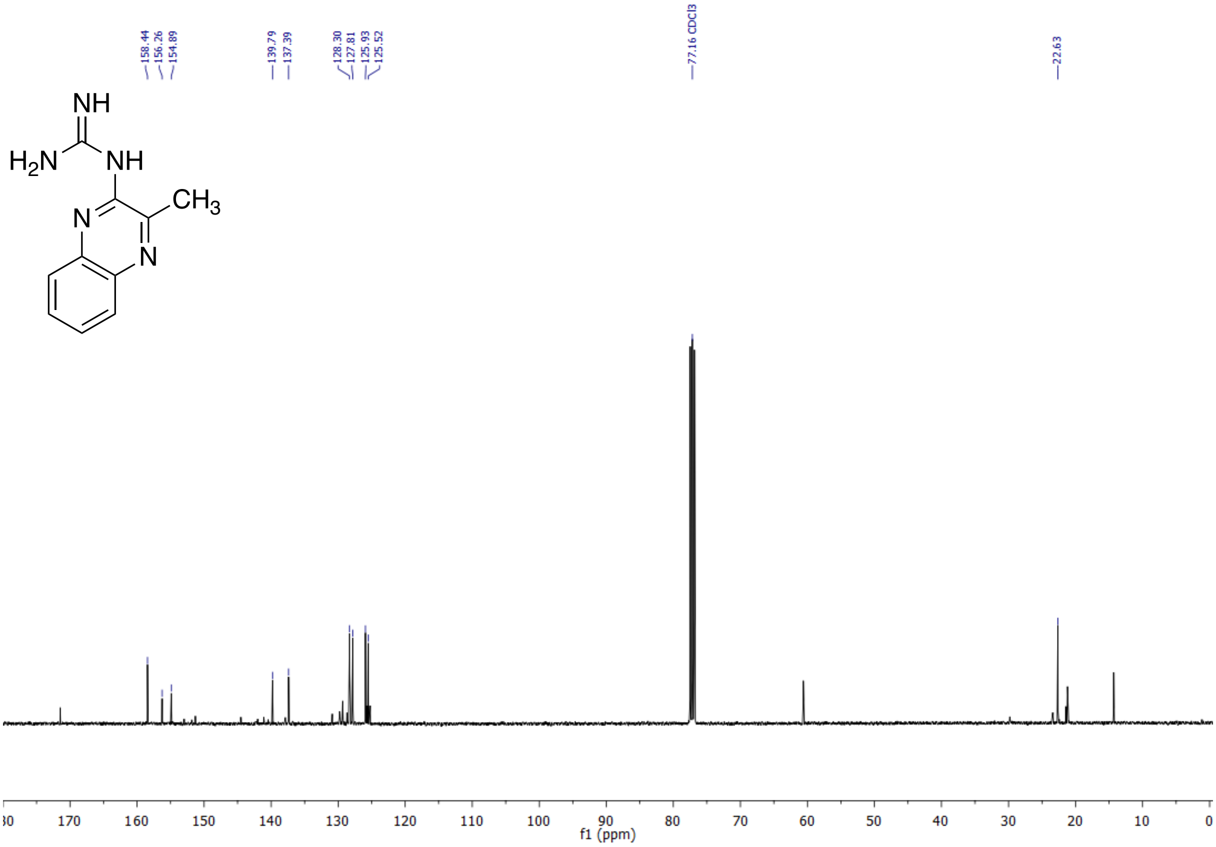

^{13}C NMR spectrum of **23a** in DMSO-d_6 measured at 100 MHz.

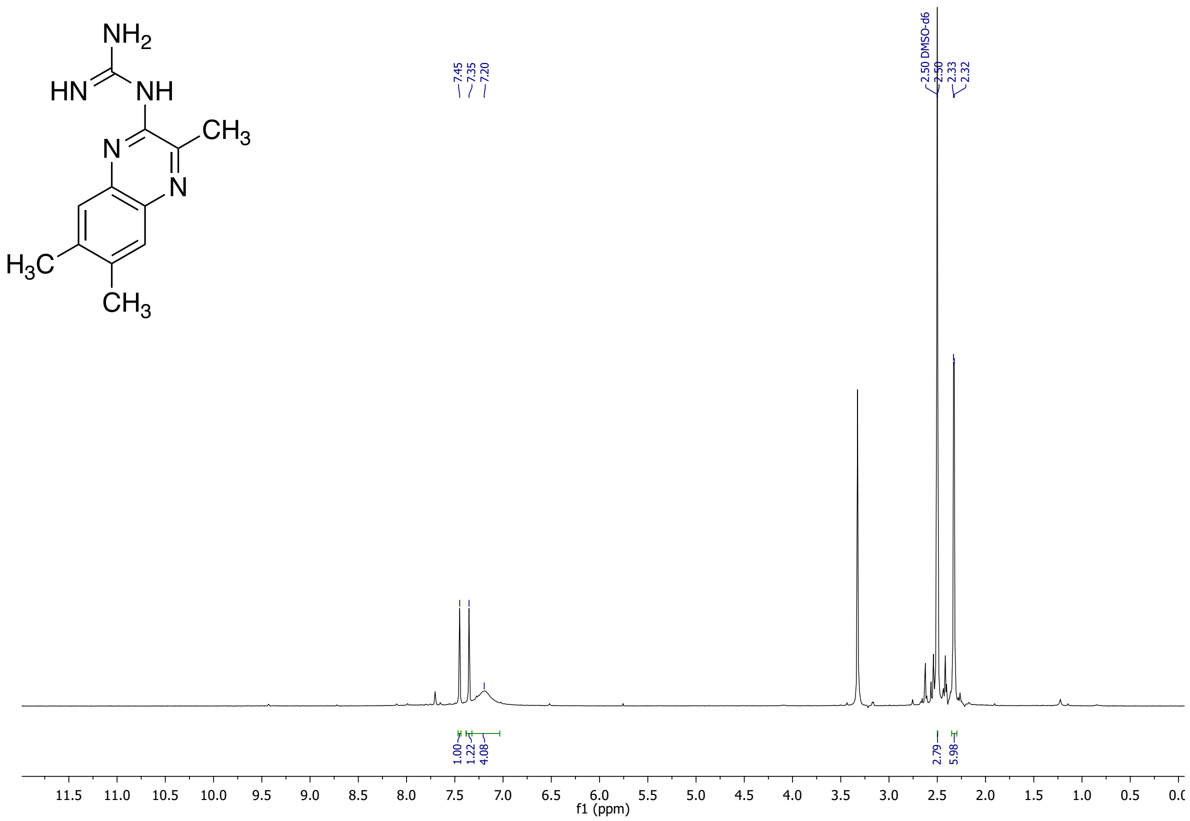

^{1}H NMR spectrum of **23b** in DMSO-d_6 measured at 400 MHz.

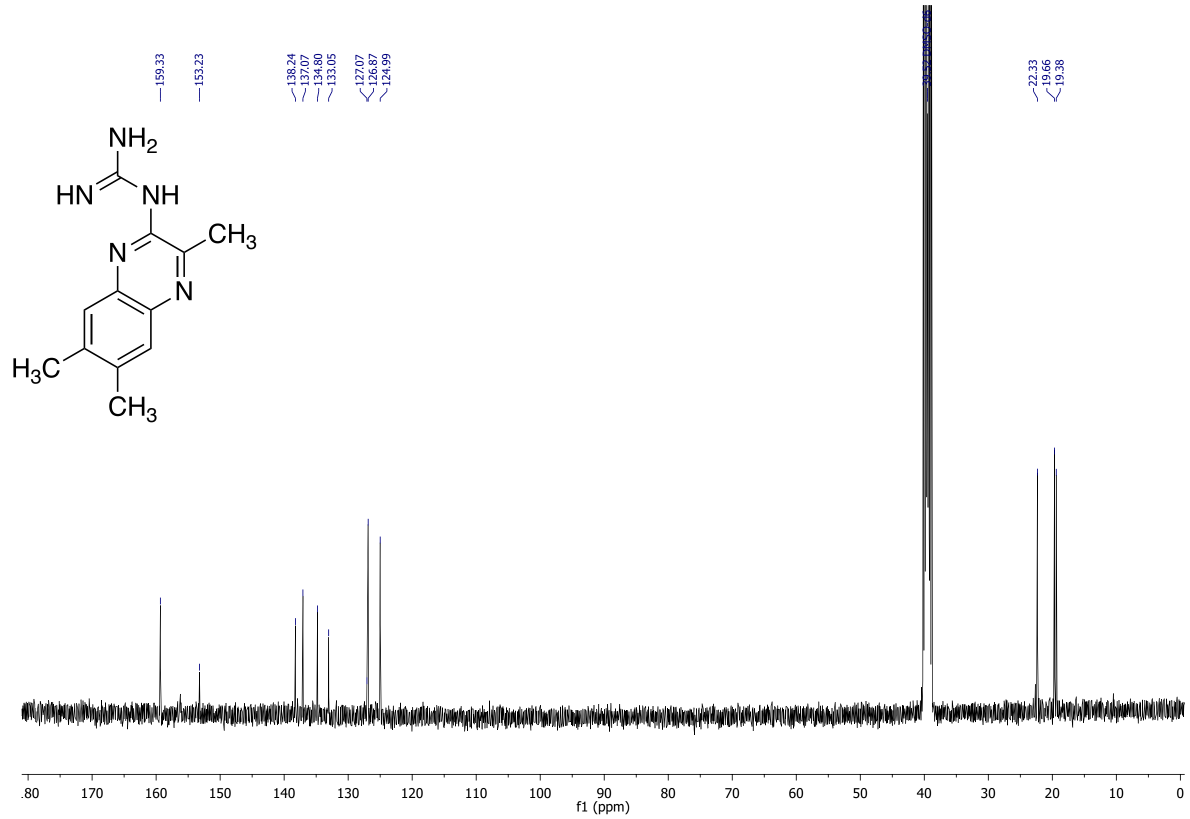

^{13}C NMR spectrum of **23b** in DMSO-d_6 measured at 100 MHz.

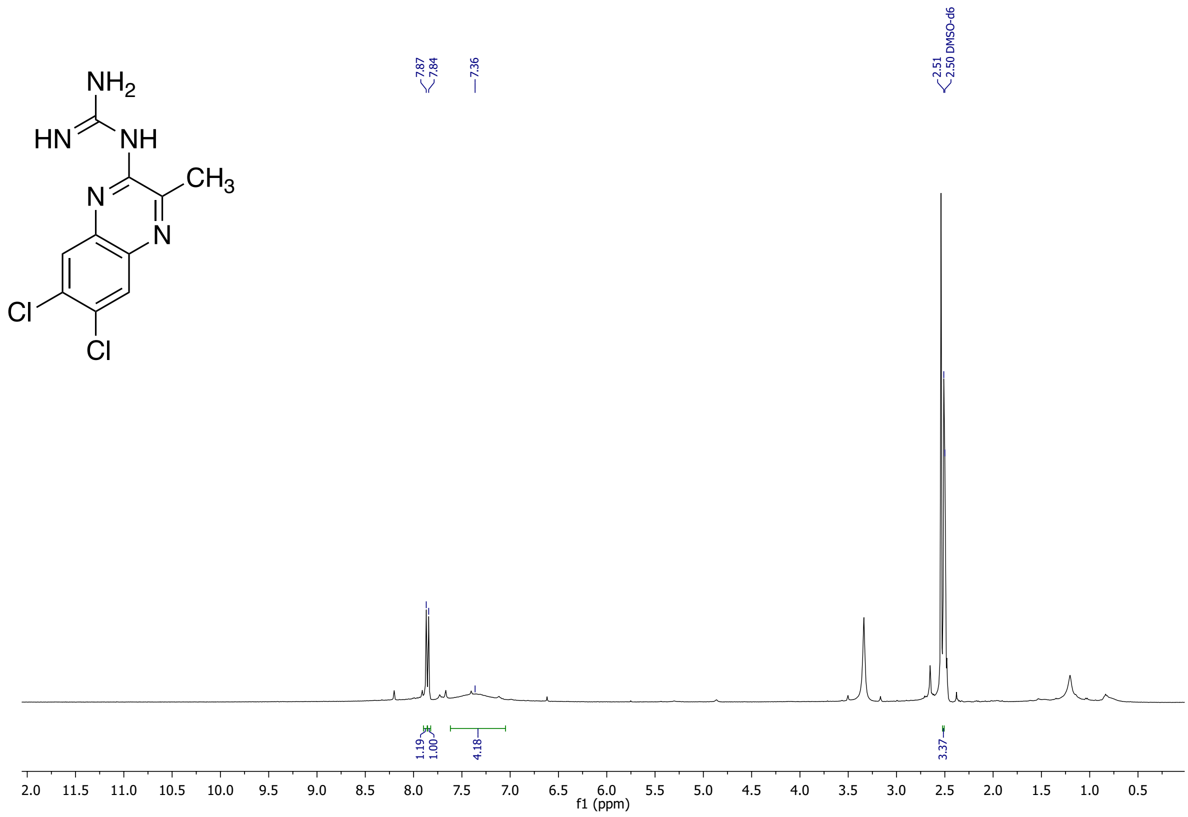

^{1}H NMR spectrum of **23c** in DMSO-d_6 measured at 400 MHz.

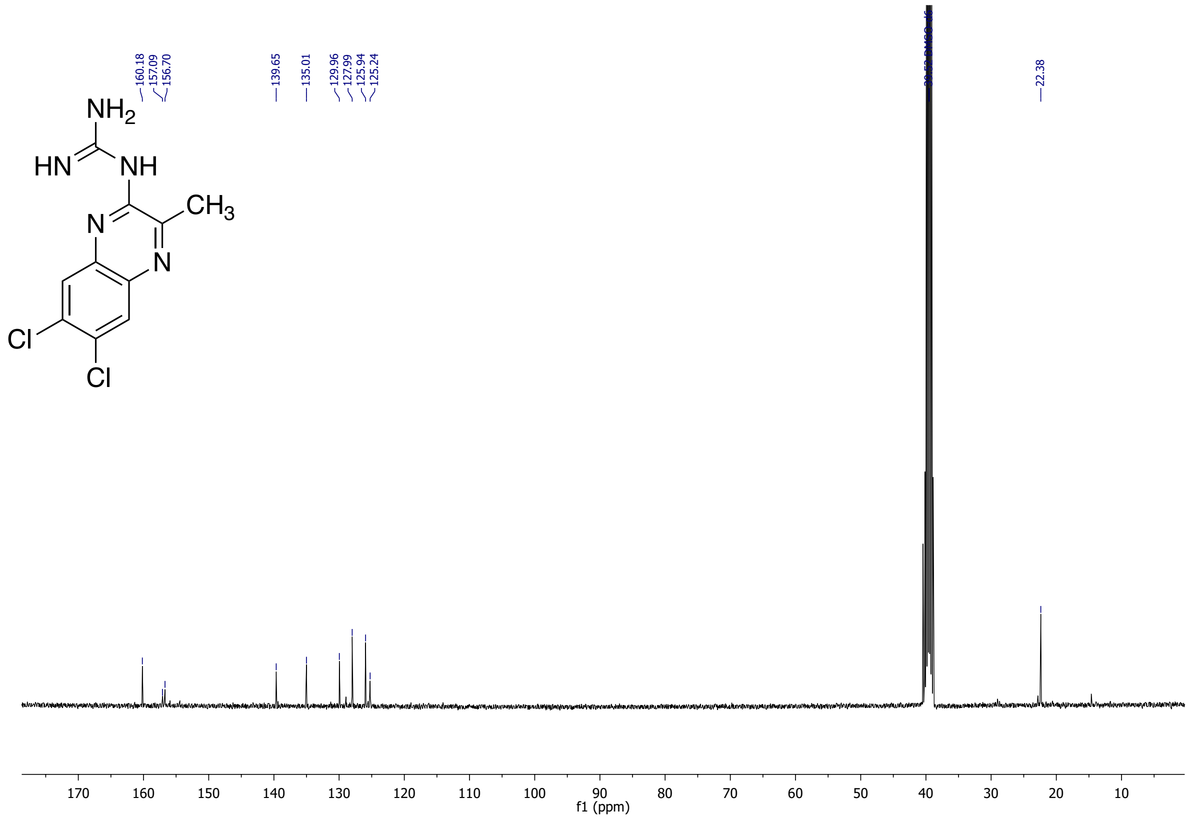

^{13}C NMR spectrum of **23c** in DMSO-d_6 measured at 100 MHz.

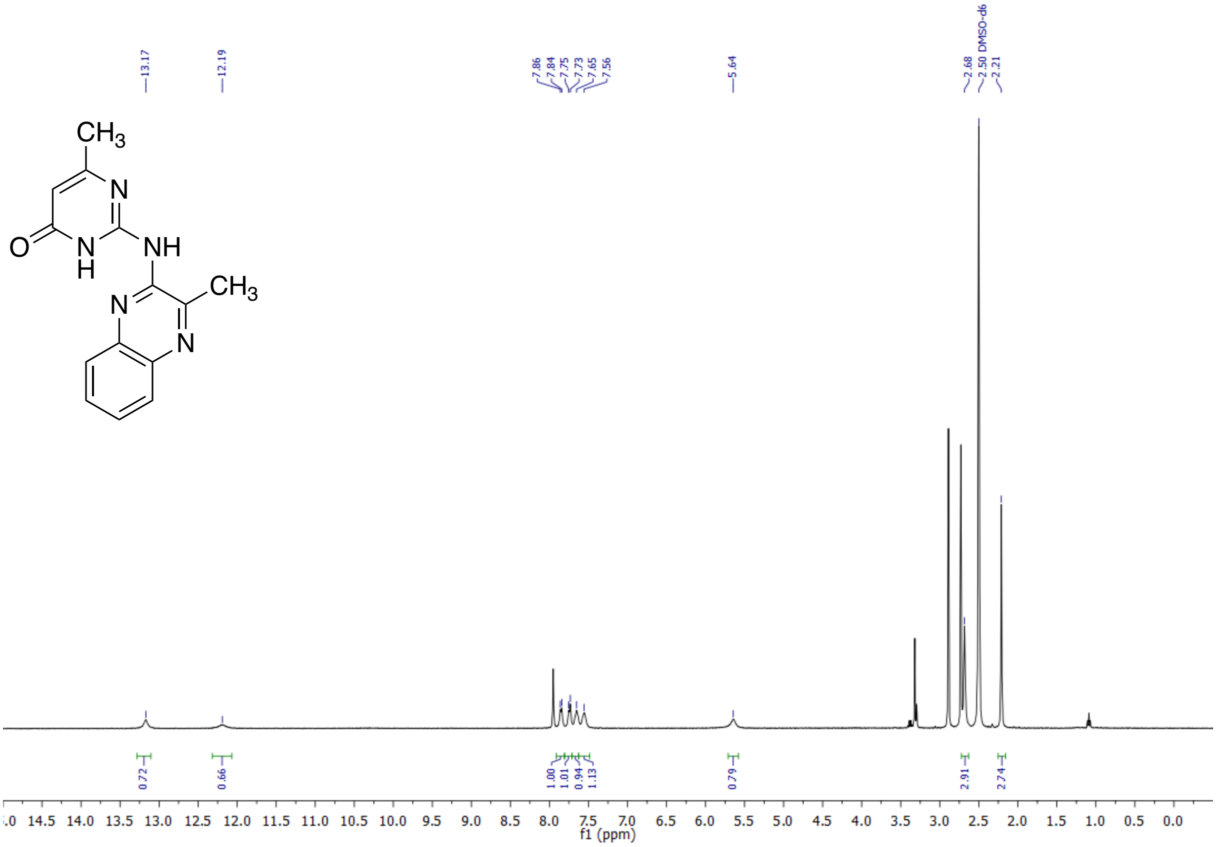

^{1}H NMR spectrum of **24a** in DMSO-d_6 measured at 400 MHz.

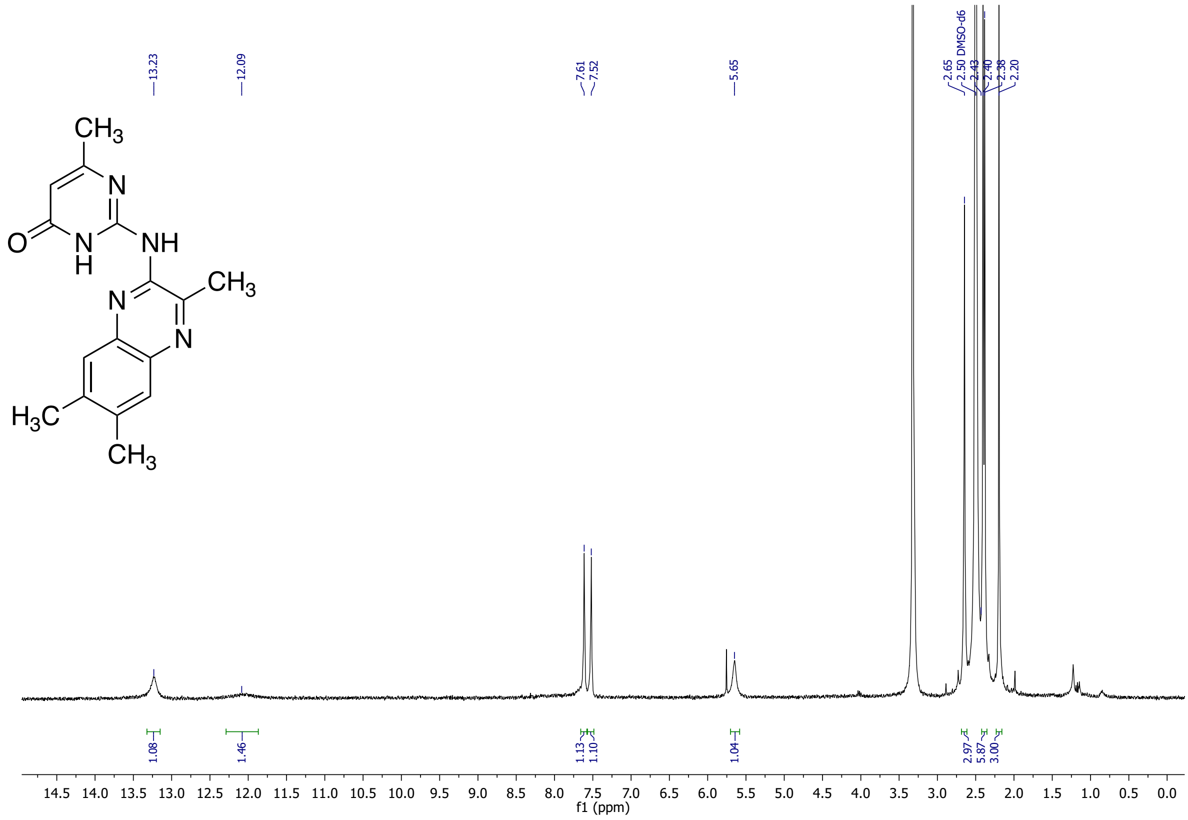

^{1}H NMR spectrum of **24b** in DMSO-d_6 measured at 400 MHz.

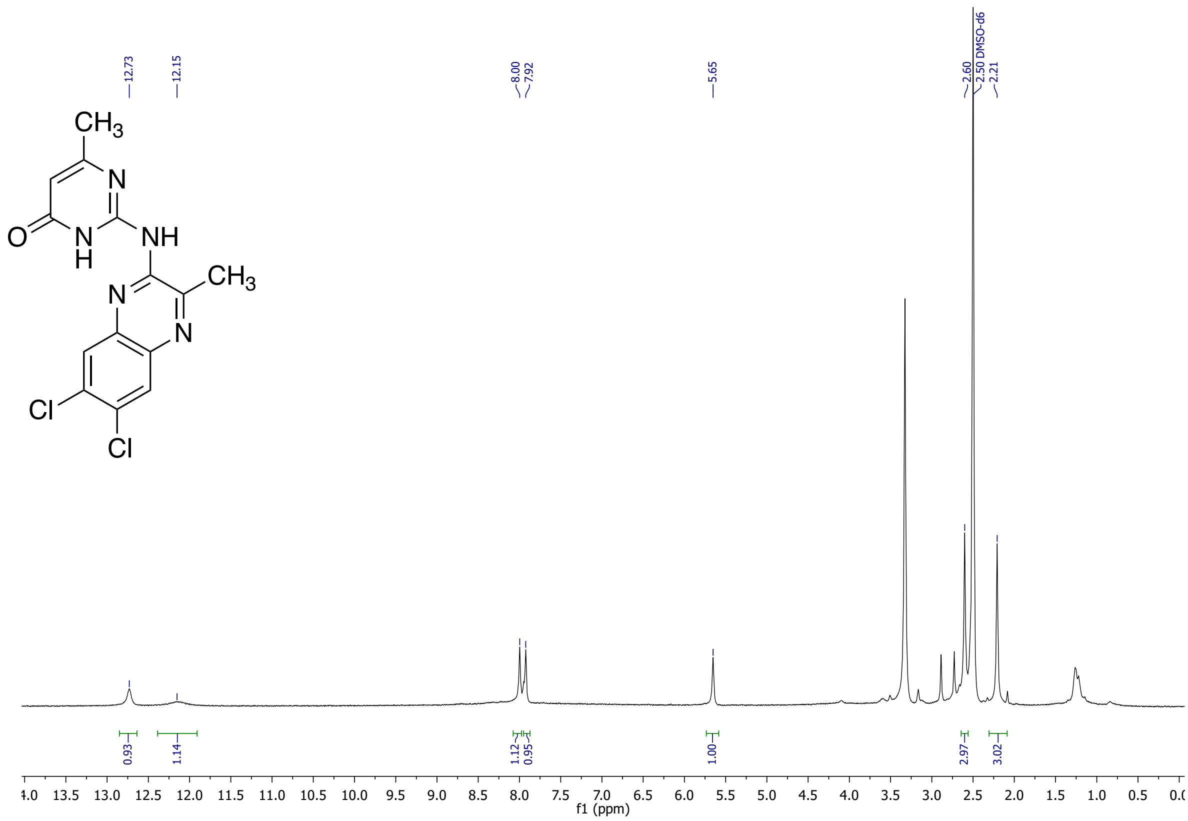

^{1}H NMR spectrum of **24c** in DMSO-d_6 measured at 400 MHz.

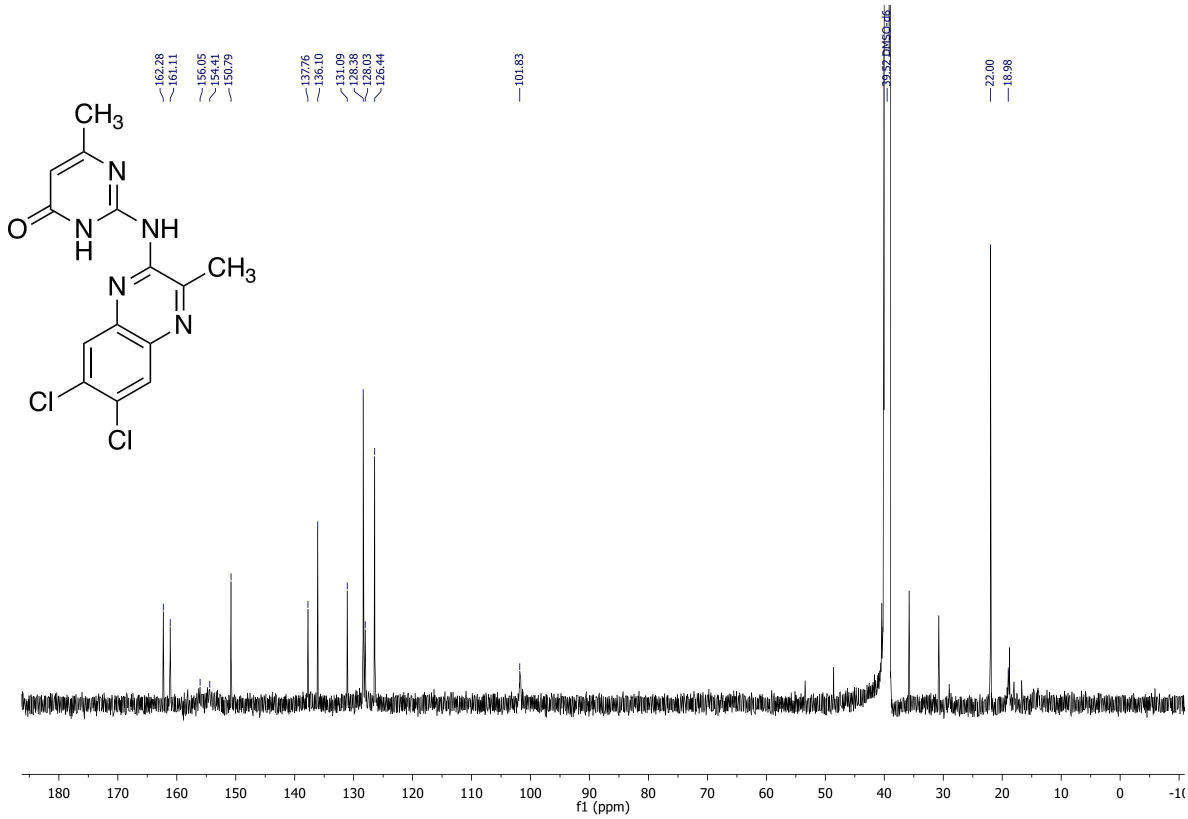

^{13}C NMR spectrum of **24c** in DMSO-d_6 measured at 151 MHz.

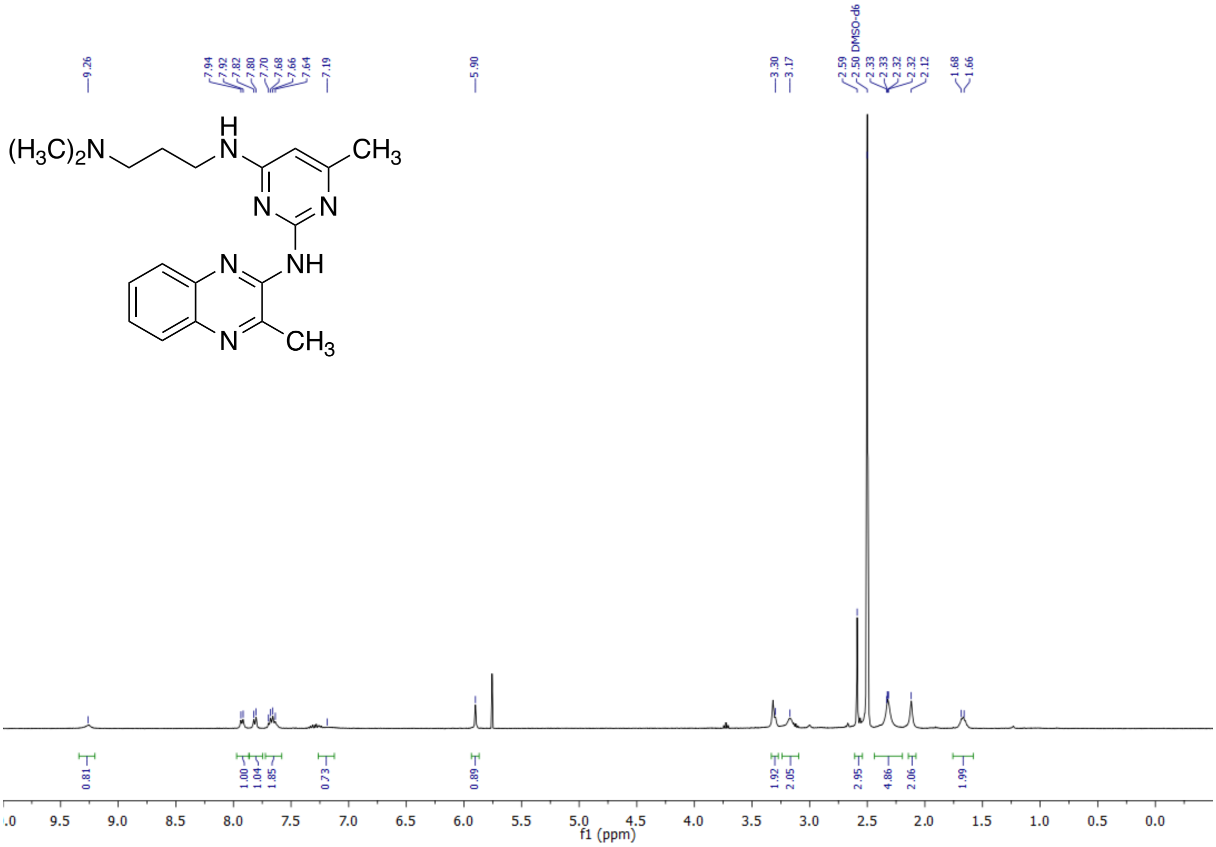

^{1}H NMR spectrum of **9** in DMSO-d_6 measured at 400 MHz.

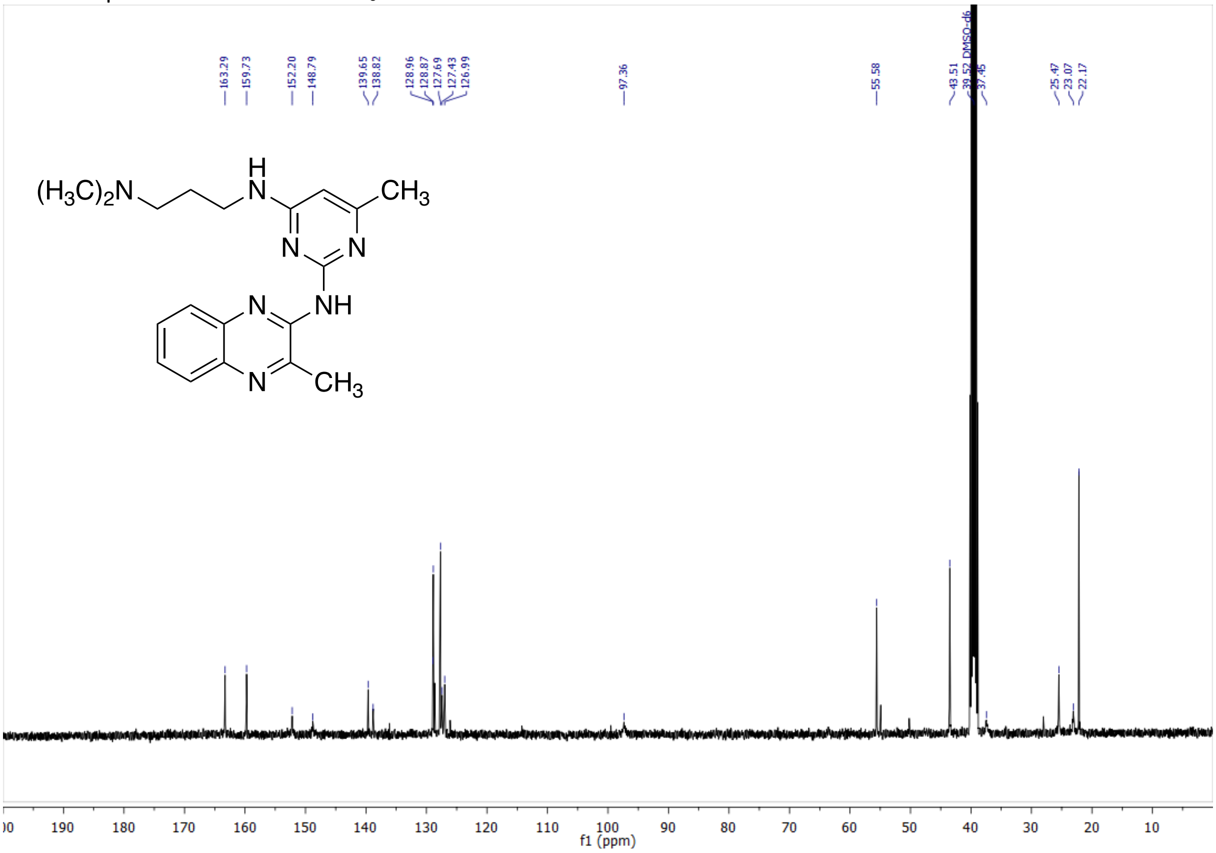

^{13}C NMR spectrum of **9** in DMSO-d_6 measured at 100 MHz.

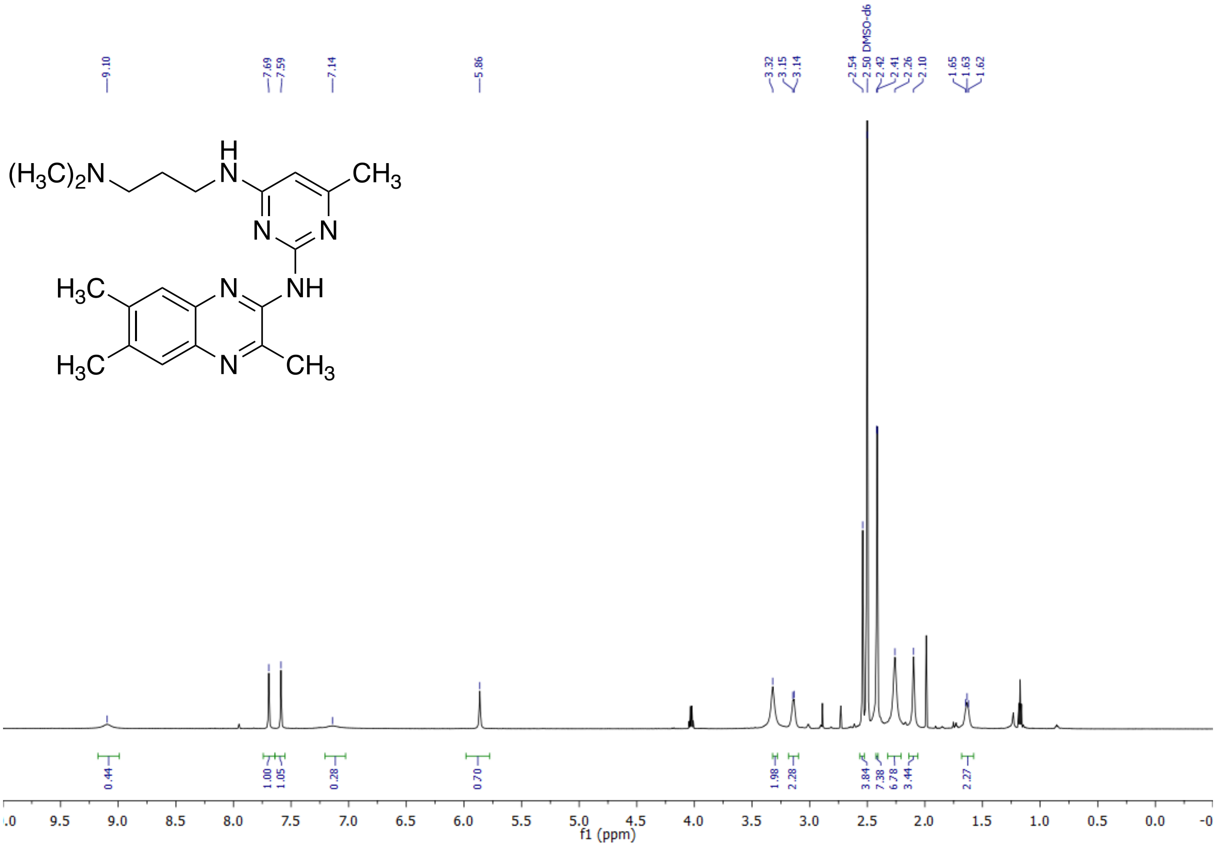

^{1}H NMR spectrum of **10** in DMSO-d_6 measured at 600 MHz.

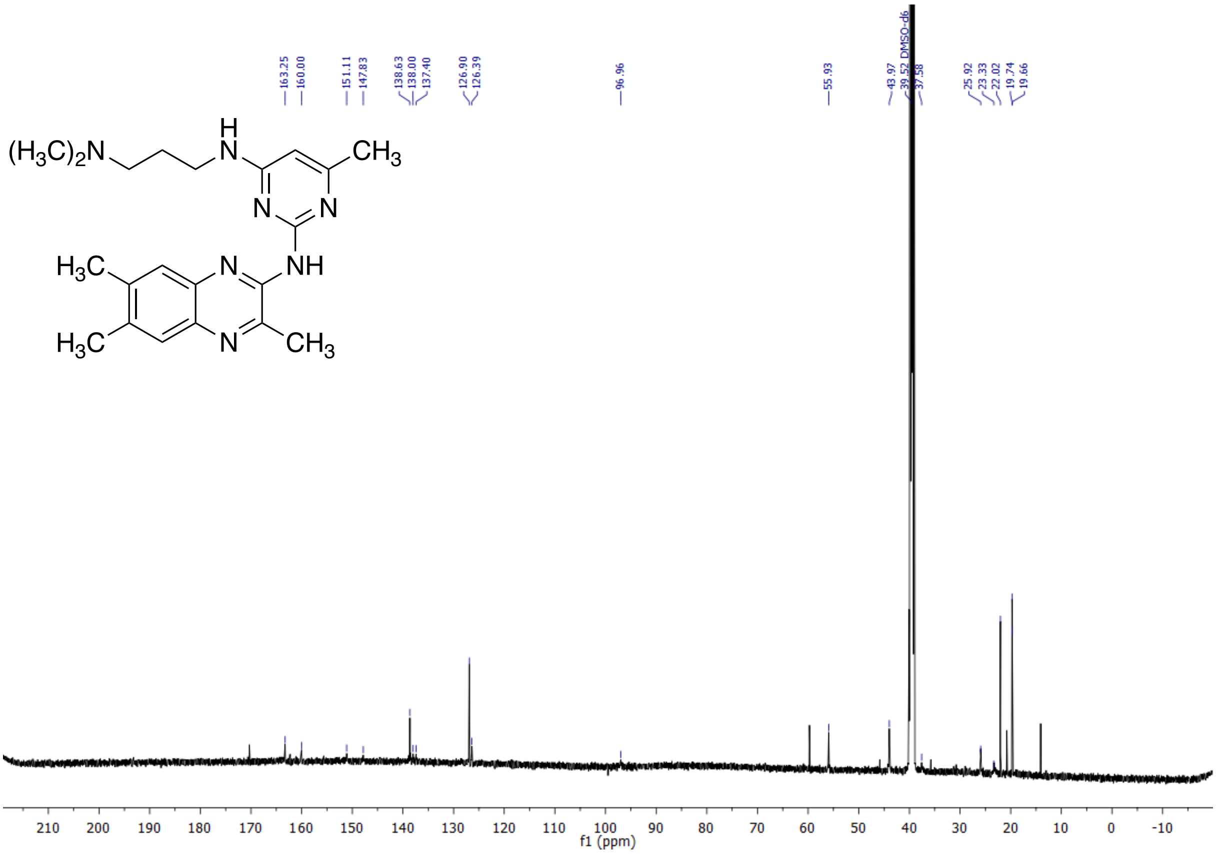

^{13}C NMR spectrum of **10** in DMSO-d_6 measured at 151 MHz.

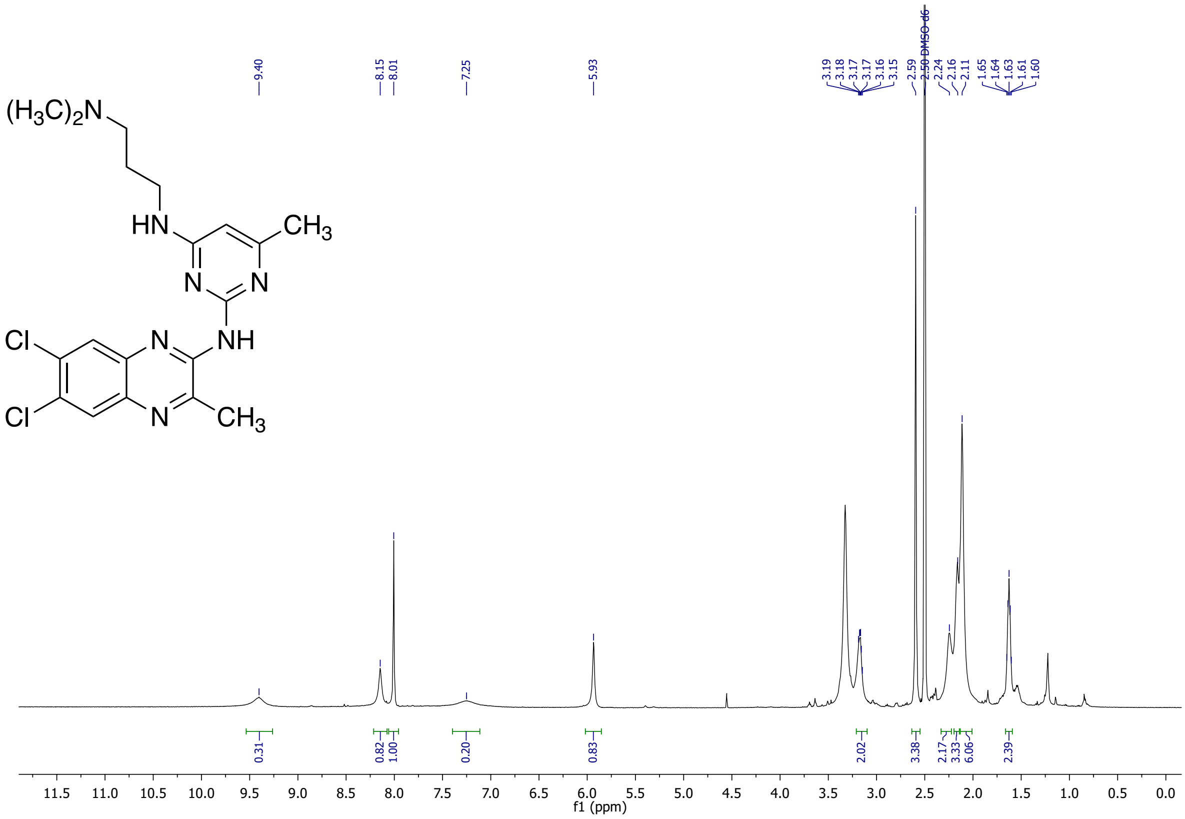

^{1}H NMR spectrum of **11** in DMSO-d_6 measured at 600 MHz.

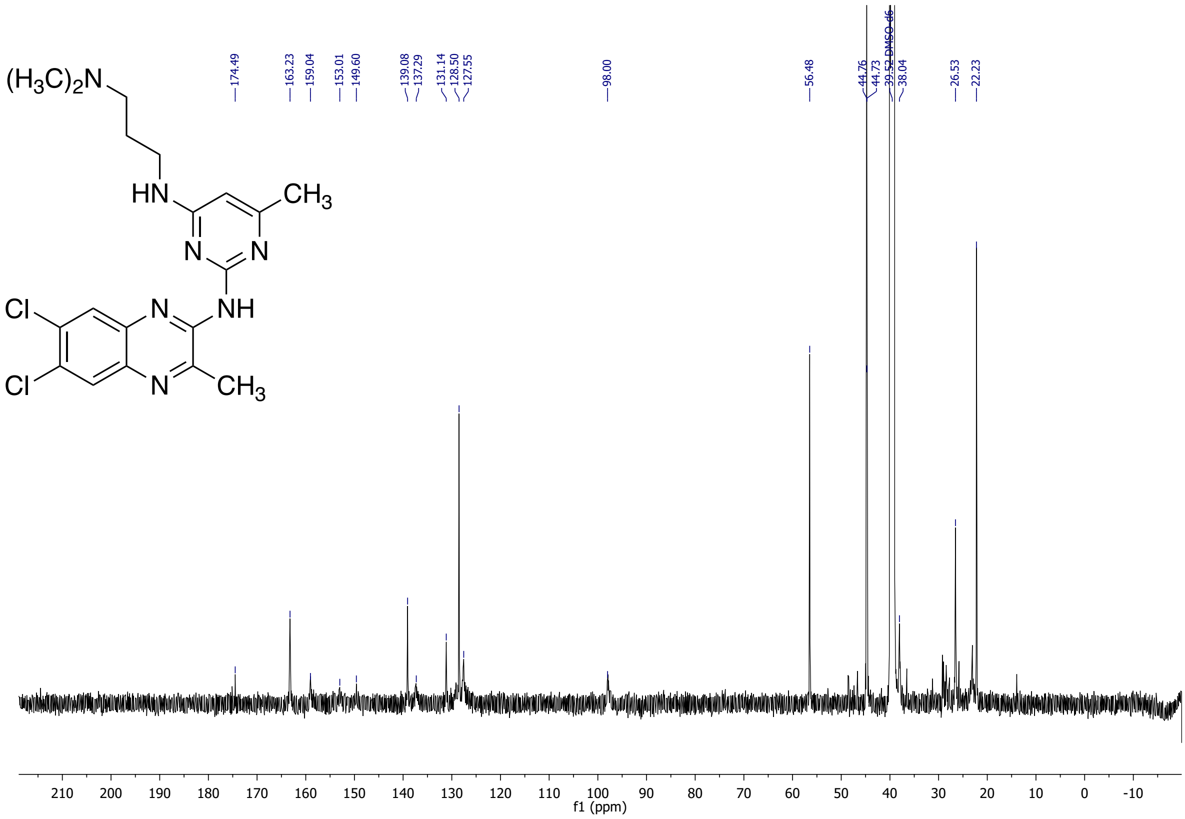

^{13}C NMR spectrum of **11** in DMSO-d_6 measured at 151 MHz. *(> 3000 scans with a concentrated sample resulted in similar resolution of the broadened carbon peaks.)*

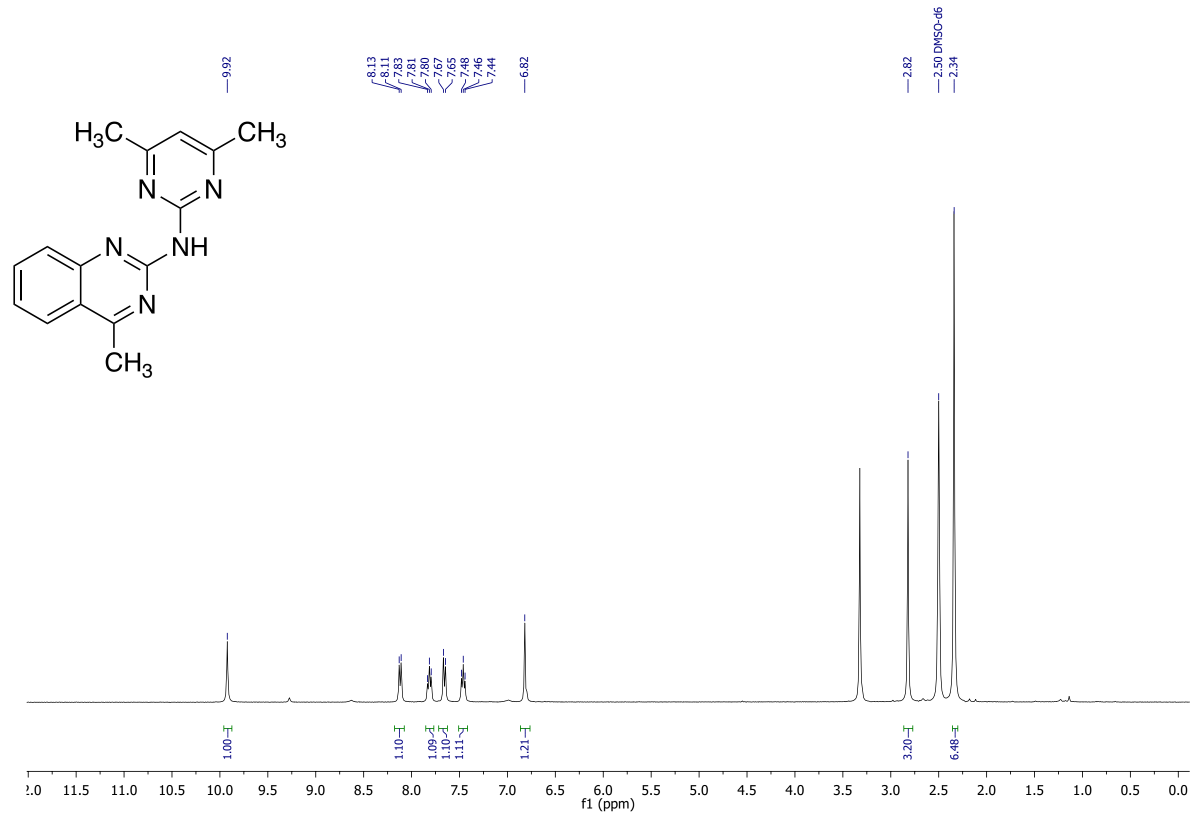

^{1}H NMR spectrum of **25** in DMSO-d_6 measured at 400 MHz.

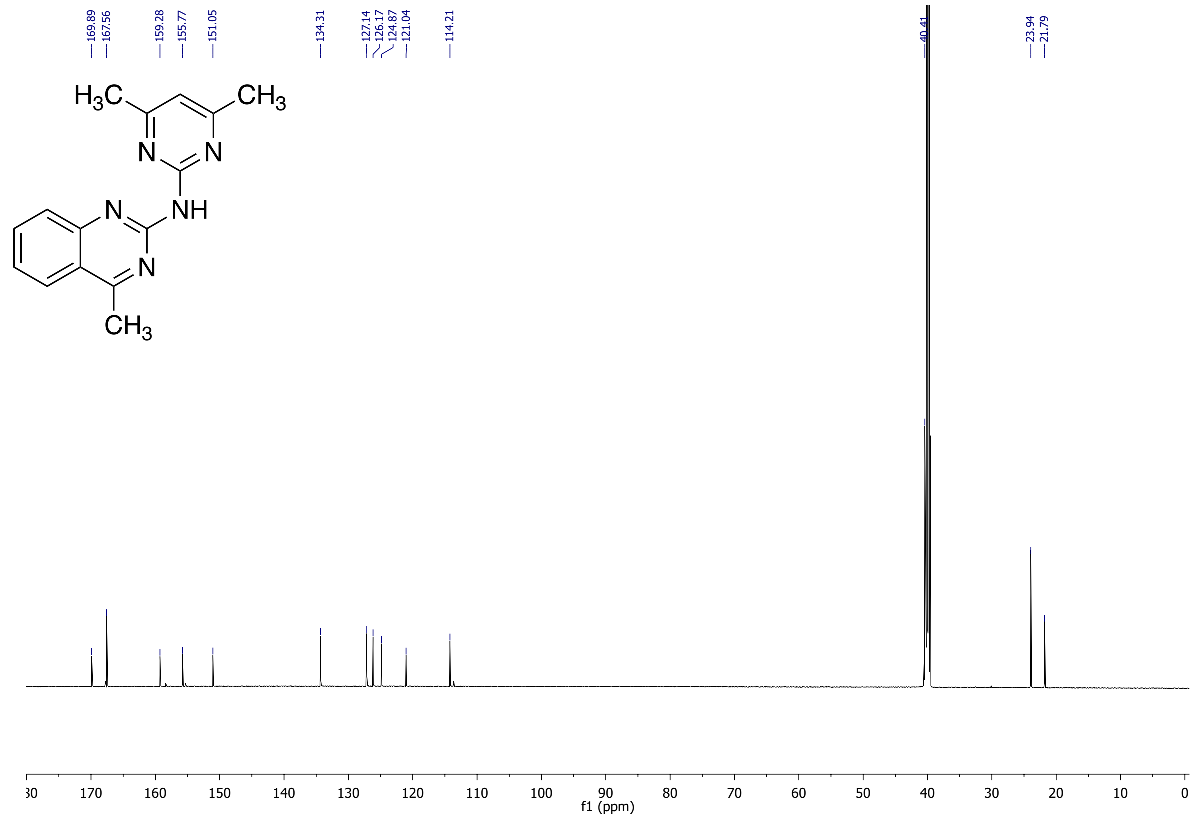

^{13}C NMR spectrum of **25** in DMSO-d_6 measured at 151 MHz.

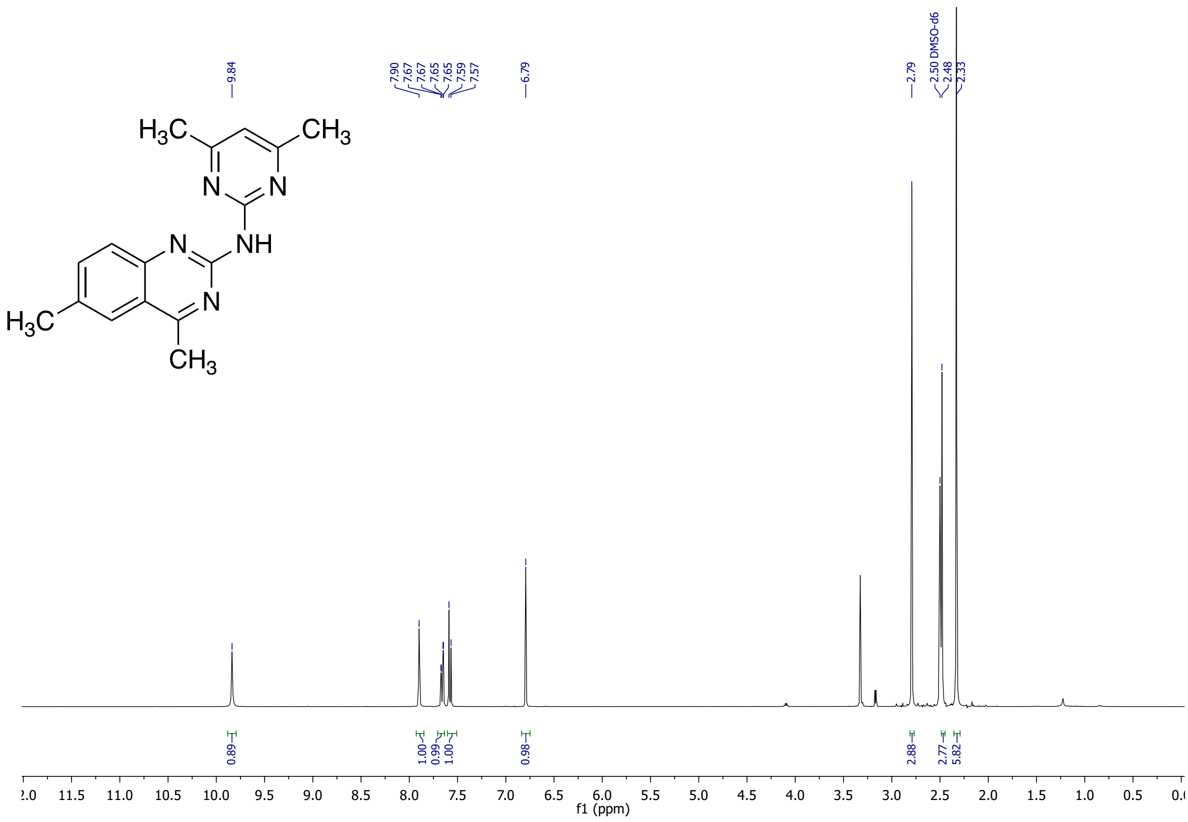

^{1}H NMR spectrum of **26** in DMSO-d_6 measured at 400 MHz.

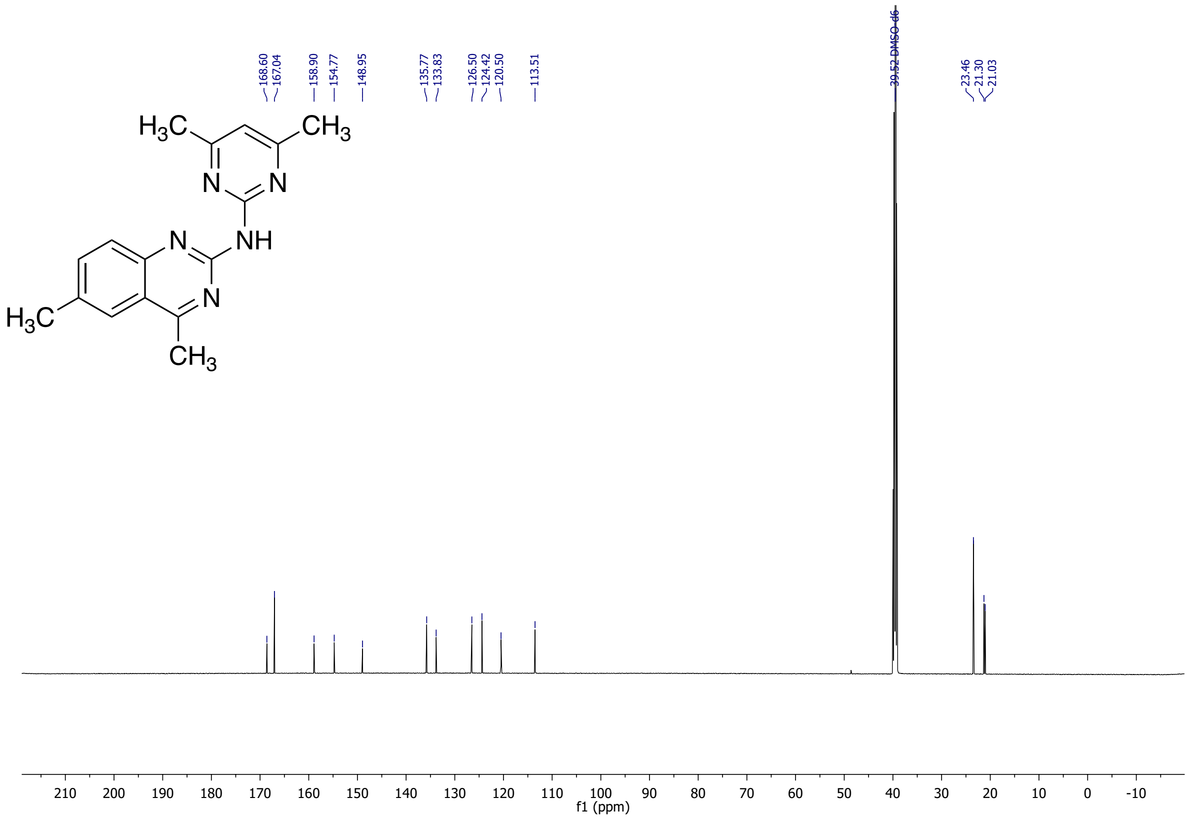

^{13}C NMR spectrum of **26** in DMSO-d_6 measured at 151 MHz.

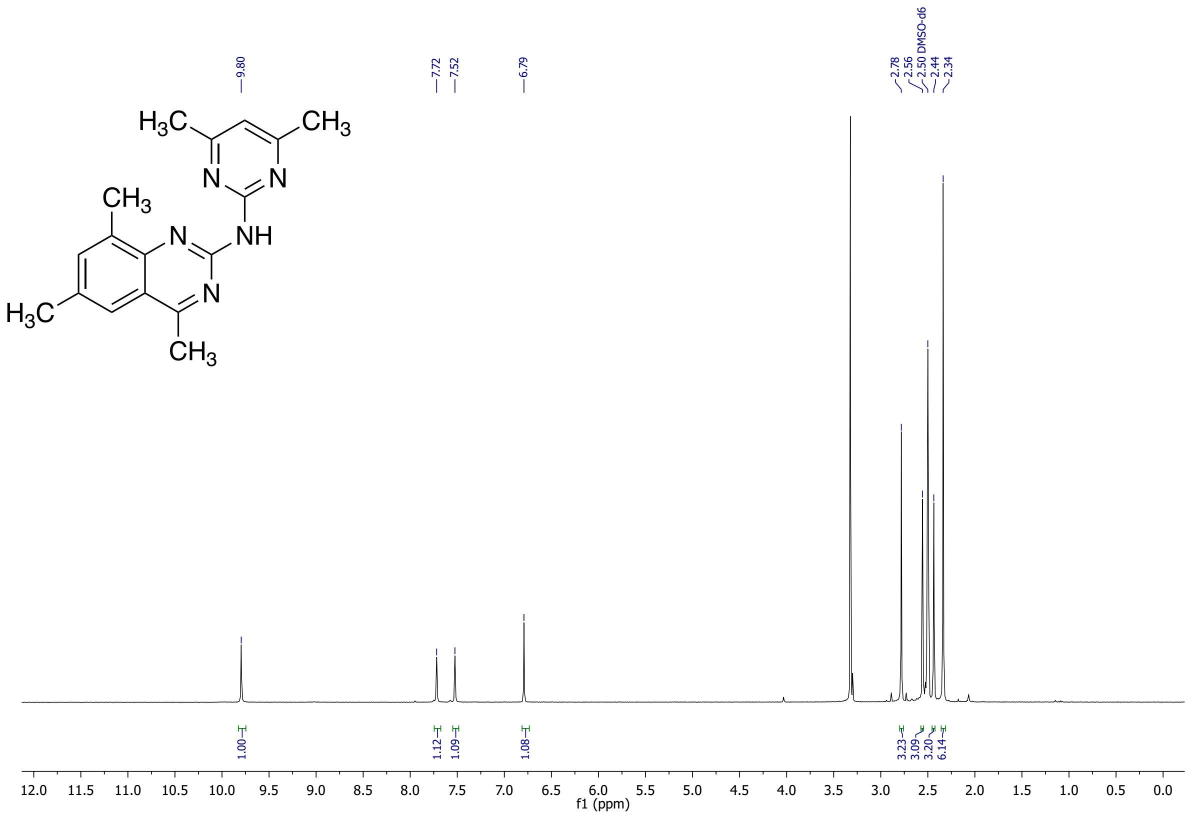

^{1}H NMR spectrum of **27** in DMSO-d_6 measured at 400 MHz.

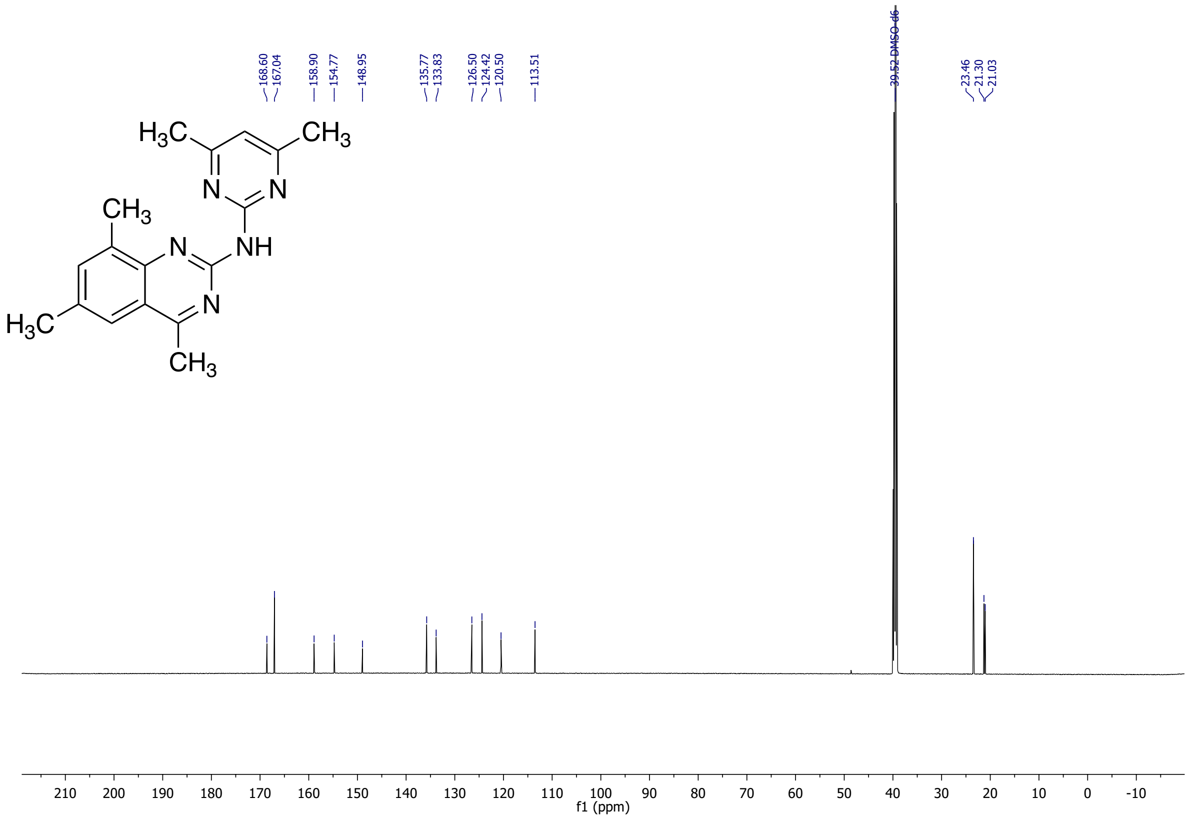

^{13}C NMR spectrum of **27** in DMSO-d_6 measured at 151 MHz.

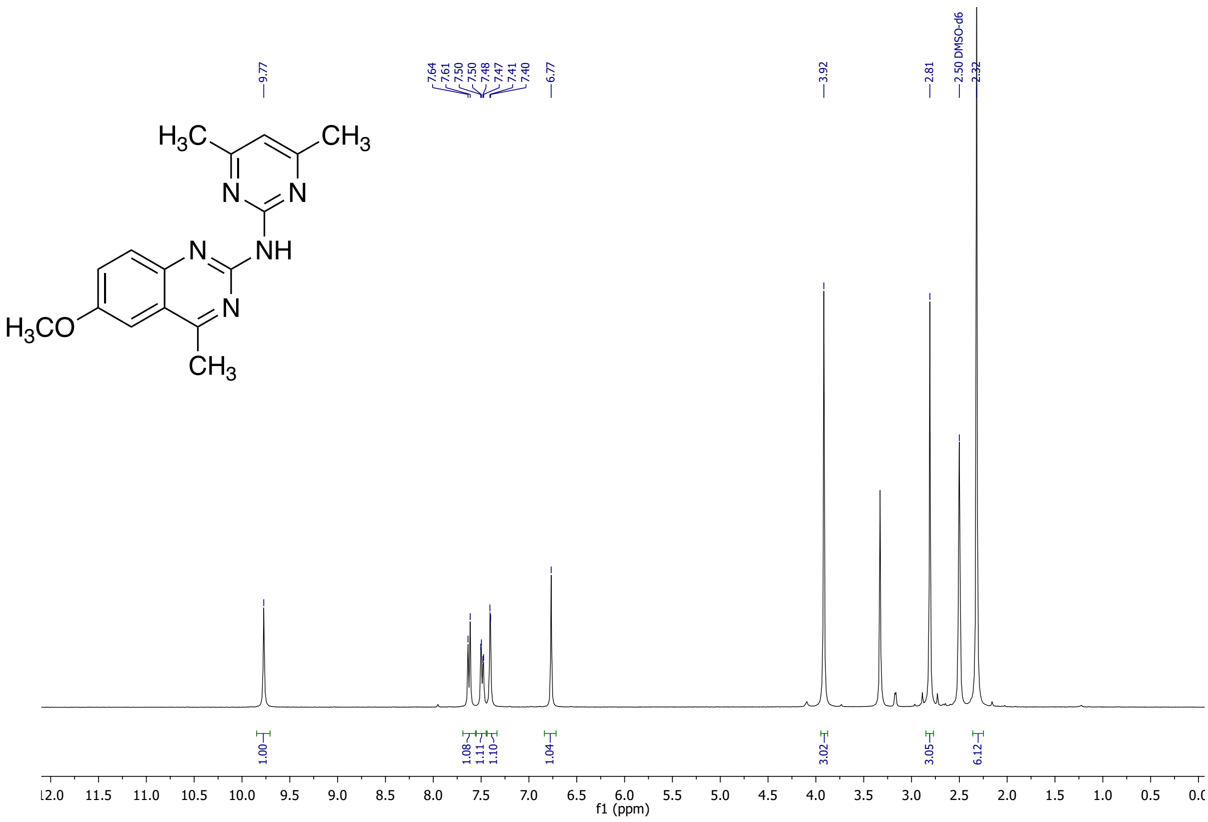

^{1}H NMR spectrum of **28** in DMSO-d_6 measured at 400 MHz.

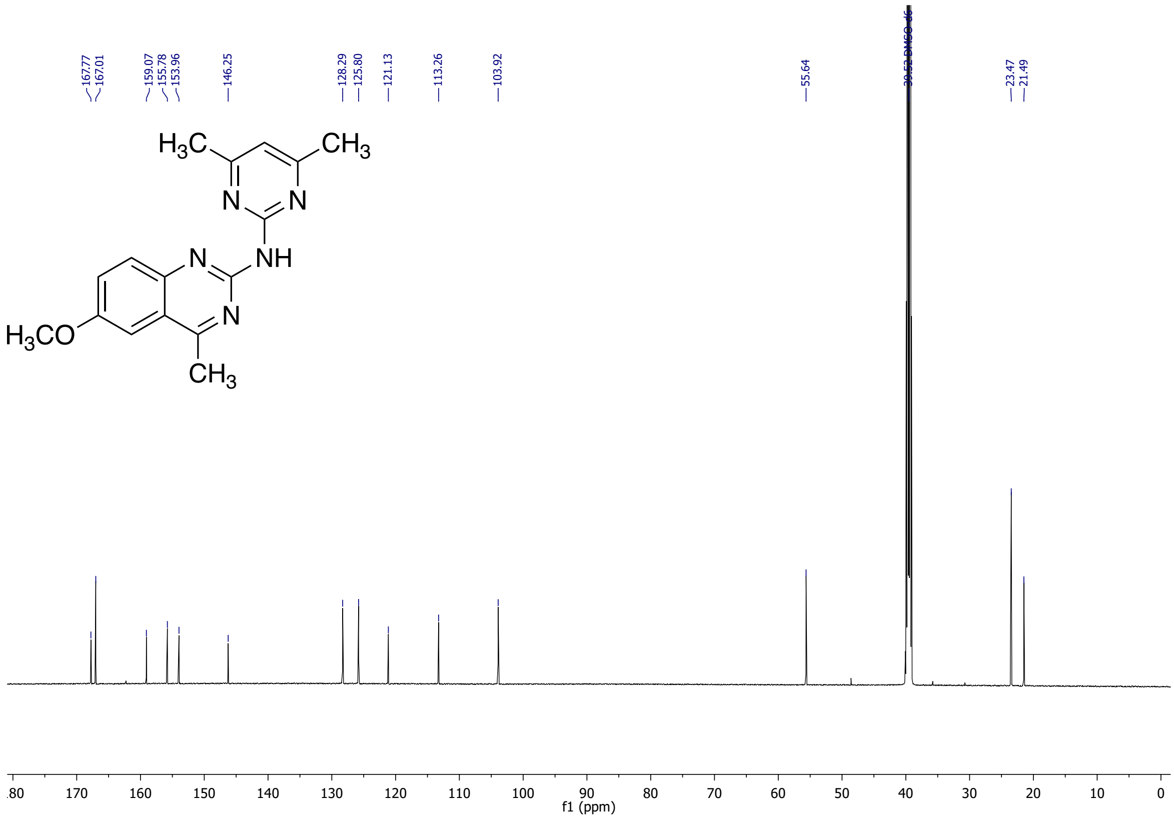

^{13}C NMR spectrum of **28** in DMSO-d_6 measured at 151 MHz.

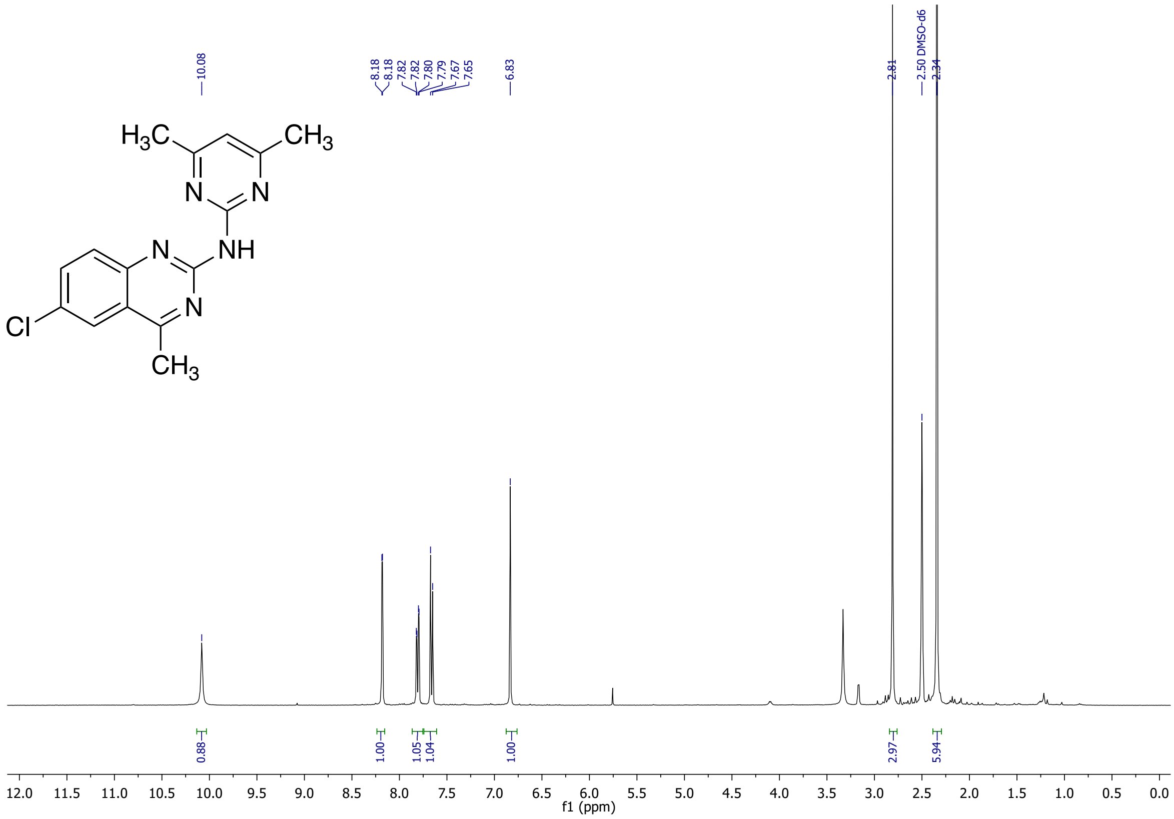

^{1}H NMR spectrum of **29** in DMSO-d_6 measured at 400 MHz.

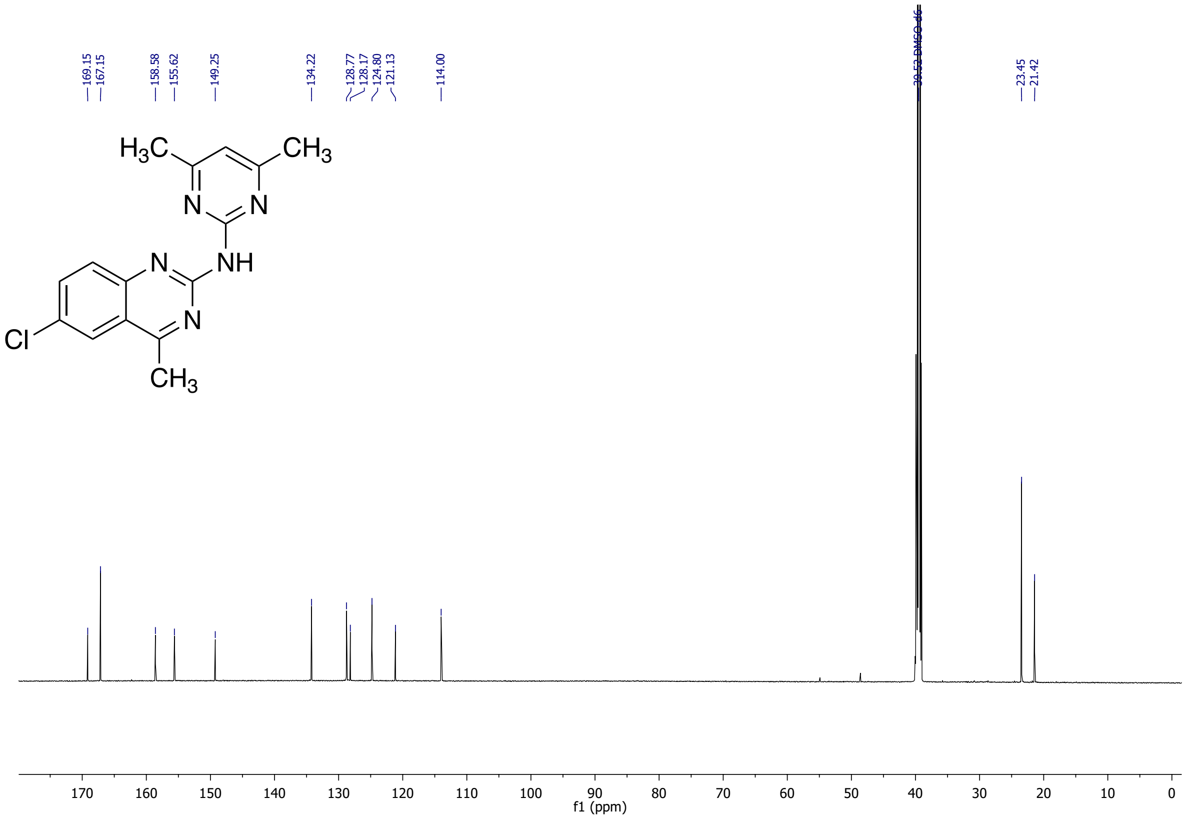

^{13}C NMR spectrum of **29** in DMSO-d_6 measured at 151 MHz.

HPLC traces of tested compounds

UV trace: **Blank** (DMSO) for compounds **2-3**, **6-11**, **25-29**

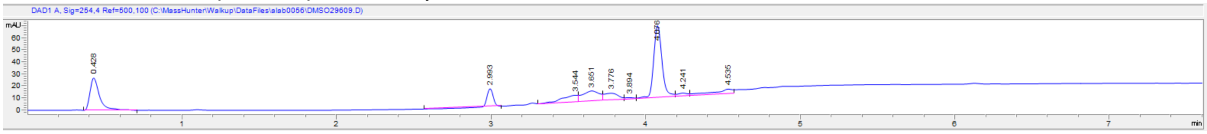

The peaks from the blank are subtracted from the UV traces of the compounds.

UV trace: **Blank** (DMSO) for compounds **4** and **5**

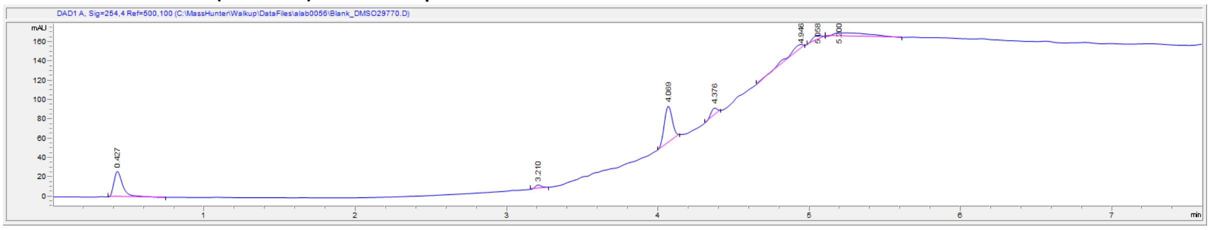

The peaks from the blank are subtracted from the UV traces of compounds.

UV trace: **2**

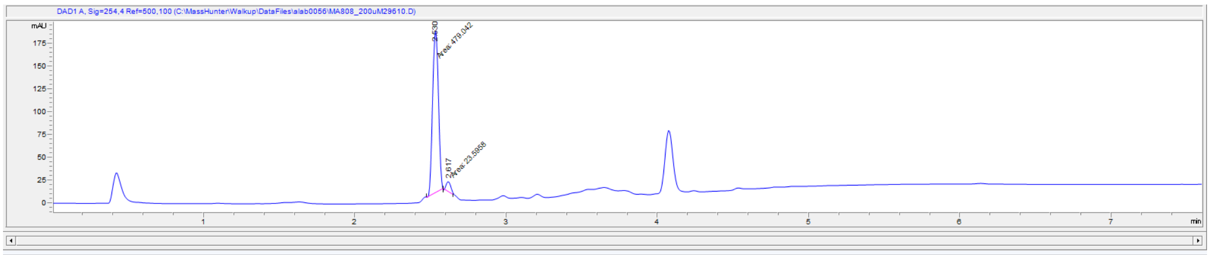

#	Time	Type	Area	Height	Width	Area%	Symmetry
1	2.53	MM	479	179.6	0.0444	95.306	1.003
2	2.617	MM	23.6	11	0.0358	4.694	0.599

UV trace: **3**

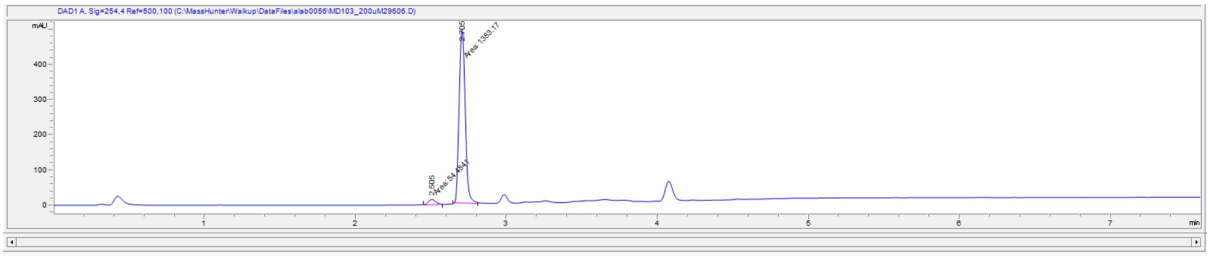

#	Time	Type	Area	Height	Width	Area%	Symmetry
1	2.505	MM	54.5	15.6	0.0582	3.790	0.902
2	2.705	MM	1383.2	495.4	0.0465	96.210	0.906

UV trace: **4**

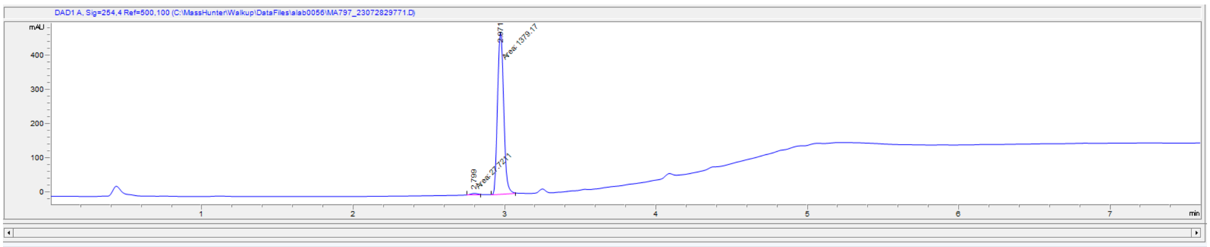

#	Time	Type	Area	Height	Width	Area%	Symmetry
1	2.799	MM	27.7	6.8	0.0681	1.970	0.83
2	2.971	MM	1379.2	482.8	0.0476	98.030	0.905

UV trace: **5**

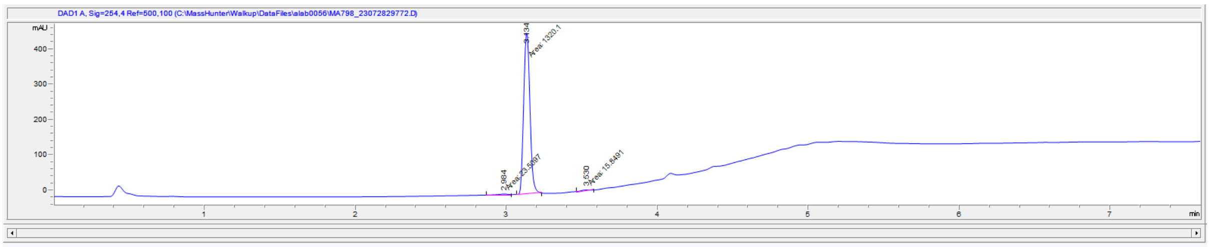

#	Time	Type	Area	Height	Width	Area%	Symmetry
1	2.984	MM	23.5	3.9	0.1016	1.729	1.387
2	3.134	MM	1320.1	463.4	0.0475	97.105	0.911
3	3.53	MM	15.8	3.3	0.0791	1.166	2.448

UV trace: **6**

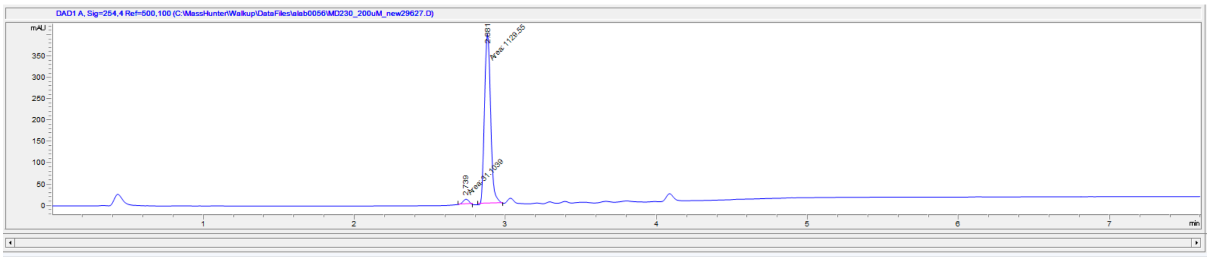

#	Time	Type	Area	Height	Width	Area%	Symmetry
1	2.739	MM	31.1	11.9	0.0434	2.680	1.124
2	2.881	MM	1129.6	402.8	0.0467	97.320	0.896

UV trace: **7**

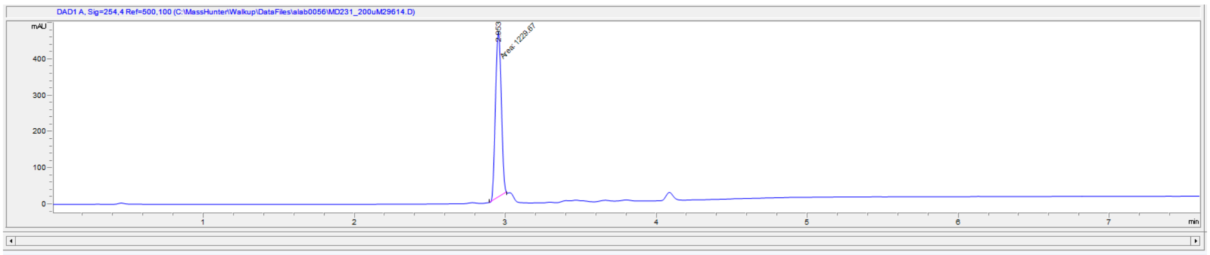

#	Time	Type	Area	Height	Width	Area%	Symmetry
1	2.953	MM	1229.7	461.9	0.0444	100.000	0.993

UV trace: **8**

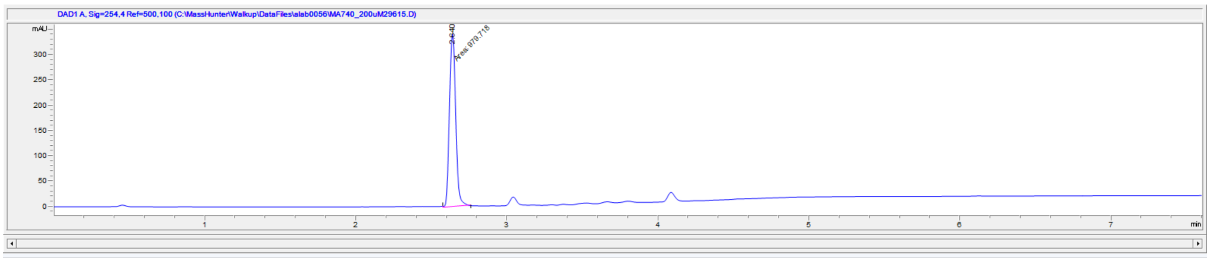

#	Time	Type	Area	Height	Width	Area%	Symmetry
1	2.64	MM	979.7	344.1	0.0475	100.000	0.919

UV trace: **10**

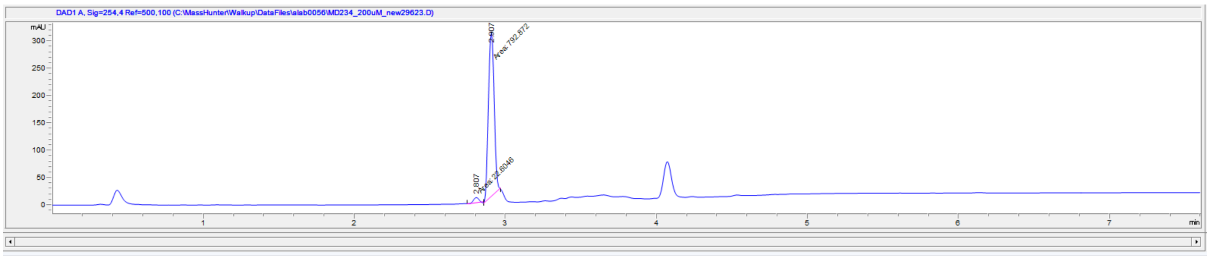

#	Time	Type	Area	Height	Width	Area%	Symmetry
1	2.807	MM	22.6	9.6	0.0394	2.772	1.228
2	2.907	MM	792.9	303	0.0436	97.228	1.01

UV trace: **11**

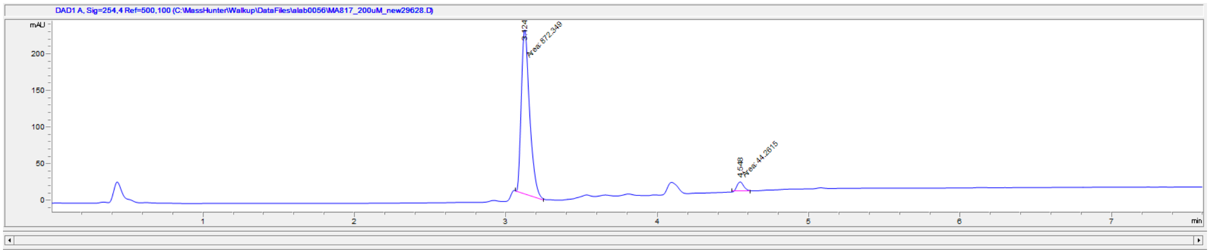

#	Time	Type	Area	Height	Width	Area%	Symmetry
1	3.124	MM	872.3	227.5	0.0639	95.171	0.662
2	4.548	MM	44.3	13.5	0.0545	4.829	0.792

UV trace: **25**

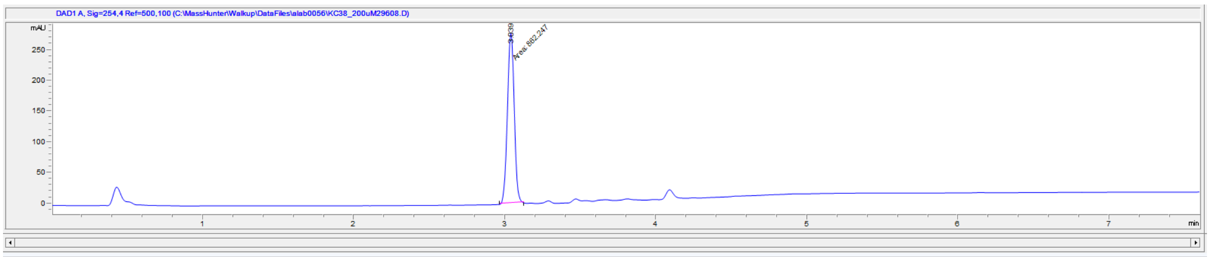

#	Time	Type	Area	Height	Width	Area%	Symmetry
1	3.039	MM	862.2	278.5	0.0516	100.000	1.01

UV trace: **26**

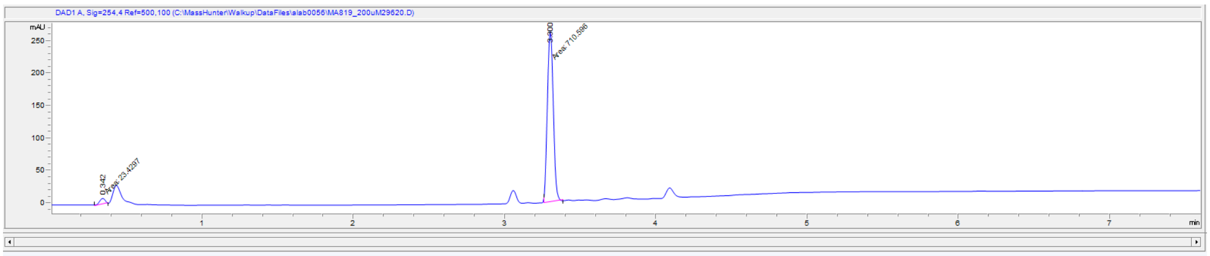

#	Time	Type	Area	Height	Width	Area%	Symmetry
1	0.342	MM	23.4	9.2	0.0425	3.192	1.36
2	3.3	MM	710.6	263.6	0.0449	96.808	0.939

UV trace: **27**

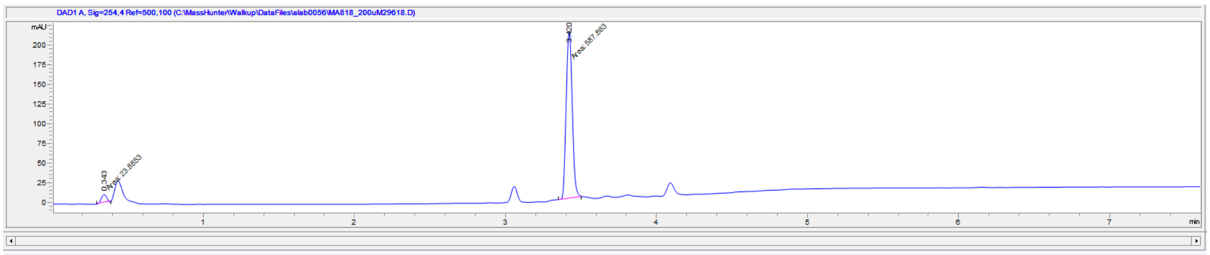

#	Time	Type	Area	Height	Width	Area%	Symmetry
1	0.343	MM	23.9	10.1	0.0392	3.904	1.256
2	3.42	MM	587.9	213.1	0.046	96.096	0.936

UV trace: **28**

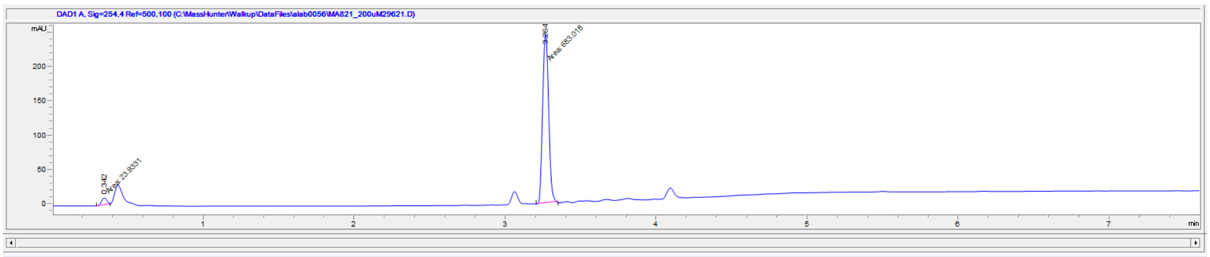

#	Time	Type	Area	Height	Width	Area%	Symmetry
1	0.342	MM	23.9	10	0.0399	3.385	1.21
2	3.264	MM	683	249.6	0.0456	96.615	0.929

UV trace: **29**

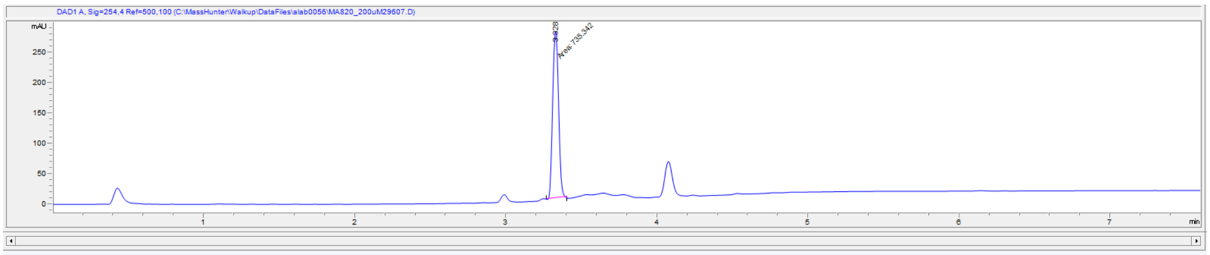

#	Time	Type	Area	Height	Width	Area%	Symmetry
1	3.328	MM	735.3	277.8	0.0441	100.000	0.962

References

(1) Maestro, Schrödinger, LLC, New York, NY, 2021.

(2) *MacroModel, Schrödinger, LLC, New York, NY, 2021.*

(3) Roos, K.; Wu, C.; Damm, W.; Reboul, M.; Stevenson, J. M.; Lu, C.; Dahlgren, M. K.; Mondal, S.; Chen, W.; Wang, L.; Abel, R.; Friesner, R. A.; Harder, E. D. OPLS3e: Extending Force Field Coverage for Drug-Like Small Molecules. *J. Chem. Theory Comput.* **2019**, *15* (3), 1863–1874. https://doi.org/10.1021/acs.jctc.8b01026.

(4) Becke, A. D. Density-Functional Exchange-Energy Approximation with Correct Asymptotic Behavior. *Phys. Rev. A* **1988**, *38* (6), 3098–3100. https://doi.org/10.1103/PhysRevA.38.3098.

(5) Lee, C.; Yang, W.; Parr, R. G. Development of the Colle-Salvetti Correlation-Energy Formula into a Functional of the Electron Density. *Phys. Rev. B* **1988**, *37* (2), 785–789. https://doi.org/10.1103/PhysRevB.37.785.

(6) Grimme, S.; Antony, J.; Ehrlich, S.; Krieg, H. A Consistent and Accurate Ab Initio Parametrization of Density Functional Dispersion Correction (DFT-D) for the 94 Elements H-Pu. *J. Chem. Phys.* **2010**, *132* (15), 154104. https://doi.org/10.1063/1.3382344.

(7) Bochevarov, A. D.; Harder, E.; Hughes, T. F.; Greenwood, J. R.; Braden, D. A.; Philipp, D. M.; Rinaldo, D.; Halls, M. D.; Zhang, J.; Friesner, R. A. Jaguar: A High-Performance Quantum Chemistry Software Program with Strengths in Life and Materials Sciences. *International Journal of Quantum Chemistry* **2013**, *113* (18), 2110–2142. https://doi.org/10.1002/qua.24481.

(8) Hamm, G. R.; Bäckström, E.; Brülls, M.; Nilsson, A.; Strittmatter, N.; Andrén, P. E.; Grime, K.; Fridén, M.; Goodwin, R. J. A. Revealing the Regional Localization and Differential Lung Retention of Inhaled Compounds by Mass Spectrometry Imaging. *J Aerosol Med Pulm Drug Deliv* **2020**, *33* (1), 43–53. https://doi.org/10.1089/jamp.2019.1536.

(9) Williams, D. P.; Lazic, S. E.; Foster, A. J.; Semenova, E.; Morgan, P. Predicting Drug-Induced Liver Injury with Bayesian Machine Learning. *Chem. Res. Toxicol.* **2020**, *33* (1), 239–248. https://doi.org/10.1021/acs.chemrestox.9b00264.

(10) Lyu, J.; Shao, R.; Kwong Yung, P. Y.; Elsässer, S. J. Genome-Wide Mapping of G-Quadruplex Structures with CUT&Tag. *Nucleic Acids Res* **2021**, *50* (3), e13. https://doi.org/10.1093/nar/gkab1073.

(11) Jamroskovic, J.; Doimo, M.; Chand, K.; Obi, I.; Kumar, R.; Brännström, K.; Hedenström, M.; Nath Das, R.; Akhunzianov, A.; Deiana, M.; Kasho, K.; Sulis Sato, S.; Pourbozorgi, P. L.; Mason, J. E.; Medini, P.; Öhlund, D.; Wanrooij, S.; Chorell, E.; Sabouri, N. Quinazoline Ligands Induce Cancer Cell Death through Selective STAT3 Inhibition and G-Quadruplex Stabilization. *J. Am. Chem. Soc.* **2020**, *142* (6), 2876–2888. https://doi.org/10.1021/jacs.9b11232.

(12) Dhameliya, T. M.; Chourasiya, S. S.; Mishra, E.; Jadhavar, P. S.; Bharatam, P. V.; Chakraborti, A. K. Rationalization of Benzazole-2-Carboxylate versus Benzazine-3-One/Benzazine-2,3-Dione Selectivity Switch during Cyclocondensation of 2-Aminothiophenols/Phenols/Anilines with 1,2-Biselectrophiles in Aqueous Medium. *J. Org. Chem.* **2017**, *82* (19), 10077–10091. https://doi.org/10.1021/acs.joc.7b01548.

(13) da Costa, E. P.; Coelho, S. E.; de Oliveira, A. H.; Araújo, R. M.; Cavalcanti, L. N.; Domingos, J. B.; Menezes, F. G. Multicomponent Synthesis of Substituted 3-Styryl-1H-Quinoxalin-2-Ones in an Aqueous Medium. *Tetrahedron Letters* **2018**, *59* (44), 3961–3964. https://doi.org/10.1016/j.tetlet.2018.09.048.

(14) Fabian, L.; Taverna Porro, M.; Gómez, N.; Salvatori, M.; Turk, G.; Estrin, D.; Moglioni, A. Design, Synthesis and Biological Evaluation of Quinoxaline Compounds as Anti-HIV Agents Targeting Reverse Transcriptase Enzyme. *European Journal of Medicinal Chemistry* **2020**, *188*, 111987. https://doi.org/10.1016/j.ejmech.2019.111987.
